# Trifluoromethylated Pyrazoles via Sequential (3 + 2)-Cycloaddition of Fluorinated Nitrile Imines with Chalcones and Solvent-Dependent Deacylative Oxidation Reactions

**DOI:** 10.1021/acs.orglett.2c00521

**Published:** 2022-03-28

**Authors:** Anna Kowalczyk, Greta Utecht-Jarzyńska, Grzegorz Mlostoń, Marcin Jasiński

**Affiliations:** † Department of Organic and Applied Chemistry, Faculty of Chemistry, University of Lodz, Tamka 12, 91403 Łódź, Poland; § The University of Lodz Doctoral School of Exact and Natural Sciences, Banacha 12/16, 90237 Łódź, Poland

## Abstract

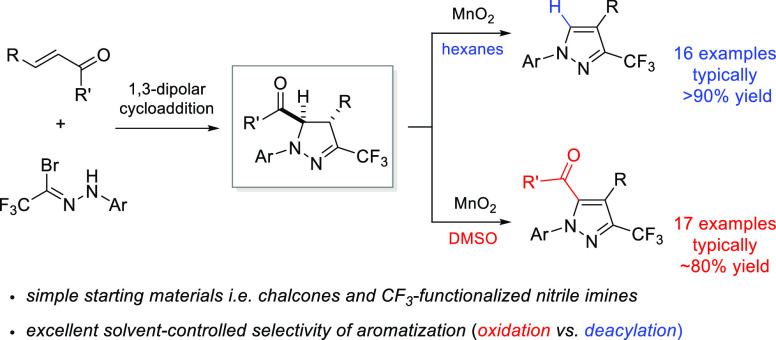

A general approach for preparation of two types of polyfunctionalized 3-trifluoromethylpyrazoles is reported. The protocol comprises (3 + 2)-cycloaddition of the *in situ* generated trifluoroacetonitrile imines with enones leading to *trans*-configured 5-acyl-pyrazolines in a fully regio- and diastereoselective manner. Initially formed cycloadducts were aromatized by treatment with manganese dioxide. Depending on the solvent used, the oxidation step either led to fully substituted pyrazoles (DMSO) or proceeded via a deacylative pathway to afford 1,3,4-trisubstituted derivatives (hexane) with excellent selectivity.

In the past two decades, great attention has been focused toward the chemistry of pyrazoles functionalized by introduction into the heterocyclic ring of either fluorine atom(-s) or fluoroalkylated groups.^1^ In a series of recent publications they were reported as organic materials of remarkable practical importance, and specifically 3-trifluoromethylated pyrazole has been indicated as a privileged structural scaffold for a variety of agrochemicals, pharmaceuticals, and advanced materials.^1,2^ For these reasons, development of new methods aimed at efficient and selective synthesis of multifunctionalized, fluorinated pyrazoles is a challenging problem in current organic synthesis.

In general, common access to 3-trifluoromethylpyrazoles relies on condensation of corresponding 1,3-dicarbonyl compounds (or their equivalents) with a functionalized hydrazines.^1−3^ In addition, Lewis acid mediated cyclizations and related transformations of hydrazones are also applied.^4^ Furthermore, some postcyclization, functional group interconversions leading to trifluoromethylated pyrazoles, and catalytic fluoroalkylations have been developed more recently.^5^ Another powerful approach is based on 1,3-dipolar cycloadditions employing trifluoromethylated 1,3-dipoles and appropriate dipolarophiles. In the past decade, remarkable progress has been achieved in the chemistry of 2,2,2-trifluorodiazoethane; however, some drawbacks such as difficult handling, low selectivity, and the scope limited to pyrazoles lacking a substituent at N(1) have been pointed out.^6^ In contrast, applications of alternative 1,3-dipolar intermediates, i.e. trifluoroacetonitrile imines **1**, offer access to *N*-functionalized heterocycles, and typically, their reactions proceed with excellent regio- and chemoselectivity.^7^ Nevertheless, application of easily accessible nitrile imines **1** for preparation of the title 3-trifluoromethylated pyrazoles remain underexplored.

Some time ago, Oh (but also our group) demonstrated that by using electron-rich C=C dipolarophiles such as enamines or vinyl ethers,^8^ the problem of low regioselectivity, reported by Tanaka in his pioneering work on 1,3-dipolar cycloadditions of **1** with nonactivated alkenes, could easily be overcome.^9^ As shown in Scheme 1, the presence of −NR_2_ or −OR as a leaving group in an ethylenic dipolarophile assures complete regioselectivity in the (3 + 2)-cycloaddition step and the initially formed products undergo either spontaneous or Brönsted acid induced elimination of an amine or alcohol molecule, respectively, to give the final aromatized heterocycle. More recently, Ma and co-workers developed an interesting one-pot decarboxylative (3 + 2)-cycloaddition route leading to fully substituted CF_3_-pyrazoles, starting with nitrile imines and isoxazolidinediones as dipolarophiles.^10^ In that case, thermal extrusion of CO_2_ from the corresponding intermediate was pointed out as a driving force leading to the final, aromatized product. Remarkably, neither of the methods developed thus far explores the orthogonal properties of the initially formed (3 + 2)-cycloadducts. Thus, in the search for new synthetic protocols toward polyfunctionalized 3-trifluoromethylpyrazoles, we envisioned possible access to three- and tetra-substituted analogues by using 5-acylpyrazolines as common precursors. The requisite starting materials can be obtained by employing azomethine imines as reported by Xie,^11^ but they should also be accessible via anticipated regioselective (3 + 2)-cycloaddition of acyclic enones with *in situ* generated fluorinated nitrile imines **1** (Scheme 1). Here we report on the efficient synthesis of two distinct classes of polysubstituted 3-trifluoromethylpyrazoles via a two-step protocol comprising (*i*) diastereoselective (3 + 2)-cycloaddition of **1** with chalcones followed by (*ii*) solvent-controlled, competitive oxidation vs deacylative aromatization of the intermediate pyrazolines by using MnO_2_ as a convenient oxidant.

**Scheme 1:**
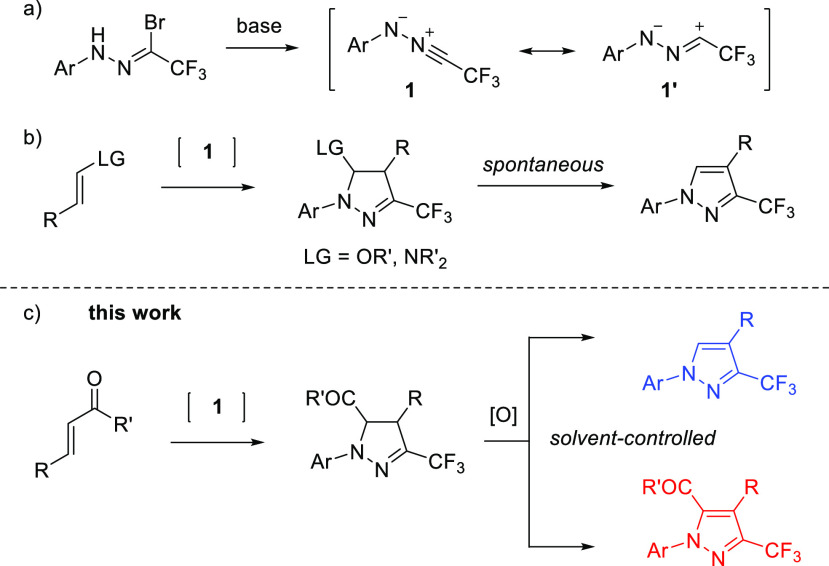
General Schemes of (a) Generation of Nitrile Imines **1**, (b) Their Reactions with Electron-Rich Alkenes, (c) and the Solvent-Controlled Synthesis of Polysubstituted 3-Trifluoromethylpyrazoles Reported Herein

The model 5-benzoylpyrazoline **2a** was prepared by the reaction of chalcone **4a** with an excess of hydrazonoyl bromide **3a** in the presence of Et_3_N as a base, at room temperature (Scheme 2).^7b^ Gratifyingly, the expected *trans-*configured pyrazoline **2a** was formed as the only product under the applied conditions. In the search for an efficient oxidizing reagent, we directed attention to MnO_2_ as a common oxidant which has broadly been applied, e.g. in diverse dehydrohalogenation processes.^12,13^ More importantly, despite its well-known mildness under neutral conditions, successful oxidation of some carbonyl compounds into respective carboxylic acids is also known.^14^ The first experiment was aimed at oxidation of model pyrazoline **2a** with excess MnO_2_ (ca. 85%, <10 μm), which was carried out in DCM solution, and the formation of a single product **5a** in ca. 37% yield was observed after 2 d at room temperature (Table 1, entry 1). Interestingly, in the ^13^C NMR (151 MHz, CDCl_3_) spectrum of 1,4-diphenyl-3-trifluoromethylpyrazole (**5a**), along with the expected quartets found at δ = 122.7 (^1^*J*_C–F_ = 269.9 Hz) and δ = 140.5 (^2^*J*_C–F_ = 36.6 Hz) attributed to the CF_3_ group and the C(3) atom, respectively, the presence of another quartet at δ = 128.8 (*J*_C–F_ ≈ 1.2 Hz) resulting from through-space coupling between F atoms and the *ortho*-C atoms of the neighboring Ph ring additionally confirmed the expected substitution pattern in **5a**.

**Scheme 2:**
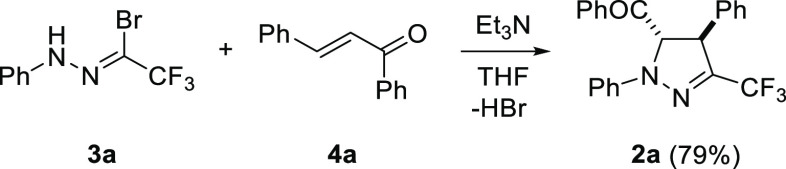
Synthesis of 3-Trifluoromethylpyrazoline **2a**

**Table 1:**
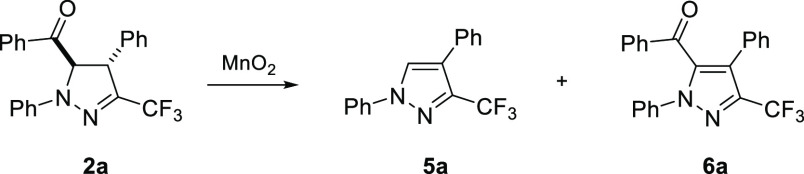
Oxidation of 3-Trifluoromethylpyrazoline **2a** with MnO_2_^a^

			ratio [%]^b^ (isolated yield)		
entry	solvent	temp	**2a**	**5a**	**6a**
1	DCM	rt	63	37	–
2	hexane	rt	46	54	–
3	toluene	rt	79	21	–
4	hexane	60 °C	–	96 (94)	4
5	hexane^c^	60 °C	–	98 (97)	2
6	THF	rt	89	11	–
7	MeCN	rt	90	10	–
8	DMSO	rt	100	–	–
9	MeCN	75 °C	–	53	47
10	DMF	100 °C	–	33	67
11	DMF	130 °C	–	35	65
12	DMSO	100 °C	–	7	93 (79)
13	DMSO^c^	100 °C	–	10	90 (81)
14	DMSO^d^	100 °C	–	8	92
15	DMSO^e^	100 °C	100	–	–

a Reaction conditions: a solution of **2a** (0.20 mmol) in corresponding solvent (3 mL) and solid MnO_2_ (20 equiv) were stirred magnetically in a 10 mL flask for 2 d.

b Estimated based on ^1^H NMR spectra of crude mixtures.

c 1 mmol (**2a**) scale.

d Reaction performed in the presence of atmospheric moisture (open flask).

e Heating in absence of MnO_2_.

Examination of the solvent effects revealed that decreased polarity of the organic medium favors deacylative oxidation leading to pyrazole **5a** (54% in hexane, entry 2), whereas only traces or no formation of this product was observed in THF, MeCN, and DMSO solutions.^15^ Increasing the temperature of the hexane solution resulted in complete conversion of starting pyrazoline **2a** into **5a** (96%) which was accompanied only by trace amounts of 5-benzoyl-functionalized pyrazole **6a** formed as a side product. On the other hand, oxidation of **2a** at elevated temperature in polar media such as MeCN, DMF, and DMSO proceeded partially with preservation of the benzoyl group and led to mixtures of **5a** and **6a** (entries 9–12). In the latter experiment performed in DMSO, preferential formation of the tetrasubstituted product was observed. Gratifyingly, both oxidation reactions could successfully be scaled up (1.0 mmol) without any remarkable loss of selectivity (entries 5 and 13). Furthermore, the optimized deacylative protocol was found to be operationally very simple; both the benzoic acid formed as the only byproduct and the remaining solid MnO_2_ could be filtered off to give, after removal of the solvent, spectroscopically pure product **5a**. Subsequent filtration of this material through a short silica gel pad provided analytically pure sample. The observed switch of chemoselectivity also deserves a brief comment. Possibly, the reaction carried out in the nonpolar hexane solution is initiated by oxidation at the benzyl-like position C(4) of the *trans*-configured pyrazoline **2** and proceeds preferentially via deacylative fashion due to close proximity of the benzoyl group and the “activated surface” of MnO_2_. Apparently, replacement of the nonpolar solvent by polar DMSO reduces the oxidative potential of MnO_2_,^12^ and hence, observed *trans* elimination of two H-atoms takes place.

With the optimized conditions in hand, we investigated the scope and limitations of the developed solvent-controlled oxidation procedure. Hence, a series of 5-benzoylpyrazolines **2b**–**2q** were prepared in analogy to the model reaction depicted in Scheme 2 in acceptable yields of 44–96%, and next, the obtained products **2** were subjected to reaction with MnO_2_ (Scheme 3; for detailed procedure, see Supporting Information). First, a series of pyrazolines **2b**–**2h**, derived from chalcone **4a** and differently substituted nitrile imines **1**, were examined in oxidation reactions.

**Scheme 3:**
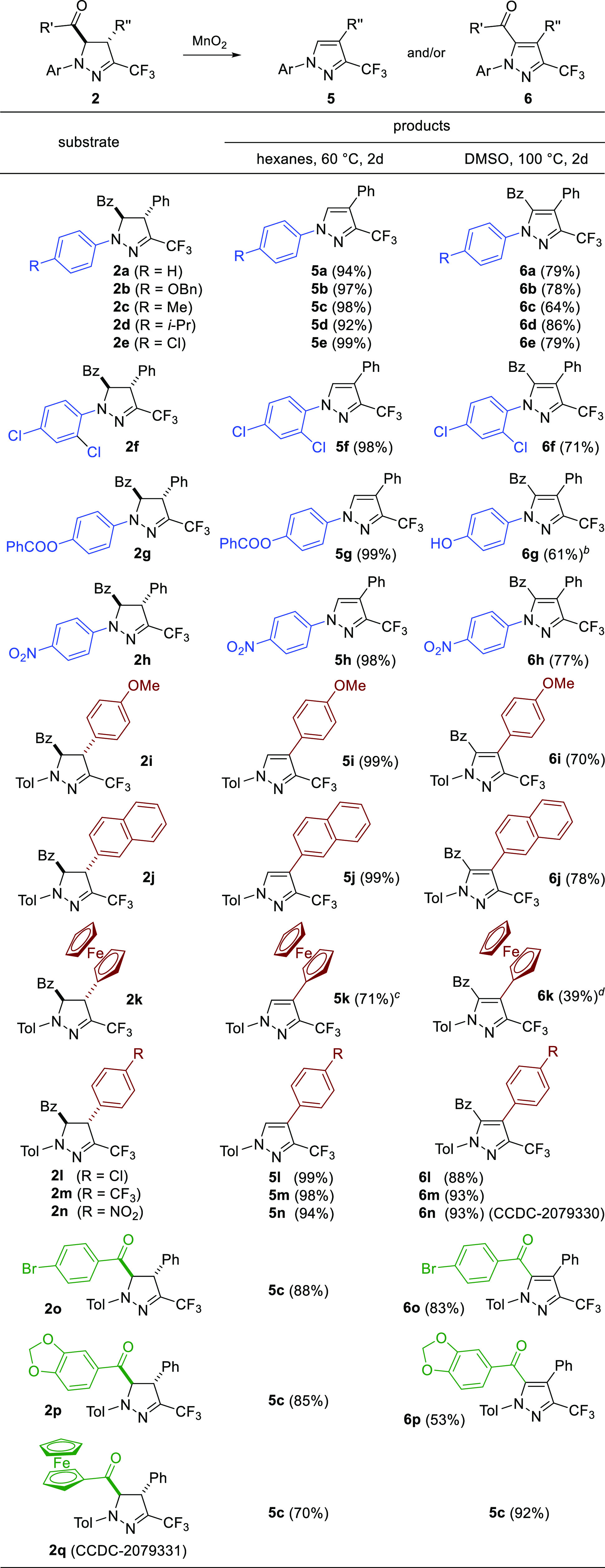
Oxidation of Pyrazolines **2** with MnO_2_; Scope of Substrates If not stated otherwise, the yields refer to isolated yields. Obtained from pyrazoline **2g**. The formation of **6k** (*ca*. 27% based on ^1^H NMR of crude mixture) was observed. The formation of **5k** (59%) was observed; yield estimated based on ^1^H NMR spectrum of crude mixture.

In all the tested examples, the expected products **5** and **6** were formed in high yields and with excellent selectivity, regardless of the electronic (OBn, NO_2_) and steric (2,4-di-Cl) features of the substituent present in the aryl ring located at N(1). Only in the case of 4-benzoyloxy derivative **2g** oxidation in hot DMSO proceeded with complete deprotection of the ester unit to afford phenol **6g** as the only product. Next, a second set of pyrazolines (**2i**–**2q**) obtained by condensation of differently substituted chalcones **4** with *p*-tolyl functionalized nitrile imine was examined. Again, excellent selectivity and high yields were noticed for this series except from the ferrocenyl-functionalized analogues **2k** and **2q**. In the first case, the presence of the redox-active Fc group located at C(4) interfered with complete selectivity of the oxidation to provide *ca*. 7:3 and *ca*. 6:4 mixtures of **5k** and **6k** in hexane and DMSO, respectively. On the other hand, introduction of ferrocenoyl unit at C(5) in pyrazoline **2q** favored debenzoylative aromatization to provide pyrazole **5c** as a major product in both experiments. The structures of two representative compounds in this series, **2q** and **6n**, were unambiguously confirmed by X-ray analysis.^16^

In order to demonstrate the essential role of the electron-withdrawing C=O group located at the C(5) in the formation of 1,4-disubstituted 3-trifluoromethylpyrazoles **5**, the stilbene-derived *trans*-pyrazoline **7** was synthesized and applied for the reaction with MnO_2_ in hexane (Scheme 4). In that case, the expected 1,4,5-triphenyl-3-trifluoromethylpyrazole (**8**, 90%) was obtained as the sole product after 2 d of heating at 60 °C. Next, (*E*)-4-phenyl-3-buten-2-one and methyl *trans*-cinnamate were also reacted with nitrile imine **1a** to yield the expected pyrazolines **9a** and **9b**, respectively. Subsequent treatment with MnO_2_ in hot hexane provided the known pyrazole **5a** lacking a substituent at C(5), hence indicating also methoxycarbonyl- and acetyl-functionalized pyrazolines as suitable substrates for the described deacylative aromatization reaction. Furthermore, two more bis-trifluoromethylated pyrazoles **5r** and **6r** were efficiently prepared via solvent-controlled oxidation starting with pyrazoline **2r** obtained via (3 + 2)-cycloaddition of nitrile imine **1c** with the known CF_3_-functionalized enone, namely, with (*E*)-4,4,4-trifluoro-1-phenyl-2-buten-1-one (Scheme 4).^17^ This result demonstrates again that electron-deficient nitrile imines **1** derived from trifluoroacetonitrile are very prone 1,3-dipoles which are able to react even with strongly electron-deficient dipolarophiles such as fluorinated thioamides,^7d^ and fluorinated enones. It is also worth noting that the presented protocol nicely supplements previously reported methods for the synthesis of rarely reported bis-trifluoromethylated pyrazoles, which are of interest in the context of not only pharmaceutical applications but also coordination chemistry.^1b,6d,18^

**Scheme 4:**
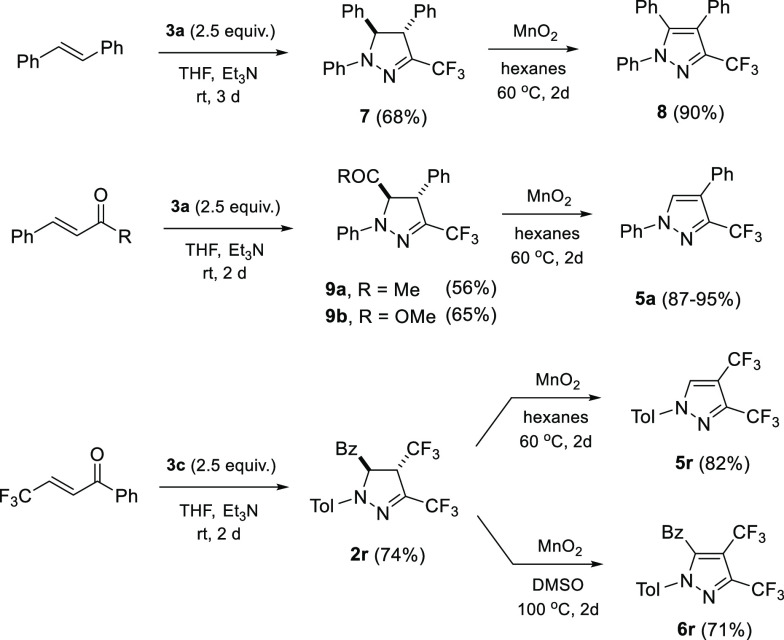
Control Experiments Aimed at Aromatization of Pyrazole Ring

In summary, a novel protocol for the synthesis of two types of 3-trifluoromethylated pyrazoles, by using 5-acylpyrazolines as common precursors for highly selective, solvent-dependent oxidative aromatization with MnO_2_, was elaborated and examined in a series of experiments. Starting pyrazolines are readily available via fully regio- and diastereoselective (3 + 2)-cycloaddition reactions starting with corresponding chalcones and hydrazonoyl bromides applied as precursors of the *in situ* generated fluorinated nitrile imines, derived from trifluoroacetonitrile. The reported method is scalable and characterized by a wide tolerance of functional groups. For all these reasons it can be recommended for preparation of polysubstituted 3-trifluoromethylpyrazoles which can be of potential interest, e.g. for medicinal chemistry, crop protection industry, and materials chemistry. The presented work demonstrates once more the utility of 1,3-dipolar cycloaddition reactions (the Huisgen reaction^19^) in method development for synthesis of trifluoromethylated heterocycles.^20^

## Supporting Information Available

FAIR data, including the primary NMR FID files, for compounds **2a**–**2r**, **5a**–**5r**, **6a**–**6r**, **7**, **8**, **9a**, and **9b** (ZIP)
Experimental procedures, characterization data and NMR spectra of all compounds (PDF)

## Accession Codes

CCDC 2079230–2079231 contain the supplementary crystallographic data for this paper. These data can be obtained free of charge via www.ccdc.cam.ac.uk/data_request/cif, or by emailing data_request@ccdc.cam.ac.uk, or by contacting The Cambridge Crystallographic Data Centre, 12 Union Road, Cambridge CB2 1EZ, UK; fax: +44 1223 336033.
