# Supplementary figures and images for: Trifluoromethylated Pyrazoles via Sequential (3 + 2)-Cycloaddition of Fluorinated Nitrile Imines with Chalcones and Solvent-Dependent Deacylative Oxidation Reactions
Source: Org Lett. 2022 Mar 28;24(13):2499–503. doi: 10.1021/acs.orglett.2c00521 (PMC9003577; doi:10.1021/acs.orglett.2c00521)

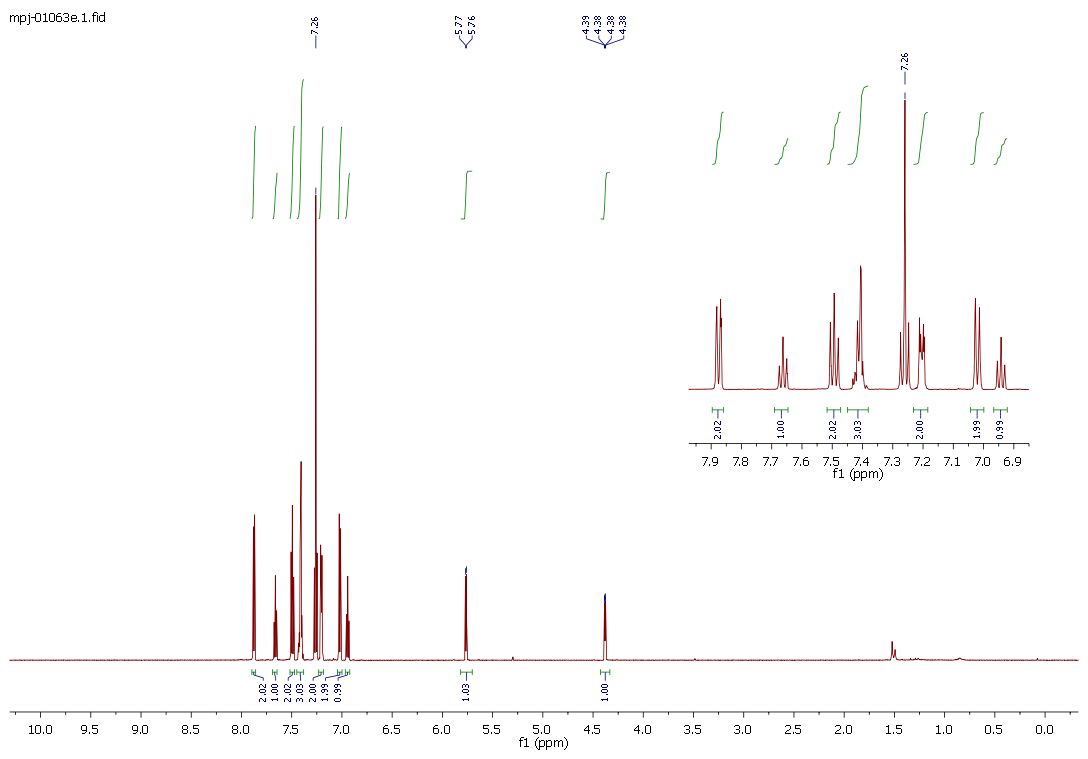

Supplement: Supplementary file 1 — ol2c00521_si_002.zip [file ol2c00521_si_002.zip › FIDs for publication/2a/1/1H trans.bmp]

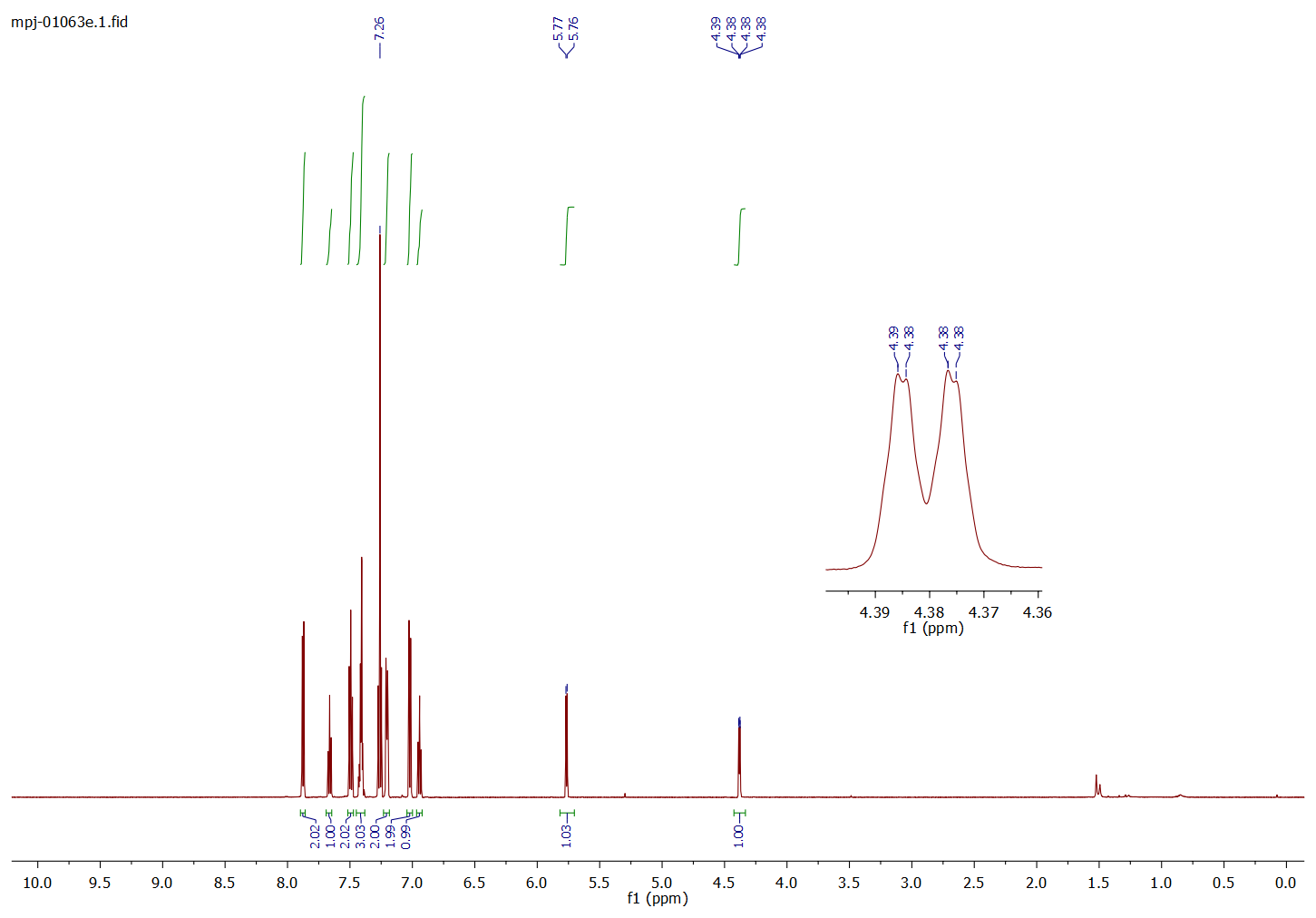

Supplement: Supplementary file 1 — ol2c00521_si_002.zip [file ol2c00521_si_002.zip › FIDs for publication/2a/1/2a 1h.png]

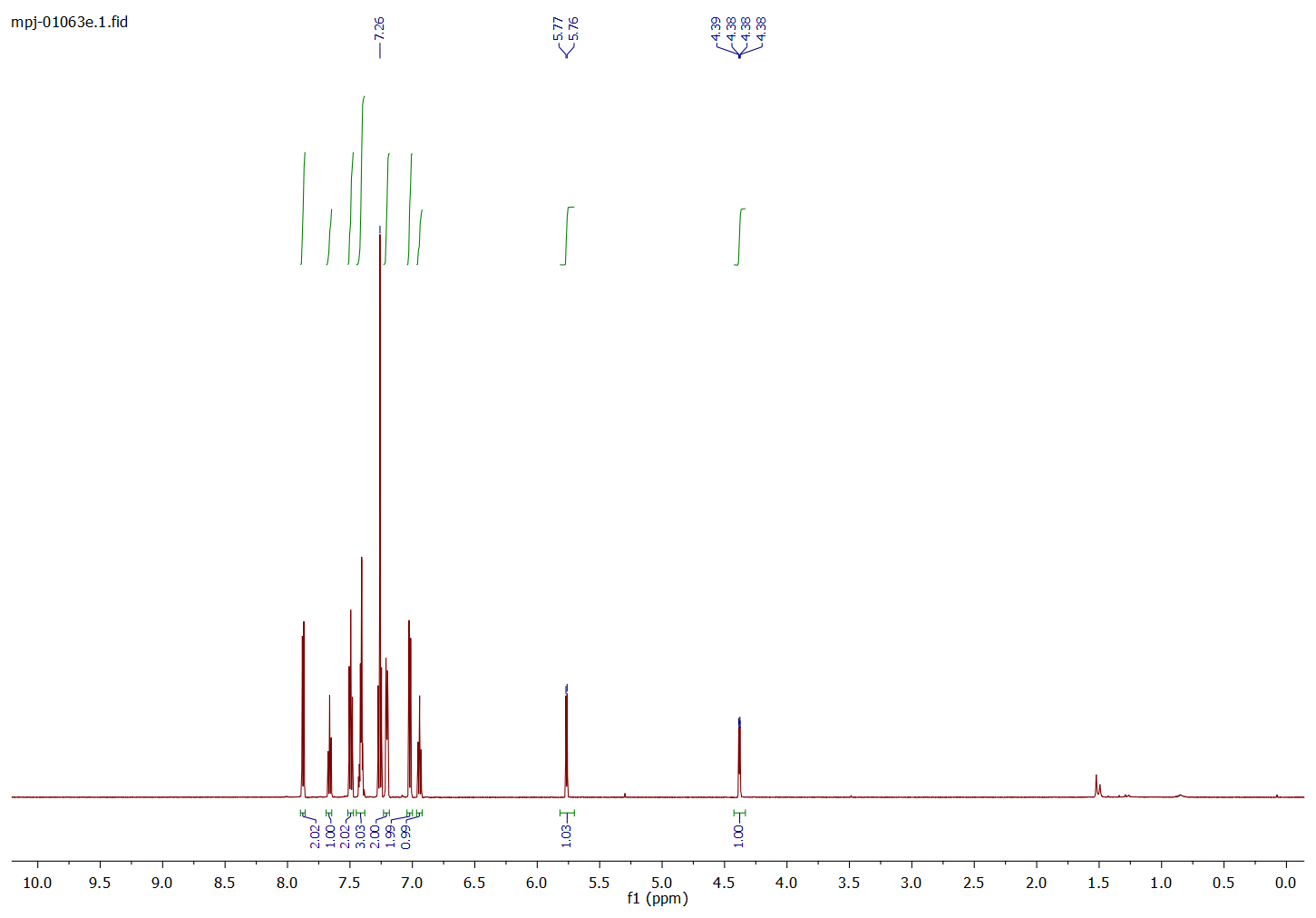

Supplement: Supplementary file 1 — ol2c00521_si_002.zip [file ol2c00521_si_002.zip › FIDs for publication/2a/1/trans 1H.png]

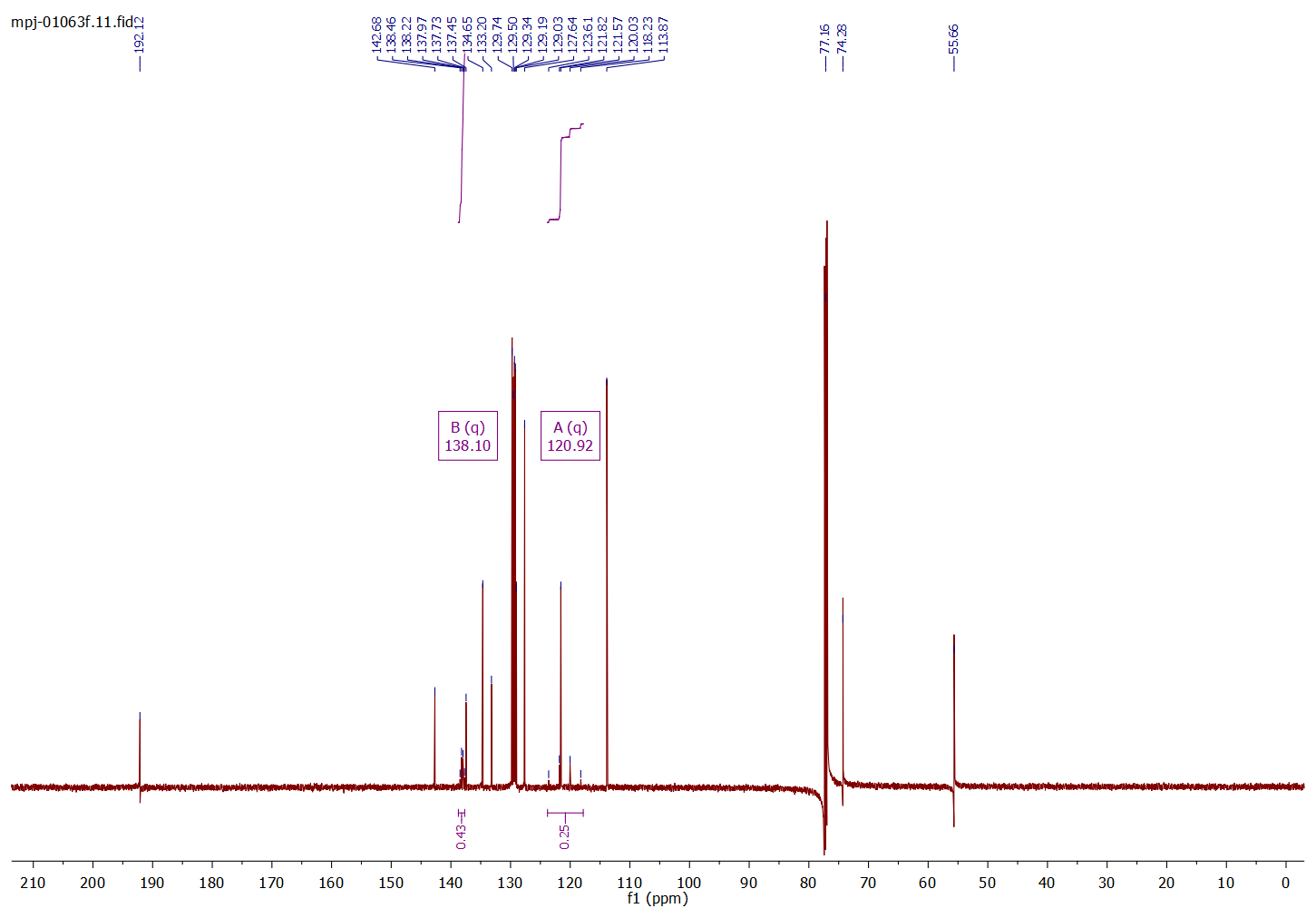

Supplement: Supplementary file 1 — ol2c00521_si_002.zip [file ol2c00521_si_002.zip › FIDs for publication/2a/11/2a 13c.png]

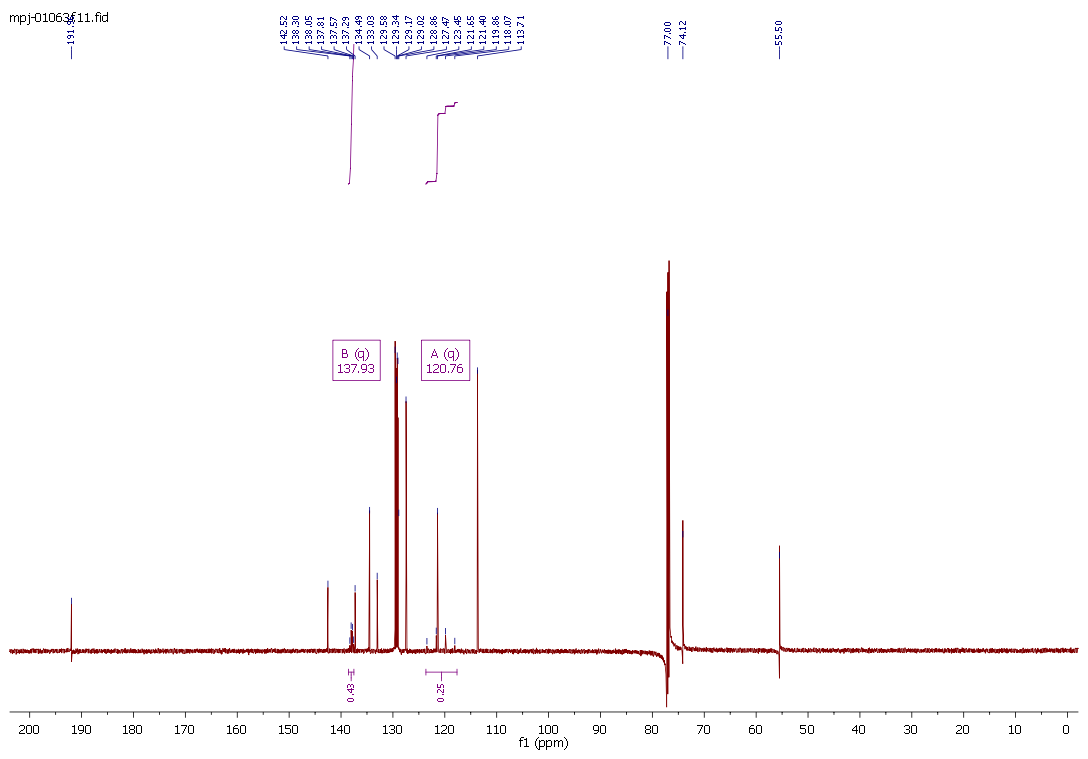

Supplement: Supplementary file 1 — ol2c00521_si_002.zip [file ol2c00521_si_002.zip › FIDs for publication/2a/11/trans 13C.bmp]

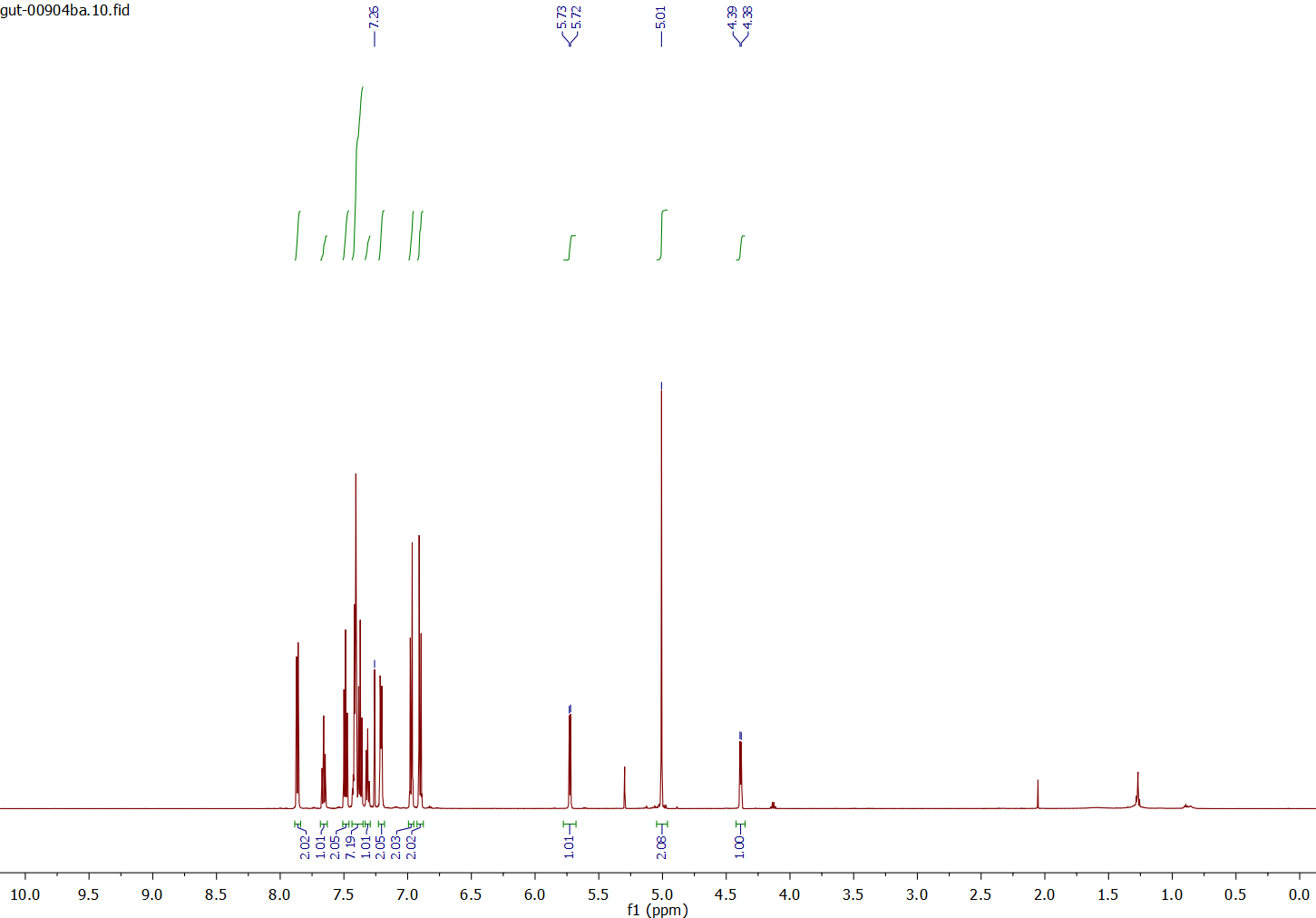

Supplement: Supplementary file 1 — ol2c00521_si_002.zip [file ol2c00521_si_002.zip › FIDs for publication/2b/10/2b 1h.png]

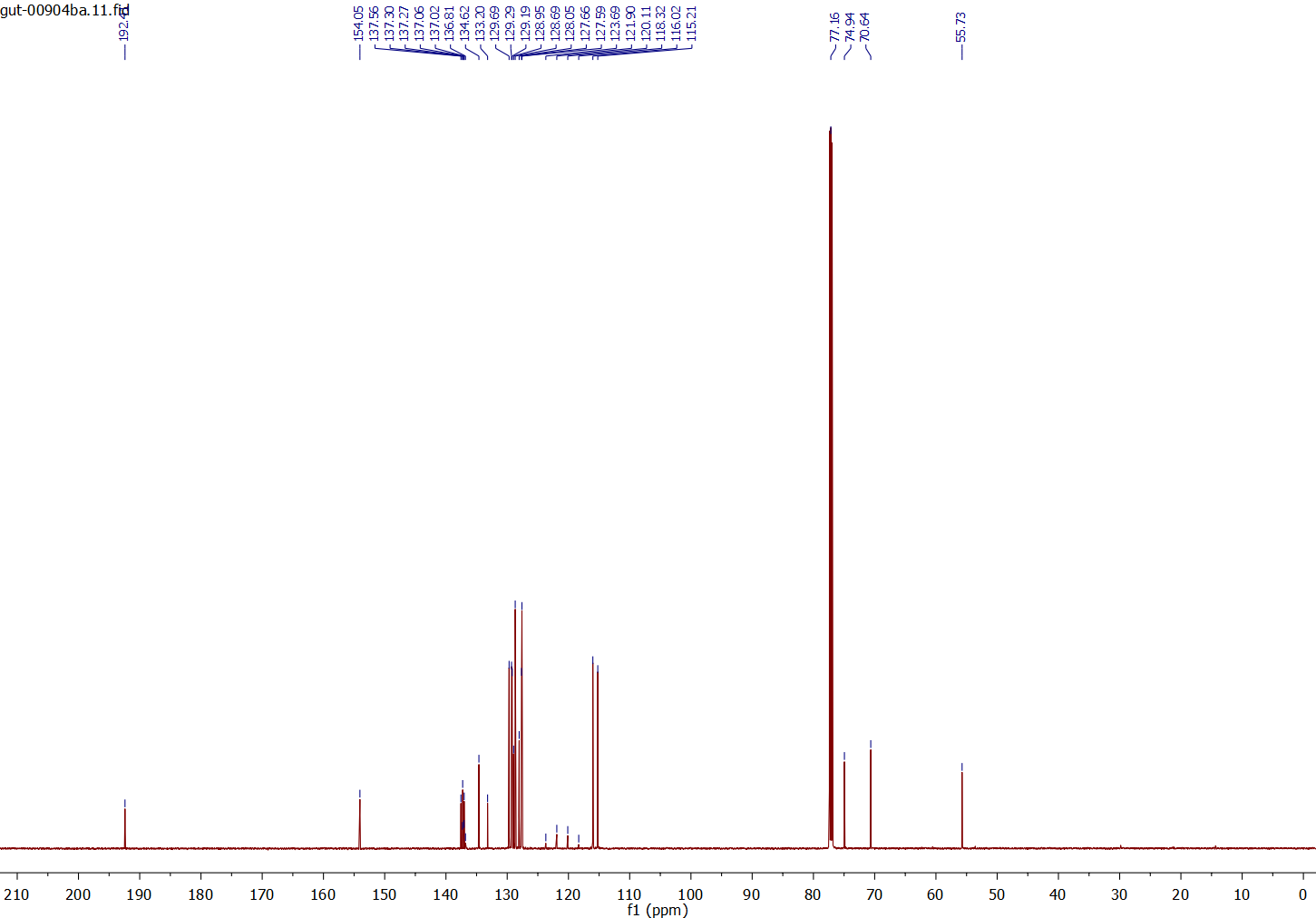

Supplement: Supplementary file 1 — ol2c00521_si_002.zip [file ol2c00521_si_002.zip › FIDs for publication/2b/11/2b 13c.png]

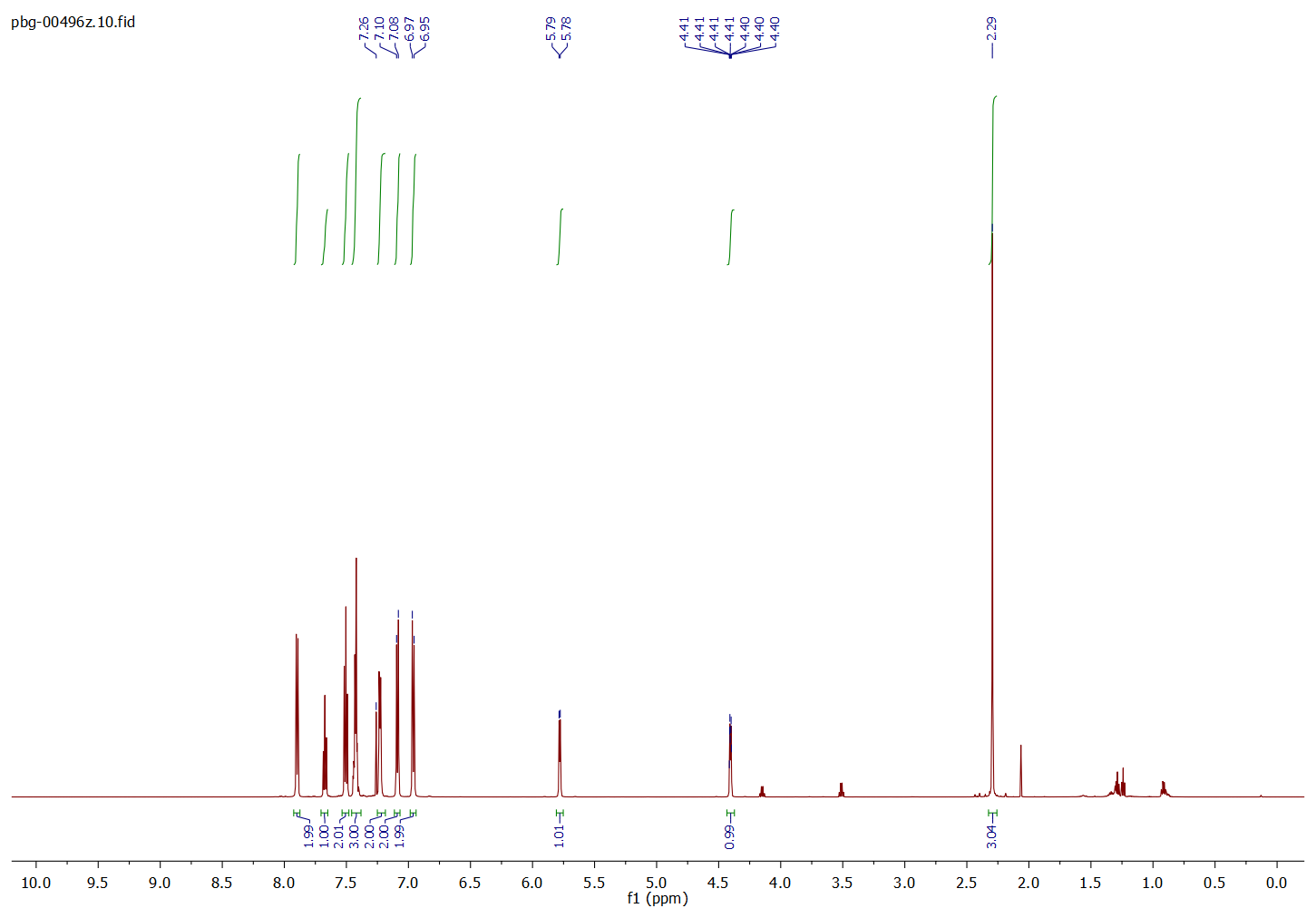

Supplement: Supplementary file 1 — ol2c00521_si_002.zip [file ol2c00521_si_002.zip › FIDs for publication/2c/10/2c 1h.png]

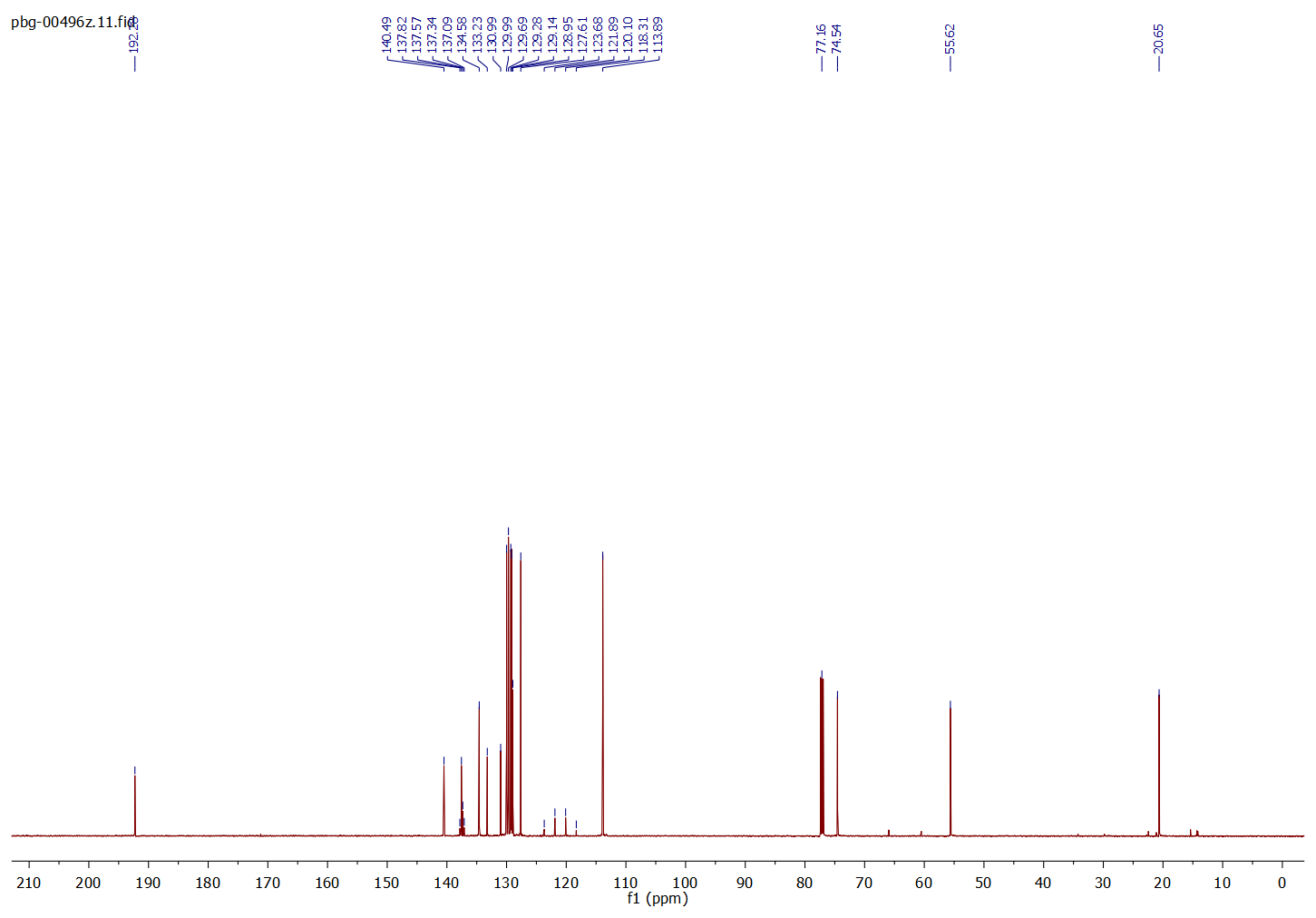

Supplement: Supplementary file 1 — ol2c00521_si_002.zip [file ol2c00521_si_002.zip › FIDs for publication/2c/11/2c 13c.png]

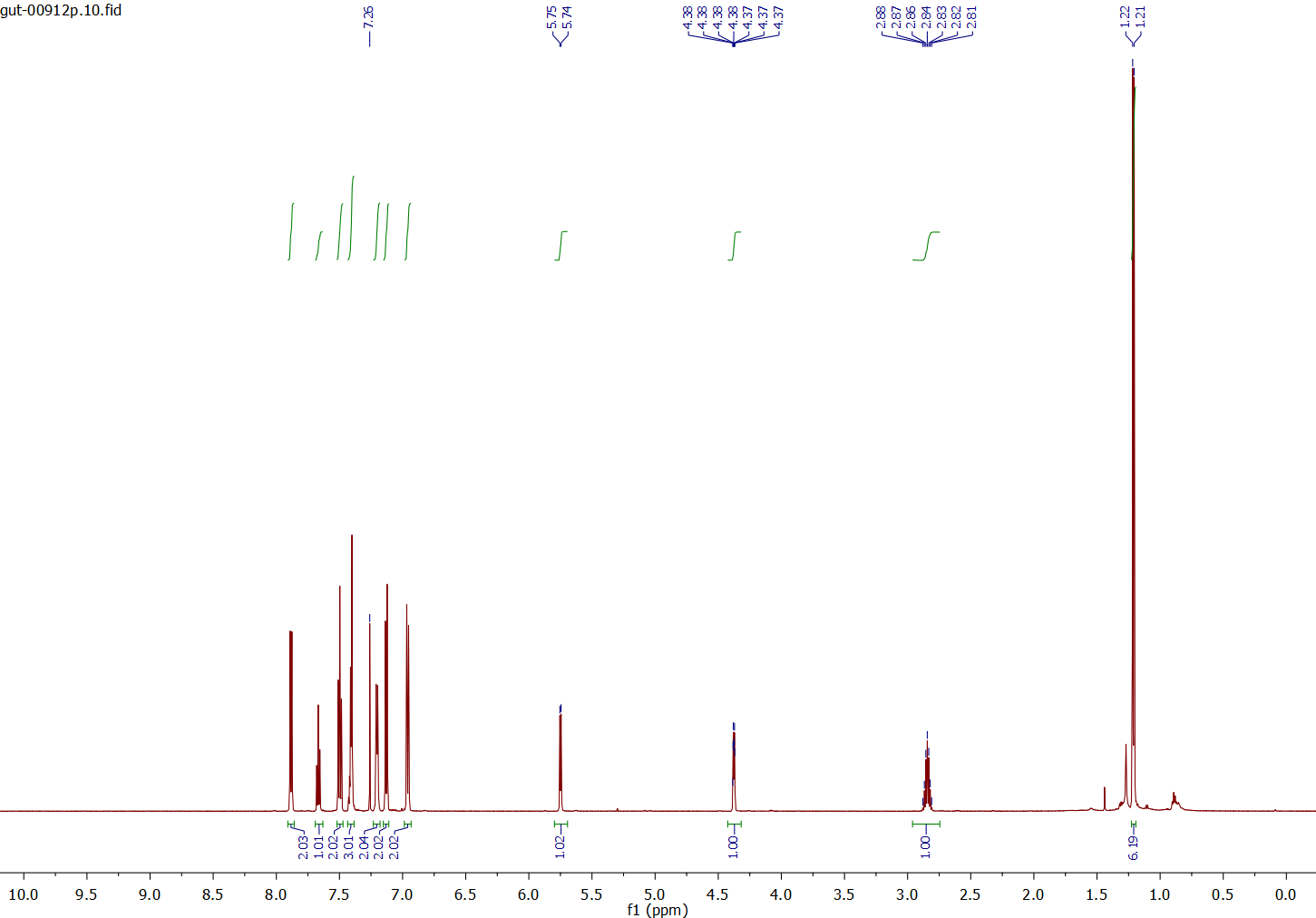

Supplement: Supplementary file 1 — ol2c00521_si_002.zip [file ol2c00521_si_002.zip › FIDs for publication/2d/10/4d 1h.png]

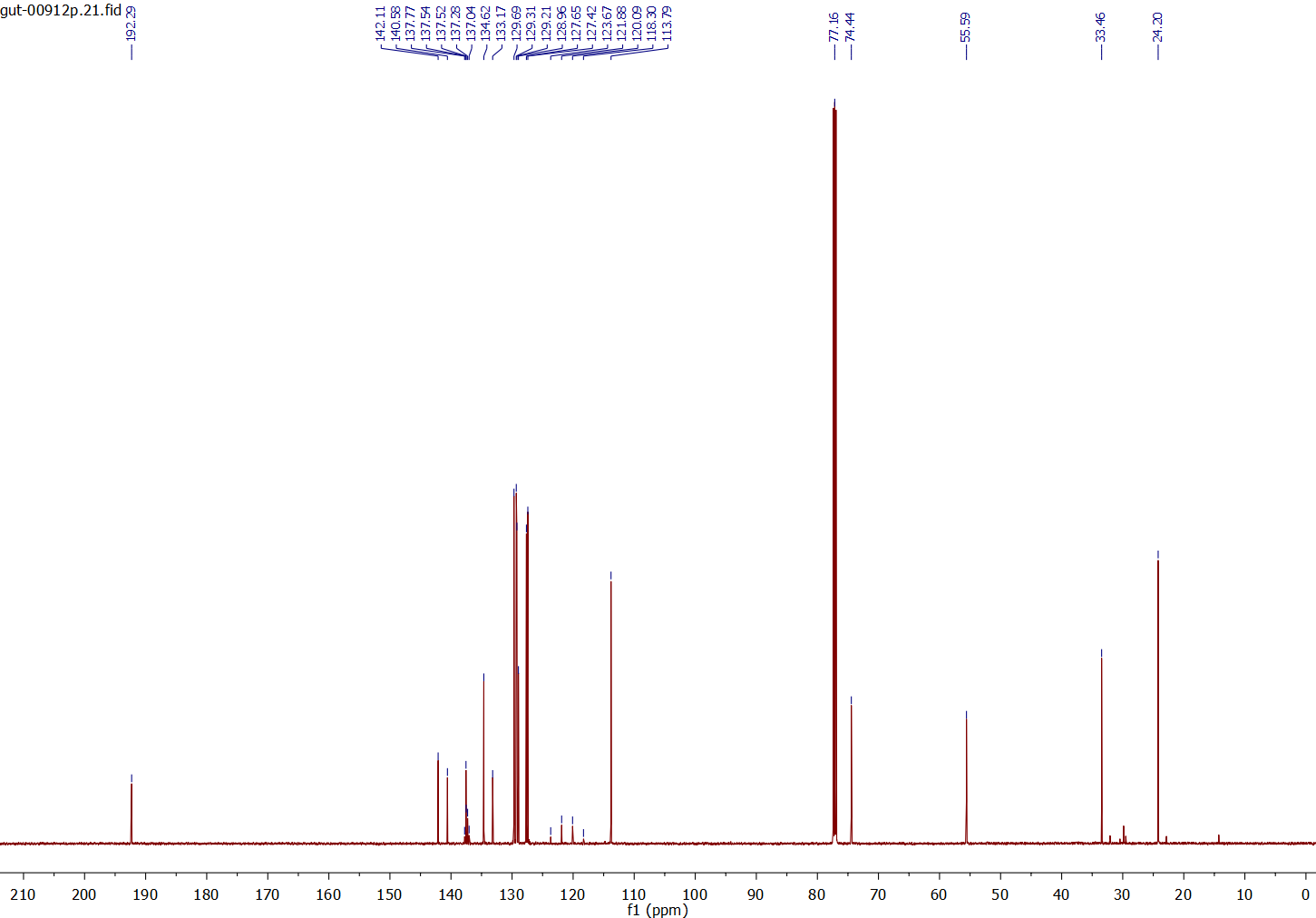

Supplement: Supplementary file 1 — ol2c00521_si_002.zip [file ol2c00521_si_002.zip › FIDs for publication/2d/21/4d 13c.png]

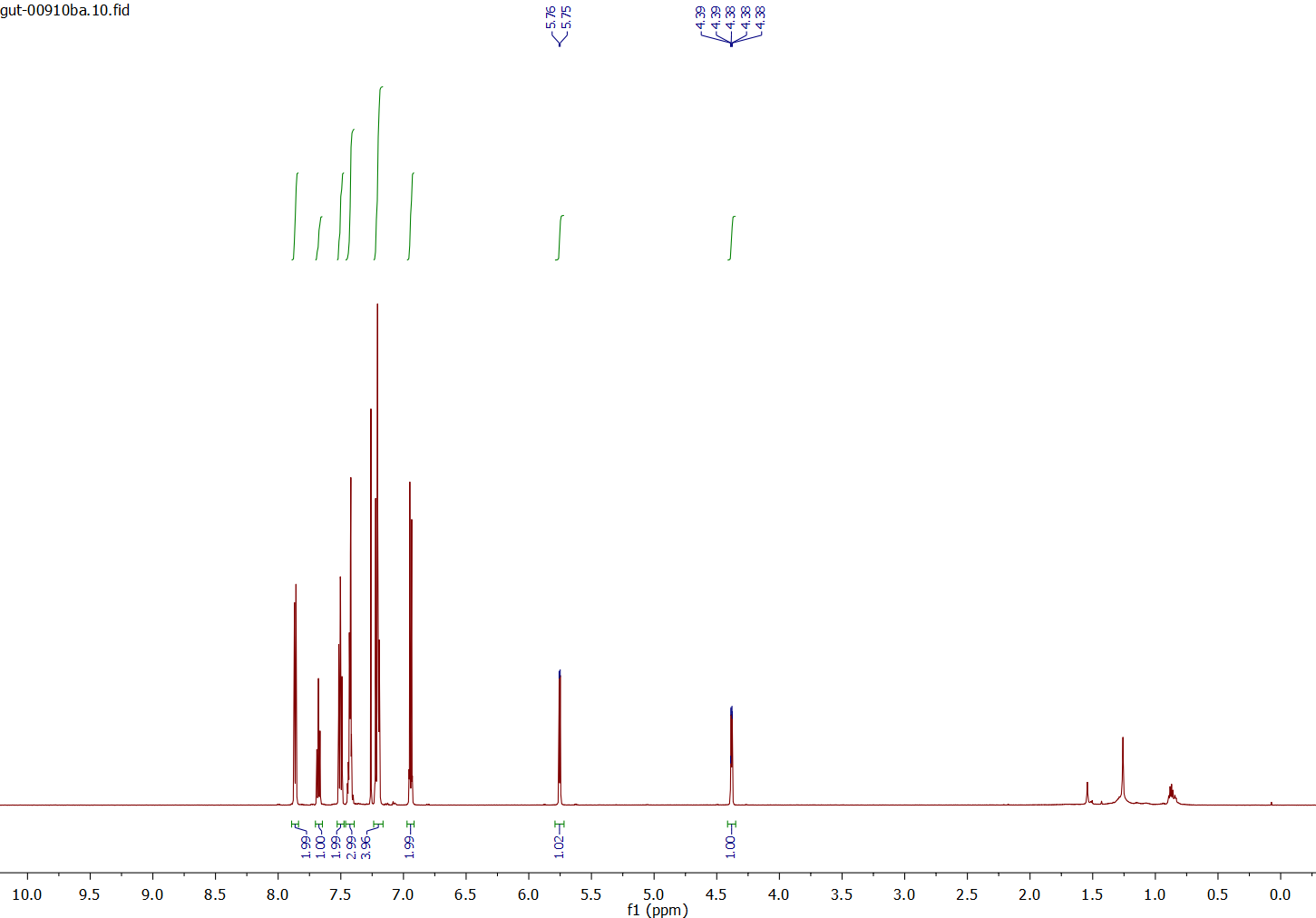

Supplement: Supplementary file 1 — ol2c00521_si_002.zip [file ol2c00521_si_002.zip › FIDs for publication/2e/10/2e 1h.png]

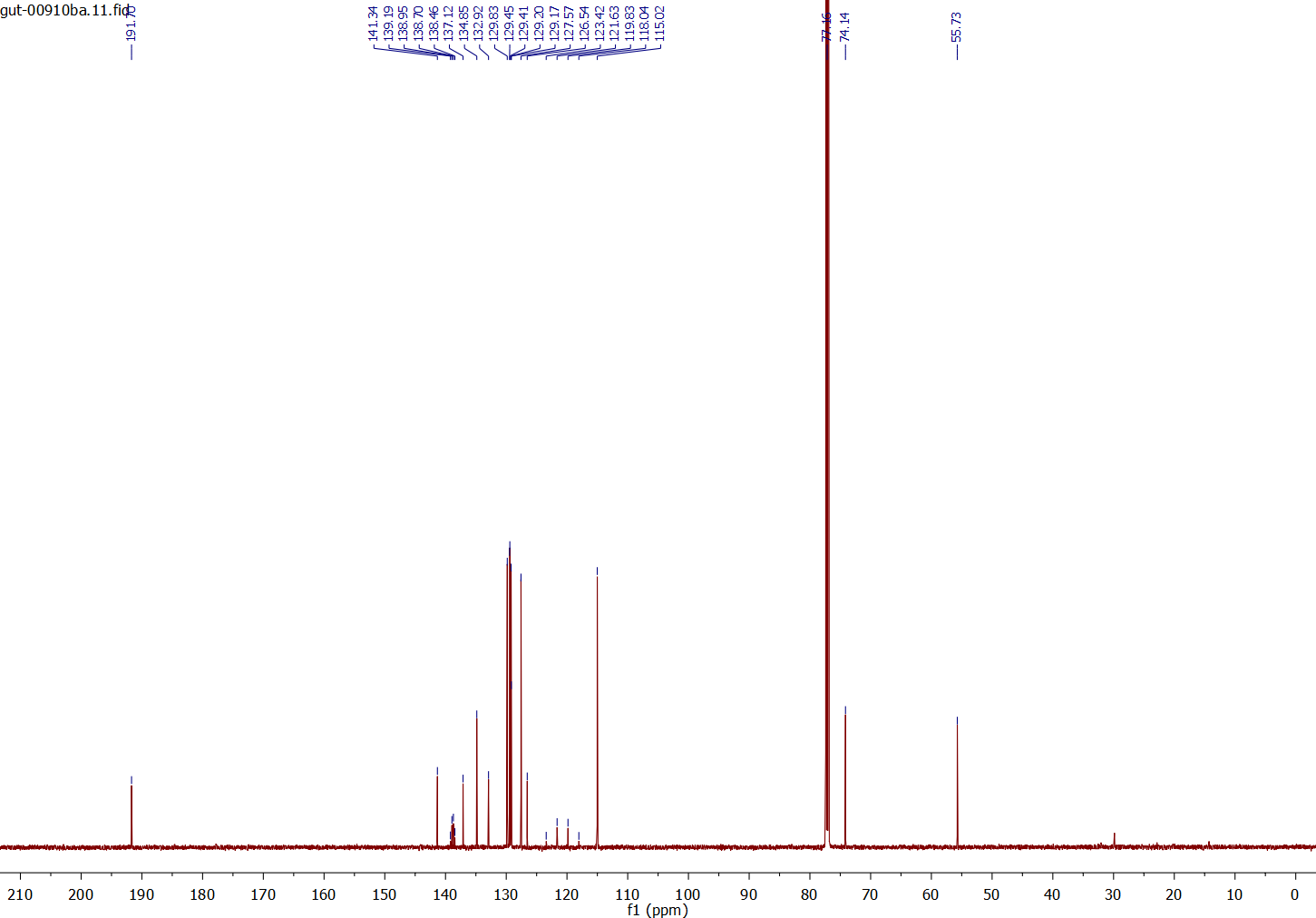

Supplement: Supplementary file 1 — ol2c00521_si_002.zip [file ol2c00521_si_002.zip › FIDs for publication/2e/11/2e 13c.png]

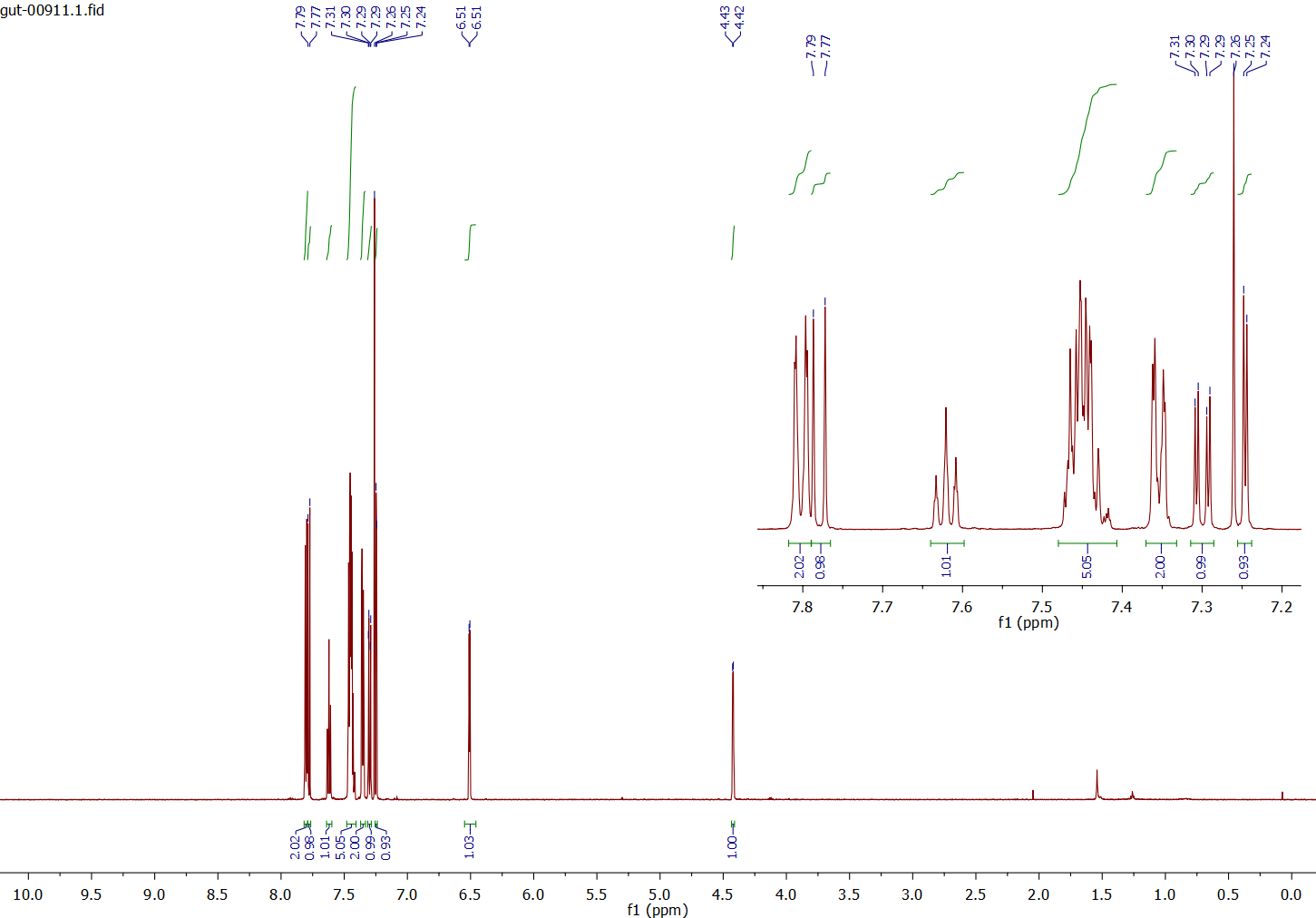

Supplement: Supplementary file 1 — ol2c00521_si_002.zip [file ol2c00521_si_002.zip › FIDs for publication/2f/1/2f 1h.png]

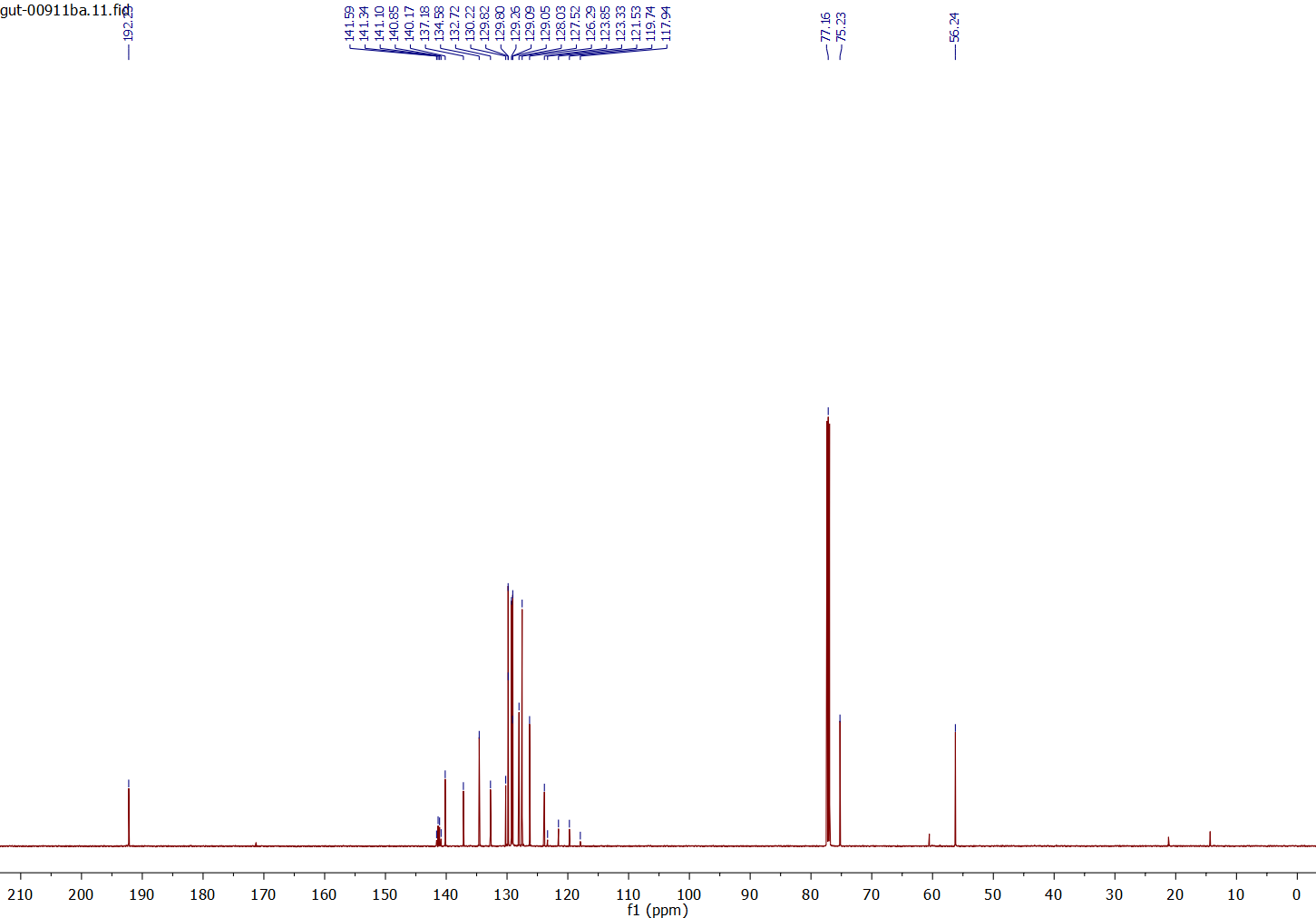

Supplement: Supplementary file 1 — ol2c00521_si_002.zip [file ol2c00521_si_002.zip › FIDs for publication/2f/11/2f 13c.png]

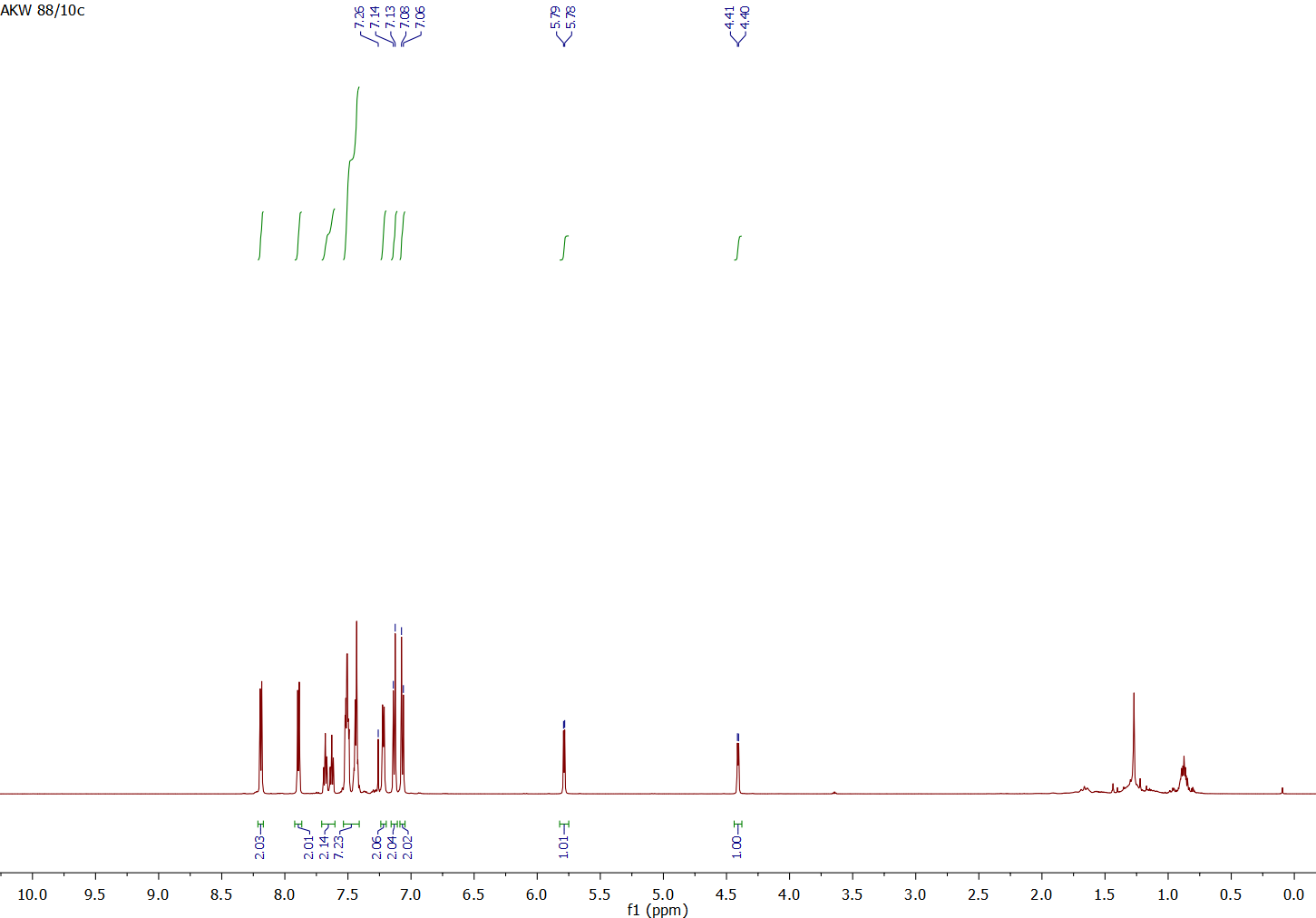

Supplement: Supplementary file 1 — ol2c00521_si_002.zip [file ol2c00521_si_002.zip › FIDs for publication/2g/1/2g 1h.png]

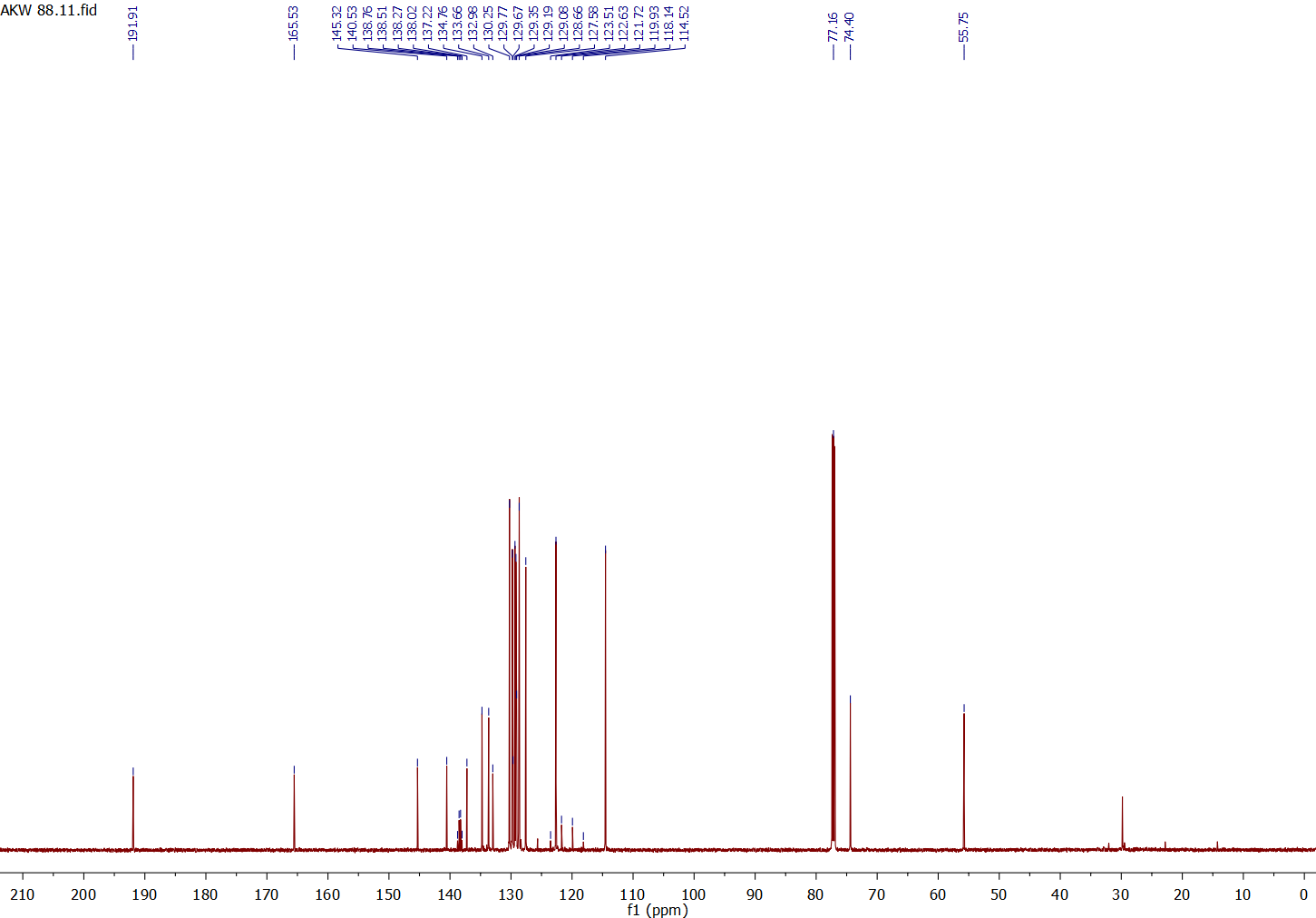

Supplement: Supplementary file 1 — ol2c00521_si_002.zip [file ol2c00521_si_002.zip › FIDs for publication/2g/11/2g 13c.png]

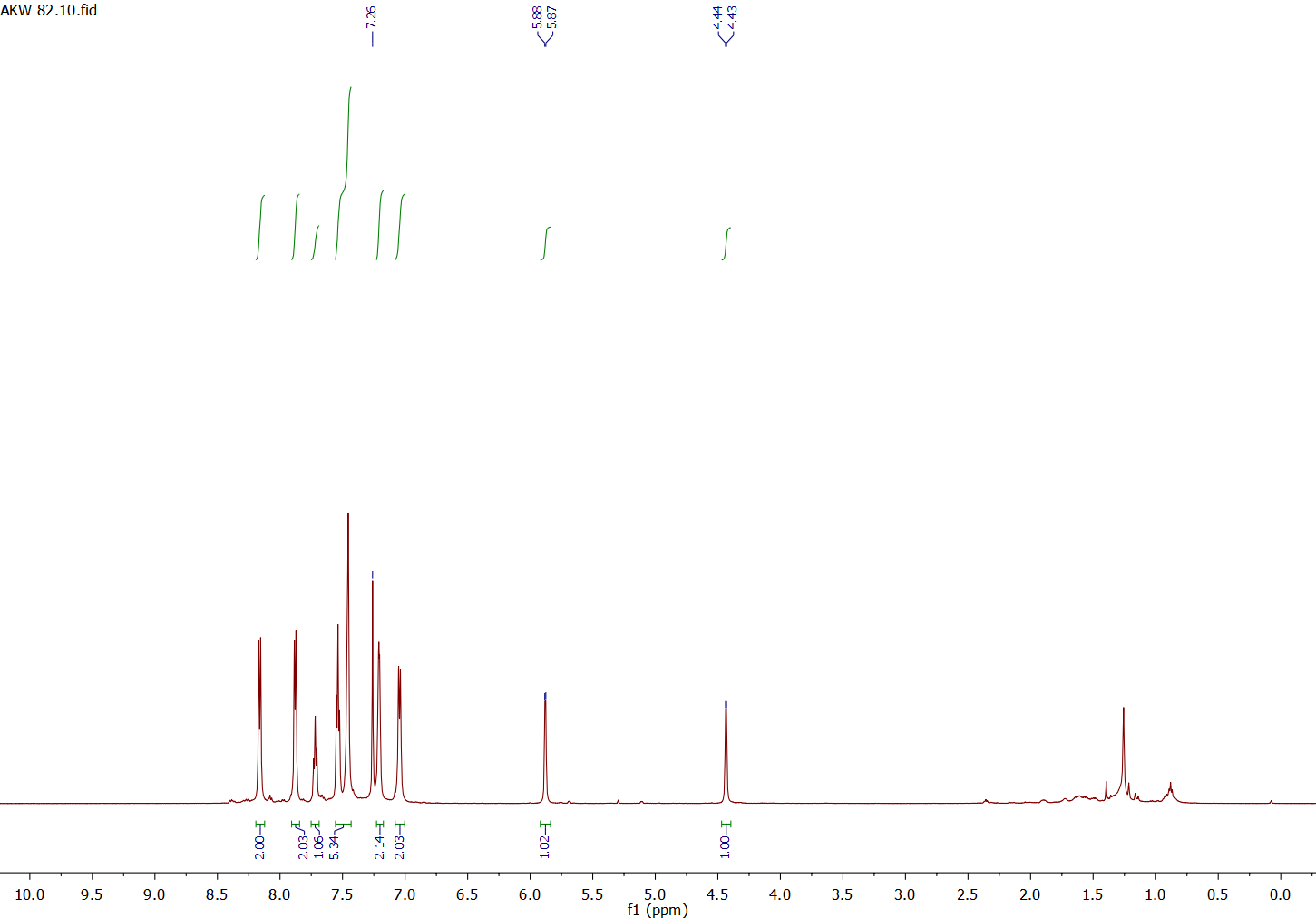

Supplement: Supplementary file 1 — ol2c00521_si_002.zip [file ol2c00521_si_002.zip › FIDs for publication/2h/10/2h 1h.png]
