# Supplementary material for: Trifluoromethylated Pyrazoles via Sequential (3 + 2)-Cycloaddition of Fluorinated Nitrile Imines with Chalcones and Solvent-Dependent Deacylative Oxidation Reactions
Source: Org Lett. 2022 Mar 28;24(13):2499–503. doi: 10.1021/acs.orglett.2c00521 (PMC9003577; doi:10.1021/acs.orglett.2c00521)
Supplement: Supplementary file 2 — ol2c00521_si_003.pdf [file ol2c00521_si_003.pdf]

Supporting Information

for

Trifluoromethylated pyrazoles via sequential (3+2)-cycloaddition of fluorinated nitrile imines with chalcones and solvent-dependent deacylative oxidation reactions

Anna Kowalczyk,[a,b,‡] Greta Utecht-Jarzyńska,[a,‡] Grzegorz Mlostoń[a], Marcin Jasiński[a,*]

[a] *Faculty of Chemistry, University of Lodz, Tamka 12, 91403 Łódź, Poland*

[b] *The University of Lodz Doctoral School of Exact and Natural Sciences, Banacha 12/16, 90237 Łódź, Poland*

[*] Corresponding author: Marcin Jasiński – *University of Lodz, Faculty of Chemistry, Łódź, Poland;* https://orcid.org/0000-0002-8789-9690; Phone: 48-42-6355766;

Email: marcin.jasinski@chemia.uni.lodz.pl

Content

1. General information S2

2. Synthetic procedures and characterization data S3

3. Copies of ^{1}H and ^{13}C NMR spectra S21

4. Crystallographic analyses S75

5. References S98

1. General information

Experimental procedures: If not stated otherwise, reactions were carried out under inert atmosphere of argon, in flame-dried flasks; subsequent manipulations were conducted in air. THF was dried over sodium/benzophenone and freshly distilled before use; dichloromethane was dried over CaH_2 and freshly distilled before use; other anhydrous solvents (hexane, toluene, DMF, MeCN, DMSO) were purchased and used as received. Products were purified by filtration through short silica gel plug or by standard column chromatography (CC) on silica gel (230–400 mesh) by using freshly distilled solvents as eluents or by recrystallization from appropriate solvents. The NMR spectra were taken on a Bruker AVIII instrument (^{1}H at 600 MHz, ^{13}C at 151 MHz, and ^{19}F at 565 MHz). Chemical shifts are reported relative to solvent residual peaks [for $CDCl_3$: ^{1}H NMR: δ = 7.26, ^{13}C NMR: δ = 77.16; for methanol-d$_4$ (CD_3OD): ^{1}H NMR: δ = 3.31, ^{13}C NMR: δ = 49.00][1] or to $CFCl_3$ (^{19}F NMR: δ = 0.00) used as external standard. Multiplicity of the signals in ^{13}C NMR spectra were deduced based on supplementary 2D measurements (HMQC, HMBC). The IR spectra were measured with an Agilent Cary 630 FTIR spectrometer, in neat. MS (ESI) were performed with a Varian 500-MS LC Ion Trap; high resolution MS (ESI-TOF) measurements were performed with a Synapt G2-Si mass spectrometer (Waters). Elemental analyses were obtained with a Vario EL III (Elementar Analysensysteme GmbH) instrument. Melting points were determined in capillaries with a MEL-TEMP apparatus (Aldrich) or with a polarizing optical microscope (Opta-Tech), and are uncorrected. Mechanochemical reactions were performed by using Retsch Mixer Mill MM400.

Starting materials: The nitrile imines precursors, *i.e.* hydrazonoyl bromides **3** were obtained by NBS-mediated electrophilic bromination of the corresponding trifluoroacetaldehyde arylhydrazones, in dry DMF at room temperature following general protocol.[2] The starting fluoral hydrazones were obtained according to a general literature procedure by condensation of aqueous fluoral hydrate (~75% in H_2O) with commercially available hydrazines in a closed ampoule at 75 °C, in methanol, in the presence of molecular sieves 4Å.[3] Chalcones **4** were purchased or prepared via Claisen–Schmidt condensation by using appropriate aldehydes and methyl ketones, in ethanol.[4] (*E*)-4,4,4-Trifluoro-1-phenyl-2-buten-1-one was prepared as described.[5] Activated MnO_2 (ca. 85%, <10 μm) was purchased (Sigma-Aldrich, product no. 217646-100G) and used as received (Figure S1). All the other commercially available solvents and starting materials were used as received.

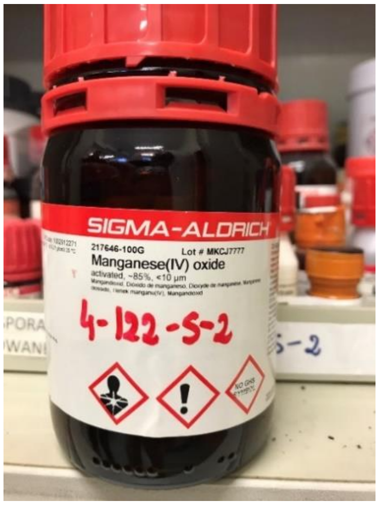

Figure S1. Activated MnO_2 (ca. 85%, <10 μm; Sigma-Aldrich product no. 217646-100G) used in this study.

2. Synthetic procedures and characterization data

General procedure for synthesis of *trans*-pyrazolines 2a-2q, 7 and 9a,b: To a solution of the respective enone or stilbene (1.0 mmol) in a mixture of dry Et_3N (2.0 mL) and dry THF (5.0 mL) was added hydrazonoyl bromide **3** (2.5 equiv., in two equal portions; the second portion added after 24 h) and the stirring was continued at room temperature until the starting olefin was fully consumed (TLC monitoring, typically 2-4 days). The precipitates were filtered off and the solvents were removed under reduced pressure. The product was isolated by standard column chromatography (CC) using silica gel as the stationary phase and petroleum ether/dichloromethane or petroleum ether/EtOAc mixtures as an eluent.

trans-5-Benzoyl-1,4-diphenyl-3-trifluoromethyl-4,5-dihydro-1*H*-pyrazole (**2a**)[6]:

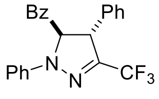

2a

CC (SiO_2, petroleum ether/EtOAc 95:5): pale yellow solid, 311 mg (79%); mp 160–161 °C. ^{1}H NMR (600 MHz, $CDCl_3$) δ 4.38 (dq, $^4J_{H\text{-}F} \approx 0.9$ Hz, $J_{H\text{-}H} = 5.6$ Hz, 1H, 4-H), 5.76 (d, $J_{H\text{-}H} = 5.6$ Hz, 1H, 5-H), 6.93-6.97, 7.02-7.05, 7.20-7.23, 7.25-7.29, 7.39-7.44, 7.48-7.52, 7.65-7.68, 7.87-7.89 (8m, 1H, 2H, 2H, 2H, 3H, 2H, 1H, 2H). ^{13}C NMR (151 MHz, $CDCl_3$) δ 55.7, 74.3, 113.9, 120.9 (q, $^1J_{C\text{-}F} = 270.6$ Hz, CF_3), 121.6, 127.7, 129.0, 129.2, 129.3, 129.5, 129.7, 133.2, 134.7, 137.5, 138.1 (q, $^2J_{C\text{-}F} = 37.0$ Hz, C-3), 142.7, 192.1. ^{19}F NMR (565 MHz, $CDCl_3$) δ –63.0 (s_{br}, CF_3). IR (neat) ν 1690, 1595, 1297, 1131 cm^{-1}. ESI-MS (*m/z*): 417.2 (100, $[M+Na]^+$), 395.1 (23, $[M+H]^+$). Anal. calcd for $C_{23}H_{17}F_3N_2O$ (394.1): C 70.04, H 4.34, N 7.10; found: C 70.21, H 4.34, N 7.32.

trans-5-Benzoyl-1-(4'-benzyloxyphenyl)-4-phenyl-3-trifluoromethyl-4,5-dihydro-1*H*-pyrazole (**2b**):

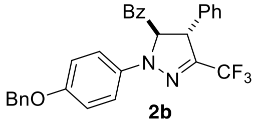

2b

CC (SiO_2, petroleum ether/EtOAc 95:5): light orange solid, 285 mg (57%); mp 62–63 °C. ^{1}H NMR (600 MHz, $CDCl_3$) δ 4.39 (d_{br}, $J = 5.8$ Hz, 1H, 4-H), 5.01 (s, 2H, CH_2O), 5.73 (d, $J = 5.8$ Hz, 1H, 5-H), 6.89-6.92, 6.96-6.99, 7.20-7.22, 7.30-7.33, 7.35-7.43, 7.47-7.50, 7.64-7.67, 7.85-7.88 (8 m, 2H, 2H, 2H, 1H, 7H, 2H, 1H, 2H). ^{13}C NMR (151 MHz, $CDCl_3$) δ 55.7, 70.6, 74.9, 115.2, 116.0, 121.0 (q, $^1J_{C\text{-}F} = 270.3$ Hz, CF_3), 127.6, 127.7, 128.1, 128.7, 129.0, 129.2, 129.3, 129.7, 133.2, 134.6, 137.0, 137.2 (q, $^2J_{C\text{-}F} = 36.8$ Hz, C-3), 137.3, 137.6, 154.1, 192.4. ^{19}F NMR (565 MHz, $CDCl_3$) δ –62.3 (s_{br}, CF_3). IR (neat) ν 1694, 1510, 1230, 1120 cm^{-1}. ESI-MS (*m/z*): 501.5 (100, $[M+H]^+$). Anal. calcd for $C_{30}H_{23}F_3N_2O_2$ (500.2): C 71.99, H 4.63, N 5.60; found: C 71.96, H 4.66, N 5.81.

trans-5-Benzoyl-1-(4'-tolyl)-4-phenyl-3-trifluoromethyl-4,5-dihydro-1*H*-pyrazole (**2c**)[7]:

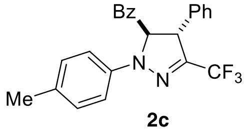

2c

CC (SiO_2, petroleum ether/EtOAc 95:5): light yellow solid, 392 mg (96%); mp 145–146 °C. ^{1}H NMR (600 MHz, $CDCl_3$) δ 2.29 (s, 3H, Me), 4.38 (dq, $^4J_{H\text{-}F} \approx 1.0$ Hz, $J_{H\text{-}H} \approx 5.7$ Hz, 1H, 4-H),

5.78 (d_{br}, $J \approx 5.7$ Hz, 1H, 5-H), 6.96, 7.09 (2 d, $J = 8.6$ Hz, 2H each), 7.21-7.25, 7.40-7.45, 7.49-7.52, 7.66-7.69, 7.88-7.91 (5 m, 2H, 3H, 2H, 1H, 2H). ^{13}C NMR (151 MHz, $CDCl_3$) δ 20.7, 55.6, 74.5, 113.9, 121.0 (q, $^{1}J_{C\text{-}F} = 270.3$ Hz, CF_3), 127.6, 129.0, 129.1, 129.3, 129.7, 130.0, 131.0, 133.2, 134.6, 137.4 (q, $^{2}J_{C\text{-}F} = 36.8$ Hz, C-3), 137.6, 140.5, 192.3. ^{19}F NMR (565 MHz, $CDCl_3$) δ –63.1 (s_{br}, CF_3). IR (neat) ν 1687, 1301, 1141, 1109, 1079 cm^{-1}. ESI-MS (*m/z*): 431.1 (100, [M+Na]$^+$), 409.2 (20, [M+H]$^+$), 389.2. Anal. calcd for $C_{24}H_{19}F_3N_2O$ (408.1): C 70.58, H 4.69, N 6.86; found: C 70.47, H 4.79, N 6.89.

trans-5-Benzoyl-1-(4'-isopropylphenyl)-4-phenyl-3-trifluoromethyl-4,5-dihydro-1*H*-pyrazole (**2d**):

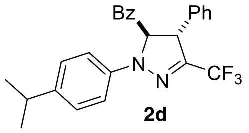

2d CC (SiO_2, petroleum ether/EtOAc 95:5): light yellow solid, 275 mg (63%); mp 96–97 °C. ^{1}H NMR (600 MHz, $CDCl_3$) δ 1.21 (d, $J = 7.0$ Hz, 6H), 2.85 (hept, $J = 7.0$ Hz, 1H), 4.38 (dq, $^{4}J_{H\text{-}F} \approx 1.2$ Hz, $J_{H\text{-}H} = 5.6$ Hz, 1H, 4-H), 5.75 (d, $J_{H\text{-}H} = 5.6$ Hz, 1H, 5-H), 6.94-6.97, 7.11-7.14, 7.19-7.22, 7.39-7.43, 7.48-7.52, 7.65-7.68, 7.87-7.90 (7 m, 2H, 2H, 2H, 3H, 2H, 1H, 2H). ^{13}C NMR (151 MHz, $CDCl_3$) δ 24.2, 33.5, 55.6, 74.4, 113.8, 121.0 (q, $^{1}J_{C\text{-}F} = 270.3$ Hz, CF_3), 127.4, 127.7, 129.0, 129.2, 129.3, 129.7, 133.2, 134.6, 137.4 (q, $^{2}J_{C\text{-}F} = 36.9$ Hz, C-3), 140.6, 142.1, 192.3. ^{19}F NMR (565 MHz, $CDCl_3$) δ –62.3 (s_{br}, CF_3). IR (neat) ν 1687, 1299, 1129 cm^{-1}. ESI-MS (*m/z*): 475.4 (100, [M+K]$^+$), 459.4 (59, [M+Na]$^+$), 437.4 (25, [M+H]$^+$). Anal. calcd for $C_{26}H_{23}F_3N_2O$ (436.2): C 71.55, H 5.31, N 6.42; found: C 71.70, H 5.36, N 6.62.

trans-5-Benzoyl-1-(4'-chlorophenyl)-4-phenyl-3-trifluoromethyl-4,5-dihydro-1*H*-pyrazole (**2e**):

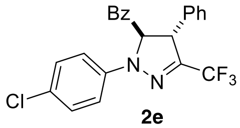

2e CC (SiO_2, petroleum ether/CH_2Cl_2 4:1): light yellow solid, 193 mg (45%); mp 169–170 °C. ^{1}H NMR (600 MHz, $CDCl_3$) δ 4.38 (dq, $^{4}J_{H\text{-}F} \approx 1.2$ Hz, $J_{H\text{-}H} = 5.3$ Hz, 1H, 4-H), 5.75 (d, $J_{H\text{-}H} = 5.3$ Hz, 1H, 5-H), 6.93-6.96, 7.18-7.23, 7.40-7.45, 7.49-7.52, 7.66-7.69, 7.85-7.88 (6 m, 2H, 4H, 3H, 2H, 1H, 2H). ^{13}C NMR (151 MHz, $CDCl_3$) δ 55.7, 74.1, 115.0, 120.7 (q, $^{1}J_{C\text{-}F} = 270.6$ Hz, CF_3), 126.5, 127.6, 129.17, 129.20, 129.4, 129.5, 129.8, 132.9, 134.9, 137.1, 138.8 (q, $^{2}J_{C\text{-}F} = 37.0$ Hz, C-3), 141.3, 191.7. ^{19}F NMR (565 MHz, $CDCl_3$) δ –62.6 (s_{br}, CF_3). IR (neat) ν 1689, 1498, 1305, 1131 cm^{-1}. ESI-MS (*m/z*): 431.3 (43, [M{^{37}Cl}+H]$^+$), 429.3 (100, [M{^{35}Cl}+H]$^+$). Anal. calcd for $C_{23}H_{16}ClF_3N_2O$ (428.1): C 64.42, H 3.76, N 6.53; found: C 64.59, H 3.71, N 6.79.

trans-5-Benzoyl-1-(2',4'-dichlorophenyl)-4-phenyl-3-trifluoromethyl-4,5-dihydro-1*H*-pyrazole (**2f**):

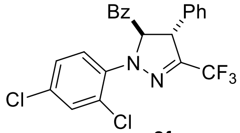

2f CC (SiO_2, petroleum ether/EtOAc 95:5): light brown thick oil, 222 mg (48%). ^{1}H NMR (600 MHz, $CDCl_3$) δ 4.42 (d_{br}, $J \approx 4.1$ Hz, 1H, 4-H), 6.51 (d, $J = 4.1$ Hz, 1H, 5-H), 7.25 (d, $J = 2.4$ Hz, 1H), 7.30 (dd, $J = 2.4$, 8.8 Hz, 1H), 7.34-7.36, 7.42-7.47, 7.61-7.64 (3 m, 2H, 5H, 1H), 7.78 (d, $J = 8.8$ Hz, 1H), 7.79-7.81 (m, 2H). ^{13}C NMR (151 MHz, $CDCl_3$) δ 56.2, 75.2, 120.6 (q, $^{1}J_{C\text{-}F} = 271.1$ Hz, CF_3), 123.8, 126.3, 127.5, 128.0, 129.05, 129.09, 129.3, 129.80, 129.82, 130.2, 132.7, 134.6, 137.2, 140.2, 141.2 (q, $^{2}J_{C\text{-}F} = 37.0$

Hz, C-3), 192.2. ^{19}F NMR (565 MHz, $CDCl_3$) δ –63.2 (s_{br}, CF_3). IR (neat) ν 1694, 1478, 1231, 1116, 1075 cm^{-1}. ESI-MS (*m/z*): 465.3 (55), 464.3 (23), 463.4 (100, $[M+H]^+$). Anal. calcd for $C_{23}H_{15}Cl_2F_3N_2O$ (462.1): C 59.63, H 3.26, N 6.05; found: C 59.57, H 3.49, N 6.32.

4-(*trans*-5'-Benzoyl-4'-phenyl-3'-trifluoromethyl-4',5'-dihydro-1'*H*-pyrazol-1'-yl)phenyl benzoate (**2g**):

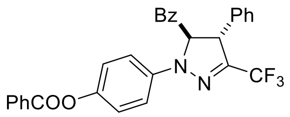

2g

reaction was carried out in hot THF (reflux; heated by immersing a reaction flask into an oil bath at 85 °C) for 4d. CC (SiO_2, petroleum ether/EtOAc 9:1): orange solid, 267 mg (52%); mp 79–80 °C. ^{1}H NMR (600 MHz, $CDCl_3$) δ 4.41 (d_{br}, $J \approx 5.3$ Hz, 1H, 4'-H), 5.79 (d, J = 5.3 Hz, 1H, 5'-H), 7.07, 7.13 (2 d_{br}, $J \approx 9.0$ Hz, 2H each), 7.20-7.23, 7.41-7.53, 7.61-7.69, 7.88-7.91, 8.17-8.20 (5 m, 2H, 7H, 2H, 2H, 2H). ^{13}C NMR (151 MHz, $CDCl_3$) δ 55.8, 74.4, 114.5, 120.8 (q, $^1J_{C\text{-}F}$ = 270.4 Hz, CF_3), 122.6, 127.6, 128.7, 129.1, 129.2, 129.4, 129.7, 129.8, 130.3, 133.0, 133.7, 134.8, 137.2, 138.4 (q, $^2J_{C\text{-}F}$ = 36.9 Hz, C-3'), 140.5, 145.3, 165.5, 191.9. ^{19}F NMR (565 MHz, $CDCl_3$) δ –62.4 (s_{br}, CF_3). IR (neat) ν 1733, 1696, 1510, 1200, 1126, 1062 cm^{-1}. ESI-MS (*m/z*): 537.4 (98, $[M+Na]^+$), 515.4 (100, $[M+H]^+$). Anal. calcd for $C_{30}H_{21}F_3N_2O_3$ (514.2): C 70.03, H 4.11, N 5.44; found: C 70.17, H 4.31, N 5.26.

trans-5-Benzoyl-1-(4'-nitrophenyl)-4-phenyl-3-trifluoromethyl-4,5-dihydro-1*H*-pyrazole (**2h**):

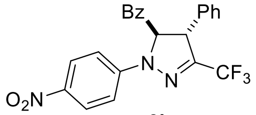

2h

reaction was carried out in hot THF (reflux; heated by immersing a reaction flask into an oil bath at 85 °C) for 5d. CC (SiO_2, hexanes/EtOAc 9:1): red solid, 153 mg (35%); mp 82–83 °C. ^{1}H NMR (600 MHz, $CDCl_3$) δ 4.43 (d_{br}, $J \approx 4.8$ Hz, 1H, 4-H), 5.88 (d_{br}, $J \approx 4.8$ Hz, 1H, 5-H), 7.02-7.05, 7.19-7.23, 7.44-7.56, 7.70-7.74, 7.85-7.89, 8.14-8.19 (6 m, 2H, 2H, 5H, 1H, 2H, 2H). ^{13}C NMR (151 MHz, $CDCl_3$) δ 55.8, 73.5, 113.3, 120.3 (q, $^1J_{C\text{-}F}$ = 271.4 Hz, CF_3), 126.0, 127.5, 129.3, 129.5, 129.6, 130.1, 132.5, 135.3, 136.2, 141.5, 142.4 (q, $^2J_{C\text{-}F}$ = 37.5 Hz, C-3), 147.5, 190.6. ^{19}F NMR (565 MHz, $CDCl_3$) δ –63.0 (s_{br}, CF_3). IR (neat) ν 1692, 1592, 1506, 1297, 1133, 1111 cm^{-1}. ESI-MS (*m/z*): 462.4 (100, $[M+Na]^+$), 440.3 (76, $[M+H]^+$). Anal. calcd for $C_{23}H_{16}F_3N_3O_3$ (439.1): C 62.87, H 3.67, N 9.56; found: C 62.84, H 3.80, N 9.47.

trans-5-Benzoyl-4-(4'-methoxyphenyl)-1-tolyl-3-trifluoromethyl-4,5-dihydro-1*H*-pyrazole (**2i**):

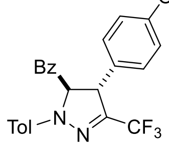

2i

CC (SiO_2, petroleum ether/CH_2Cl_2 3:2): light yellow solid, 342 mg (78%); mp 108–109 °C. ^{1}H NMR (600 MHz, $CDCl_3$) δ 2.28 (s, 3H, Me), 3.84 (s, 3H, OMe), 4.35 (dq, $^4J_{H\text{-}F} \approx 1.3$ Hz, $J_{H\text{-}H}$ = 5.6 Hz, 1H, 4-H), 5.72 (d, J = 5.6 Hz, 1H, 5-H), 6.92-6.95, 7.05-7.08, 7.11-7.15, 7.48-7.51, 7.45-7.48, 7.87-7.90 (6 m, 4H, 2H, 2H, 2H, 1H, 2H). ^{13}C NMR (151 MHz, $CDCl_3$) δ 20.7, 55.0, 55.5, 74.6, 113.8, 115.0, 121.0 (q, $^1J_{C\text{-}F}$ = 270.4 Hz, CF_3), 128.8, 129.2, 129.3, 129.5, 130.0, 130.9, 133.2, 134.6, 137.7 (q, $^2J_{C\text{-}F}$ = 36.6 Hz, C-3), 140.5, 160.0,

192.3. ^{19}F NMR (565 MHz, $CDCl_3$) δ –62.3 (s_{br}, CF_3). IR (neat) ν 1696, 1599, 1513, 1297, 1126 cm^{-1}. ESI-MS (*m/z*): 439.4 (100, $[M+H]^+$). Anal. calcd for $C_{25}H_{21}F_3N_2O_2$ (438.2): C 68.49, H 4.83, N 6.39; found: C 68.33, H 4.86, N 6.49.

trans-5-Benzoyl-4-(2'-naphthyl)-1-tolyl-3-trifluoromethyl-4,5-dihydro-1*H*-pyrazole (**2j**):

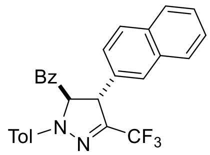

2j CC (SiO_2, petroleum ether/CH_2Cl_2 3:1): yellow solid, 225 mg (49%); mp 74–75 °C. ^{1}H NMR (600 MHz, $CDCl_3$) δ 2.31 (s, 3H, Me), 4.59 (dq, $^4J_{H\text{-}F} \approx 1.4$ Hz, $J_{H\text{-}H} = 5.6$ Hz, 1H, 4-H), 5.89 (d, $J = 5.6$ Hz, 1H, 5-H), 6.98-7.02, 7.10-7.13, 7.32-7.35, 7.46-7.50, 7.55-7.58, 7.66-7.70, 7.82-7.86, 7.89-7.95 (8 m, 2H, 2H, 1H, 2H, 2H, 2H, 1H, 4H). ^{13}C NMR (151 MHz, $CDCl_3$) δ 20.7, 55.7, 74.3, 113.6, 121.0 (q, $^1J_{C\text{-}F} = 270.4$ Hz, CF_3), 124.7, 126.9, 127.00, 127.02, 128.0, 128.1, 129.2, 129.3, 130.03, 130.04, 131.0, 133.1, 133.4, 133.6, 134.6, 134.7, 137.3 (q, $^2J_{C\text{-}F} = 36.9$ Hz, C-3), 140.4, 192.2. ^{19}F NMR (565 MHz, $CDCl_3$) δ –62.2 (s_{br}, CF_3). IR (neat) ν 1692, 1517, 1118 cm^{-1}. ESI-MS (*m/z*): 481.2 (25, $[M+Na]^+$), 459.1 (100, $[M+H]^+$). Anal. calcd for $C_{28}H_{21}F_3N_2O$ (458.2): C 73.35, H 4.62, N 6.11; found: C 73.39, H 4.81, N 6.21.

trans-5-Benzoyl-4-ferrocenyl-1-tolyl-3-trifluoromethyl-4,5-dihydro-1*H*-pyrazole (**2k**):

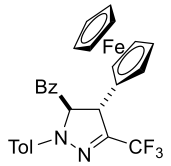

2k CC (SiO_2, petroleum ether/CH_2Cl_2 2:1): thick orange oil, 196 mg (38%). ^{1}H NMR (600 MHz, $CDCl_3$) δ 2.27 (s, 3H, Me), 4.09, 4.20, 4.22, 4.25 (4 s_{br}, 1H, 1H, 5H, 2H, Fc), 4.41 (d_{br}, $J \approx 5.7$ Hz, 1H, 4-H), 5.69 (d, $J = 5.7$ Hz, 1H, 5-H), 7.03, 7.08 (2 d_{br}, $J \approx 8.5$ Hz, 2H each), 7.51-7.55, 7.63-7.67, 8.01-8.04 (3 m, 2H, 1H, 2H). ^{13}C NMR (151 MHz, $CDCl_3$) δ 20.7, 50.7, 65.5, 68.28, 68.30, 68.8, 69.1, 75.8, 86.4, 114.0, 121.1 (q, $^1J_{C\text{-}F} = 270.3$ Hz, CF_3), 128.9, 129.3, 130.1, 131.3, 133.9, 134.4, 137.6 (q, $^2J_{C\text{-}F} = 36.8$ Hz, C-3), 140.8, 195.4. ^{19}F NMR (565 MHz, $CDCl_3$) δ –61.3 (s_{br}, CF_3). IR (neat) ν 1692, 1517, 1122, 1059, 1003 cm^{-1}. ESI-MS (*m/z*): 539.6 (37, $[M+Na]^+$), 517.2 (100, $[M+H]^+$). Anal. calcd for $C_{28}H_{23}F_3FeN_2O$ (516.3): C 65.13, H 4.49, N 5.43; found: C 65.14, H 4.50, N 5.51.

trans-5-Benzoyl-4-(4'-chlorophenyl)-1-tolyl-3-trifluoromethyl-4,5-dihydro-1*H*-pyrazole (**2l**):

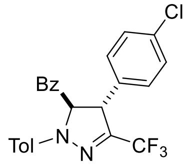

2l CC (SiO_2, petroleum ether/CH_2Cl_2 3:1): light yellow solid, 234 mg (53%); mp 155–156 °C. ^{1}H NMR (600 MHz, $CDCl_3$) δ 2.27 (s, 3H, Me), 4.35 (d_{br}, $J = 5.6$ Hz, 1H, 4-H), 5.70 (d, $J = 5.6$ Hz, 1H, 5-H), 6.90-6.93, 7.05-7.08, 7.13-7.16, 7.37-7.40, 7.49-7.53, 7.65-7.69, 7.85-7.88 (7 m, 2H, 2H, 2H, 2H, 2H, 1H, 2H). ^{13}C NMR (151 MHz, $CDCl_3$) δ 20.7, 54.9, 74.3, 113.9, 120.9 (q, $^1J_{C\text{-}F} = 270.3$ Hz, CF_3), 129.0, 129.1, 129.4,

130.0, 130.1, 131.2, 133.1, 134.8, 135.0, 136.1, 137.0 (q, $^2J_{C\text{-}F}$ = 37.0 Hz, C-3), 140.3, 191.9. ^{19}F NMR (565 MHz, $CDCl_3$) δ –62.3 (s_{br}, CF_3). IR (neat) ν 1692, 1517, 1297, 1121 cm^{-1}. ESI-MS (*m/z*): 465.4 (100, $[M+Na]^+$), 443.2 (19, $[M+H]^+$). Anal. calcd for $C_{24}H_{18}ClF_3N_2O$ (442.1): C 65.09, H 4.10, N 6.33; found: C 65.21, H 4.10, N 6.41.

trans-5-Benzoyl-1-tolyl-3-trifluoromethyl-4-(4'-trifluoromethylphenyl)-4,5-dihydro-1*H*-pyrazole (**2m**):

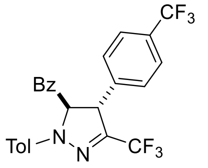

2m CC (SiO_2, petroleum ether/EtOAc 10:1, followed by recrystallization from hexanes): light yellow solid, 457 mg (96%); mp 151–152 °C. ^{1}H NMR (600 MHz, $CDCl_3$) δ 2.27 (s, 3H, Me), 4.43 (dq, $^4J_{H\text{-}F} \approx$ 1.3 Hz, $J_{H\text{-}H}$ = 5.4 Hz, 1H, 4-H), 5.74 (d, J = 5.4 Hz, 1H, 5-H), 6.91-6.95, 7.06-7.09, 7.32-7.35, 7.49-7.53, 7.66-7.70, 7.85-7.87 (6 m, 2H, 2H, 2H, 2H, 3H, 2H). ^{13}C NMR (151 MHz, $CDCl_3$) δ 20.7, 55.0, 74.1, 114.0, 120.9 (q, $^1J_{C\text{-}F}$ = 270.3 Hz, CF_3), 123.9 (q, $^1J_{C\text{-}F}$ = 272.3 Hz, CF_3), 126.8 (q, $^3J_{C\text{-}F}$ = 3.7 Hz, 2 *C*H), 128.1, 129.1, 129.5, 130.1, 131.3 (q, $^2J_{C\text{-}F}$ = 32.7 Hz, *i*-C), 131.4, 133.0, 134.9, 136.6 (q, $^2J_{C\text{-}F}$ = 37.3 Hz, C-3), 140.2, 141.4, 191.7. ^{19}F NMR (565 MHz, $CDCl_3$) δ –62.7, –62.3 (2 s, 2 CF_3). IR (neat) ν 1697, 1517, 1323, 1111 cm^{-1}. ESI-MS (*m/z*): 499.3 (100, $[M+Na]^+$), 477.3 (46, $[M+H]^+$). Anal. calcd for $C_{25}H_{18}F_6N_2O$ (476.1): C 63.03, H 3.81, N 5.88; found: C 62.89, H 3.95, N 6.10.\

trans-5-Benzoyl-4-(4'-nitrophenyl)-1-tolyl-3-trifluoromethyl-4,5-dihydro-1*H*-pyrazole (**2n**):

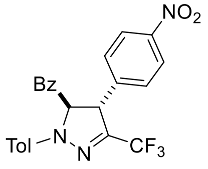

2n CC (SiO_2, petroleum ether/CH_2Cl_2 1:1): yellow solid, 217 mg (48%); mp 222–223 °C (decomp.). ^{1}H NMR (600 MHz, $CDCl_3$) δ 2.28 (s, 3H, Me), 4.48 (dq, $^4J_{H\text{-}F} \approx$ 1.3 Hz, $J_{H\text{-}H}$ = 5.5 Hz, 1H, 4-H), 5.76 (d, J = 5.5 Hz, 1H, 5-H), 6.94, 7.08 (2 d_{br}, $J \approx$ 8.4 Hz, 2H each), 7.40 (d_{br}, $J \approx$ 8.7 Hz, 2H), 7.50-7.54, 7.68-7.72, 7.84-7.87 (3 m, 2H, 1H, 2H), 8.28 (d_{br}, $J \approx$ 8.7 Hz, 2H). ^{13}C NMR (151 MHz, $CDCl_3$) δ 20.7, 54.8, 73.9, 114.0, 120.8 (q, $^1J_{C\text{-}F}$ = 270.2 Hz, CF_3), 125.0, 128.7, 129.0, 129.5, 130.1, 131.7, 132.9, 135.0, 136.0 (q, $^2J_{C\text{-}F}$ = 37.4 Hz, C-3), 140.0, 144.4, 148.3, 191.5. ^{19}F NMR (565 MHz, $CDCl_3$) δ –62.2 (s_{br}, CF_3). IR (neat) ν 1689, 1517, 1345, 1140, 1114 cm^{-1}. ESI-MS (*m/z*): 476.4 (100, $[M+Na]^+$), 454.4 (38, $[M+H]^+$). Anal. calcd for $C_{24}H_{18}F_3N_3O_3$ (453.1): C 63.57, H 4.00, N 9.27; found: C 63.71, H 4.22, N 9.44.

trans-5-(4'-Bromobezoyl)-4-phenyl-1-tolyl-3-trifluoromethyl-4,5-dihydro-1*H*-pyrazole (**2o**):

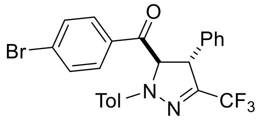

2o CC (SiO_2, petroleum ether/CH_2Cl_2 3:1): yellow solid, 326 mg (67%); mp 155–156 °C. ^{1}H NMR (600 MHz, $CDCl_3$) δ 2.28 (s, 3H, Me), 4.36 (dq, $^4J_{H\text{-}F} \approx$ 1.3 Hz, $J_{H\text{-}H}$ = 5.9 Hz, 1H, 4-H), 5.69 (d, J =

5.9 Hz, 1H, 5-H), 6.92, 7.08 (2 d_{br}, $J \approx 8.5$ Hz, 2H each), 7.19-7.22, 7.40-7.45, 7.63-7.66, 7.71-7.75 (4 m, 2H, 3H, 2H, 2H). ^{13}C NMR (151 MHz, $CDCl_3$) δ 20.7, 55.6, 74.6, 113.8, 120.9 (q, $^1J_{C\text{-}F}$ = 270.3 Hz, CF_3), 127.6, 129.1, 129.8, 130.0, 130.2, 130.6, 131.2, 131.9, 132.7, 137.3, 137.4 (q, $^2J_{C\text{-}F}$ = 37.0 Hz, C-3), 140.3, 191.6. ^{19}F NMR (565 MHz, $CDCl_3$) δ –62.3 (s_{br}, CF_3). IR (neat) ν 1692, 1580, 1517, 1293, 1133, 1062 cm^{-1}. ESI-MS (*m/z*): 489.0 (100, [M{^{81}Br}+H]$^+$), 487.0 (98, [M{^{79}Br}+H]$^+$). Anal. calcd for $C_{24}H_{18}BrF_3N_2O$ (486.1): C 59.15, H 3.72, N 5.75; found: C 59.23, H 3.89, N 5.87.

trans-5-[(3′,4′-Methylenedioxyphenyl)carbonyl]-4-phenyl-1-tolyl-3-trifluoromethyl-4,5-dihydro-1*H*-pyrazole (**2p**):

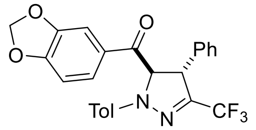

2p

CC (SiO_2, petroleum ether/CH_2Cl_2 3:1): yellow solid, 231 mg (51%); mp 64–65 °C. ^1H NMR (600 MHz, $CDCl_3$) δ 2.27 (s, 3H, Me), 4.34 (dq, $^4J_{H\text{-}F} \approx 1.3$ Hz, $J_{H\text{-}H}$ = 5.8 Hz, 1H, 4-H), 5.64 (d, J = 5.8 Hz, 1H, 5-H), 6.08 (s, 2H, OC*H*$_2$O), 6.82 (d, J = 8.2 Hz, 1H), 6.89-6.92, 7.04-7.07, 7.18-7.21 (3 m, 2H each), 6.82 (dd, J = 1.8, 8.2 Hz, 1H), 7.38-7.42 (m, 4H). ^{13}C NMR (151 MHz, $CDCl_3$) δ 20.7, 55.9, 74.4, 102.4, 108.4, 108.8, 113.8, 121.0 (q, $^1J_{C\text{-}F}$ = 270.2 Hz, CF_3), 125.6, 127.7, 128.0, 128.9, 129.7, 130.0, 130.9, 137.4 (q, $^2J_{C\text{-}F}$ = 36.7 Hz, C-3), 137.7, 149.0, 153.2, 190.5. ^{19}F NMR (565 MHz, $CDCl_3$) δ –62.3 (s_{br}, CF_3). IR (neat) ν 1685, 1603, 1517, 1443, 1252, 1118, 1036 cm^{-1}. ESI-MS (*m/z*): 475.1 (28, [M+Na]$^+$), 453.1 (100, [M+H]$^+$). Anal. calcd for $C_{25}H_{19}F_3N_2O_3$ (452.1): C 66.37, H 4.23, N 6.19; found: C 66.34, H 4.44, N 6.36.

trans-5-Ferrocenoyl-4-phenyl-1-tolyl-3-trifluoromethyl-4,5-dihydro-1*H*-pyrazole (**2q**):

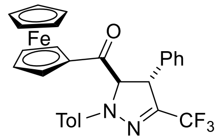

2q

CC (SiO_2, petroleum ether/CH_2Cl_2 2:1): red solid, 181 mg (35%); mp 160–161 °C. ^1H NMR (600 MHz, $CDCl_3$) δ 2.30 (s, 3H, Me), 4.19 (s, 5H, Fc), 4.49 (dq, $^4J_{H\text{-}F} \approx 1.3$ Hz, $J_{H\text{-}H}$ = 5.6 Hz, 1H, 4-H), 4.57, 4.60, 4.64, 4.87 (4 m_c, 4 × 1H, Fc), 5.21 (d, J = 5.6 Hz, 1H, 5-H), 7.09-7.16, 7.19-7.22, 7.36-7.42 (3 m, 4H, 2H, 3H). ^{13}C NMR (151 MHz, $CDCl_3$) δ 20.7, 56.3, 69.7, 69.9, 70.2, 73.0, 73.3, 75.3, 76.7, 114.4, 121.0 (q, $^1J_{C\text{-}F}$ = 270.2 Hz, CF_3), 127.6, 128.8, 129.6, 130.0, 131.3, 137.9 (q, $^2J_{C\text{-}F}$ = 36.8 Hz, C-3), 138.0, 141.0, 198.6. ^{19}F NMR (565 MHz, $CDCl_3$) δ –62.3 (s_{br}, CF_3). IR (neat) ν 1681, 1521, 1118, 1059 cm^{-1}. ESI-MS (*m/z*): 539.3 (100, [M+Na]$^+$), 517.4 (55, [M+H]$^+$). Anal. calcd for $C_{28}H_{23}F_3FeN_2O$ (516.3): C 65.13, H 4.49, N 5.43; found: C 65.05, H 4.53, N 5.50.

Crystals of **2q** for an X-ray structure determination were obtained from hexane/dichloromethane solution by slow evaporation of the solvents.

trans-5-Benzoyl-1-tolyl-3,4-bis(trifluoromethyl)-4,5-dihydro-1*H*-pyrazole (**2r**):

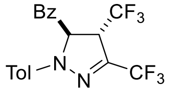

2r CC (SiO_2, petroleum ether/CH_2Cl_2 4:1): yellow crystals, 296 mg (74%); mp 120–121 °C. ^1H NMR (600 MHz, $CDCl_3$) δ 2.27 (s, 3H, Me), 4.07 (dq$_{br}$, $J_{H\text{-}H} \approx 5.7$ Hz, $J_{H\text{-}F} \approx 7.9$ Hz, 1H, 4-H), 6.01 (d$_{br}$, $J \approx 5.7$ Hz, 1H, 5-H), 6.94, 7.07 (2 d$_{br}$, $J \approx 8.4$ Hz, 2H each), 7.57-7.61, 7.71-7.74, 8.04-8.07 (3 m, 2H, 1H, 2H). ^{13}C NMR (151 MHz, $CDCl_3$) δ 20.7, 53.5 (q, $^2J_{C\text{-}F} = 31.8$ Hz, C-4), 65.8, 114.4, 120.4 (q, $^1J_{C\text{-}F} = 269.3$ Hz, 3-CF_3), 123.8 (q, $^1J_{C\text{-}F} = 280.6$ Hz, 4-CF_3), 127.7 (q, $^2J_{C\text{-}F} = 40.1$ Hz, C-3), 129.1, 129.6, 130.1, 132.4, 132.8, 135.2, 139.3, 191.2. ^{19}F NMR (565 MHz, $CDCl_3$) δ –63.7 (q, $J_{F\text{-}F} = 5.0$ Hz, 3-CF_3), –70.0 (dq, $J_{F\text{-}F} = 5.0$ Hz, $J_{H\text{-}F} = 7.9$ Hz, 4-CF_3). IR (neat) ν 1689, 1517, 1223, 1163, 1126 cm^{-1}. HRMS (ESI-TOF) *m/z*: $[M+Na]^+$ calcd for $C_{19}H_{14}F_6N_2NaO$ 423.0908, found 423.0900. Anal. calcd for $C_{19}H_{14}F_6N_2O$ (400.1): C 57.01, H 3.52, N 7.00; found: C 57.14, H 3.56, N 7.09.

trans-1,4,5-Triphenyl-3-trifluoromethyl-4,5-dihydro-1*H*-pyrazole (**7**)[8]:

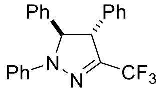

7 CC (SiO_2, petroleum ether/CH_2Cl_2 4:1): colourless solid, 249 mg (68%); mp 102–103 °C. ^1H NMR (600 MHz, $CDCl_3$) δ 4.25 (dq, $J_{H\text{-}F} \approx 1.5$ Hz, $J_{H\text{-}H} = 6.5$ Hz, 1H, 4-H), 5.23 (d, $J = 6.5$ Hz, 1H, 5-H), 6.88-6.91, 7.04-7.07, 7.15-7.23, 7.31-7.39 (4 m, 1H, 2H, 6H, 6H). ^{13}C NMR (151 MHz, $CDCl_3$) δ 61.2, 75.6, 114.4, 121.3 (q, $^1J_{C\text{-}F} = 270.3$ Hz, 3-CF_3), 121.3, 125.5, 127.6, 128.4, 128.5, 129.3, 129.5, 129.7, 138.5 (q, $^2J_{C\text{-}F} = 36.3$ Hz, C-3),138.8, 140.2, 143.1. ^{19}F NMR (565 MHz, $CDCl_3$) δ –62.6 (s$_{br}$, CF_3). IR (neat) ν 1504, 1143, 1118 cm^{-1}. ESI-MS (*m/z*): 367.2 (100, $[M+H]^+$). Anal. calcd for $C_{22}H_{17}F_3N_2$ (366.1): C 72.12, H 4.68, N 7.65; found: C 72.09, H 4.84, N 7.82.

trans-5-Acetyl-1,4-diphenyl-3-trifluoromethyl-4,5-dihydro-1*H*-pyrazole (**9a**):

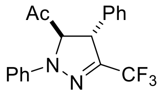

9a CC (SiO_2, petroleum ether/EtOAc 10:1): light yellow solid, 186 mg (56%); mp 134–135 °C. ^1H NMR (600 MHz, $CDCl_3$) δ 2.19 (s, 3H, Me) 4.40 (d$_{br}$, $J \approx 6.0$ Hz, 1H, 4-H), 4.77 (d$_{br}$, $J \approx 6.0$ Hz, 1H, 5-H), 7.00-7.09, 7.16-7.20, 7.32-7.40 (3 m, 3H, 2H, 5H). ^{13}C NMR (151 MHz, $CDCl_3$) δ 25.6, 54.9, 78.4, 113.6, 120.7 (q, $^1J_{C\text{-}F} = 270.6$ Hz, 3-CF_3), 122.0, 127.4, 128.9, 129.6, 129.8, 137.4 138.9 (q, $^2J_{C\text{-}F} = 36.9$ Hz, C-3), 142.6, 204.4. ^{19}F NMR (565 MHz, $CDCl_3$) δ –62.5 (s$_{br}$, CF_3). IR (neat) ν 1595, 1502, 1144, 1111 cm^{-1}. HRMS (ESI-TOF) *m/z*: $[M+H]^+$ calcd for $C_{18}H_{16}F_3N_2O$ 333.1215, found 333.1214.

Methyl (*trans*-1,4-diphenyl-3-trifluoromethyl-4,5-dihydro-1*H*-pyrazol-4-yl)carboxylate (**9b**)[9]:

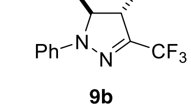

9b CC (SiO_2, petroleum ether/EtOAc 15:1): light yellow solid, 226 mg (65%); mp 137–138 °C. ^1H NMR (600 MHz, $CDCl_3$) δ 3.81 (s, 3H, OMe) 4.61 (d$_{br}$, $J \approx 5.2$ Hz, 1H, 4-H), 4.87 (d, $J = 5.2$

Hz, 1H, 5-H), 7.00-7.04, 7.12-7.15, 7.19-7.22, 7.32-7.41 (4 m, 1H, 2H, 2H, 5H). ^{13}C NMR (151 MHz, $CDCl_3$) δ 53.2, 55.6, 71.5 113.9, 120.8 (q, $^{1}J_{C\text{-}F}$ = 270.6 Hz, 3-CF_3), 121.8, 127.3, 128.8, 129.50, 129.53, 137.4, 139.2 (q, $^{2}J_{C\text{-}F}$ = 36.9 Hz, C-3), 142.7, 169.8. ^{19}F NMR (565 MHz, $CDCl_3$) δ –62.5 (s_{br}, CF_3). IR (neat) v 1730, 1595, 1282, 1230, 1118 cm^{-1}. HRMS (ESI-TOF) *m/z*: $[M+H]^+$ calcd for $C_{18}H_{16}F_3N_2O_2$ 349.1164, found 349.1164.

Synthesis of 3-trifluoromethylpyrazoles of type 5 and 8: A mixture of the respective pyrazoline **2** or **7** (0.2 mmol) in hexane (3 mL) and activated MnO_2 (ca. 85%, <10 μm, 8.0 mmol) was heated by immersing the reaction flask into a preheated oil bath at 60˚C for 2 d. The resulting mixture was cooled to room temperature, diluted with EtOAc (5 mL), filtered and the solvents were removed in vacuo. The crude material was purified by filtration through short silica gel pad by using petroleum ether/CH_2Cl_2 mixture or pure CH_2Cl_2 as eluents to afford spectroscopically pure product.

1,4-Diphenyl-3-trifluoromethyl-1*H*-pyrazole (**5a**)[10]:

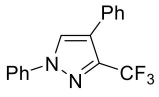

5a CC (SiO_2, petroleum ether/CH_2Cl_2 1:1): colourless solid, 54 mg (94%); mp 40–41 °C. ^{1}H NMR (600 MHz, $CDCl_3$) δ 7.37-7.40, 7.42-7.45, 7.47-7.53, 7.74-7.77 (4 m, 2H, 2H, 4H, 2H), 8.01 (s_{br}, 1H, 5-H). ^{13}C NMR (151 MHz, $CDCl_3$) δ 119.8, 121.7 (q, $^{1}J_{C\text{-}F}$ = 269.9 Hz, CF_3), 124.0 (q_{br}, $^{3}J_{C\text{-}F} \approx$ 1.0 Hz, C-4), 127.6, 127.9, 128.1, 128.7, 128.8 (q_{br}, $J \approx$ 1.2 Hz, 2 *ortho*-*C*H)*, 129.8, 130.4, 139.4, 140.5 (q, $^{2}J_{C\text{-}F}$ = 36.6 Hz, C-3); *through-space coupling. ^{19}F NMR (565 MHz, $CDCl_3$) δ –60.0 (s, CF_3). IR (neat) v 1478, 1232, 1114 cm^{-1}. ESI-MS (*m/z*): 289.1 (100, $[M+H]^+$). Anal. calcd for $C_{16}H_{11}F_3N_2$ (288.1): C 66.66, H 3.85, N 9.72; found: C 66.55, H 3.88, N 9.88.

1-(4'-Benzyloxyphenyl)-4-phenyl-3-trifluoromethyl-1*H*-pyrazole (**5b**):

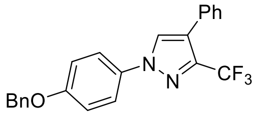

5b CC (SiO_2, petroleum ether/CH_2Cl_2 1:1): light orange solid, 76 mg (97%); mp 80–81 °C. ^{1}H NMR (600 MHz, $CDCl_3$) δ 5.12 (s, 2H, OCH_2), 7.08 (d_{br}, $J \approx$ 9.0 Hz, 2H), 7.35-7.50 (m, 10H), 7.64 (d_{br}, $J \approx$ 9.0 Hz, 2H), 7.91 (s_{br}, 1H, 5-H). ^{13}C NMR (151 MHz, $CDCl_3$) δ 70.5, 115.8, 121.5, 121.8 (q, $^{1}J_{C\text{-}F}$ = 269.8 Hz, CF_3), 123.6 (q_{br}, $^{3}J_{C\text{-}F} \approx$ 0.9 Hz, C-4), 127.6, 127.7, 128.0, 128.3, 128.7, 128.8 (q_{br}, $J \approx$ 1.2 Hz, 2 *ortho*-*C*H), 128.9, 130.5, 133.2, 136.6, 140.0 (q, $^{2}J_{C\text{-}F}$ = 36.8 Hz, C-3), 158.4. ^{19}F NMR (565 MHz, $CDCl_3$) δ –59.4 (s, CF_3). IR (neat) v 1521, 1230, 1174, 1118 cm^{-1}. ESI-MS (*m/z*): 395.2 (100, $[M+H]^+$). Anal. calcd for $C_{23}H_{17}F_3N_2O$ (394.1): C 70.04, H 4.34, N 7.10; found: C 69.98, H 4.53, N 7.15.

4-Phenyl-1-(*p*-tolyl)-3-trifluoromethyl-1*H*-pyrazole (**5c**):

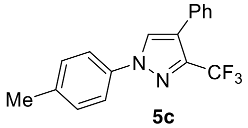

5c CC (SiO_2, CH_2Cl_2): light yellow solid, 59 mg (98%); mp 59–60 °C. ^{1}H NMR (600 MHz, $CDCl_3$) δ 2.41 (s, 3H, Me), 7.30 (d_{br}, $J \approx$ 8.4 Hz, 2H), 7.37-7.40, 7.42-7.45, 7.48-7.50 (3 m, 1H, 2H, 2H), 7.62 (d_{br}, $J \approx$ 8.4 Hz, 2H), 7.97 (s_{br}, 1H, 5-H). ^{13}C NMR (151 MHz, $CDCl_3$) δ 21.1, 119.8, 121.7 (q, $^{1}J_{C\text{-}F}$ = 269.7 Hz,

CF_3), 123.7 (q_{br}, $^3J_{C\text{-}F} \approx 1.0$ Hz, C-4), 127.6, 128.0, 128.7, 128.8 (q_{br}, $J \approx 1.1$ Hz, 2 *ortho*-*C*H), 130.3, 130.5, 137.1, 137.9, 140.1 (q, $^2J_{C\text{-}F} = 36.6$ Hz, C-3). ^{19}F NMR (565 MHz, $CDCl_3$) δ –59.4 (s, CF_3). IR (neat) ν 1478, 1120, 1094 cm^{-1}. ESI-MS (*m/z*): 303.3 (100, [M+H]$^+$). Anal. calcd for $C_{17}H_{13}F_3N_2$ (302.1): C 67.54, H 4.33, N 9.27; found: C 67.50, H 4.59, N 9.09.

1-(4'-Isopropylphenyl)-4-phenyl-3-trifluoromethyl-1*H*-pyrazole (**5d**):

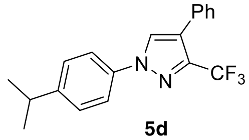

5d

CC (SiO_2, petroleum ether/CH_2Cl_2 3:1): colourless oil, 61 mg (92%). ^{1}H NMR (600 MHz, $CDCl_3$) δ 1.29 (d, $J = 6.9$ Hz, 6H), 2.98 (hept, $J = 6.9$ Hz, 1H), 7.34-7.40, 7.42-7.45, 7.48-7.50, 7.64-7.66 (4 m, 3H, 2H, 2H, 2H), 7.97 (s_{br}, 1H, 5-H). ^{13}C NMR (151 MHz, $CDCl_3$) δ 24.1, 33.9, 119.1, 120.0, 121.7 (q, $^1J_{C\text{-}F} = 270.0$ Hz, CF_3), 123.7 (q_{br}, $^3J_{C\text{-}F} \approx 1.1$ Hz, C-4), 127.6, 127.7, 128.0, 128.7, 128.8 (q_{br}, $J \approx 1.3$ Hz, 2 *ortho*-*C*H), 130.4, 137.3, 140.2 (q, $^2J_{C\text{-}F} = 36.8$ Hz, C-3), 148.9. ^{19}F NMR (565 MHz, $CDCl_3$) δ –59.4 (s, CF_3). IR (neat) ν 1480, 1170, 1114 cm^{-1}. ESI-MS (*m/z*): 331.2 (100, [M+H]$^+$). Anal. calcd for $C_{19}H_{17}F_3N_2$ (330.1): C 69.08, H 5.19, N 8.48; found: C 69.18, H 5.23, N 8.63.

1-(4'-Chlorophenyl)-4-phenyl-3-trifluoromethyl-1*H*-pyrazole (**5e**):

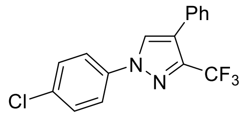

5e

CC (SiO_2, CH_2Cl_2): thick colourless oil, 64 mg (99%). ^{1}H NMR (600 MHz, $CDCl_3$) δ 7.37-7.50, 7.68-7.71 (2 m, 7H, 2H), 7.98 (s_{br}, 1H, 5-H). ^{13}C NMR (151 MHz, $CDCl_3$) δ 120.9, 121.5 (q, $^1J_{C\text{-}F}$ = 270.0 Hz, CF_3), 124.3 (q_{br}, $^3J_{C\text{-}F} \approx 0.9$ Hz, C-4), 127.5, 128.2, 128.8(br)*, 129.9, 130.1, 133.6, 137.9, 140.8 (q, $^2J_{C\text{-}F} = 36.8$ Hz, C-3); *higher intensity. ^{19}F NMR (565 MHz, $CDCl_3$) δ –59.6 (s, CF_3). IR (neat) ν 1476, 1118, 1092 cm^{-1}. ESI-MS (*m/z*): 325.1 (49, [M{^{37}Cl}+H]$^+$), 323.1 (100, [M{^{35}Cl}+H]$^+$). Anal. calcd for $C_{16}H_{10}ClF_3N_2$ (322.1): C 59.55, H 3.12, N 8.68; found: C 59.61, H 3.38, N 8.64.

1-(2',4'-Dichlorophenyl)-4-phenyl-3-trifluoromethyl-1*H*-pyrazole (**5f**):

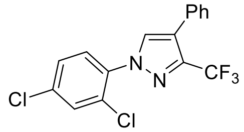

5f

CC (SiO_2, CH_2Cl_2): thick colourless oil, 70 mg (98%). ^{1}H NMR (600 MHz, $CDCl_3$) δ 7.38-7.45, 7.47-7.50 (2 m, 4H, 2H), 7.58 (d, $J = 2.3$ Hz, 1H), 7.63 (d, $J = 8.6$ Hz, 1H), 7.97 (s_{br}, 1H, 5-H). ^{13}C NMR (151 MHz, $CDCl_3$) δ 121.5 (q, $^1J_{C\text{-}F} = 269.9$ Hz, CF_3), 123.3 (q_{br}, $^3J_{C\text{-}F} \approx 0.9$ Hz, C-4), 128.2, 128.4, 128.78, 128.80*, 128.9 (q_{br}, $J \approx 1.1$ Hz, 2 *ortho*-*C*H), 129.2, 130.0, 130.7, 132.1, 135.6, 136.0, 140.8 (q, $^2J_{C\text{-}F} = 36.9$ Hz, C-3); *higher intensity. ^{19}F NMR (565 MHz, $CDCl_3$) δ –59.6 (s, CF_3). IR (neat) ν 1476, 1219, 1170, 1107 cm^{-1}. ESI-MS (*m/z*): 359.1 (63), 357.1 (100, [M+H]$^+$). Anal. calcd for $C_{16}H_9Cl_2F_3N_2$ (356.0): C 53.81, H 2.54, N 7.84; found: C 53.92, H 2.68, N 7.87.

4-(4′-phenyl-3′-trifluoromethyl-1′*H*-pyrazol-1′-yl)phenyl benzoate (**5g**):

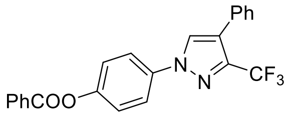

5g CC (SiO_2, petroleum ether/CH_2Cl_2 1:2): colourless solid, 81 mg (99%); mp 114–115 °C. ^{1}H NMR (600 MHz, $CDCl_3$) δ 7.37-7.56, 7.66-7.69, 7.80-7.83 (3 m, 9H, 1H, 2H), 8.01 (s_{br}, 1H, 5-H), 8.21-8.24 (m, 2H). ^{13}C NMR (151 MHz, $CDCl_3$) δ 121.0, 121.6 (q, $^{1}J_{C\text{-}F}$ = 269.8 Hz, CF_3), 123.2, 124.1 (q_{br}, $^{3}J_{C\text{-}F} \approx$ 1.0 Hz, C-4), 127.7, 128.2, 128.78, 128.84(br)*, 129.3, 130.2, 130.4, 134.1, 137.0, 140.7 (q, $^{2}J_{C\text{-}F}$ = 36.9 Hz, C-3), 150.3, 165.1; *higher intensity. ^{19}F NMR (565 MHz, $CDCl_3$) δ –59.5 (s, CF_3). IR (neat) ν 1730, 1495, 1267, 1170, 1115 cm^{-1}. ESI-MS (*m/z*): 431.3 (100, $[M+Na]^+$), 409.3 (69, $[M+H]^+$). Anal. calcd for $C_{23}H_{15}F_3N_2O_2$ (408.1): C 67.65, H 3.70, N 6.86; found: C 67.54, H 3.85, N 6.85.

1-(4′-Nitrophenyl)-4-phenyl-3-trifluoromethyl-1*H*-pyrazole (**5h**):

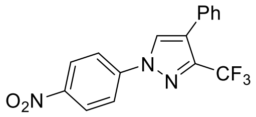

5h CC (SiO_2, petroleum ether/CH_2Cl_2 1:3): light orange solid, 65 mg (98%); mp 117–119 °C. ^{1}H NMR (600 MHz, $CDCl_3$) δ 7.40-7.49 (m, 5H), 7.97 (d_{br}, $J \approx$ 9.2 Hz, 2H), 8.13 (s_{br}, 1H, 5-H), 8.40 (d_{br}, $J \approx$ 9.2 Hz, 2H). ^{13}C NMR (151 MHz, $CDCl_3$) δ 119.5, 121.3 (q, $^{1}J_{C\text{-}F}$ = 270.3 Hz, CF_3), 125.4 (q_{br}, $^{3}J_{C\text{-}F}$ $\approx$ 1.2 Hz, C-4), 125.7, 127.6, 128.6, 128.8 (q_{br}, $J \approx$ 1.1 Hz, 2 *ortho*-*C*H), 128.9, 129.5, 142.3 (q, $^{2}J_{C\text{-}F}$ = 37.2 Hz, C-3), 143.5, 146.6. ^{19}F NMR (565 MHz, $CDCl_3$) δ –60.0 (s, CF_3). IR (neat) ν 1517, 1338, 1230, 1107 cm^{-1}. ESI-MS (*m/z*): 334.3 (100, $[M+H]^+$). HRMS (ESI-TOF) *m/z*: $[M+H]^+$ calcd for $C_{16}H_{11}F_3N_3O_2$ 334.0803, found 334.0804.

4-(4′-Methoxyphenyl)-1-tolyl-3-trifluoromethyl-1*H*-pyrazole (**5i**):

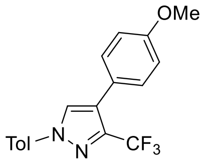

5i CC (SiO_2, CH_2Cl_2): colourless solid, 66 mg (99%); mp 109–110 °C. ^{1}H NMR (600 MHz, $CDCl_3$) δ 2.41 (s, 3H, Me), 3.85 (s, 3H, OMe), 6.96 (d_{br}, $J \approx$ 8.8 Hz, 2H), 7.29 (d_{br}, $J \approx$ 8.3 Hz, 2H), 7.40 (d_{br}, $J \approx$ 8.8 Hz, 2H), 7.61 (d_{br}, $J \approx$ 8.3 Hz, 2H), 7.92 (s_{br}, 1H, 5-H). ^{13}C NMR (151 MHz, $CDCl_3$) δ 21.1, 55.4, 114.2, 119.1, 121.8 (q, $^{1}J_{C\text{-}F}$ = 269.9 Hz, CF_3), 122.8, 123.4 (q_{br}, $^{3}J_{C\text{-}F} \approx$ 1.0 Hz, C-4), 127.3, 130.0 (q_{br}, $J \approx$ 1.1 Hz, 2 *ortho*-*C*H), 130.2, 137.2, 137.8, 140.1 (q, $^{2}J_{C\text{-}F}$ = 36.4 Hz, C-3), 159.5. ^{19}F NMR (565 MHz, $CDCl_3$) δ –59.5 (s, CF_3). IR (neat) ν 1498, 1170, 1103 cm^{-1}. ESI-MS (*m/z*): 355.2 (26, $[M+Na]^+$), 333.3 (100, $[M+H]^+$). Anal. calcd for $C_{18}H_{15}F_3N_2O$ (332.1): C 65.06, H 4.55, N 8.43; found: C 64.89, H 4.70, N 8.52.

4-(Naphth-2'-yl)-1-tolyl-3-trifluoromethyl-1*H*-pyrazole (**5j**):

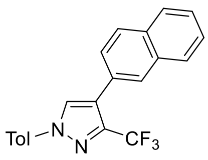

5j CC (SiO_2, CH_2Cl_2): colourless solid, 70 mg (99%); mp 89–90 °C. ^{1}H NMR (600 MHz, $CDCl_3$) δ 2.42 (s, 3H, Me), 7.30-7.33, 7.50-7.54 (2 m, 2H, 2H), 7.59 (dd, J = 1.8, 8.4 Hz, 1H), 7.64-7.67, 7.86-7.91 (2 m, 2H, 3H), 7.95 (m$_c$, 1H), 8.07 (s$_{br}$, 1H, 5-H). ^{13}C NMR (151 MHz, $CDCl_3$) δ 21.2, 119.8, 121.8 (q, $^1J_{C\text{-}F}$ = 269.8 Hz, CF_3), 123.7 (q$_{br}$, $^3J_{C\text{-}F} \approx$ 1.0 Hz, C-4), 126.5, 126.6, 126.8 (q$_{br}$, $J \approx$ 1.0 Hz, *ortho*-*C*H), 127.7 (q$_{br}$, $J \approx$ 1.0 Hz, *ortho*-*C*H), 127.83, 127.84, 127.9, 128.2, 128.4, 130.3, 132.9, 133.4, 137.1, 138.0, 140.3 (q, $^2J_{C\text{-}F}$ = 36.8 Hz, C-3). ^{19}F NMR (565 MHz, $CDCl_3$) δ –59.3 (s, CF_3). IR (neat) ν 1491, 1170, 1115 cm^{-1}. ESI-MS (*m/z*): 353.1 (100, $[M+H]^+$). Anal. calcd for $C_{21}H_{15}F_3N_2$ (352.1): C 71.58, H 4.29, N 7.95; found: C 71.61, H 4.34, N 8.06.

4-Ferrocenyl-1-tolyl-3-trifluoromethyl-1*H*-pyrazole (**5k**):

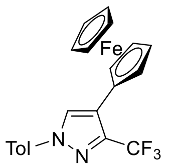

5k CC (SiO_2, petroleum ether/CH_2Cl_2 3:1): light orange solid, 58 mg (71%); mp 103–104 °C. ^{1}H NMR (600 MHz, $CDCl_3$) δ 2.41 (s, 3H, Me), 4.12 (s, 5H, Fc), 4.31, 4.57 (2 m$_c$, 2H each), 7.29, 7.61 (2 d$_{br}$, $J \approx$ 8.4 Hz, 2H each), 7.97 (s$_{br}$, 1H, 5-H). ^{13}C NMR (151 MHz, $CDCl_3$) δ 21.2, 67.9 (q$_{br}$, $J \approx$ 2.1 Hz, 2 *ortho*-*C*H, Fc), 68.7, 69.7, 74.6, 119.7, 120.8 (q$_{br}$, $^3J_{C\text{-}F} \approx$ 1.3 Hz, C-4), 121.9 (q, $^1J_{C\text{-}F}$ = 269.4 Hz, CF_3), 126.6, 130.2, 137.1, 137.7, 139.4 (q, $^2J_{C\text{-}F}$ = 37.0 Hz, C-3). ^{19}F NMR (565 MHz, $CDCl_3$) δ –60.5 (s, CF_3). IR (neat) ν 1491, 1167, 1122, 1059 cm^{-1}. ESI-MS (*m/z*): 433.3 (44, $[M+Na]^+$), 411.3 (51, $[M+H]^+$), 410.3 (100, $[M]^+$). Anal. calcd for $C_{21}H_{17}F_3FeN_2$ (410.1): C 61.49, H 4.18, N 6.83; found: C 61.55, H 4.21, N 7.00.

4-(4'-Chlorophenyl)-1-tolyl-3-trifluoromethyl-1*H*-pyrazole (**5l**):

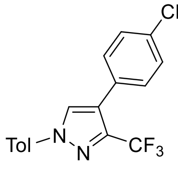

5l CC (SiO_2, CH_2Cl_2): colourless solid, 67 mg (99%); mp 71–72 °C. ^{1}H NMR (600 MHz, $CDCl_3$) δ 2.41 (s, 3H, Me), 7.30 (d$_{br}$, $J \approx$ 8.4 Hz, 2H), 7.40 (s$_{br}$, 4H), 7.61 (d$_{br}$, $J \approx$ 8.4 Hz, 2H), 7.96 (s$_{br}$, 1H, 5-H). ^{13}C NMR ($CDCl_3$, 151 MHz) δ 21.2, 119.8, 121.6 (q, $^1J_{C\text{-}F}$ = 270.1 Hz, CF_3), 122.5(br), 127.6, 128.95, 128.98, 130.1 (q$_{br}$, $J \approx$ 1.2 Hz, 2 *ortho*-*C*H), 130.3, 134.1, 137.0, 138.1, 140.1 (q, $^2J_{C\text{-}F}$ = 36.7 Hz, C-3). ^{19}F NMR (565 MHz, $CDCl_3$) δ –59.5 (s, CF_3). IR (neat) ν 1498, 1223, 1126 cm^{-1}. ESI-MS (*m/z*): 359.3 (24, $[M+Na]^+$), 337.3 (100, $[M+H]^+$). Anal. calcd for $C_{17}H_{12}ClF_3N_2$ (336.1): C 60.64, H 3.59, N 8.32; found: C 60.73, H 3.78, N 8.27.

1-Tolyl-4-(4'-trifluoromethylphenyl)-3-trifluoromethyl-1*H*-pyrazole (**5m**):

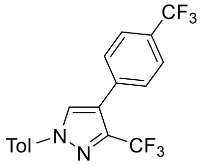

5m CC (SiO_2, CH_2Cl_2): colourless solid, 72 mg (98%); mp 82–83 °C. ^{1}H NMR (600 MHz, $CDCl_3$) δ 2.42 (s, 3H, Me), 7.31 (d_{br}, $J \approx 8.1$ Hz, 2H), 7.58-7.63 (m, 4H), 7.69 (d_{br}, $J \approx 8.1$ Hz, 2H), 8.02 (s_{br}, 1H, 5-H). ^{13}C NMR (151 MHz, $CDCl_3$) δ 21.2, 119.9, 121.6 (q, $^1J_{C\text{-}F}$ = 269.7 Hz, CF_3), 122.3(br), 124.2 (q, $^1J_{C\text{-}F}$ = 272.2 Hz, CF_3), 125.8 (q, $^3J_{C\text{-}F}$ = 7.5 Hz, 2 *C*H), 127.9, 129.1(br), 130.1 (q, $^2J_{C\text{-}F}$ = 32.6 Hz), 130.4, 134.2(br), 136.9, 138.3, 140.2 (q, $^2J_{C\text{-}F}$ = 37.0 Hz, C-3). ^{19}F NMR (565 MHz, $CDCl_3$) δ –62.6, –59.4 (2 s, 2 CF_3). IR (neat) ν 1484, 1323, 1163, 1103, 1066 cm^{-1}. ESI-MS (*m/z*): 371.3 (100, $[M+H]^+$). Anal. calcd for $C_{18}H_{12}F_6N_2$ (370.1): C 58.38, H 3.27, N 7.57; found: C 58.35, H 3.31, N 7.75.

4-(4'-Nitrophenyl)-1-tolyl-3-trifluoromethyl-1*H*-pyrazole (**5n**):

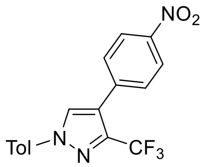

5n CC (SiO_2, CH_2Cl_2): colourless solid, 65 mg (94%); mp 155–156 °C. ^{1}H NMR (600 MHz, $CDCl_3$) δ 2.42 (s, 3H, Me), 7.30-7.33, 7.61-7.66 (2 m, 2H, 4H), 8.08 (q_{br}, $^5J_{H\text{-}F} \approx 1.0$ Hz, 1H, 5-H), 8.27-8.29 (m, 2H). ^{13}C NMR (151 MHz, $CDCl_3$) δ 21.2, 119.9, 121.4 (q, $^1J_{C\text{-}F}$ = 269.8 Hz, CF_3), 121.4 (q_{br}, $^3J_{C\text{-}F} \approx 0.9$ Hz, C-4), 124.1, 128.1, 129.4 (q_{br}, $J_{C\text{-}F} \approx 1.3$ Hz, 2 *ortho*-*C*H), 130.4, 136.7, 137.2, 138.5, 140.1 (q, $^2J_{C\text{-}F}$ = 37.2 Hz, C-3), 147.4. ^{19}F NMR (565 MHz, $CDCl_3$) δ –59.4 (s, CF_3). IR (neat) ν 1517, 1342, 1111 cm^{-1}. ESI-MS (*m/z*): 348.3 (100, $[M+H]^+$). Anal. calcd for $C_{17}H_{12}F_3N_3O_2$ (347.1): C 58.79, H 3.48, N 12.10; found: C 58.84, H 3.61, N 12.11.

1-Tolyl-3,4-trifluoromethyl-1*H*-pyrazole (**5r**):

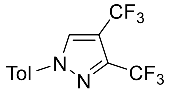

5r CC (SiO_2, CH_2Cl_2): colourless oil, 48 mg (82%). ^{1}H NMR (600 MHz, $CDCl_3$) δ 2.42 (s, 3H, Me), 7.31, 7.56 (2 d_{br}, $J \approx 8.4$ Hz, 2H each), 8.20 (s_{br}, 1H, 5-H). ^{13}C NMR (151 MHz, $CDCl_3$) δ 21.2, 113.3 (q_{br}, $^2J_{C\text{-}F} \approx 39.5$ Hz, C-4), 120.2 (q, $^1J_{C\text{-}F}$ = 269.8 Hz, CF_3), 120.2, 121.2 (q, $^1J_{C\text{-}F}$ = 267.4 Hz, CF_3), 129.1 (q, $^3J_{C\text{-}F} \approx 4.1$ Hz, C-5), 130.5, 136.3, 139.2, 140.2 (qq, $J_{C\text{-}F}$ = 1.8, 39.8 Hz, C-3). ^{19}F NMR (565 MHz, $CDCl_3$) δ –61.6 (q, J = 5.2 Hz, CF_3), –56.9 (q, J = 5.2 Hz, CF_3). IR (neat) ν 1506, 1238, 1141 cm^{-1}. HRMS (ESI-TOF) *m/z*: $[M+H]^+$ calcd for $C_{12}H_9F_6N_2$ 295.0670, found 295.0663.

3-Trifluoromethyl-1,4,5-triphenyl-1*H*-pyrazole (**8**)[8,11,12]:

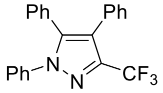

8 CC (SiO_2, petroleum ether/CH_2Cl_2 4:1): colourless solid, 65 mg (90%); mp 162–164 °C. ^{1}H NMR (600 MHz, $CDCl_3$) δ 6.99-7.01, 7.18-7.26, 7.27-7.34 (3 m, 2H, 5H, 8H). ^{13}C NMR (151 MHz, $CDCl_3$) δ

121.2(br), 121.7 (q, $^1J_{C\text{-}F}$ = 270.2 Hz, CF_3), 125.6, 127.7, 128.3, 128.4, 128.6, 128.7, 128.9, 130.46(br), 130.47, 130.6, 139.3, 140.9 (q, $^2J_{C\text{-}F}$ = 36.1 Hz, C-3), 142.5. ^{19}F NMR (565 MHz, $CDCl_3$) δ –59.5 (s, CF_3). IR (neat) ν 1152, 1122 cm^{-1}. ESI-MS (*m/z*): 387.2 (100, $[M+Na]^+$), 365.3 (85, $[M+H]^+$). HRMS (ESI-TOF) *m/z*: $[M+H]^+$ calcd for $C_{22}H_{16}F_3N_2$ 365.1266, found 365.1262.

Synthesis of fully substituted 3-trifluoromethylpyrazoles 6: A mixture of the respective 5-acylpyrazoline **2** (0.2 mmol) in DMSO (2.5 mL) and activated MnO_2 (ca. 85%, <10 μm, 8.0 mmol) was heated by immersing the reaction flask into a preheated oil bath at 100˚C for 2 d under vigorous stirring. The resulting mixture was cooled to room temperature, diluted with EtOAc (5 mL) and filtered. Water (10 mL) was added to the filtrate, the organic layer was separated, and the aqueous layer was extracted with EtOAc (3 × 3 mL). The combined organic extracts were washed with brine (5 mL), dried over anhydrous Na_2SO_4, filtered and the solvents were removed under reduced pressure. The resulting crude product was purified by short column chromatography (SiO_2) using petroleum ether/CH_2Cl_2 or petroleum ether/EtOAc mixtures.

5-Benzoyl-1,4-diphenyl-3-trifluoromethyl-1*H*-pyrazole (**6a**):

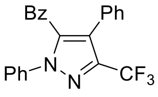

6a

CC (SiO_2, petroleum ether/CH_2Cl_2 1:1): colourless solid, 62 mg (79%); mp 112–114 °C. ^{1}H NMR (600 MHz, $CDCl_3$) δ 7.21-7.27, 7.29-7.32, 7.33-7.38, 7.40-7.43, 7.45-7.47, 7.64-7.66 (6 m, 5H, 2H, 3H, 1H, 2H, 2H). ^{13}C NMR (151 MHz, $CDCl_3$) δ 121.4 (q, $^1J_{C\text{-}F}$ = 270.5 Hz, CF_3), 124.3 (q_{br}, $^3J_{C\text{-}F} \approx$ 1.0 Hz, C-4), 124.5, 128.35*, 128.37, 128.7, 128.9, 129.1, 129.4, 129.8, 130.0, 134.3, 136.1, 139.1, 139.8, 140.6 (q, $^2J_{C\text{-}F}$ = 36.6 Hz, C-3), 187.6; *higher intensity. ^{19}F NMR (565 MHz, $CDCl_3$) δ –59.2 (s, CF_3). IR (neat) ν 1670, 1495, 1159, 1103 cm^{-1}. ESI-MS (*m/z*): 415.3 (83, $[M+Na]^+$), 393.3 (100, $[M+H]^+$). Anal. calcd for $C_{23}H_{15}F_3N_2O$ (392.1): C 70.40, H 3.85, N 7.14; found: C 70.26, H 3.95, N 7.33.

5-Benzoyl-1-(4'-benzyloxyphenyl)-4-phenyl-3-trifluoromethyl-1*H*-pyrazole (**6b**):

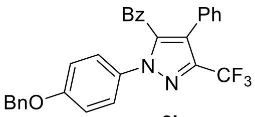

6b

CC (SiO_2, petroleum ether/CH_2Cl_2 1:1): light yellow solid, 61 mg (78%); mp 131–134 °C. ^{1}H NMR (600 MHz, $CDCl_3$) δ 4.90 (s, 2H, OCH_2), 6.80-6.82, 7.07-7.14, 7.16-7.22, 7.24-7.32, 7.51-7.53 (5 m, 2H, 5H, 3H, 7H, 2H). ^{13}C NMR (151 MHz, $CDCl_3$) δ 70.3, 121.4 (q, $^1J_{C\text{-}F}$ = 270.5 Hz, CF_3), 123.9 (q_{br}, $^3J_{C\text{-}F} \approx$ 0.8 Hz, C-4), 126.0, 127.6, 128.27, 128.29(br), 128.33, 128.6, 128.7, 129.0, 129.8, 129.9, 132.3, 134.3, 136.0, 136.4, 139.7, 140.1 (q, $^2J_{C\text{-}F}$ = 36.6 Hz, C-3), 159.1, 187.7. ^{19}F NMR (565 MHz, $CDCl_3$) δ –59.1 (s, CF_3). IR (neat) ν 1672, 1230, 1163, 1126 cm^{-1}. ESI-MS (*m/z*): 521.4 (66, $[M+Na]^+$), 499.4 (100, $[M+H]^+$). Anal. calcd for $C_{30}H_{21}F_3N_2O_2$ (498.2): C 72.28, H 4.25, N 5.62; found: C 72.12, H 4.33, N 5.81.

5-Benzoyl-4-phenyl-1-tolyl-3-trifluoromethyl-1*H*-pyrazole (**6c**):

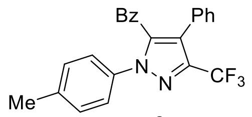

6c CC (SiO_2, petroleum ether/CH_2Cl_2 3:1): colourless solid, 52 mg (64%); mp 134–135 °C. ^{1}H NMR (600 MHz, $CDCl_3$) δ 2.38 (s, 3H, Me), 7.19-7.22, 7.25-7.38, 7.45-7.49, 7.69-7.71 (4 m, 2H, 9H, 1H, 2H). ^{13}C NMR (151 MHz, $CDCl_3$) δ 21.2, 121.4 (q, $^1J_{C\text{-}F}$ = 270.5 Hz, CF_3), 124.1(br), 124.4, 128.32, 128.34, 129.7, 129.0, 129.88, 129.93(br), 130.0, 134.3, 136.0, 136.6, 139.2, 139.7, 140.3 (q, $^2J_{C\text{-}F}$ = 36.8 Hz, C-3), 187.7. ^{19}F NMR (565 MHz, $CDCl_3$) δ –59.1 (s, CF_3). IR (neat) ν 1670, 1103 cm^{-1}. ESI-MS (*m/z*): 429.3 (89, $[M+Na]^+$), 407.4 (100, $[M+H]^+$). Anal. calcd for $C_{24}H_{17}F_3N_2O$ (406.1): C 70.93, H 4.22, N 6.89; found: C 70.83, H 4.46, N 6.93.

5-Benzoyl-1-(4'-isopropylphenyl)-4-phenyl-3-trifluoromethyl-1*H*-pyrazole (**6d**):

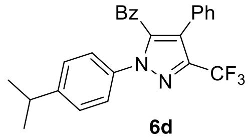

6d CC (SiO_2, petroleum ether/EtOAc 95:5): colourless solid, 75 mg (86%); mp 126–127 °C. ^{1}H NMR (600 MHz, $CDCl_3$) δ 1.20 (d, *J* = 6.9 Hz, 6H), 2.89 (hept, *J* = 6.9 Hz, 1H), 7.19-7.25, 7.27-7.30, 7.34-7.36, 7.39-7.43, 7.64-7.66 (5 m, 7H, 2H, 2H, 1H, 2H). ^{13}C NMR (151 MHz, $CDCl_3$) δ 23.9, 33.9, 121.4 (q, $^1J_{C\text{-}F}$ = 270.3 Hz, CF_3), 124.1(br), 124.5, 127.4, 128.31, 128.34, 128.6, 129.0, 129.88, 129.94(br), 134.3, 136.1, 136.8, 139.7, 140.2 (q, $^2J_{C\text{-}F}$ = 36.6 Hz, C-3), 150.0, 187.7. ^{19}F NMR (565 MHz, $CDCl_3$) δ –59.2 (s, CF_3). IR (neat) ν 1659, 1126 cm^{-1}. ESI-MS (*m/z*): 457.4 (100, $[M+Na]^+$), 435.4 (81, $[M+H]^+$). Anal. calcd for $C_{26}H_{21}F_3N_2O$ (434.2): C 71.88, H 4.87, N 6.45; found: C 71.60, H 4.81, N 6.47.

5-Benzoyl-1-(4'-chlorophenyl)-4-phenyl-3-trifluoromethyl-1*H*-pyrazole (**6e**):

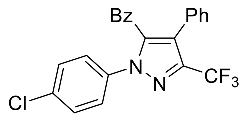

6e CC (SiO_2, petroleum ether/EtOAc 95:5): colourless solid, 67 mg (79%); mp 118–119 °C. ^{1}H NMR (600 MHz, $CDCl_3$) δ 7.20-7.24, 7.25-7.28, 7.34-7.36, 7.39-7.41, 7.42-7.46, 7.63-7.65 (6 m, 3H, 4H, 2H, 2H, 1H, 2H). ^{13}C NMR (151 MHz, $CDCl_3$) δ 121.2 (q, $^1J_{C\text{-}F}$ = 270.5 Hz, CF_3), 124.6 (q_{br}, $^3J_{C\text{-}F} \approx$ 0.9 Hz, C-4), 125.8, 128.4, 128.5, 128.7, 128.8, 129.7, 129.89, 129.90(br), 134.6,135.0, 137.5, 139.7, 140.8 (q, $^2J_{C\text{-}F}$ = 36.9 Hz, C-3), 187.5. ^{19}F NMR (565 MHz, $CDCl_3$) δ –59.3 (s, CF_3). IR (neat) ν 1670, 1491, 1107 cm^{-1}. ESI-MS (*m/z*): 449.3 (100, $[M+Na]^+$), 427.3 (68, $[M+H]^+$). Anal. calcd for $C_{23}H_{14}ClF_3N_2O$ (426.1): C 64.72, H 3.31, N 6.56; found: C 64.49, H 3.47, N 6.40.

5-Benzoyl-1-(2',4'-dichlorophenyl)-4-phenyl-3-trifluoromethyl-1*H*-pyrazole (**6f**):

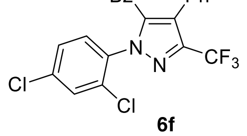

6f CC (SiO_2, petroleum ether/CH_2Cl_2 3:2): colourless solid, 65 mg (71%); mp 86–87 °C. ^{1}H NMR (600 MHz, $CDCl_3$) δ 7.14-7.17, 7.20-7.23, 7.33-7.36 (3 m, 5H, 2H, 1H), 7.42 (dd, *J* = 2.2, 8.5 Hz,

1H), 7.45 (d, J = 2.2 Hz, 1H), 7.58-7.61 (m, 3H). ^{13}C NMR (151 MHz, $CDCl_3$) δ 121.1 (q, $^1J_{C\text{-}F}$ = 270.6 Hz, CF_3), 124.4 (q_{br}, $^3J_{C\text{-}F} \approx$ 0.8 Hz, C-4), 128.2, 128.28, 128.30, 128.44, 128.8, 130.0, 130.2(br)*, 130.4, 131.9, 133.9, 135.2, 135.7, 136.5, 140.9 (q, $^2J_{C\text{-}F}$ = 37.0 Hz, C-3), 141.0, 186.3. ^{19}F NMR (565 MHz, $CDCl_3$) δ –59.3 (s, CF_3). IR (neat) ν 1655, 1484, 1122 cm^{-1}. ESI-MS (m/z): 485.3 (66), 483.3 (100, $[M+Na]^+$), 463.3 (30), 461.3 (67, $[M+H]^+$). Anal. calcd for $C_{23}H_{13}Cl_2F_3N_2O$ (460.0): C 59.89, H 2.84, N 6.07; found: C 59.77, H 3.09, N 6.29.

5-Benzoyl-1-(4'-hydroxyphenyl)-4-phenyl-3-trifluoromethyl-1*H*-pyrazole (**6g**):

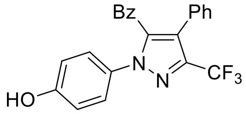

6g CC (SiO_2, petroleum ether/EtOAc 4:1): colourless solid, 50 mg (61%); mp 152–154 °C. ^{1}H NMR (600 MHz, CD_3OD) δ 6.75-6.78, 7.23-7.32, 7.46-7.49, 7.62-7.64 (4 m, 2H, 9H, 1H, 2H). ^{13}C NMR (151 MHz, CD_3OD) δ 116.7, 122.8 (q, $^1J_{C\text{-}F}$ = 269.4 Hz, CF_3), 124.6(br), 127.3, 129.29, 129.34, 129.8, 130.4, 130.8, 131.0(br), 132.2, 135.5, 137.4, 140.8 (q, $^2J_{C\text{-}F}$ = 36.39 Hz, C-3), 141.5, 159.7, 189.1. ^{19}F NMR (565 MHz, CD_3OD) δ –60.5 (s, CF_3). IR (neat) ν 3373, 1640, 1521, 1108 cm^{-1}. ESI-MS (m/z): 431.3 (100, $[M+Na]^+$), 409.4 (65, $[M+H]^+$). HRMS (ESI-TOF) m/z: $[M+H]^+$ calcd for $C_{23}H_{16}F_3N_2O_2$ 409.1164, found 409.1160.

5-Benzoyl-1-(4'-nitrophenyl)-4-phenyl-3-trifluoromethyl-1*H*-pyrazole (**6h**):

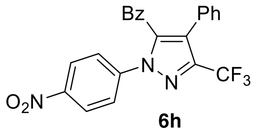

6h CC (SiO_2, petroleum ether/EtOAc 8:1): light yellow solid, 67 mg (77%); mp 110–111 °C. ^{1}H NMR (600 MHz, $CDCl_3$) δ 7.25-7.34, 7.49-7.52, 7.68-7.72, 8.29-8.32 (4 m, 7H, 1H, 4H, 2H). ^{13}C NMR (151 MHz, $CDCl_3$) δ 121.0 (q, $^1J_{C\text{-}F}$ = 270.8 Hz, CF_3), 124.8, 125.0, 125.5 (q_{br}, $^3J_{C\text{-}F} \approx$ 1.0 Hz, C-4), 128.2, 128.5, 128.8, 129.0, 129.9(br), 129.9, 134.9, 135.5, 139.9, 141.9 (q, $^2J_{C\text{-}F}$ = 37.0 Hz, C-3), 143.5, 147.4, 187.2. ^{19}F NMR (565 MHz, $CDCl_3$) δ –59.6 (s, CF_3). IR (neat) ν 1666, 1595, 1521, 1342, 1126 cm^{-1}. ESI-MS (m/z): 460.3 (11, $[M+Na]^+$), 438.3 (100, $[M+H]^+$). Anal. calcd for $C_{23}H_{14}F_3N_3O_3$ (437.1): C 63.16, H 3.23, N 9.61; found: C 63.16, H 3.27, N 9.65.

5-Benzoyl-4-(4'-methoxyphenyl)-1-tolyl-3-trifluoromethyl-1*H*-pyrazole (**6i**):

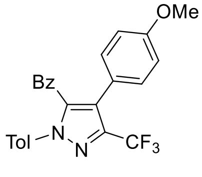

6i CC (SiO_2, petroleum ether/CH_2Cl_2 3:2): light yellow solid, 61 mg (70%); mp 113–114 °C. ^{1}H NMR (600 MHz, $CDCl_3$) δ 2.32 (s, 3H, Me), 3.73 (s, 3H, OMe), 6.76 (d_{br}, $J \approx$ 8.7 Hz, 2H), 7.15 (d_{br}, $J \approx$ 8.3 Hz, 2H), 7.21 (d_{br}, $J \approx$ 8.7 Hz, 2H), 7.25-7.28 (m, 2H), 7.31 (d_{br}, $J \approx$ 8.3 Hz, 2H), 7.42-7.45, 7.74-7.66 (2 m, 1H, 2H). ^{13}C NMR (151 MHz, $CDCl_3$) δ 21.2, 55.3, 113.8, 121.1, 121.4 (q, $^1J_{C\text{-}F}$ = 270.3 Hz, CF_3), 123.8 (q_{br}, $^3J_{C\text{-}F} \approx$ 0.8 Hz, C-4), 124.3, 128.7, 129.0, 130.0, 131.1(br), 134.3, 136.0, 136.7, 139.1, 139.6, 140.3 (q, $^2J_{C\text{-}F}$ = 36.5 Hz, C-3), 159.6, 187.9. ^{19}F NMR (565 MHz, $CDCl_3$) δ –59.2 (s, CF_3). IR (neat) ν 1662, 1226, 1111 cm^{-1}. ESI-MS (m/z): 459.4 (29, $[M+Na]^+$), 437.4 (100, $[M+H]^+$). Anal. calcd for $C_{25}H_{19}F_3N_2O_2$ (436.1): C 68.80, H 4.39, N 6.42; found: C 68.56, H 4.43, N 6.62.

5-Benzoyl-4-(naphth-2'-yl)-1-tolyl-3-trifluoromethyl-1*H*-pyrazole (**6j**):

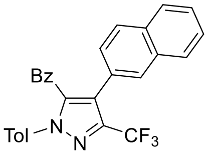

6j CC (SiO_2, petroleum ether/CH_2Cl_2 2:1): colourless solid, 71 mg (78%); mp 128–130 °C. ^{1}H NMR (600 MHz, $CDCl_3$) δ 2.33 (s, 3H, Me), 7.15-7.20, 7.32-7.35 (2 m, 4H, 3H), 7.40 (dd, J = 1.7, 8.5 Hz, 1H), 7.42-7.46, 7.65-7.67 (2 m, 2H, 2H), 7.71 (d, J = 8.5 Hz, 1H), 7.72-7.76 (m, 3H). ^{13}C NMR (151 MHz, $CDCl_3$) δ 21.3, 121.4 (q, $^{1}J_{C-F}$ = 270.5 Hz, CF_3), 123.9 (q$_{br}$, $^{3}J_{C-F} \approx$ 0.8 Hz, C-4), 124.4, 126.4, 126.5, 126.6, 127.4(br), 127.7, 128.1, 128.2, 128.7, 129.5(br), 129.8, 130.1, 132.8, 133.0, 134.3, 136.0, 136.6, 139.3, 139.9, 140.5 (q, $^{2}J_{C-F}$ = 36.6 Hz, C-3), 187.9. ^{19}F NMR (565 MHz, $CDCl_3$) δ –59.1 (s, CF_3). IR (neat) v 1666, 1107 cm^{-1}. ESI-MS (m/z): 495.4 (100 [M+K]$^+$). Anal. calcd for $C_{28}H_{19}F_3N_2O$ (456.2): C 73.68, H 4.20, N 6.14; found: C 73.42, H 4.35, N 6.22.

5-Benzoyl-4-ferrocenyl-1-tolyl-3-trifluoromethyl-1*H*-pyrazole (**6k**):

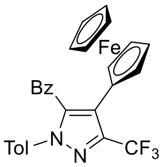

6k CC (SiO_2, petroleum ether/CH_2Cl_2 3:2): red solid, 40 mg (39%); mp 155–156 °C. ^{1}H NMR (600 MHz, $CDCl_3$) δ 2.28 (s, 3H, Me), 4.01 (s, 5H, Fc), 4.17, 4.42 (2 m$_c$, 2H each), 7.08-1.10, 7.25-7.27, 7.37-7.40, 7.51-7.54, 7.73-7.75 (5 m, 2H, 2H, 2H, 1H, 2H). ^{13}C NMR (151 MHz, $CDCl_3$) δ 21.2, 68.8, (q$_{br}$, $J \approx$ 2.6 Hz, 2 *ortho*-CH, Fc), 69.6, 73.5, 120.7(br), 121.7 (q, $^{1}J_{C-F}$ = 269.9 Hz, CF_3), 124.3, 129.1, 129.9, 130.0, 134.8, 136.5, 136.6, 138.7, 139.1, 139.4 (q, $^{2}J_{C-F}$ = 37.0 Hz, C-3), 189.3. ^{19}F NMR (565 MHz, $CDCl_3$) δ –59.6 (s, CF_3). IR (neat) v 1659, 1234, 1159, 1111 cm^{-1}. ESI-MS (m/z): 537.3 (46, [M+Na]$^+$), 515.3 (63, [M+H]$^+$), 514.3 (100, [M]$^+$). HRMS (ESI-TOF) m/z: [M+H]$^+$ calcd for $C_{28}H_{22}F_3FeN_2O$ 515.1034, found 515.1034.

5-Benzoyl-4-(4'-chlorophenyl)-1-tolyl-3-trifluoromethyl-1*H*-pyrazole (**6l**):

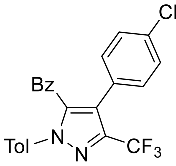

6l CC (SiO_2, petroleum ether/EtOAc 10:1): colourless solid, 77 mg (88%); mp 92–93 °C. ^{1}H NMR (600 MHz, $CDCl_3$) δ 2.32 (s, 3H, Me), 7.14-7.16, 7.20-7.23, 7.27-7.31, 7.45-7.48, 7.62-7.64 (5 m, 2H, 4H, 4H, 1H, 2H). ^{13}C NMR (151 MHz, $CDCl_3$) δ 21.2, 121.3 (q, $^{1}J_{C-F}$ = 270.3 Hz, CF_3), 122.7 (q$_{br}$, $^{3}J_{C-F} \approx$ 0.9 Hz, C-4), 124.3, 127.5, 128.7, 128.8, 129.8, 130.1, 131.2(br), 134.57, 134.58, 135.9, 136.5, 139.4, 139.8, 140.3 (q, $^{2}J_{C-F}$ = 36.8 Hz, C-3), 187.6. ^{19}F NMR (565 MHz, $CDCl_3$) δ –59.2 (s, CF_3). IR (neat) v 1662, 1498, 1122 cm^{-1}. ESI-MS (m/z): 443.3 (56, [M{^{37}Cl}+H]$^+$), 437.4 (100, [M{^{35}Cl}+H]$^+$). Anal. calcd for $C_{23}H_{16}ClF_3N_2O$ (440.1): C 65.39, H 3.66, N 6.35; found: C 65.24, H 3.72, N 6.38.

5-Benzoyl-1-tolyl-4-(4'-trifluoromethylphenyl)-3-trifluoromethyl-1*H*-pyrazole (**6m**):

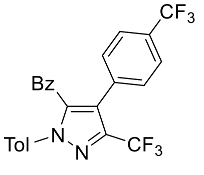

6m CC (SiO_2, petroleum ether/EtOAc 10:1): light yellow solid, 88 mg (93%); mp 120–121 °C. ^{1}H NMR (600 MHz, $CDCl_3$) δ 2.31 (s, 3H, Me), 7.15 (d_{br}, $J \approx 8.3$ Hz, 2H), 7.24-7.27 (m, 2H), 7.32 (d_{br}, $J \approx 8.3$ Hz, 2H), 7.41-7.45, 7.49-7.51, 7.61-7.63 (3 m, 3H, 2H, 2H). ^{13}C NMR (151 MHz, $CDCl_3$) δ 21.2, 121.2 (q, $^1J_{C\text{-}F}$ = 270.4 Hz, CF_3), 122.5(br), 124.0 (q, $^1J_{C\text{-}F}$ = 272.2 Hz, CF_3), 124.3, 125.3 (q, $^3J_{C\text{-}F}$ = 3.7 Hz, 2 *C*H), 128.8, 129.8, 130.1, 130.3, 130.4 (q, $^2J_{C\text{-}F}$ = 32.6 Hz, CF_3), 132.9(br), 134.6, 135.9, 136.4, 139.5, 140.0, 140.4 (q, $^2J_{C\text{-}F}$ = 37.0 Hz, C-3), 187.4. ^{19}F NMR (565 MHz, $CDCl_3$) δ –62.8, –59.1 (2 s, 2 CF_3). IR (neat) ν 1662, 1159, 1111 cm^{-1}. ESI-MS (*m/z*): 475.4 (100, $[M+H]^+$). Anal. calcd for $C_{25}H_{16}F_6N_2O$ (474.1): C 63.29, H 3.40, N 5.91; found: C 63.22, H 3.40, N 6.18.

5-Benzoyl-4-(4'-nitrophenyl)-1-tolyl-3-trifluoromethyl-1*H*-pyrazole (**6n**):

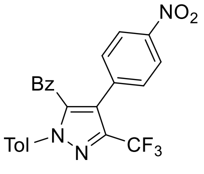

6n CC (SiO_2, petroleum ether/CH_2Cl_2 3:2): light yellow solid, 84 mg (93%); mp 153–154 °C. ^{1}H NMR (600 MHz, $CDCl_3$) δ 2.33 (s, 3H, Me), 7.15-7.17, 7.27-7.33, 7.44-7.50, 7.62-7.64, 8.10-8.13 (5 m, 2H, 4H, 3H, 2H, 2H). ^{13}C NMR (151 MHz, $CDCl_3$) δ 21.2, 121.1 (q, $^1J_{C\text{-}F}$ = 270.4 Hz, CF_3), 121.6(br), 123.6, 124.3, 129.0, 129.8, 130.1, 130.9(br), 134.9, 135.7, 136.0, 136.3, 139.7, 140.1, 140.3 (q, $^2J_{C\text{-}F}$ = 37.3 Hz, C-3), 147.7, 187.1. ^{19}F NMR (565 MHz, $CDCl_3$) δ –59.0 (s, CF_3). IR (neat) ν 1664, 1521, 1312, 1129 cm^{-1}. ESI-MS (*m/z*): 474.4 (53, $[M+Na]^+$), 452.4 (100, $[M+H]^+$). Anal. calcd for $C_{24}H_{16}F_3N_3O_3$ (451.1): C 63.86, H 3.57, N 9.31; found: C 63.94, H 3.58, N 9.25.
Suitable crystals of **6n** for an X-ray structure determination were obtained from hexane/dichloromethane solution by slow evaporation of the solvents.

5-(4’-Bromobenzoyl)-4-phenyl-1-tolyl-3-trifluoromethyl-1*H*-pyrazole (**6o**):

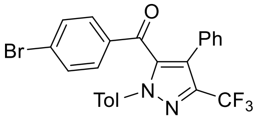

6o CC (SiO_2, petroleum ether/CH_2Cl_2 3:2): colourless solid, 80 mg (83%); mp 147–148 °C. ^{1}H NMR (600 MHz, $CDCl_3$) δ 2.37 (s, 3H, Me), 7.18-7.21, 7.25-7.30, 7.31-7.33 (3 m, 2H, 5H, 2H), 7.42, 7.53 (2 d_{br}, $J \approx 8.6$ Hz, 2H each). ^{13}C NMR (151 MHz, $CDCl_3$) δ 21.3, 121.3 (q, $^1J_{C\text{-}F}$ = 270.6 Hz, CF_3), 124.2 (q, $^3J_{C\text{-}F}$ = 0.8 Hz, C-4), 124.3, 128.48, 128.53, 128.8, 129.8, 129.9(br), 130.1, 131.2, 132.1, 134.8, 136.5, 139.2, 139.4, 140.4 (q, $^2J_{C\text{-}F}$ = 36.8 Hz, C-3), 186.6. ^{19}F NMR (565 MHz, $CDCl_3$) δ –59.2 (s, CF_3). IR (neat) ν 1685, 1252, 1118, 1036 cm^{-1}. ESI-MS (*m/z*): 509.2 (100, $[M\{^{81}Br\}+Na]^+$), 507.3 (80, $[M\{^{79}Br\}+Na]^+$), 487.3 (74,

[M{^{81}Br}+H]$^+$), 485.3 (67, [M{^{79}Br}+H]$^+$). Anal. calcd for $C_{24}H_{16}BrF_3N_2O$ (485.3): C 59.40, H 3.32, N 5.77; found: C 59.15, H 3.52, N 5.97.

5-[(3',4'-Methylenedioxyphenyl)carbonyl]-4-phenyl-1-tolyl-3-trifluoromethyl-1*H*-pyrazole (**6p**):

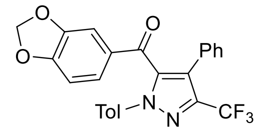

6p

CC (SiO_2, petroleum ether/EtOAc 10:1): colourless solid, 48 mg (53%); mp 158–159 °C. ^{1}H NMR (600 MHz, $CDCl_3$) δ 2.34 (s, 3H, Me), 5.97 (s, 2H, OCH_2O), 6.62 (d, J = 8.1 Hz, 1H), 7.15-7.21, 7.24-7.33 (2 m, 4H, 7H). ^{13}C NMR (151 MHz, $CDCl_3$) δ 21.3, 102.3, 108.1, 108.5, 121.4 (q, $^1J_{C\text{-}F}$ = 270.4 Hz, CF_3), 123.4 (q_{br}, $^3J_{C\text{-}F} \approx$ 0.9 Hz, C-4), 124.2, 127.9, 128.3, 128.4, 129.2, 129.8(br), 130.0, 131.0, 136.6, 139.1, 139.8, 140.2 (q, $^2J_{C\text{-}F}$ = 36.7 Hz, C-3), 148.5, 153.1, 185.8. ^{19}F NMR (565 MHz, $CDCl_3$) δ –59.1 (s_{br}, CF_3). IR (neat) ν 1667, 1491, 1264, 1115 cm^{-1}. ESI-MS (m/z): 473.3 (100, [M+Na]$^+$), 451.3 (85, [M+H]$^+$). Anal. calcd for $C_{25}H_{17}F_3N_2O_3$ (450.1): C 66.67, H 3.80, N 6.22; found: C 66.61, H 3.82, N 6.40.

5-Benzoyl-1-tolyl-3,4-bis(trifluoromethyl)-1*H*-pyrazole (**6r**):

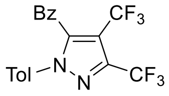

6r

CC (SiO_2, petroleum ether/EtOAc 95:5): colourless oil, 56 mg (71%). ^{1}H NMR (600 MHz, $CDCl_3$) δ 2.31 (s, 3H, Me), 7.14, 7.25 (2 d_{br}, $J \approx$ 8.2 Hz, 2H each), 7.46-7.49, 7.62-7.65, 7.74-7.76 (3 m, 2H, 1H, 2H). ^{13}C NMR (151 MHz, $CDCl_3$) δ 21.3, 111.9 (q, $^2J_{C\text{-}F}$ = 40.2 Hz, C-4), 120.0 (q, $^1J_{C\text{-}F}$ = 270.4 Hz, CF_3), 120.8 (q, $^1J_{C\text{-}F}$ = 268.7 Hz, CF_3), 124.4, 129.3, 129.8, 130.3, 135.4, 135.5, 135.6(br), 140.0 (qq, $J_{C\text{-}F}$ = 2.2, 40.1 Hz, C-3), 140.3, 141.5 (q, $^3J_{C\text{-}F}$ = 2.7 Hz, C-5), 185.8. ^{19}F NMR (565 MHz, $CDCl_3$) δ –61.4, –54.6, (2 q, $J_{F\text{-}F}$ = 5.9 Hz, 2 CF_3). IR (neat) ν 1677, 1506, 1208, 1148 cm^{-1}. HRMS (ESI-TOF) m/z: [M+H]$^+$ calcd for $C_{19}H_{13}F_6N_2O$ 399.0932, found 399.0933.

3. Copies of ^{1}H and ^{13}C NMR spectra

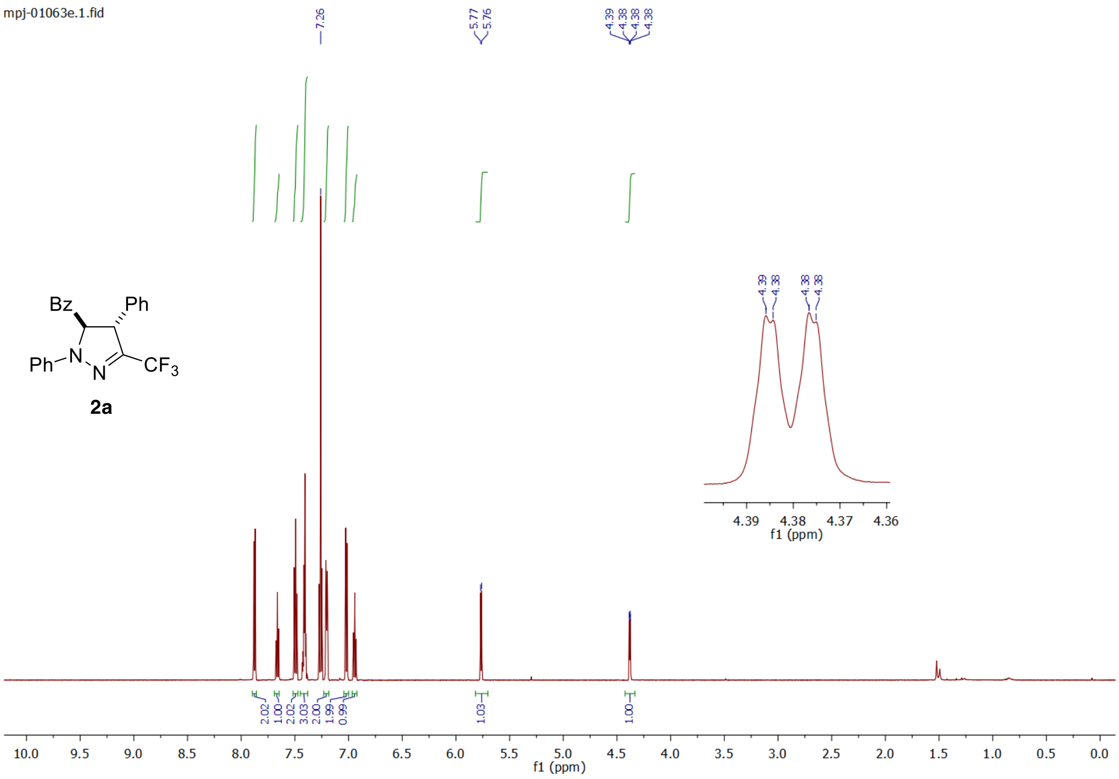

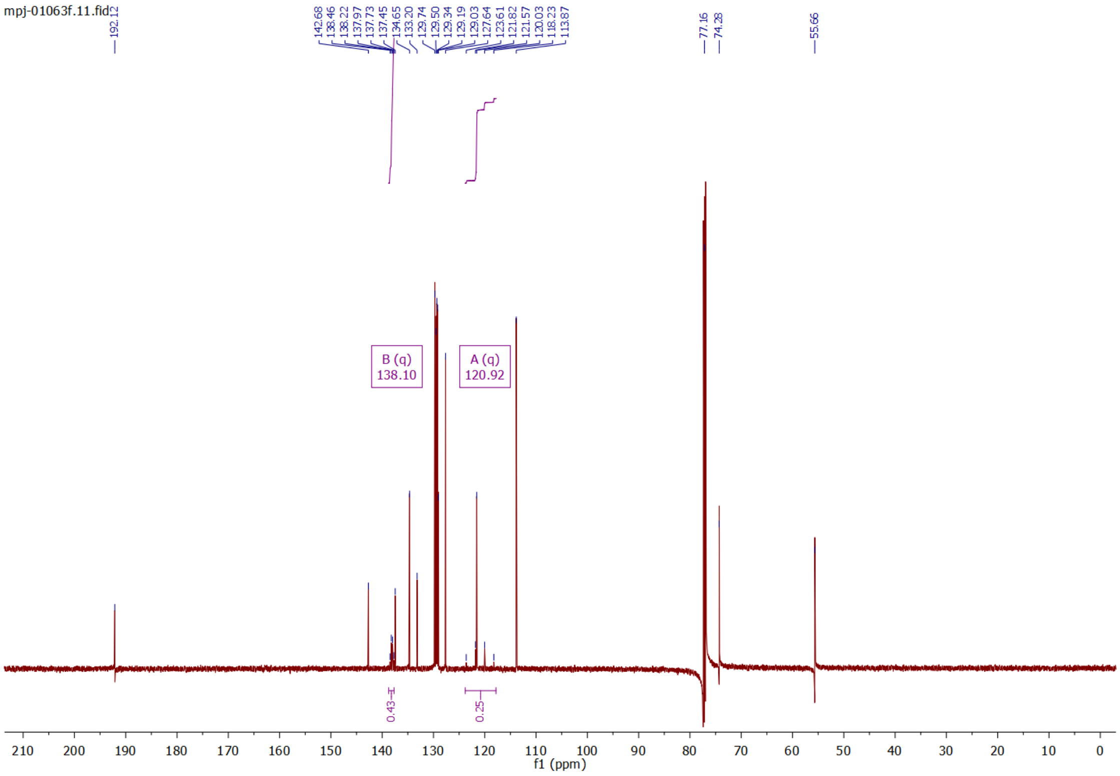

Fig S2. ^{1}H NMR (600 MHz, $CDCl_3$) and ^{13}C NMR (151 MHz, $CDCl_3$) spectra for compound **2a**.

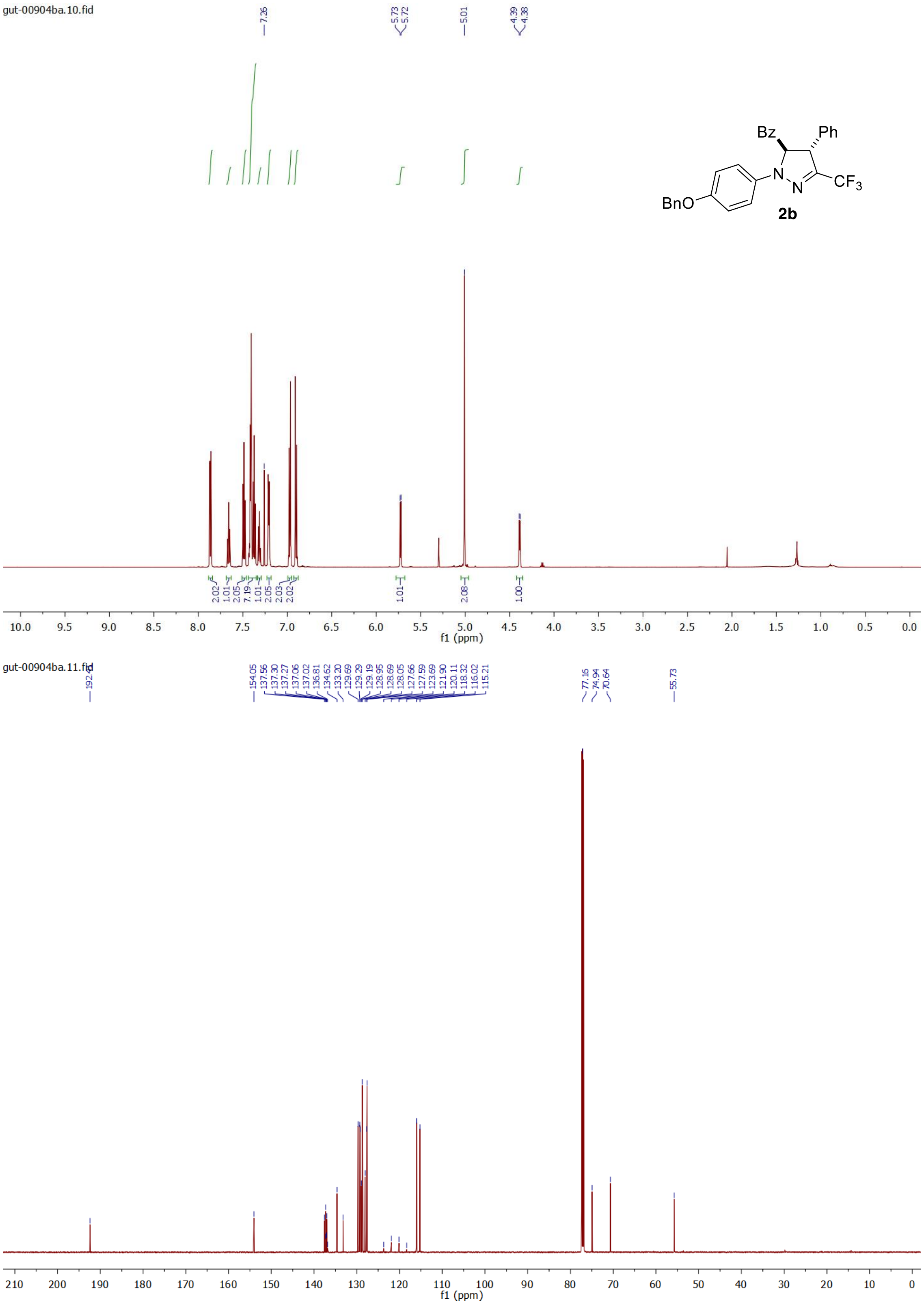

Fig S3. ^{1}H NMR (600 MHz, $CDCl_3$) and ^{13}C NMR (151 MHz, $CDCl_3$) spectra for compound **2b**.

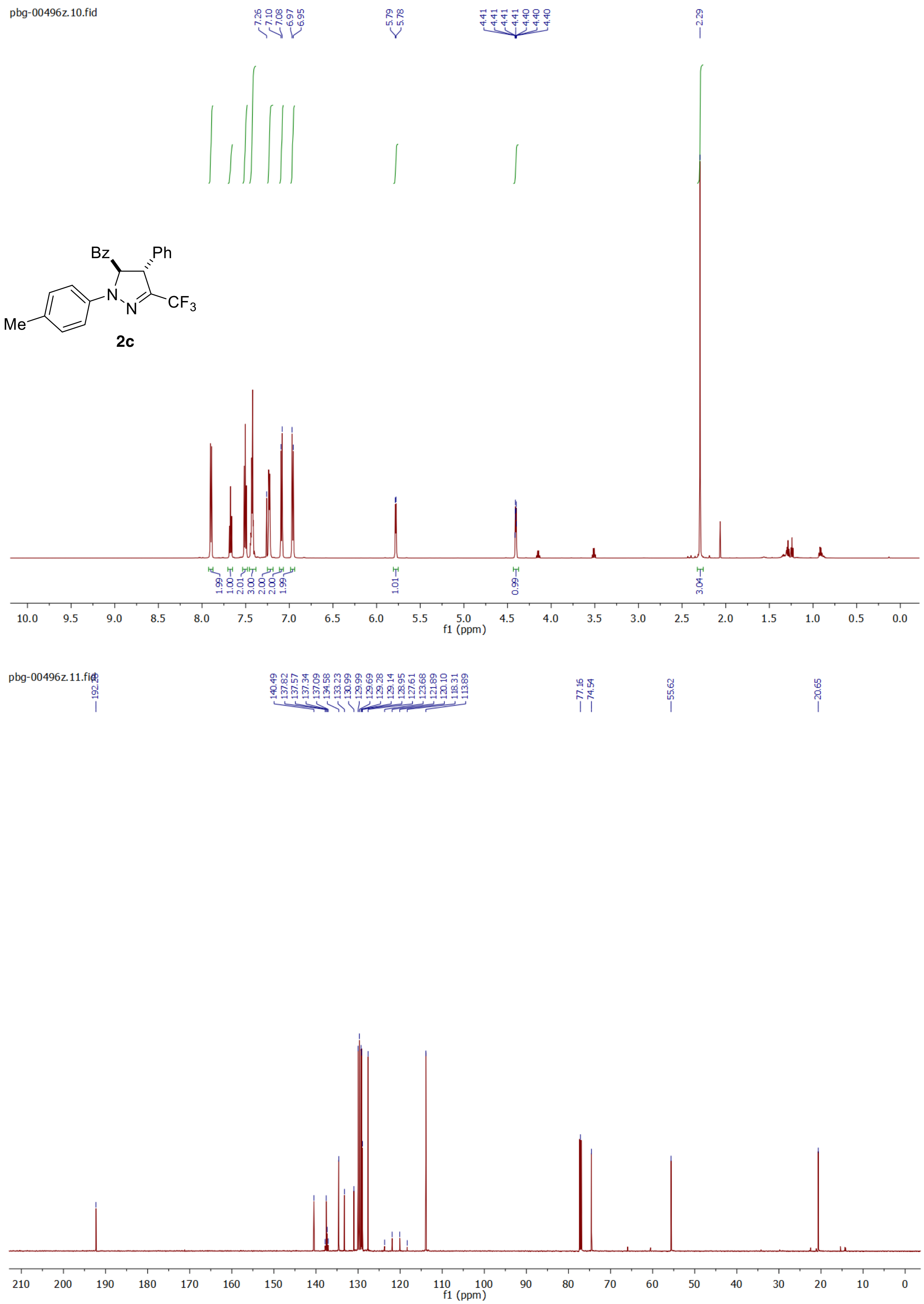

Fig S4. ^{1}H NMR (600 MHz, $CDCl_3$) and ^{13}C NMR (151 MHz, $CDCl_3$) spectra for compound **2c**.

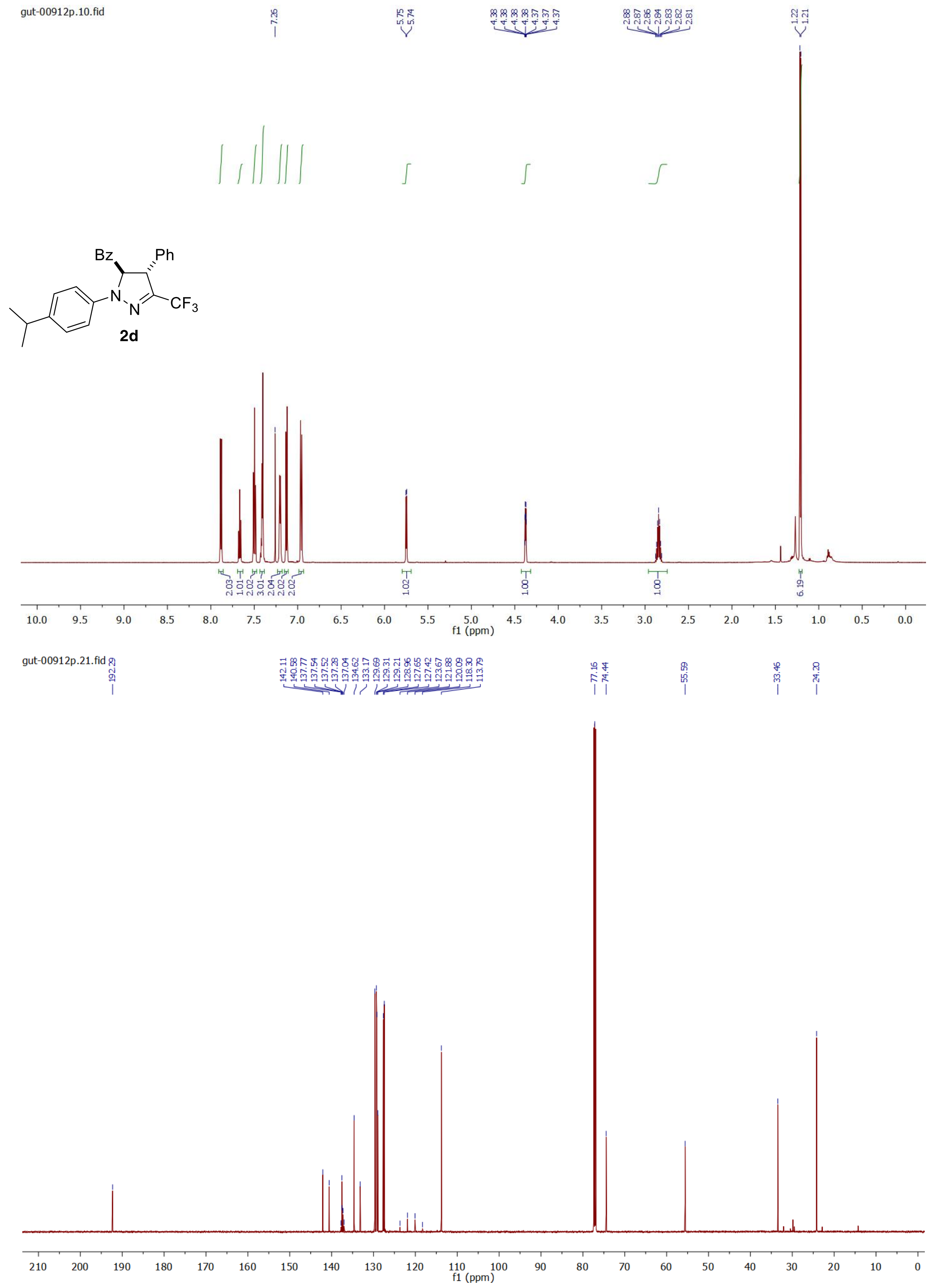

Fig S5. ^{1}H NMR (600 MHz, CDCl$_3$) and ^{13}C NMR (151 MHz, CDCl$_3$) spectra for compound **2d**.

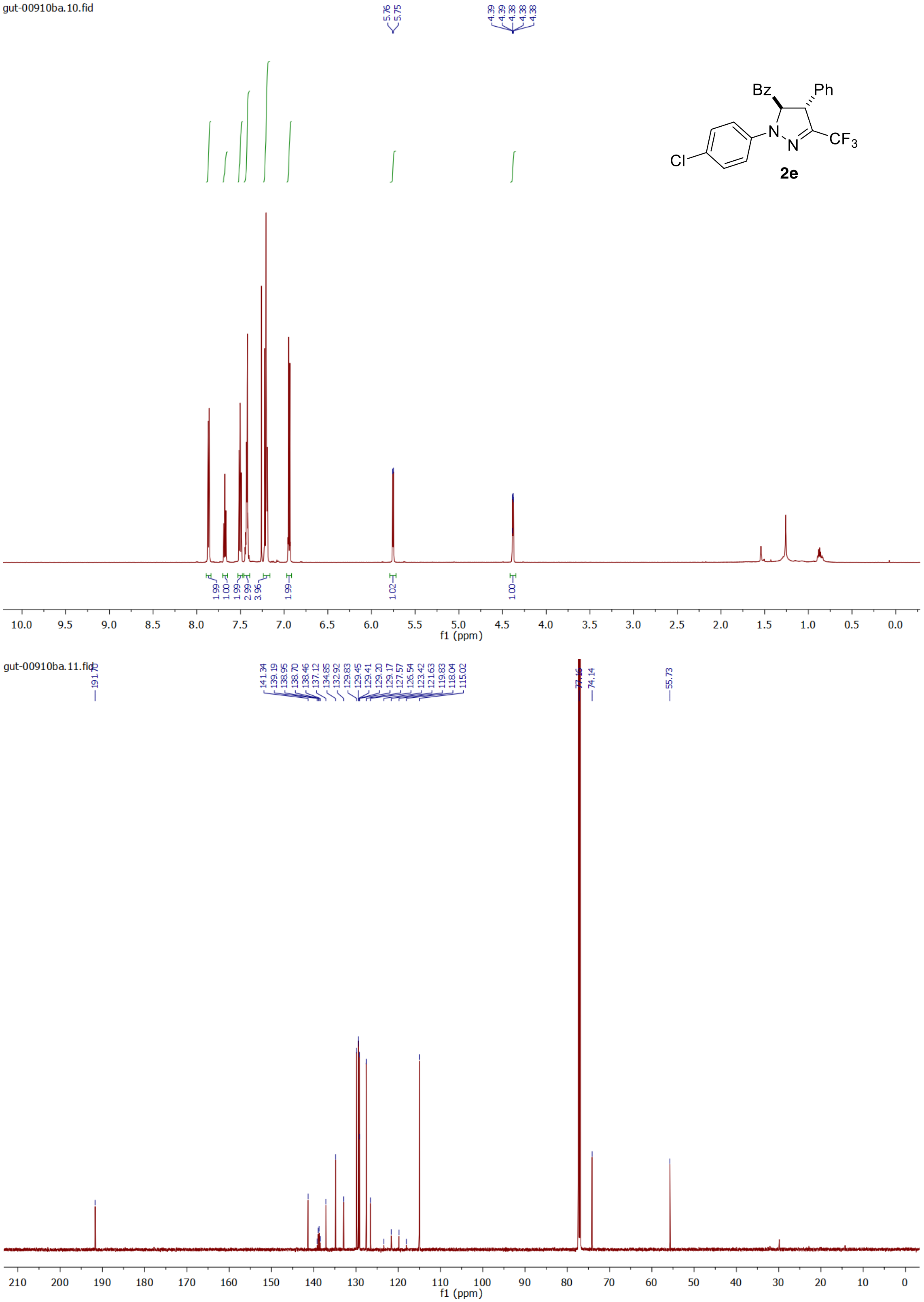

Fig S6. ^{1}H NMR (600 MHz, $CDCl_3$) and ^{13}C NMR (151 MHz, $CDCl_3$) spectra for compound **2e**.

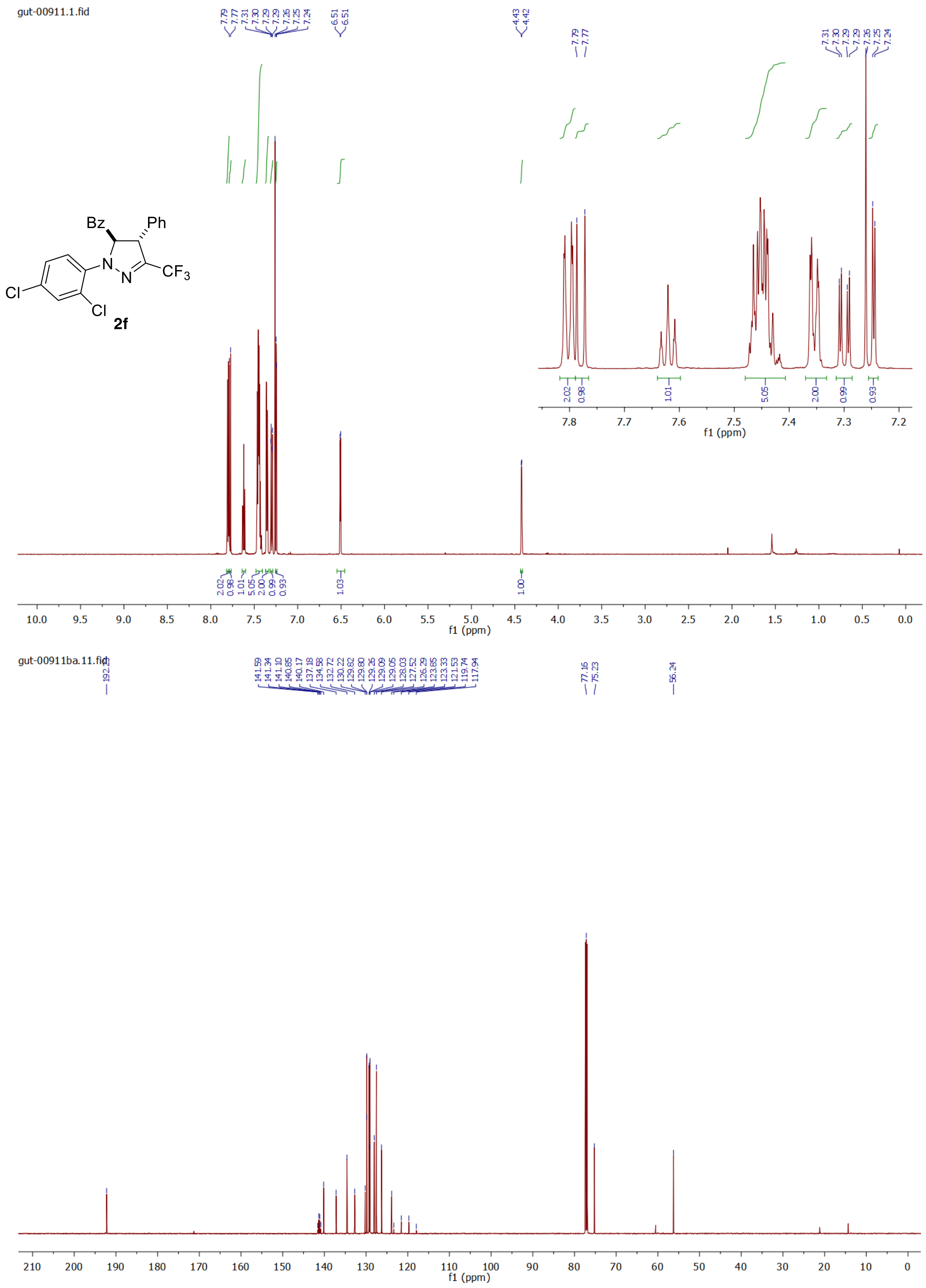

Fig S7. ^{1}H NMR (600 MHz, $CDCl_3$) and ^{13}C NMR (151 MHz, $CDCl_3$) spectra for compound **2f**.

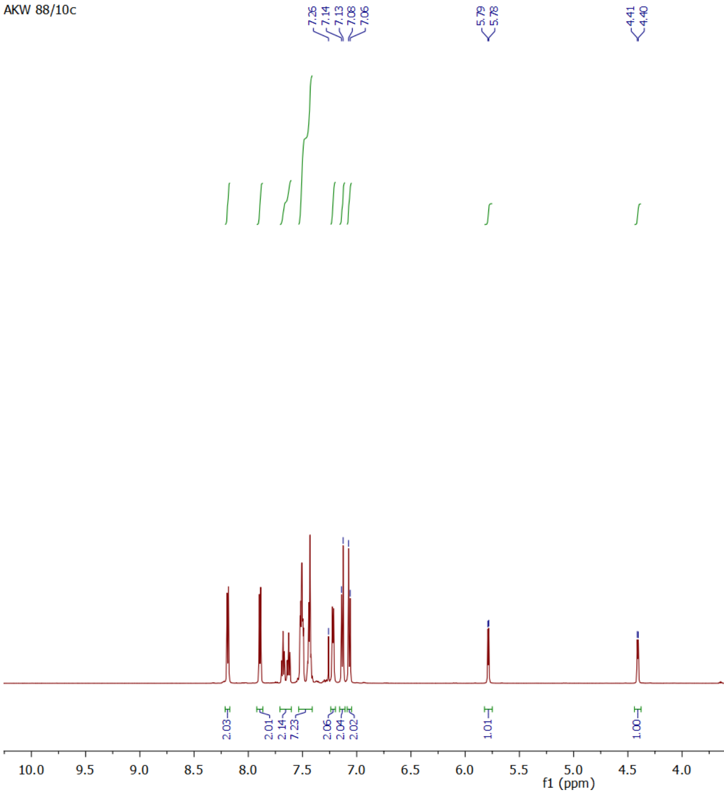

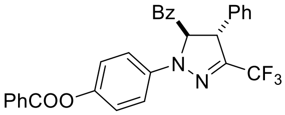

2g

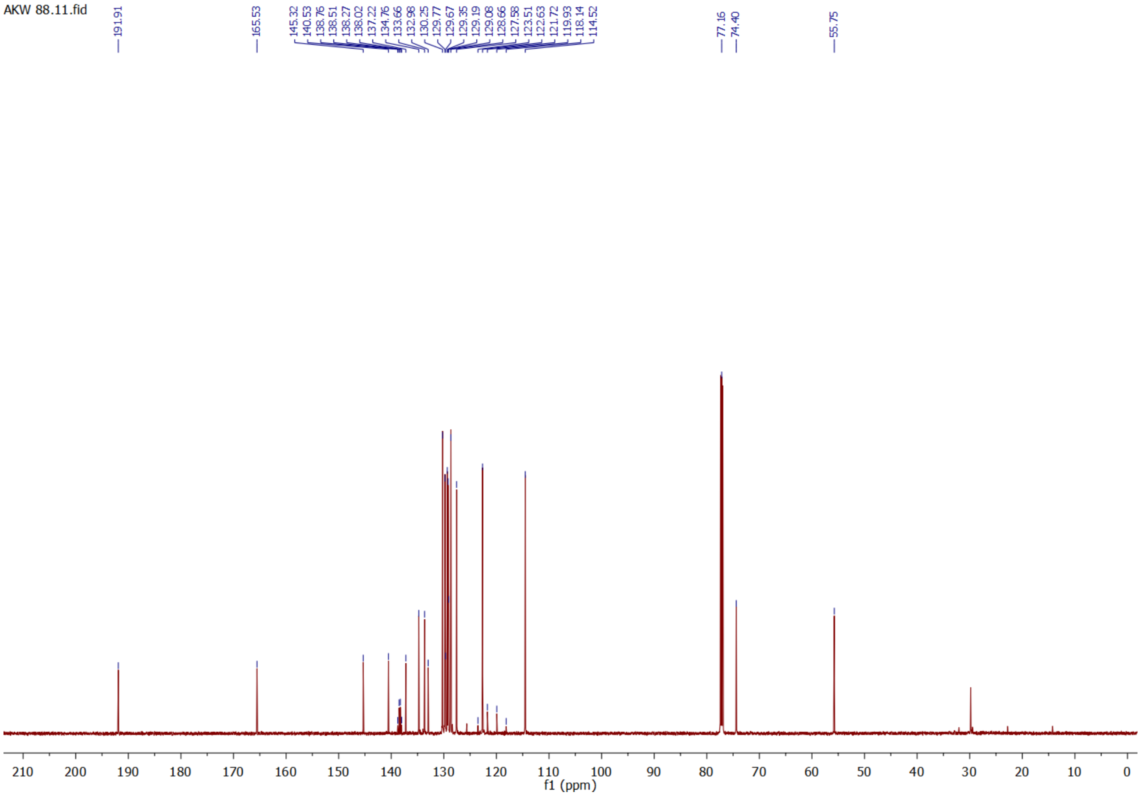

Fig S8. ^{1}H NMR (600 MHz, $CDCl_3$) and ^{13}C NMR (151 MHz, $CDCl_3$) spectra for compound **2g**.

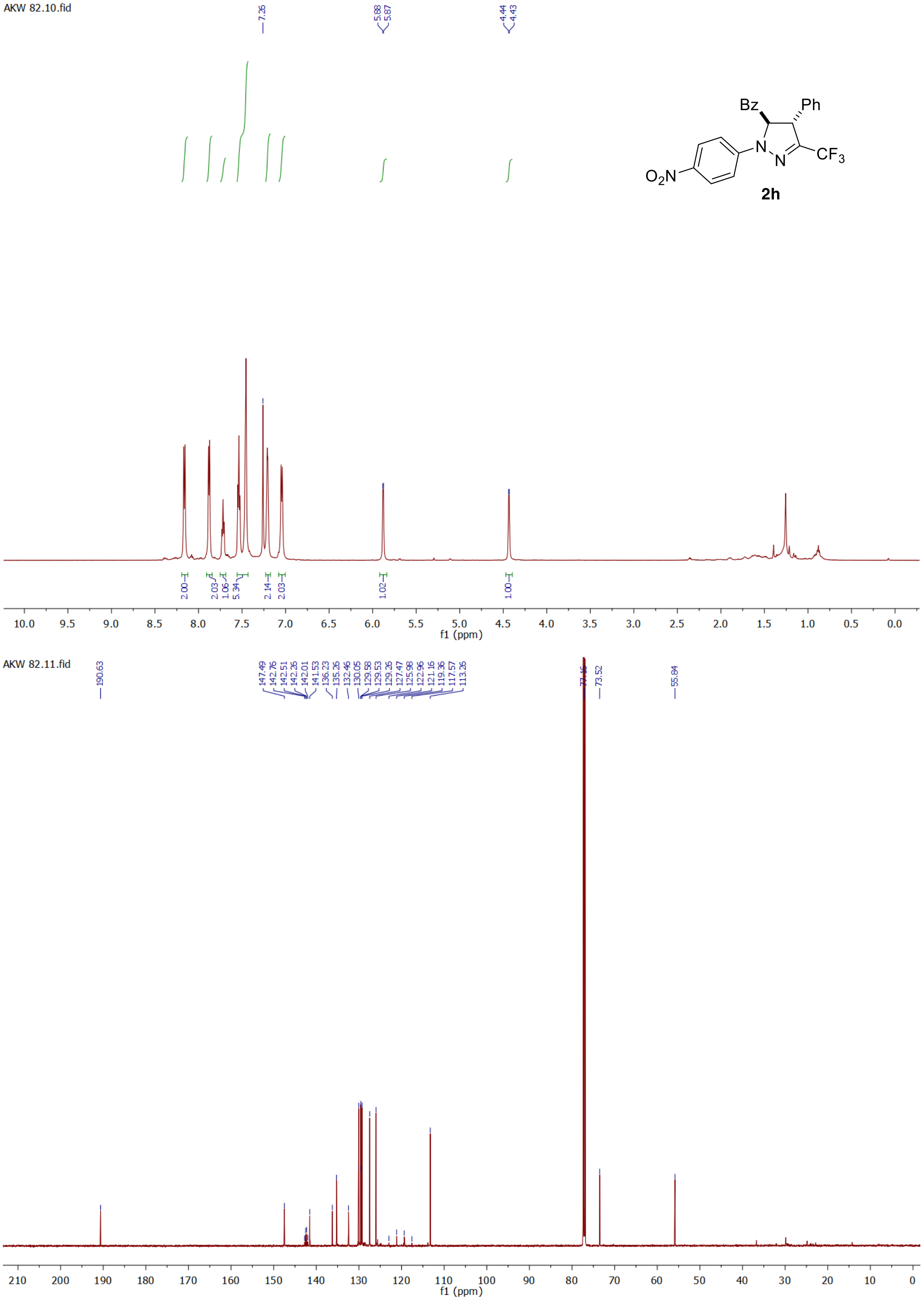

Fig S9. ^{1}H NMR (600 MHz, $CDCl_3$) and ^{13}C NMR (151 MHz, $CDCl_3$) spectra for compound **2h**.

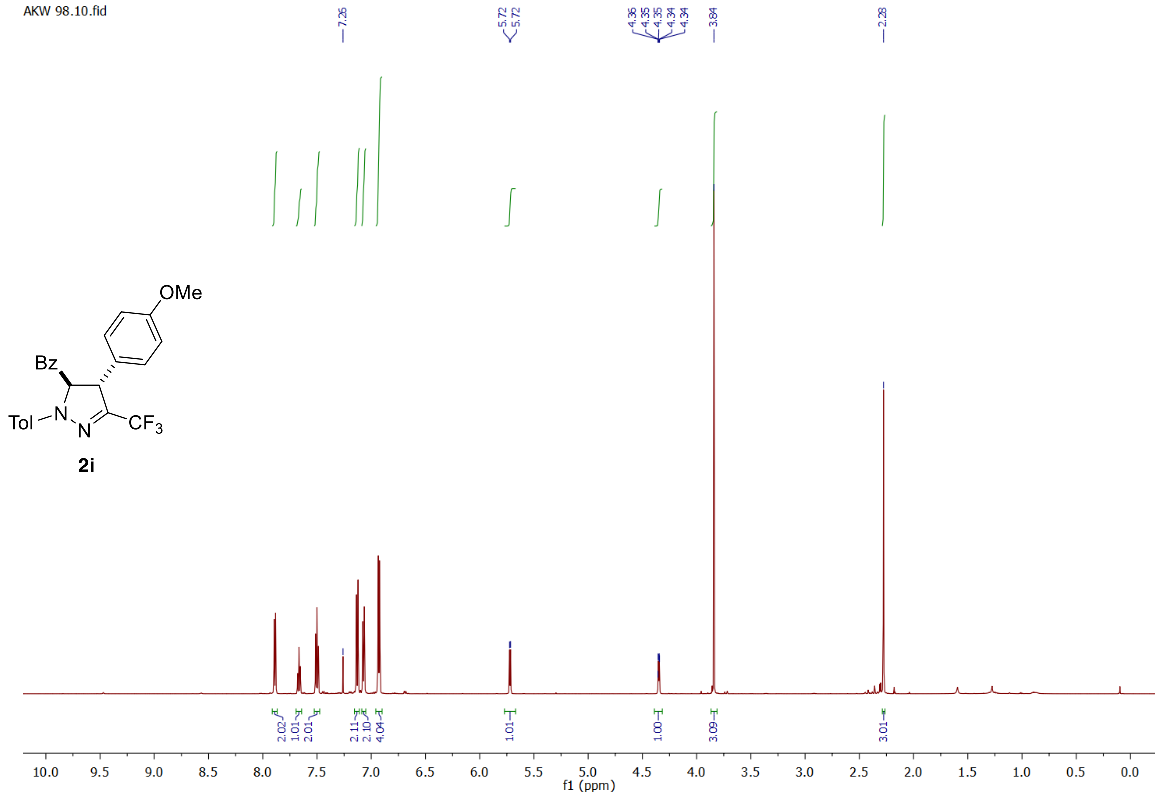

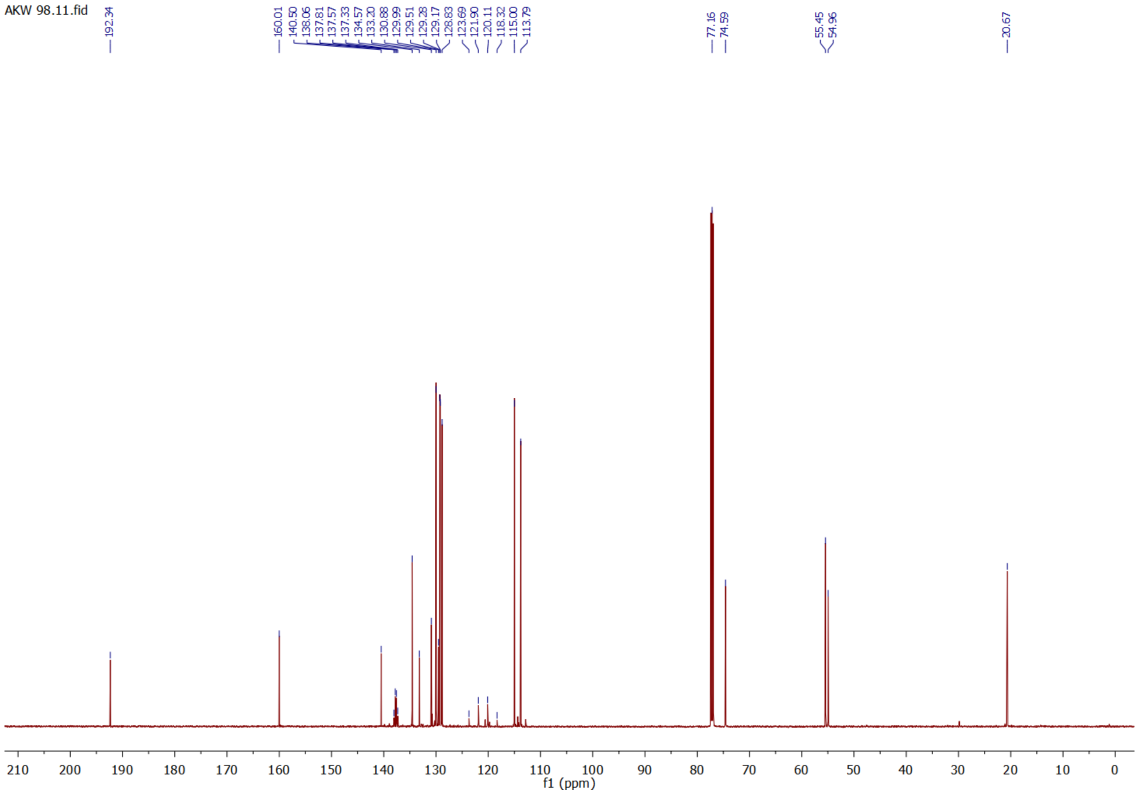

Fig S10. ^{1}H NMR (600 MHz, $CDCl_3$) and ^{13}C NMR (151 MHz, $CDCl_3$) spectra for compound **2i**.

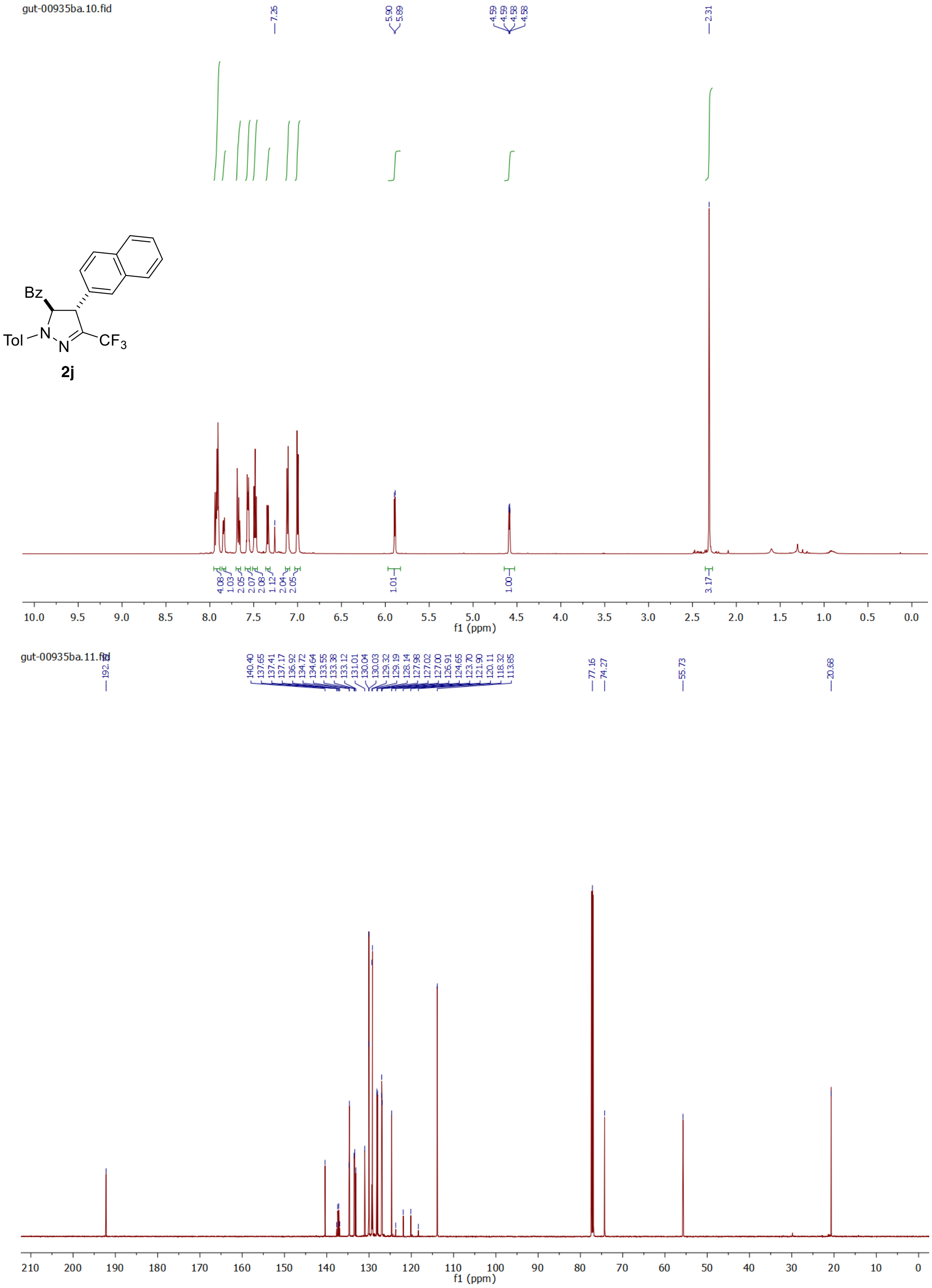

Fig S11. ^{1}H NMR (600 MHz, $CDCl_3$) and ^{13}C NMR (151 MHz, $CDCl_3$) spectra for compound **2j**.

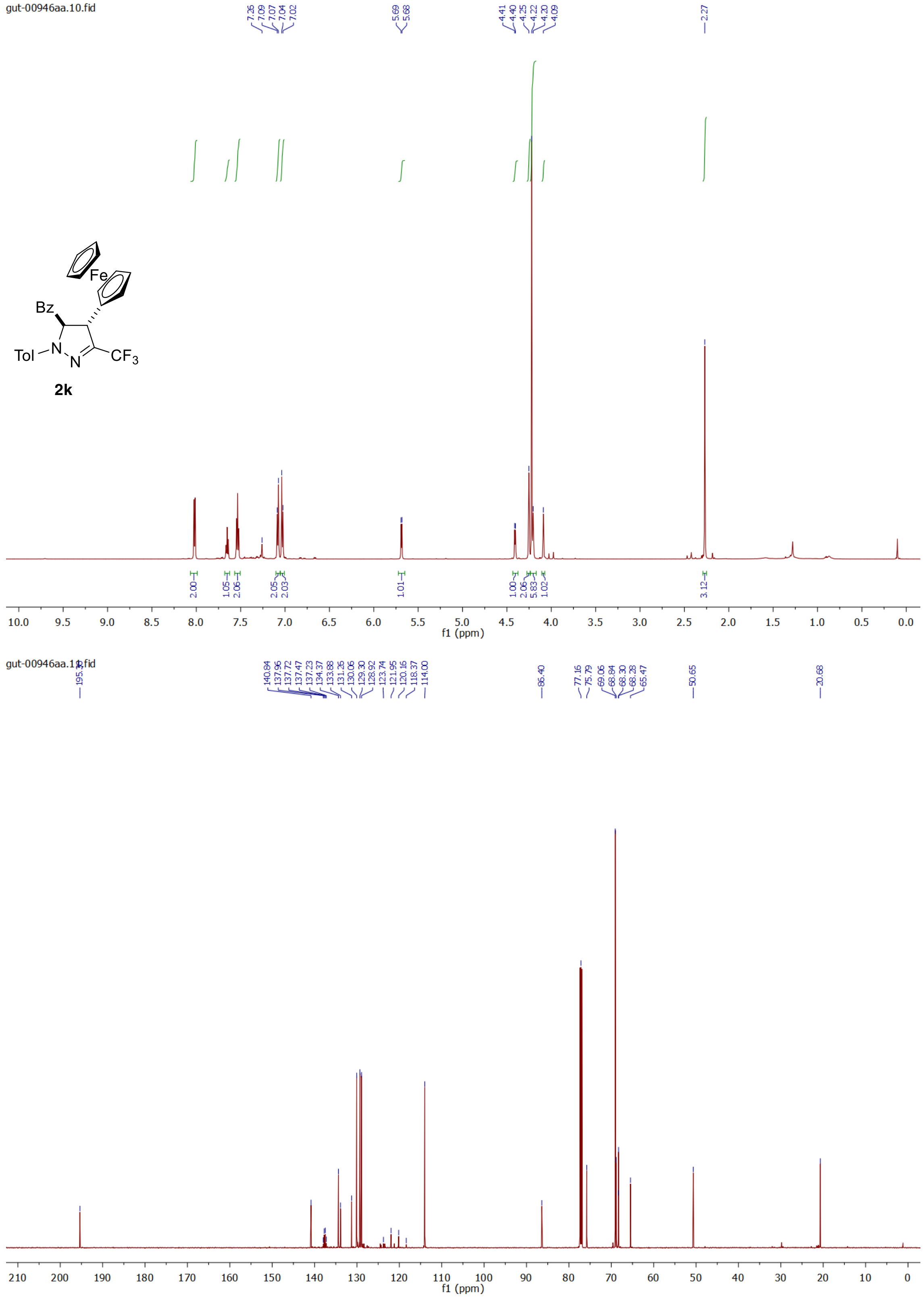

Fig S12. ^{1}H NMR (600 MHz, $CDCl_3$) and ^{13}C NMR (151 MHz, $CDCl_3$) spectra for compound **2k**.

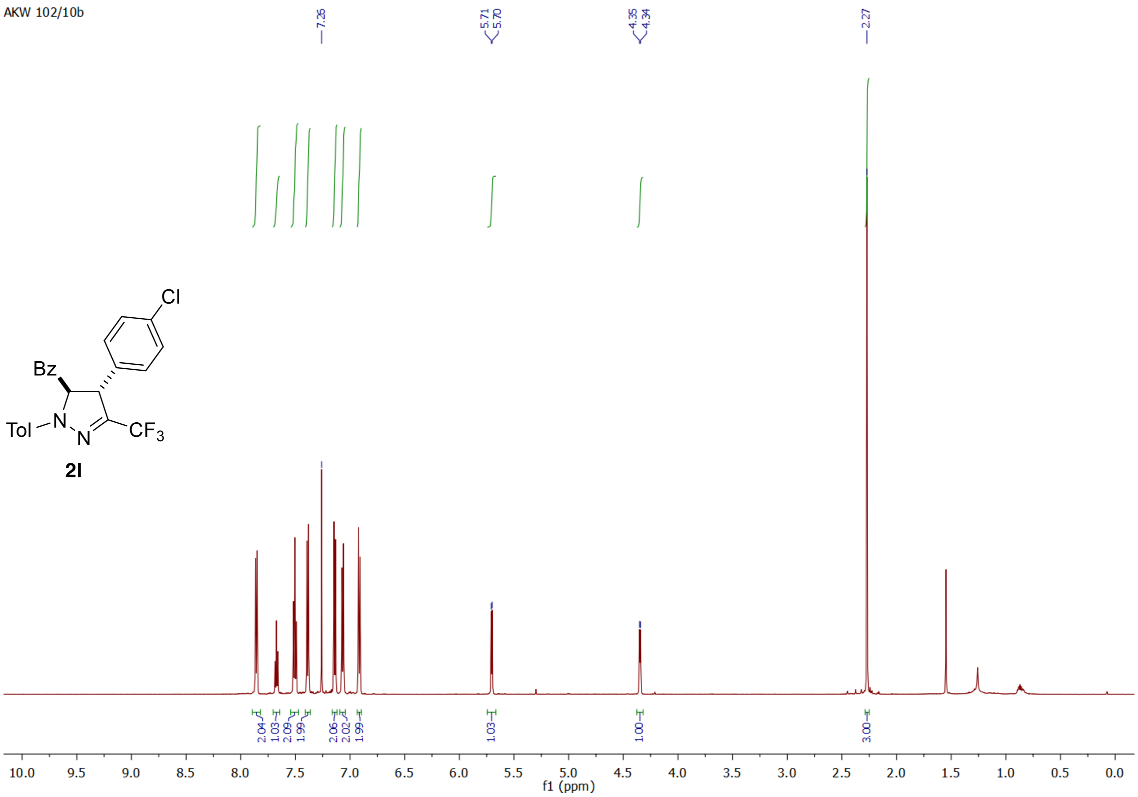

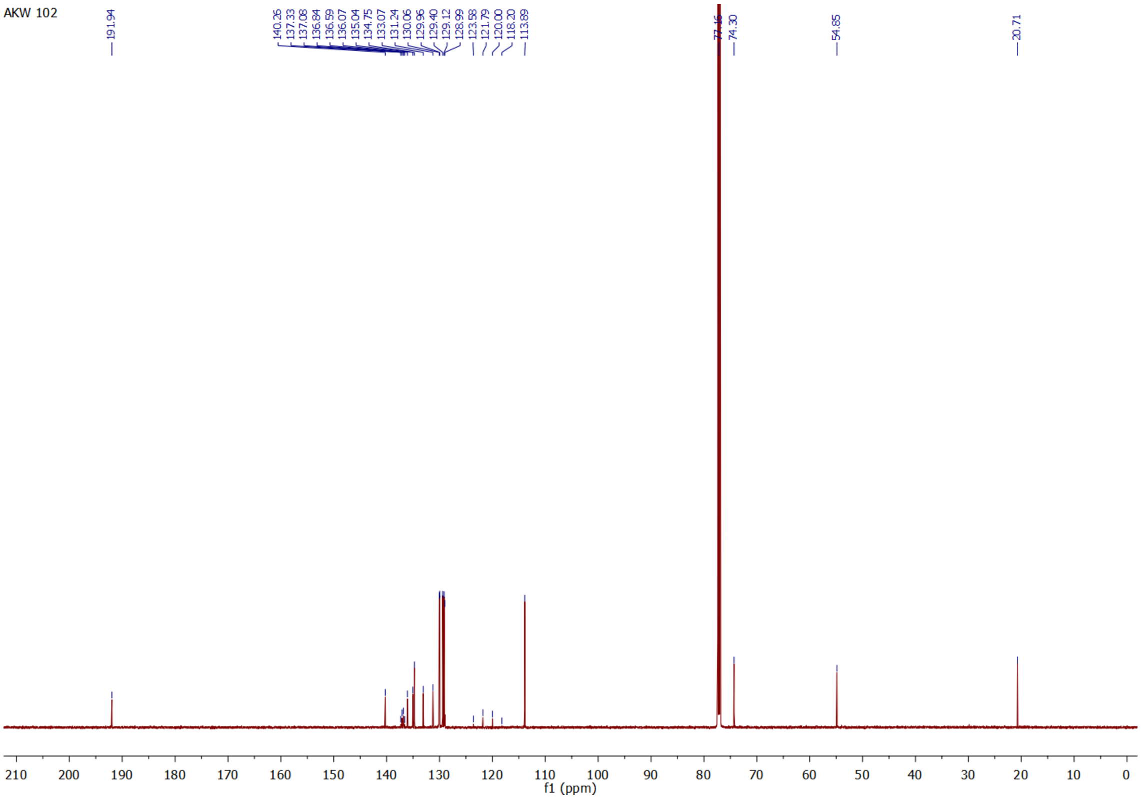

Fig S13. ^{1}H NMR (600 MHz, $CDCl_3$) and ^{13}C NMR (151 MHz, $CDCl_3$) spectra for compound **2l**.

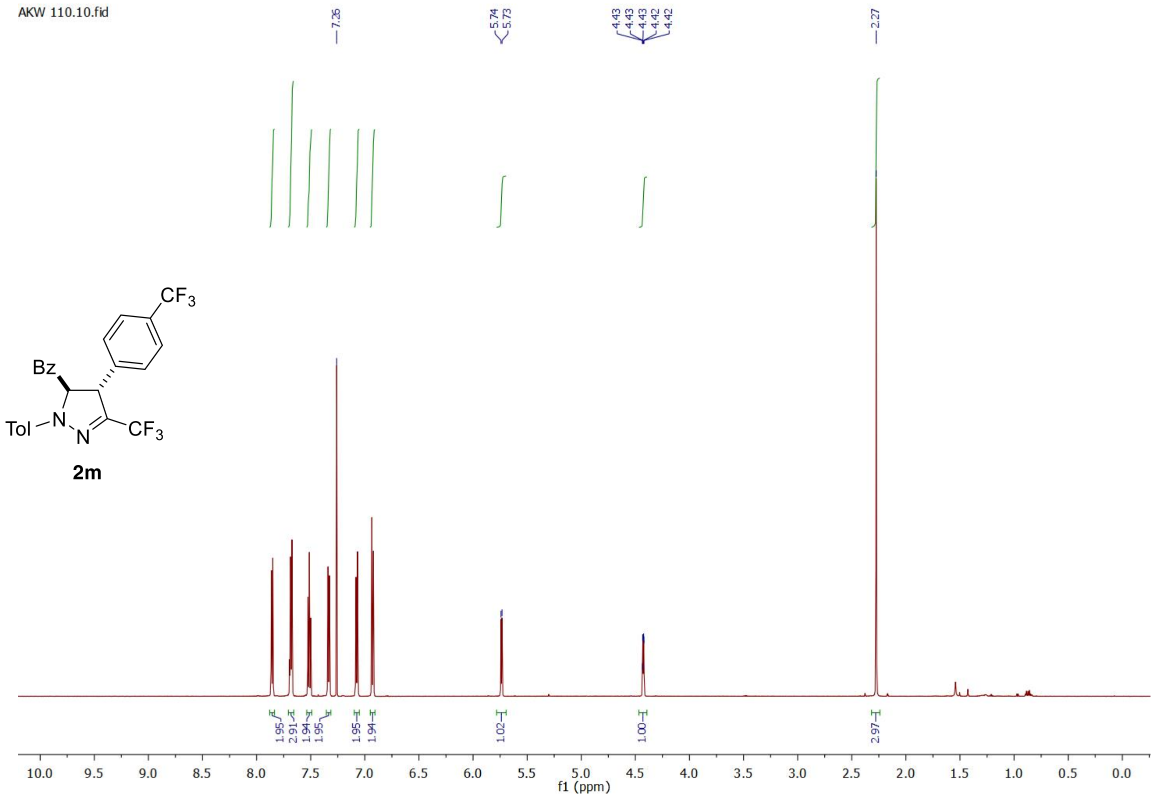

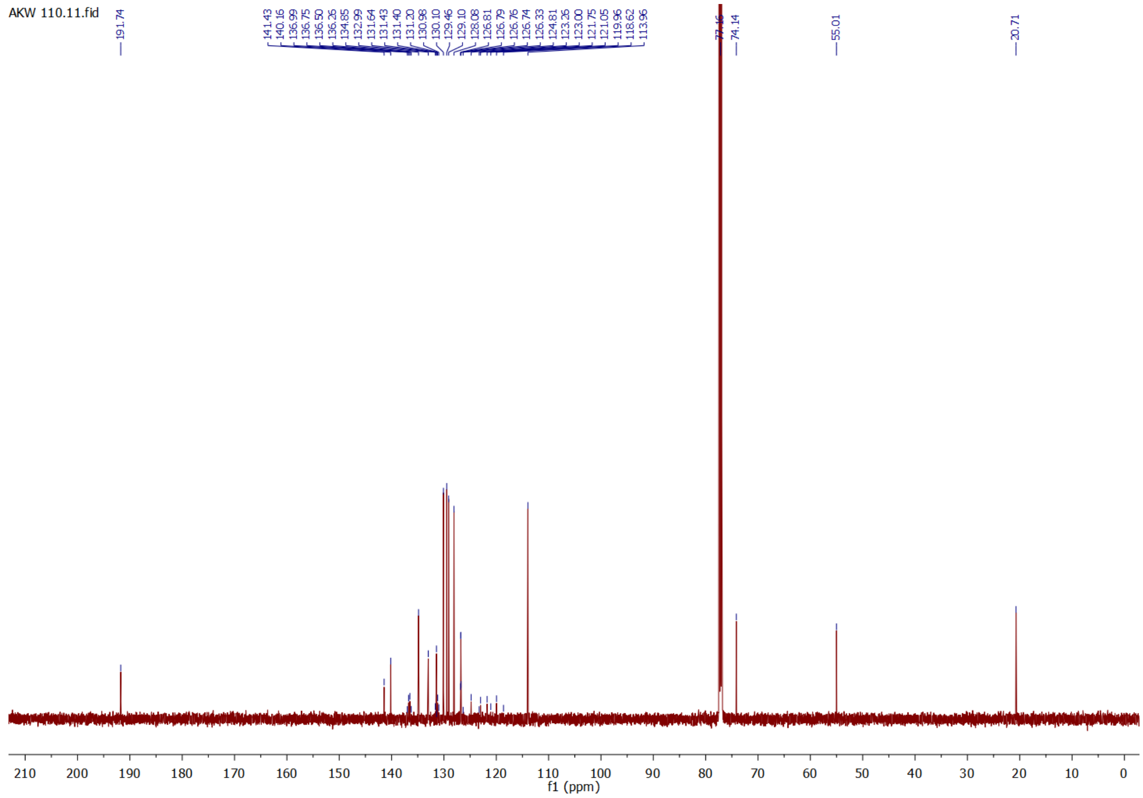

Fig S14. ^{1}H NMR (600 MHz, $CDCl_3$) and ^{13}C NMR (151 MHz, $CDCl_3$) spectra for compound **2m**.

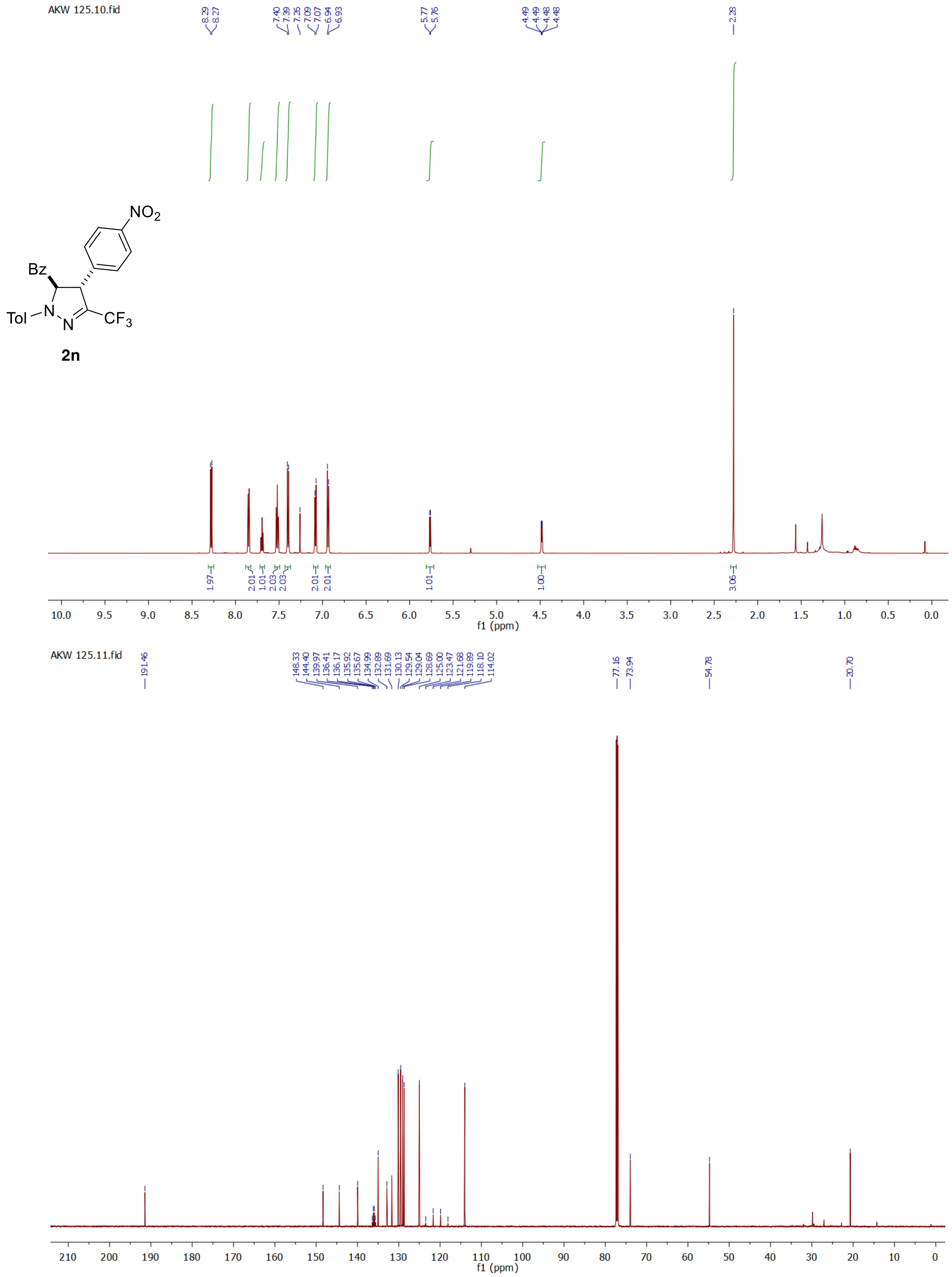

Fig S15. ^{1}H NMR (600 MHz, $CDCl_3$) and ^{13}C NMR (151 MHz, $CDCl_3$) spectra for compound **2n**.

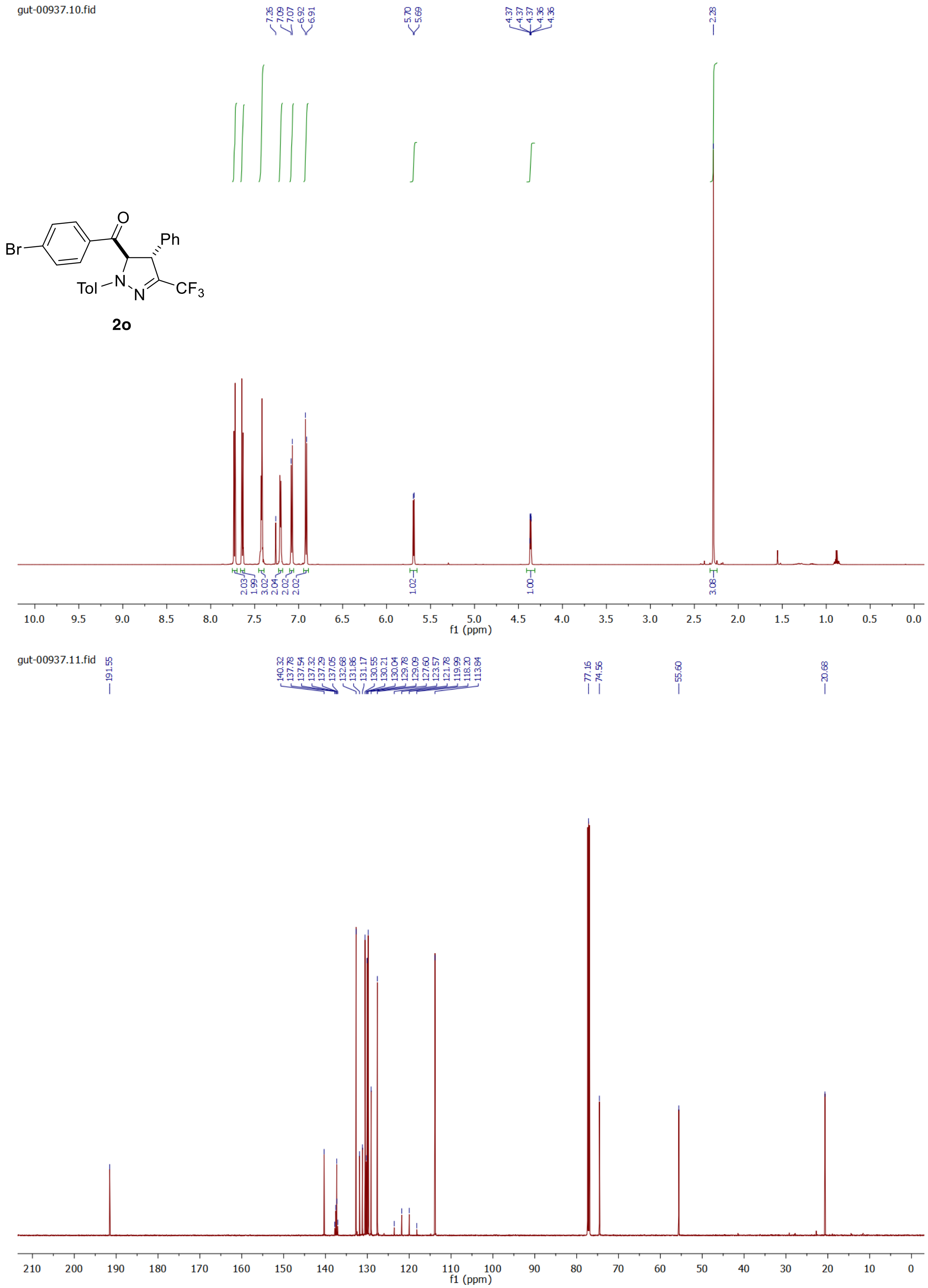

Fig S16. ^{1}H NMR (600 MHz, $CDCl_3$) and ^{13}C NMR (151 MHz, $CDCl_3$) spectra for compound **2o**.

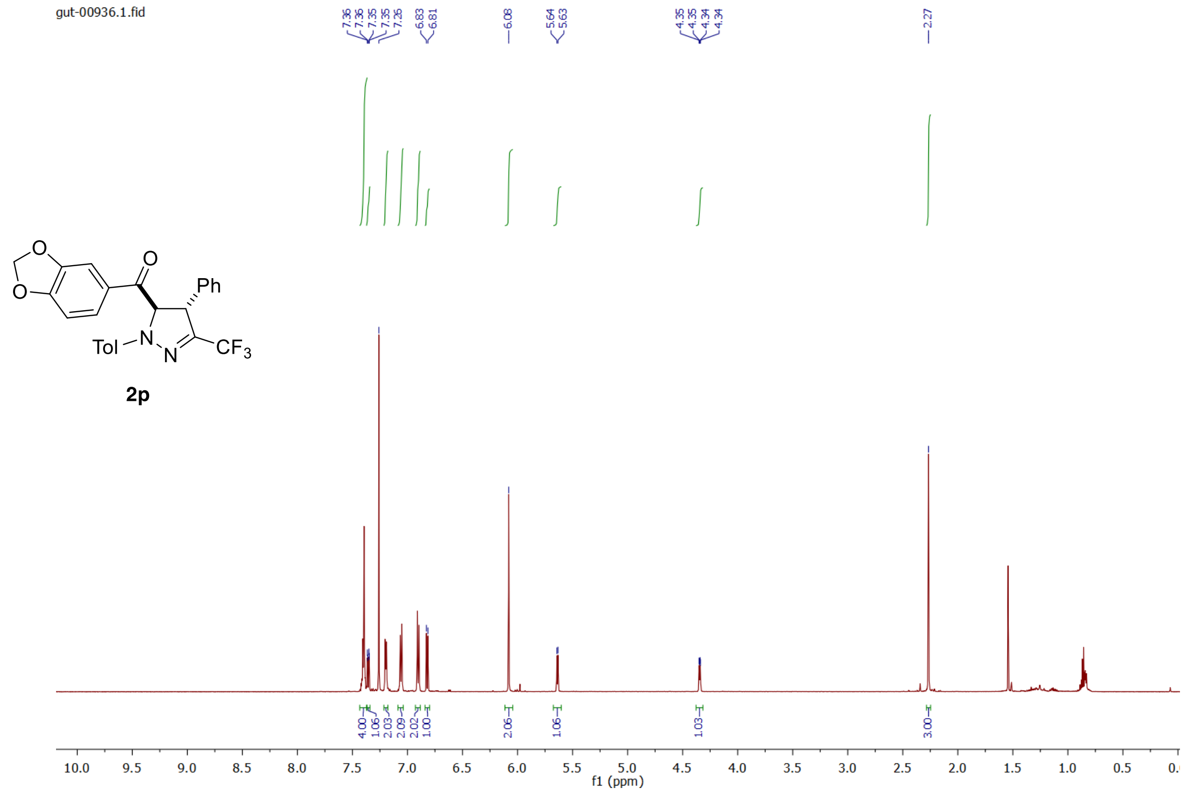

2p

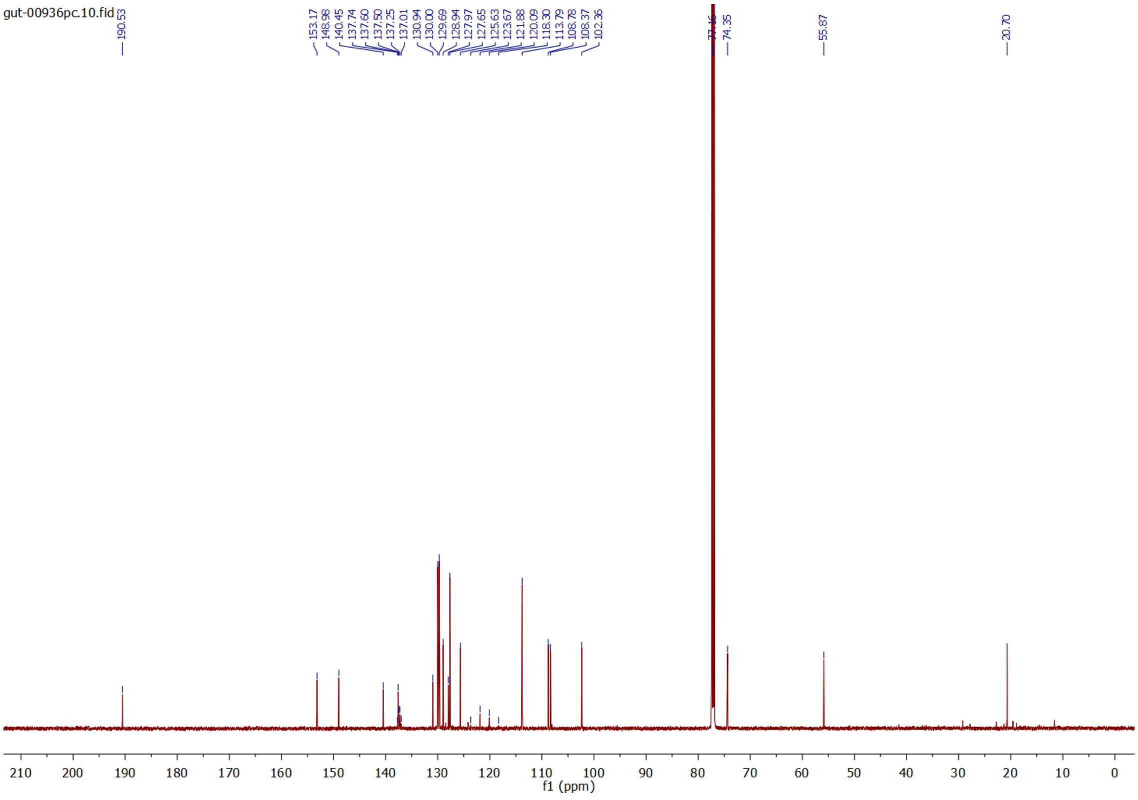

Fig S17. ^{1}H NMR (600 MHz, $CDCl_3$) and ^{13}C NMR (151 MHz, $CDCl_3$) spectra for compound **2p**.

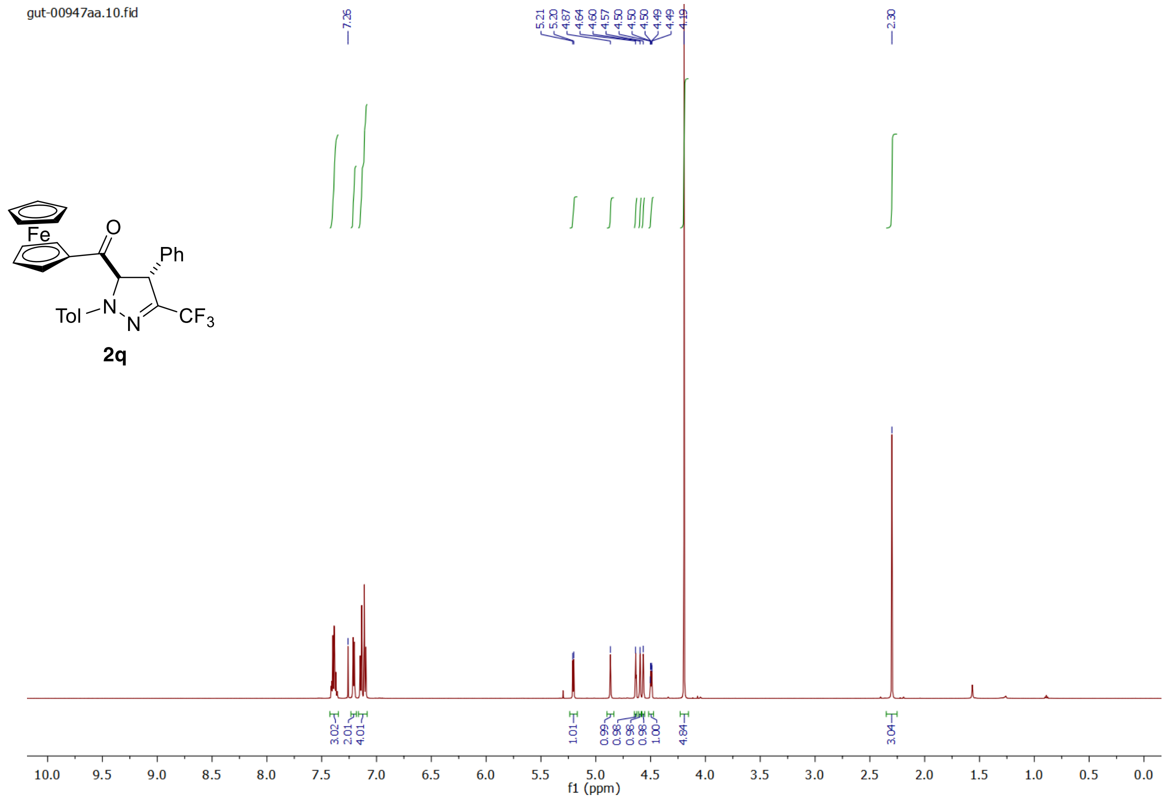

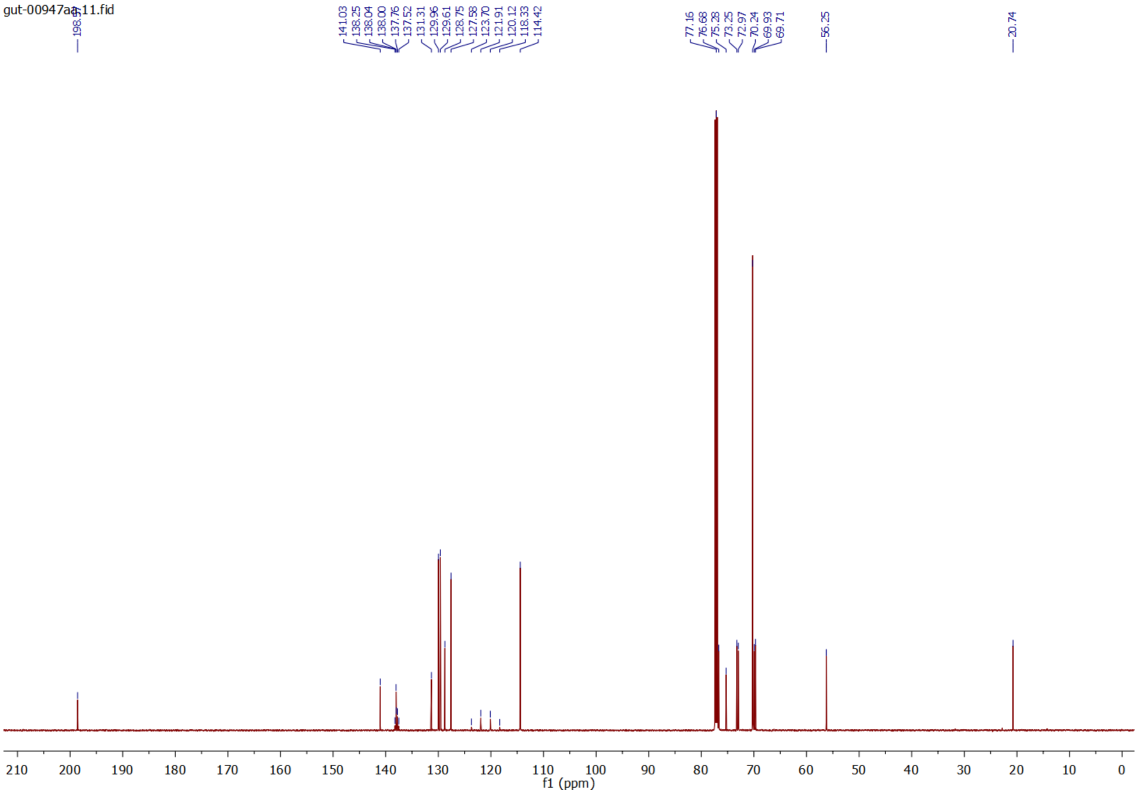

Fig S18. ^{1}H NMR (600 MHz, $CDCl_3$) and ^{13}C NMR (151 MHz, $CDCl_3$) spectra for compound **2q**.

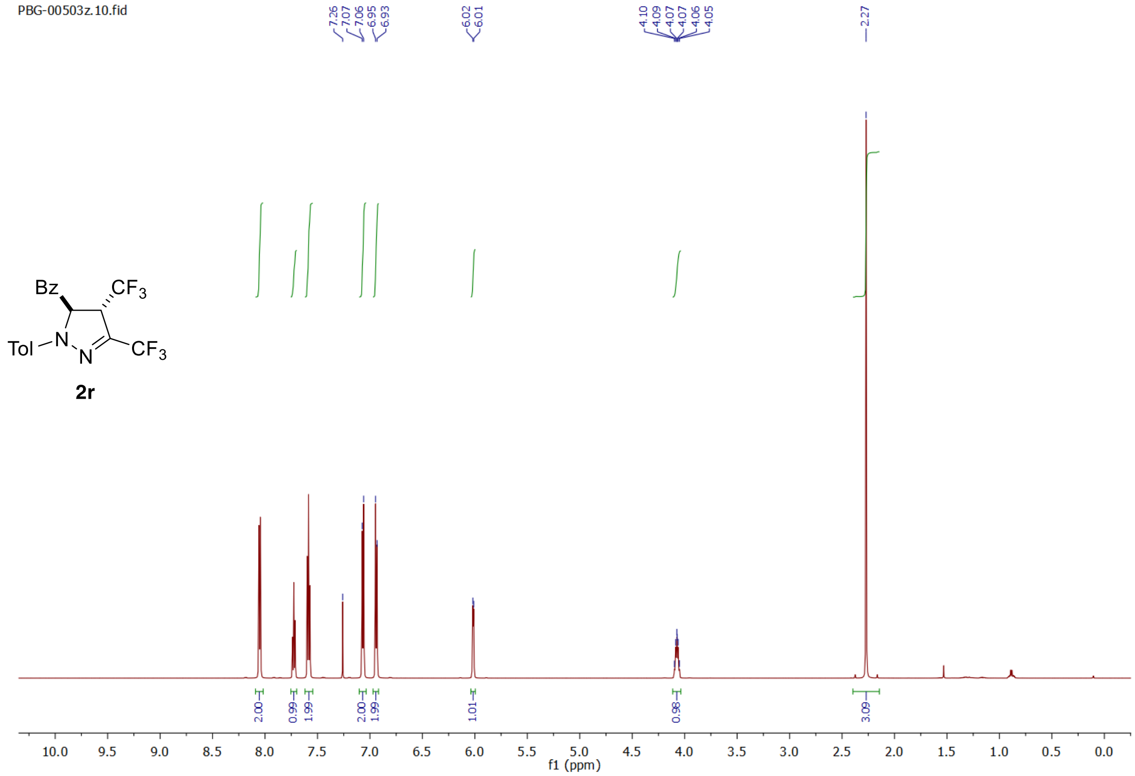

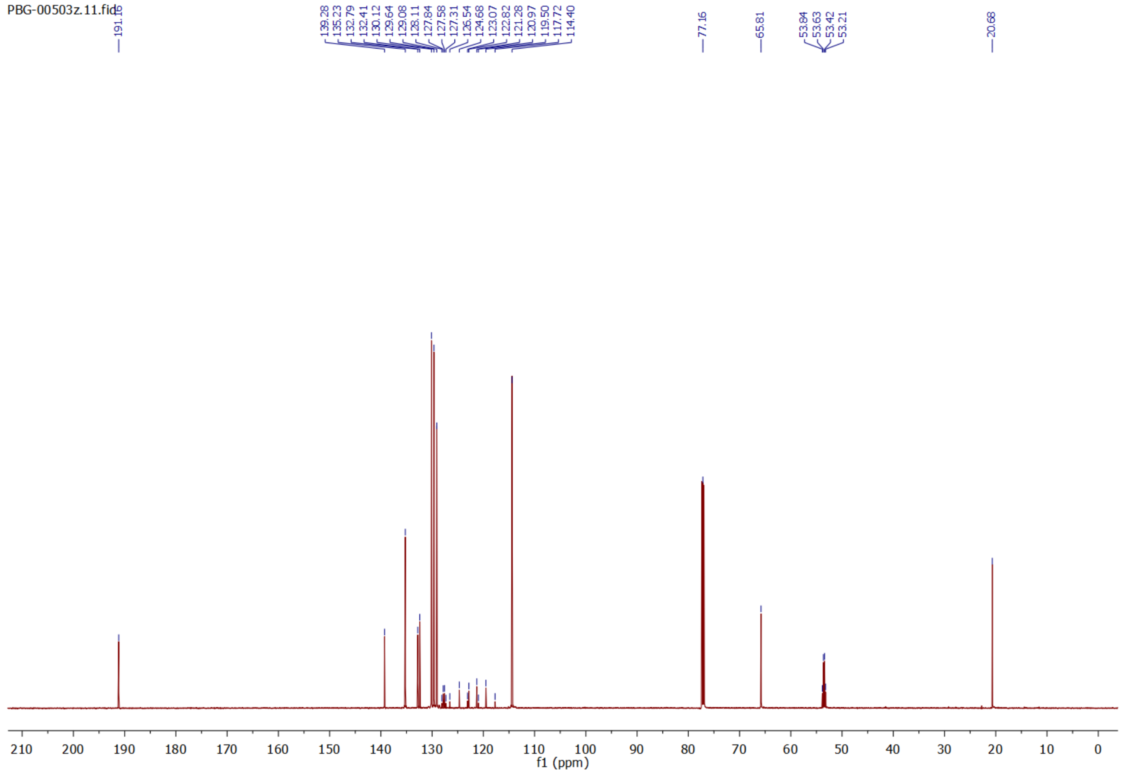

Fig S19. ^{1}H NMR (600 MHz, $CDCl_3$) and ^{13}C NMR (151 MHz, $CDCl_3$) spectra for compound **2r**.

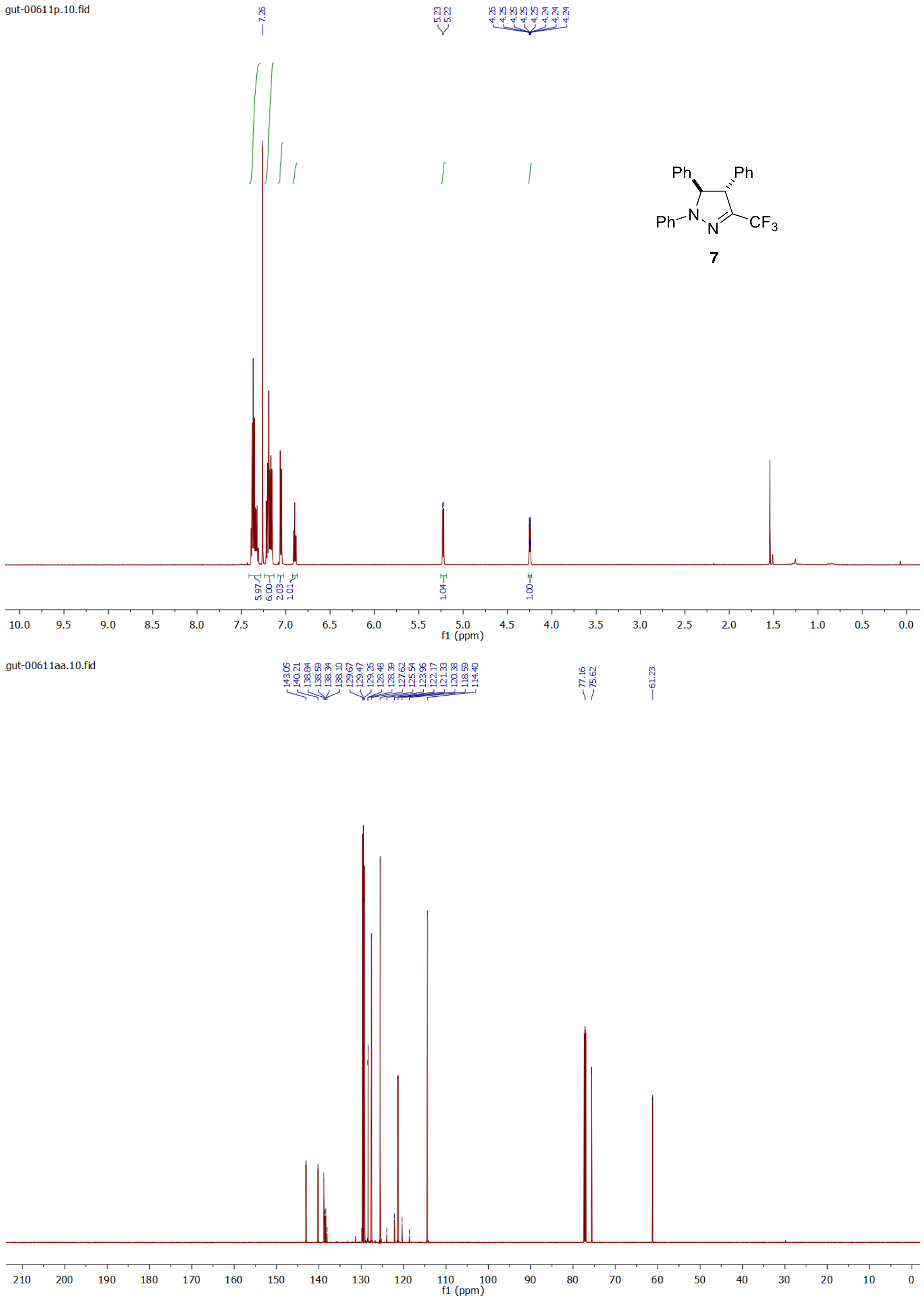

Fig S20. ^{1}H NMR (600 MHz, $CDCl_3$) and ^{13}C NMR (151 MHz, $CDCl_3$) spectra for compound **7**.

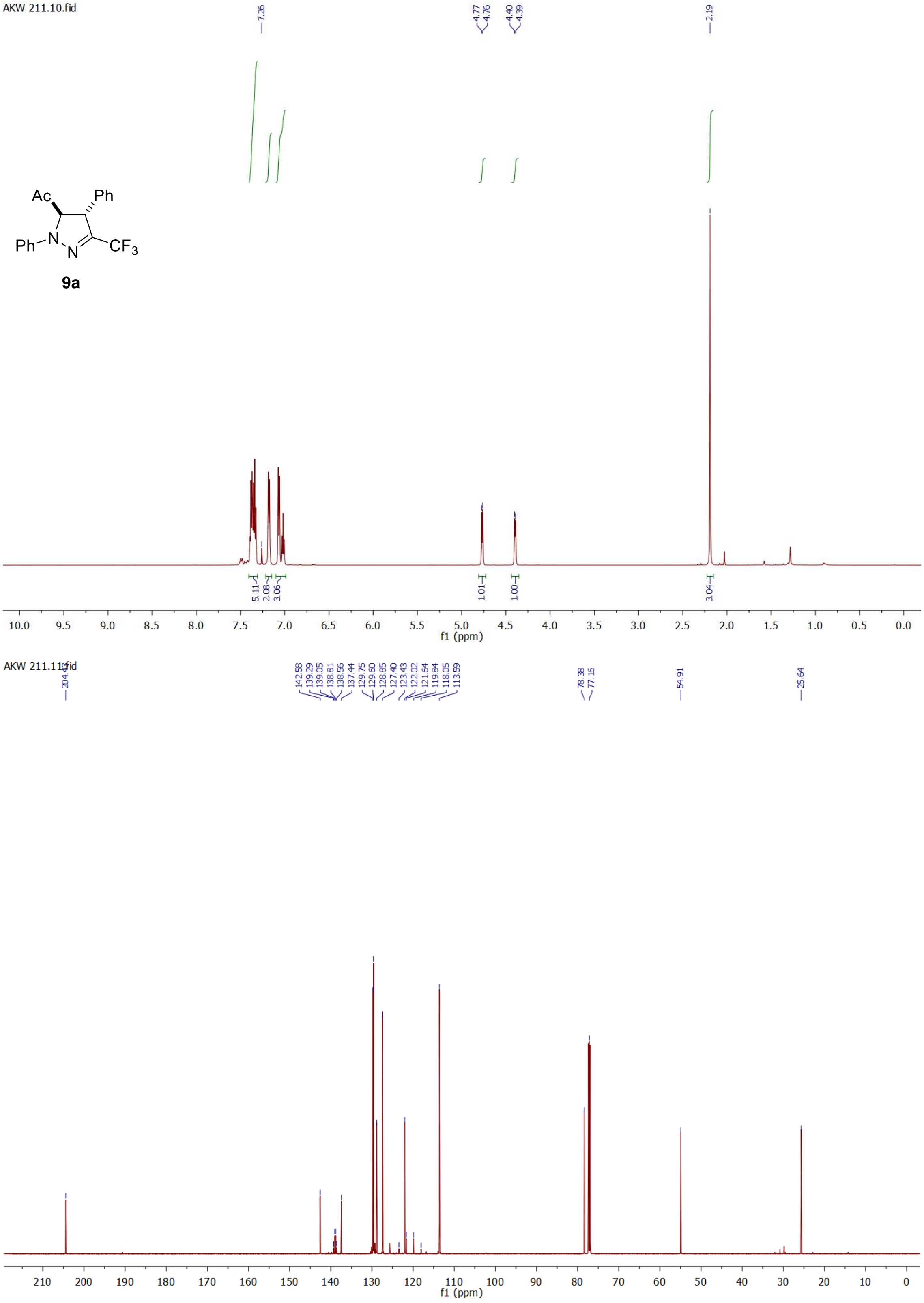

Fig S21. ^{1}H NMR (600 MHz, $CDCl_3$) and ^{13}C NMR (151 MHz, $CDCl_3$) spectra for compound **9a**.

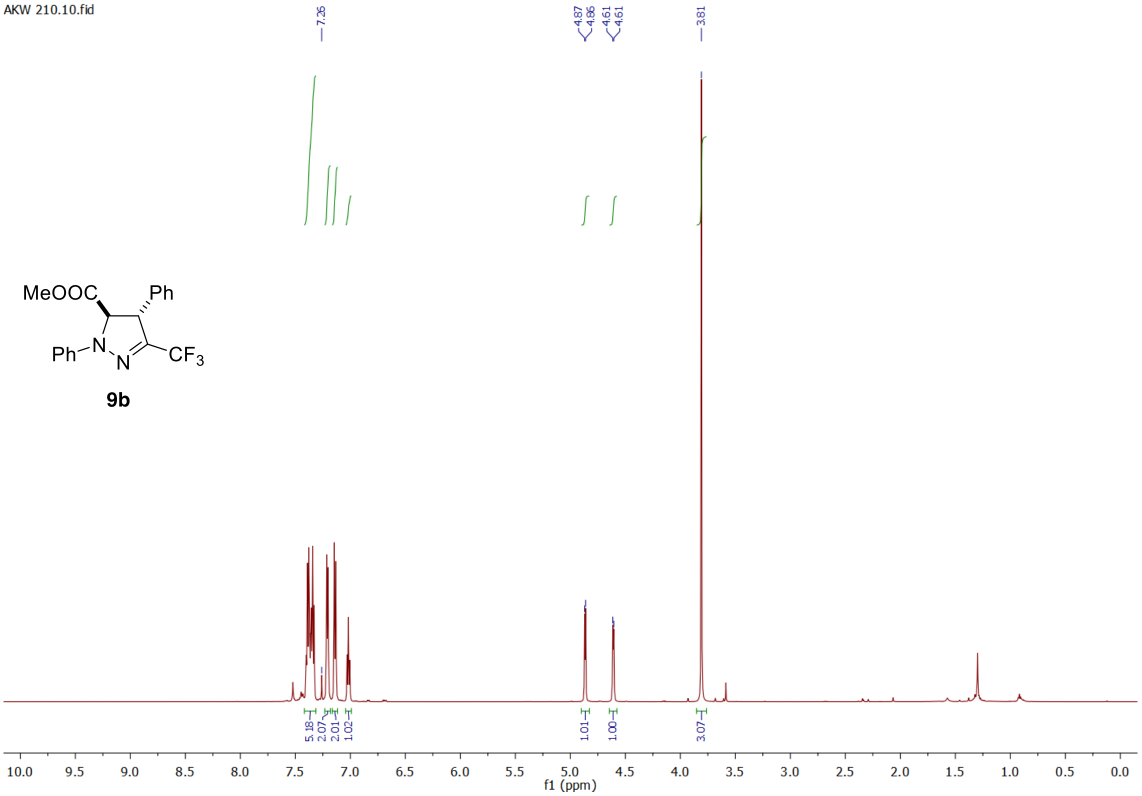

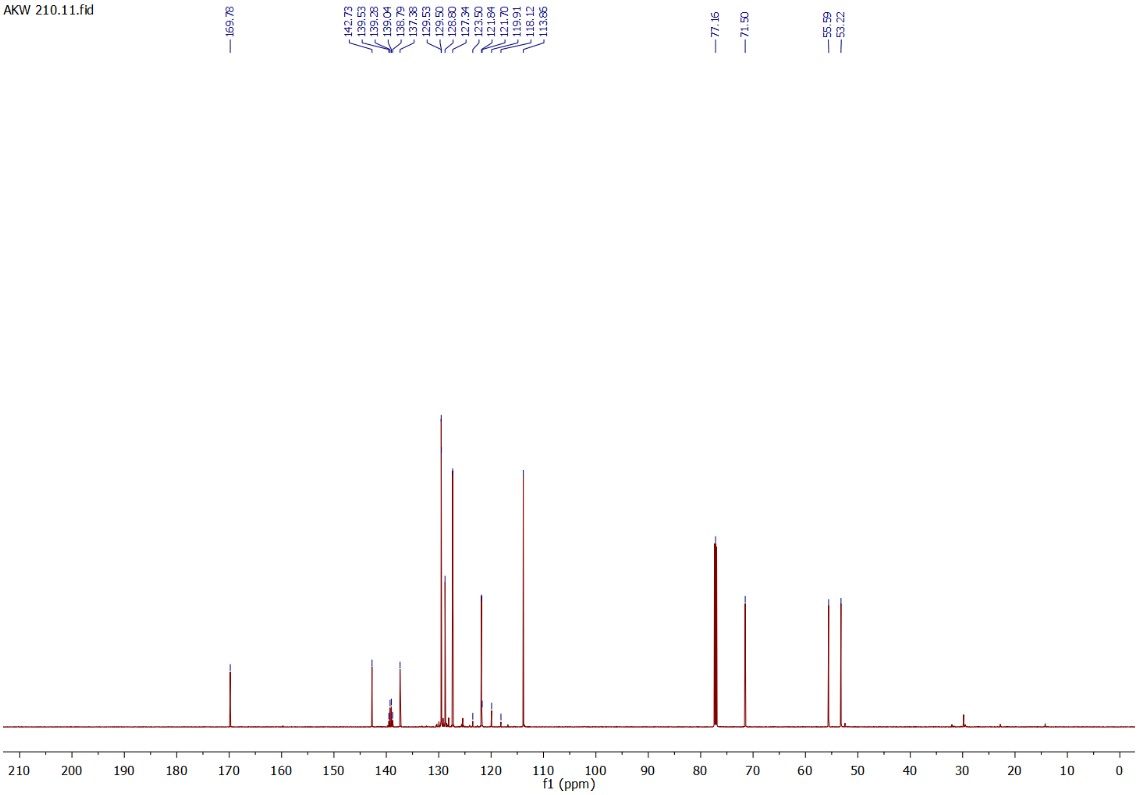

Fig S22. ^{1}H NMR (600 MHz, $CDCl_3$) and ^{13}C NMR (151 MHz, $CDCl_3$) spectra for compound **9b**.

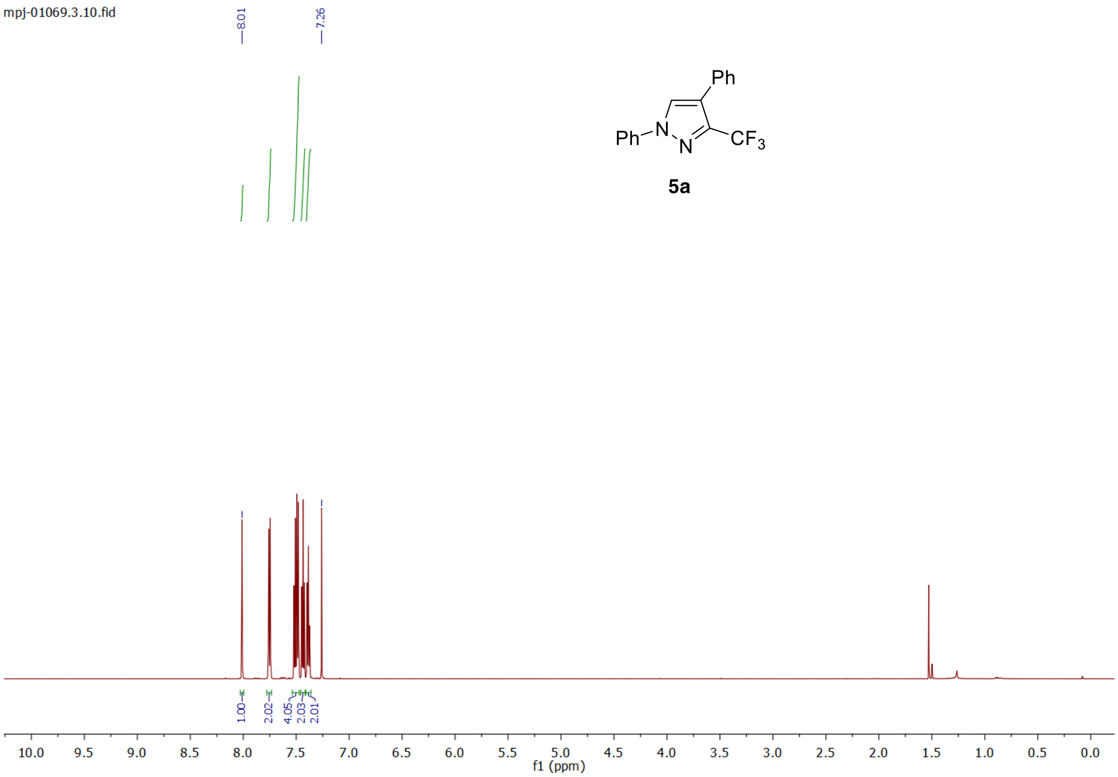

5a

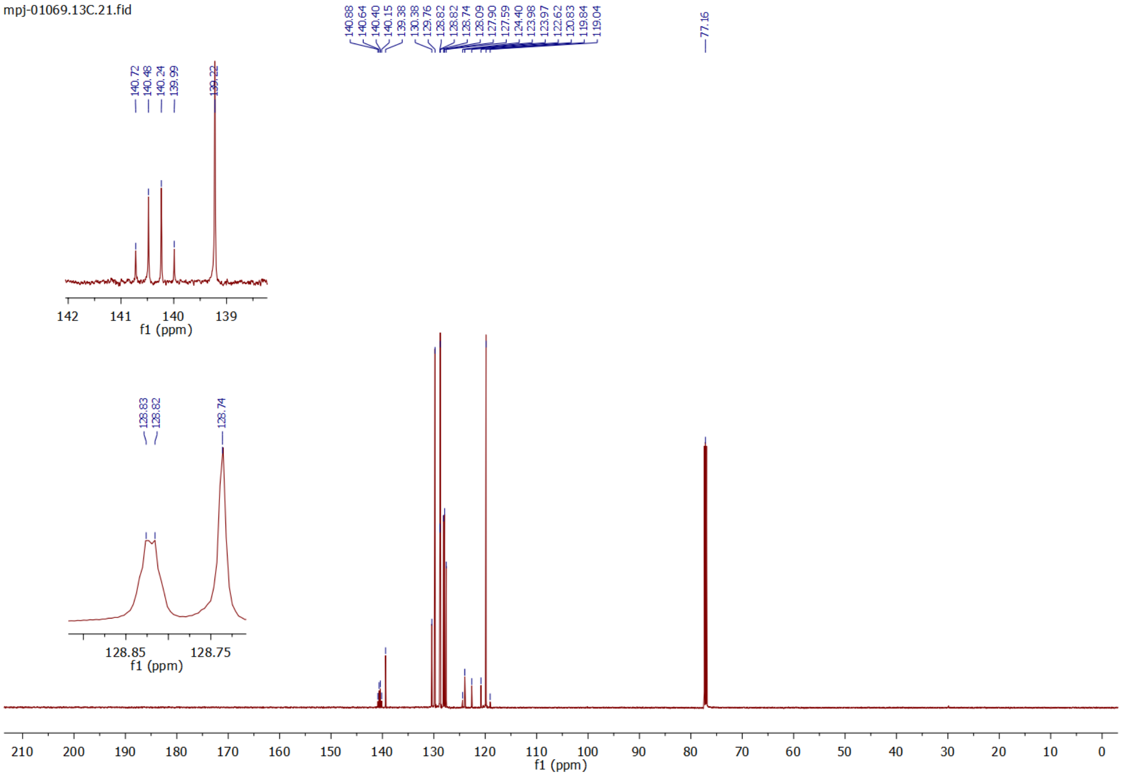

Fig S23. ^{1}H NMR (600 MHz, $CDCl_3$) and ^{13}C NMR (151 MHz, $CDCl_3$) spectra for compound **5a**.

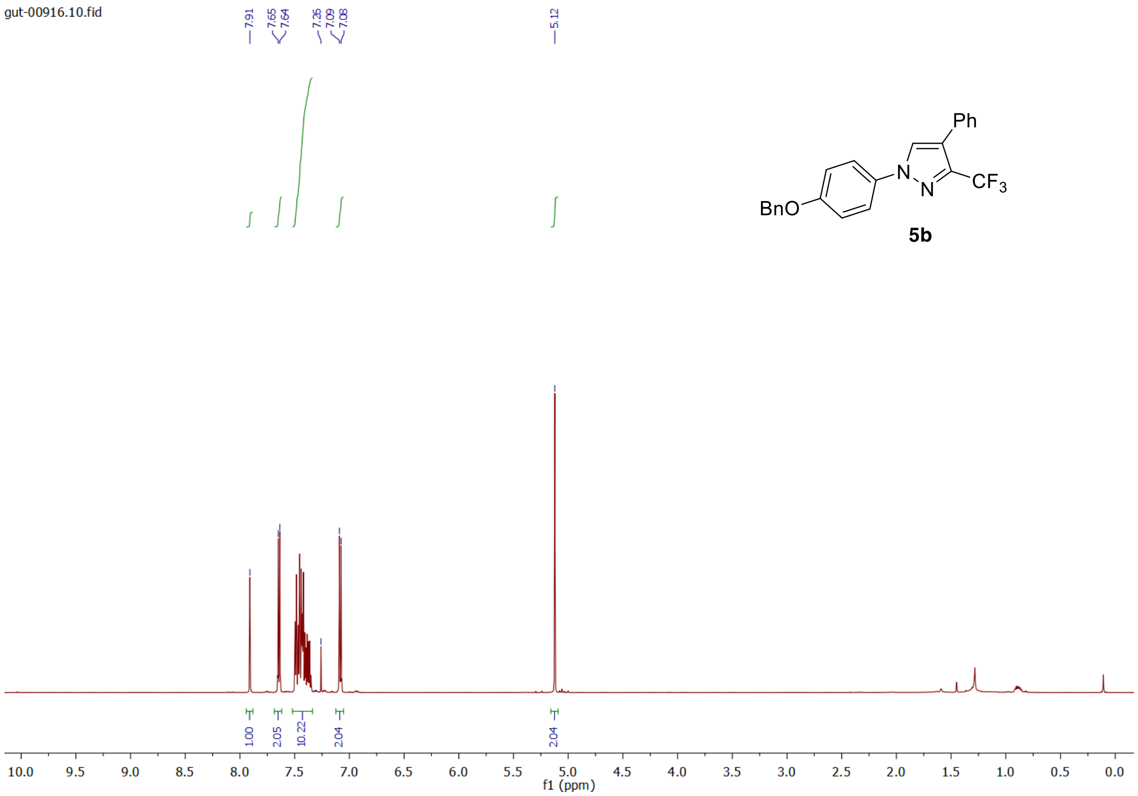

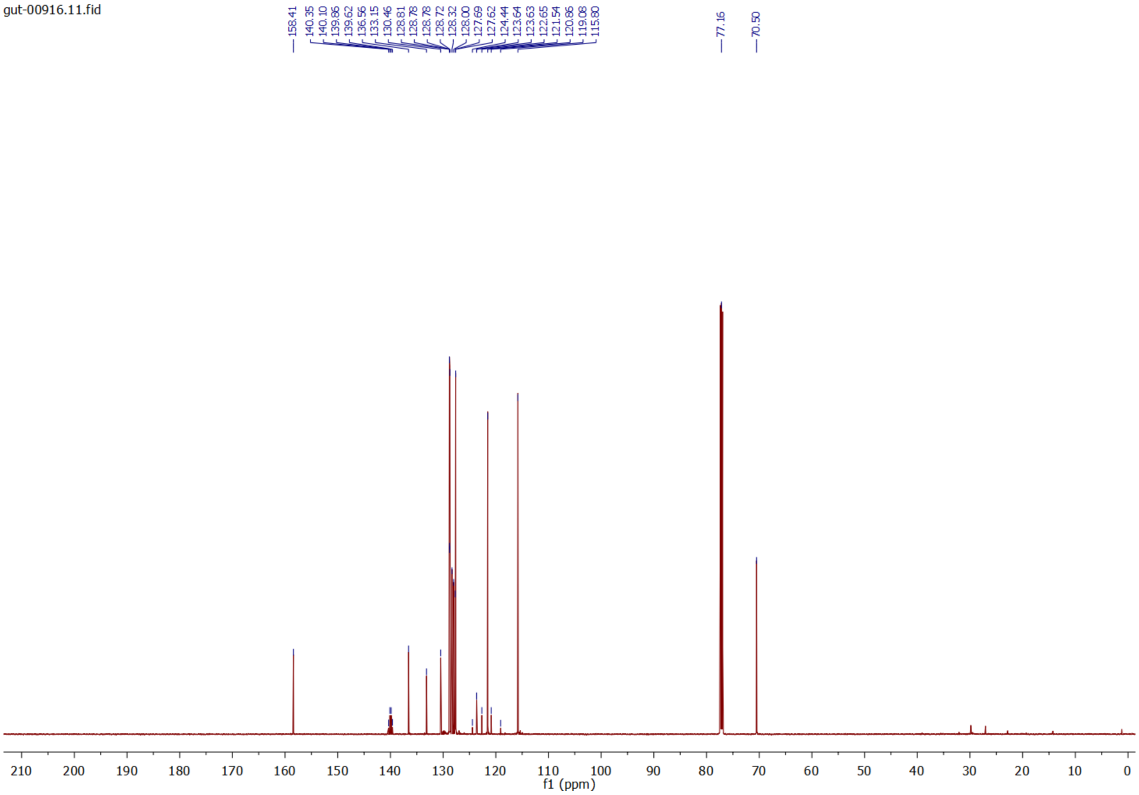

Fig S24. ^{1}H NMR (600 MHz, $CDCl_3$) and ^{13}C NMR (151 MHz, $CDCl_3$) spectra for compound **5b**.

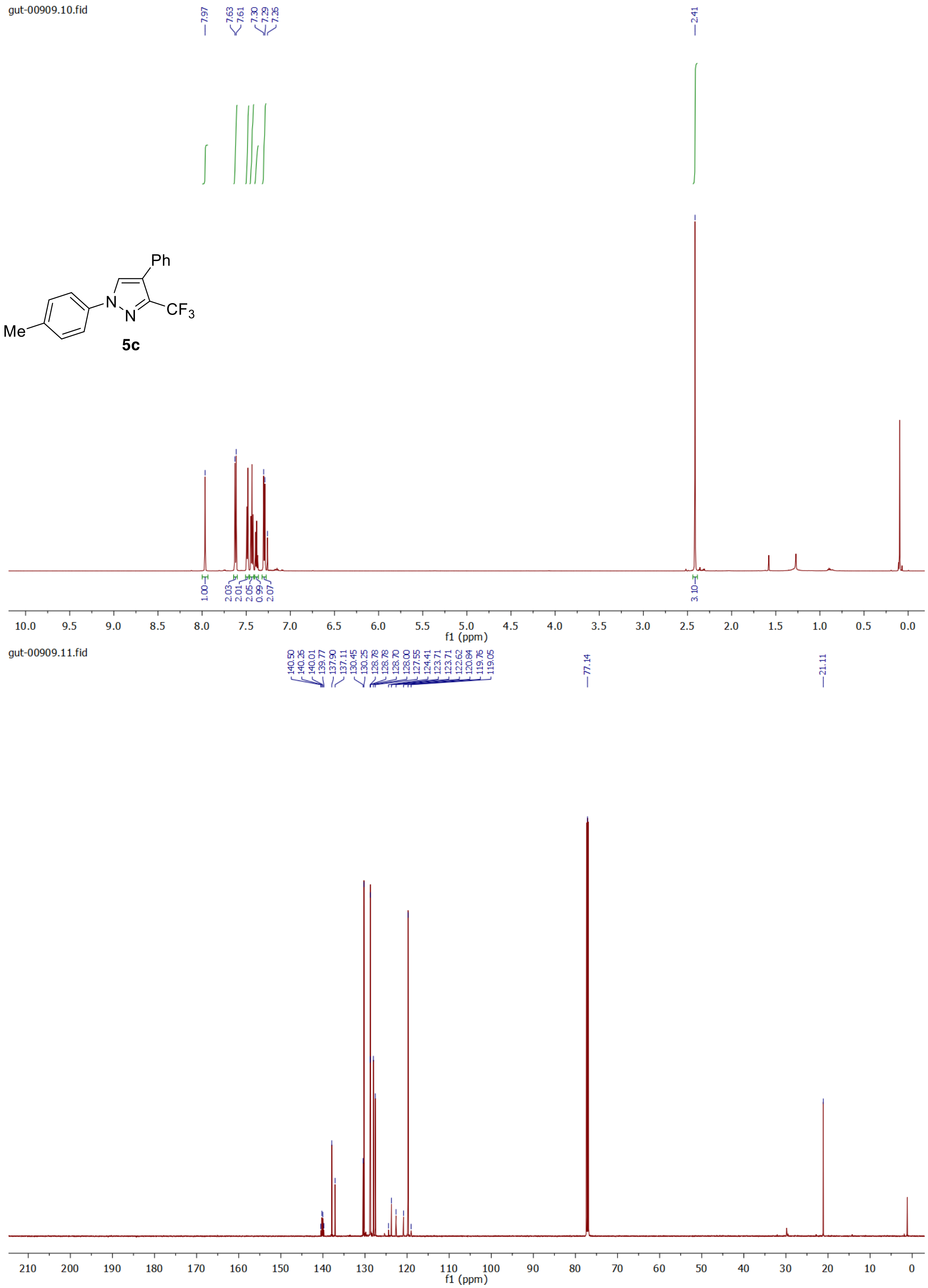

Fig S25. ^{1}H NMR (600 MHz, $CDCl_3$) and ^{13}C NMR (151 MHz, $CDCl_3$) spectra for compound **5c**.

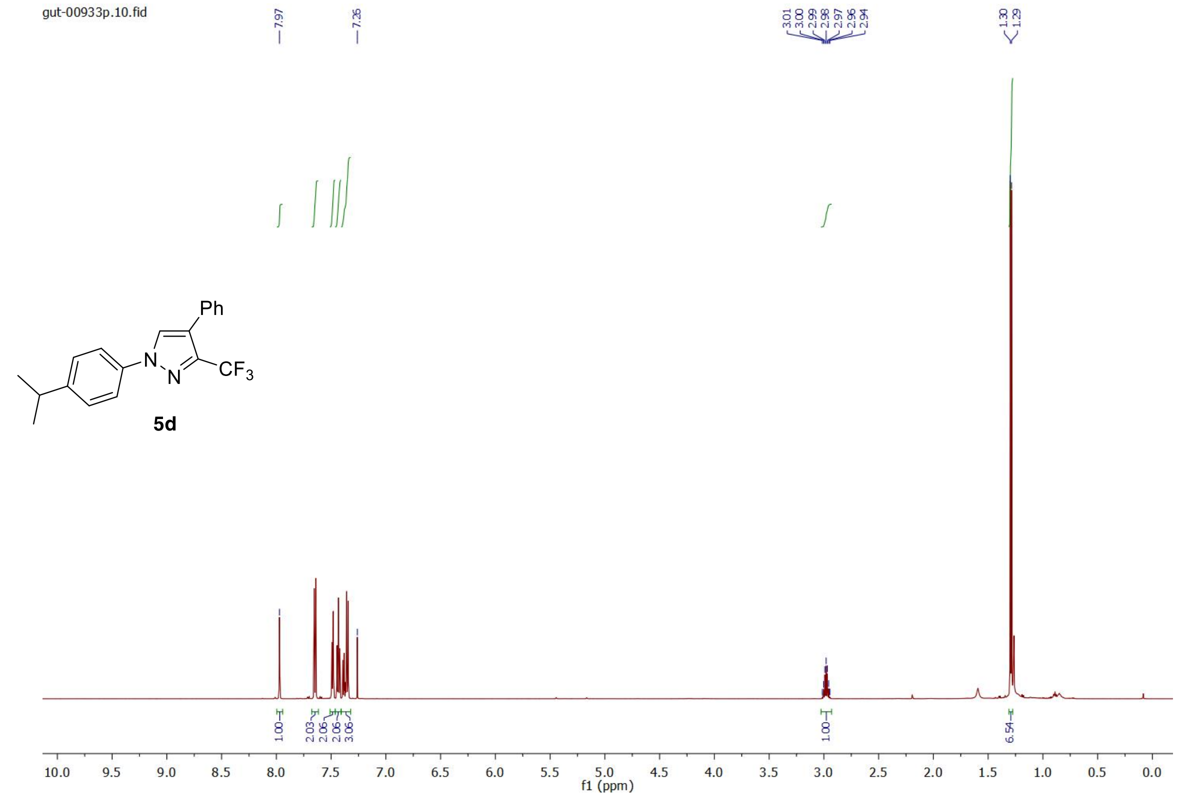

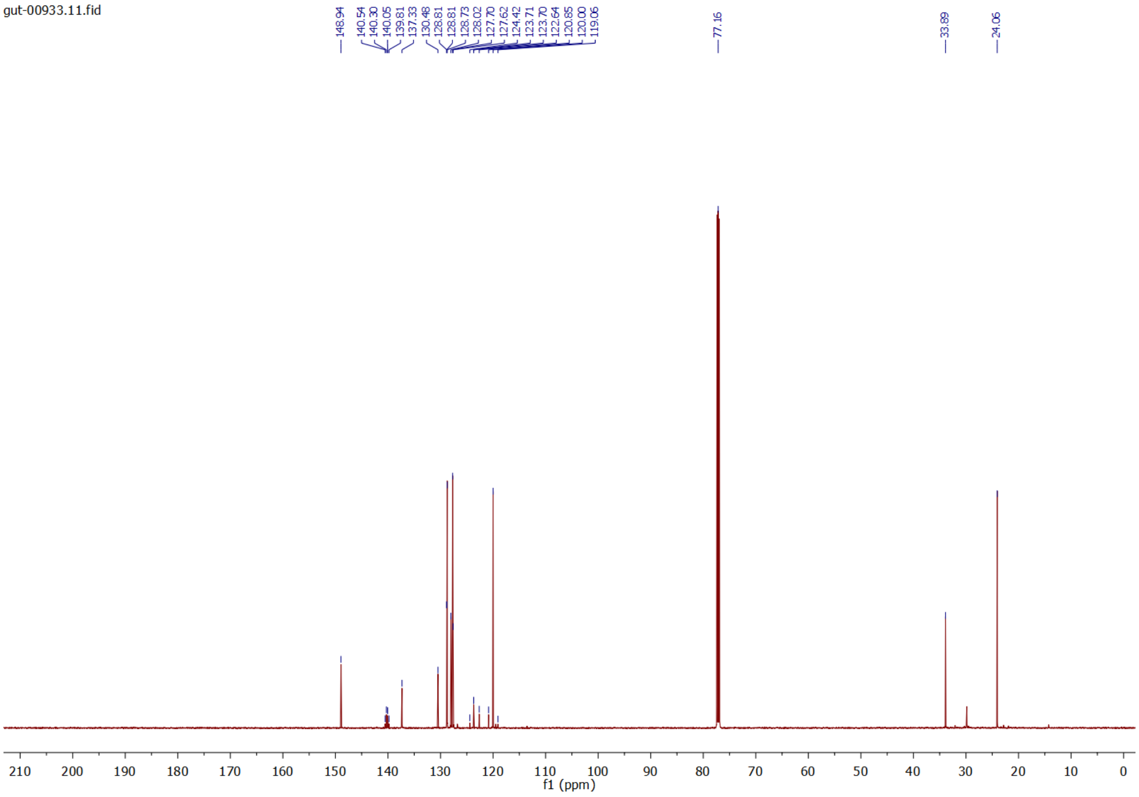

Fig S26. ^{1}H NMR (600 MHz, $CDCl_3$) and ^{13}C NMR (151 MHz, $CDCl_3$) spectra for compound **5d**.

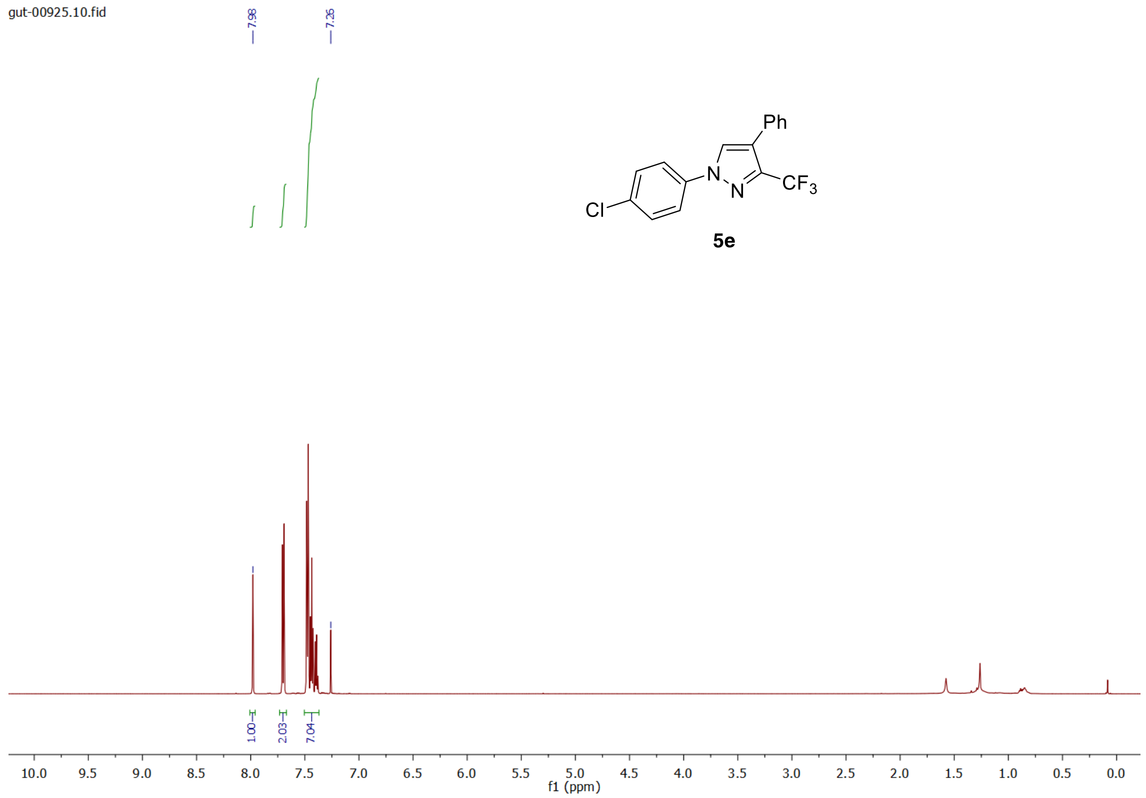

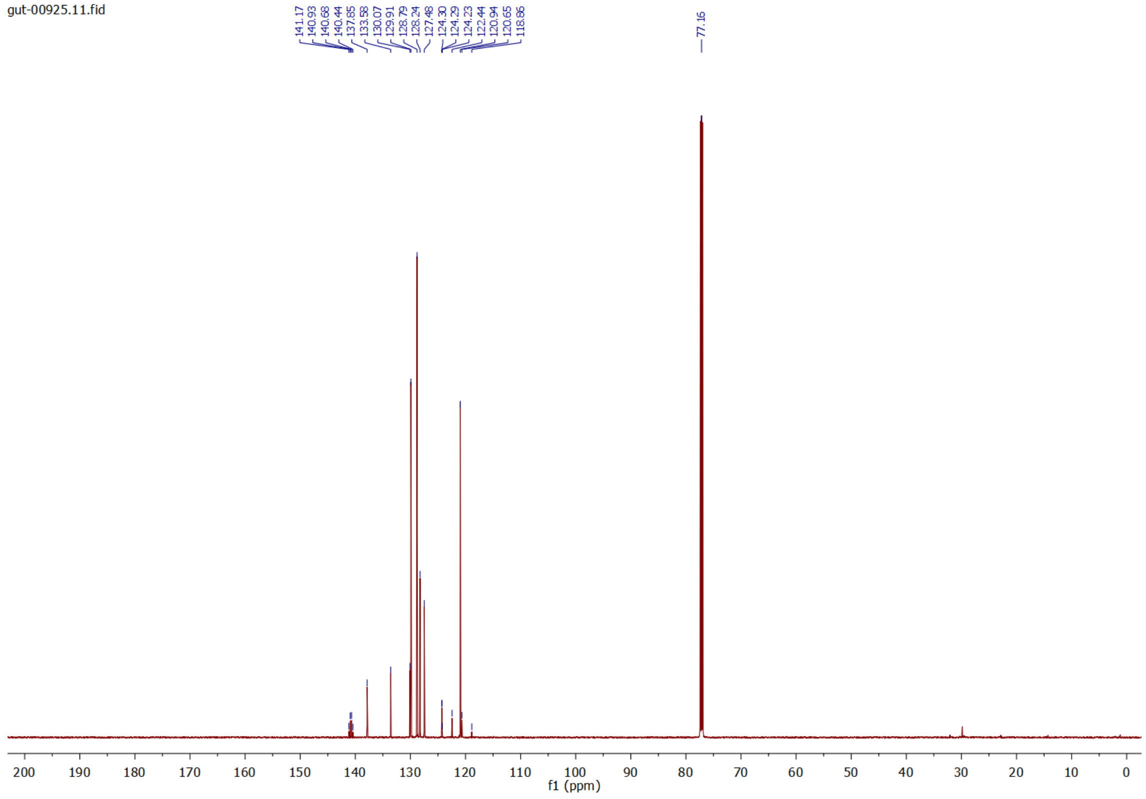

Fig S27. ^{1}H NMR (600 MHz, $CDCl_3$) and ^{13}C NMR (151 MHz, $CDCl_3$) spectra for compound **5e**.

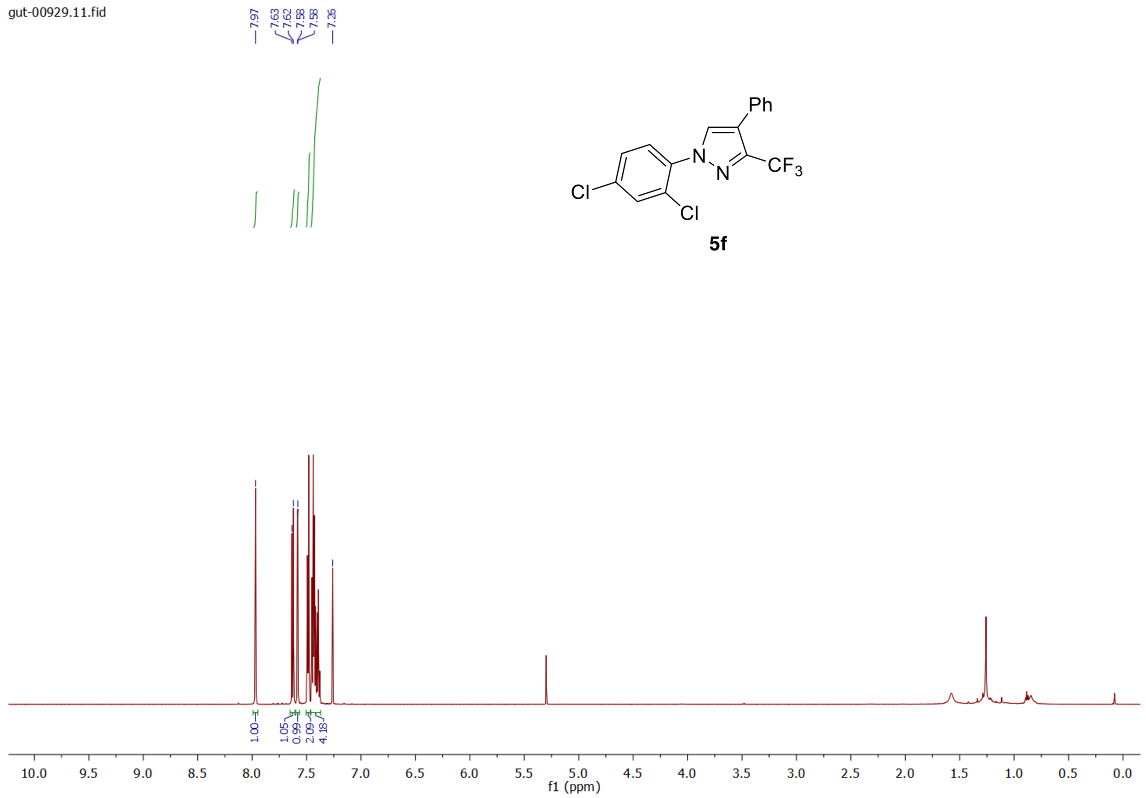

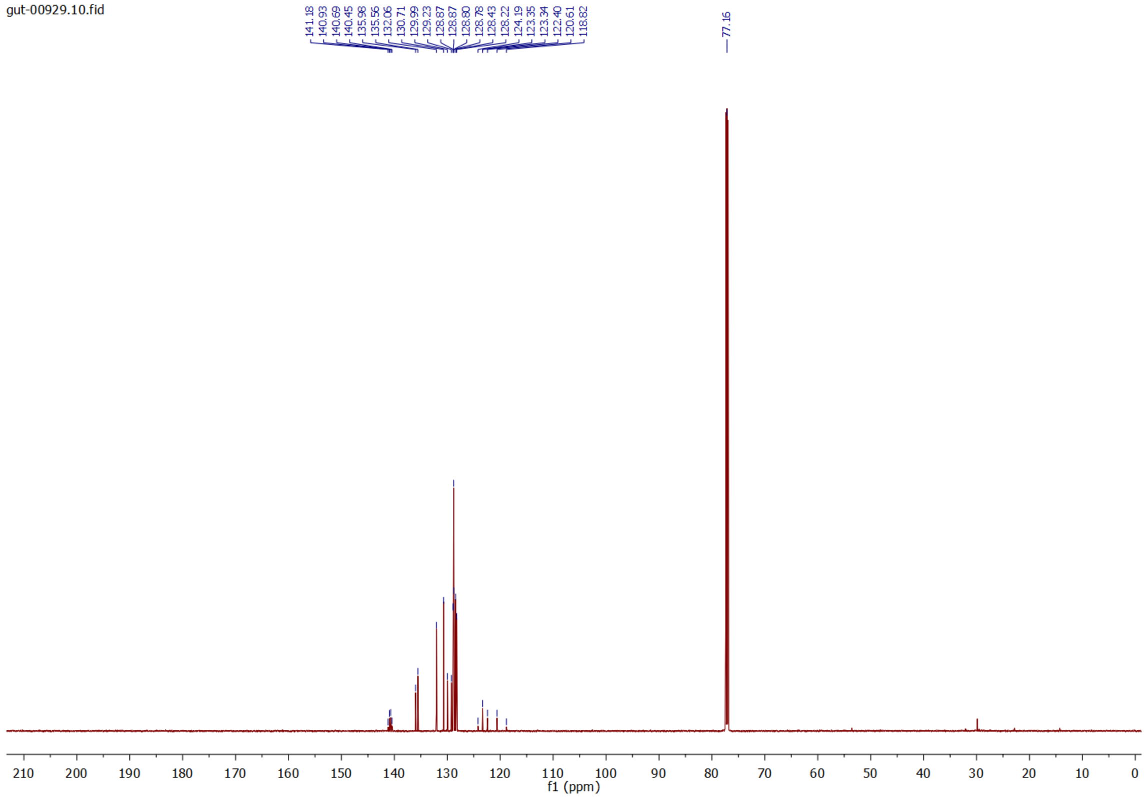

Fig S28. ^{1}H NMR (600 MHz, $CDCl_3$) and ^{13}C NMR (151 MHz, $CDCl_3$) spectra for compound **5f**.

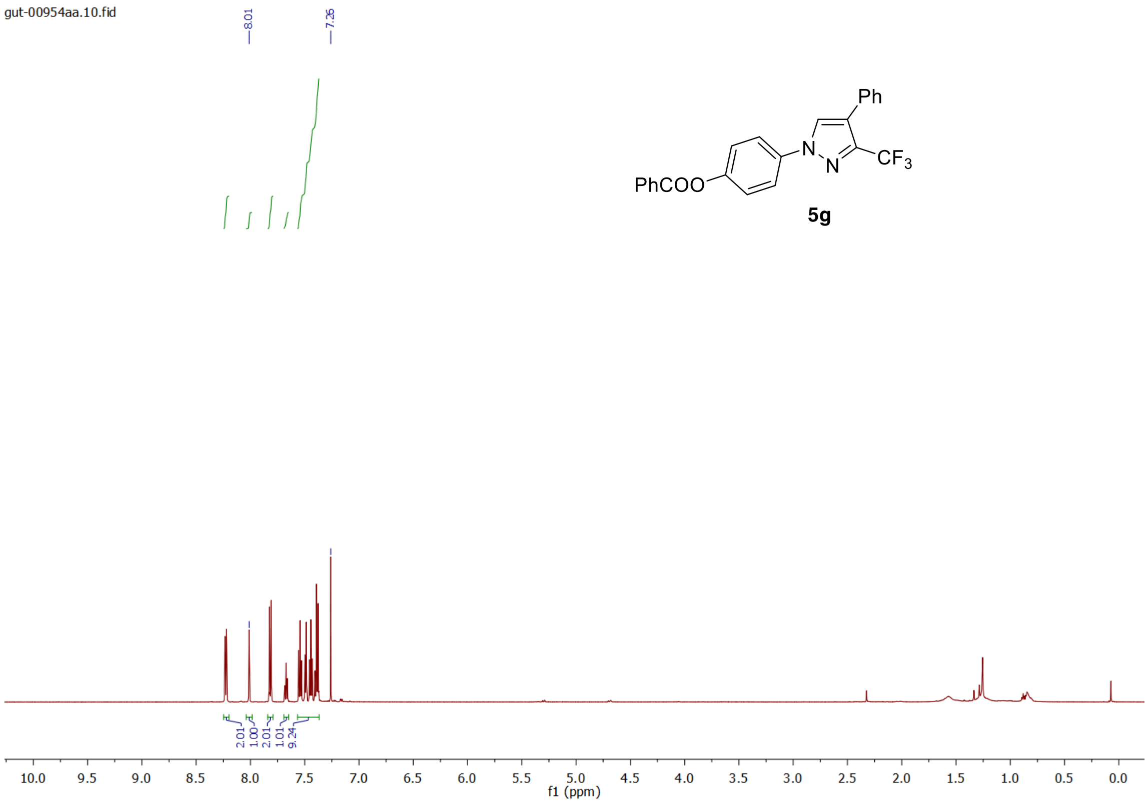

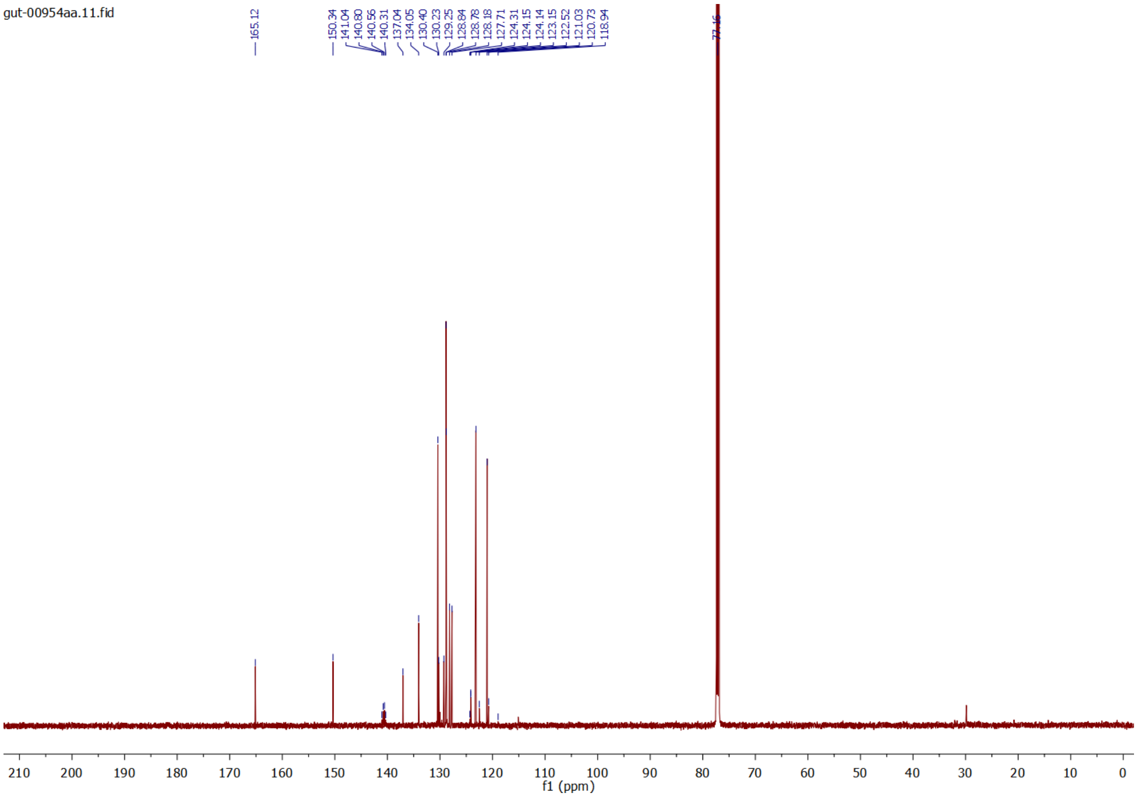

Fig S29. ^{1}H NMR (600 MHz, $CDCl_3$) and ^{13}C NMR (151 MHz, $CDCl_3$) spectra for compound **5g**.

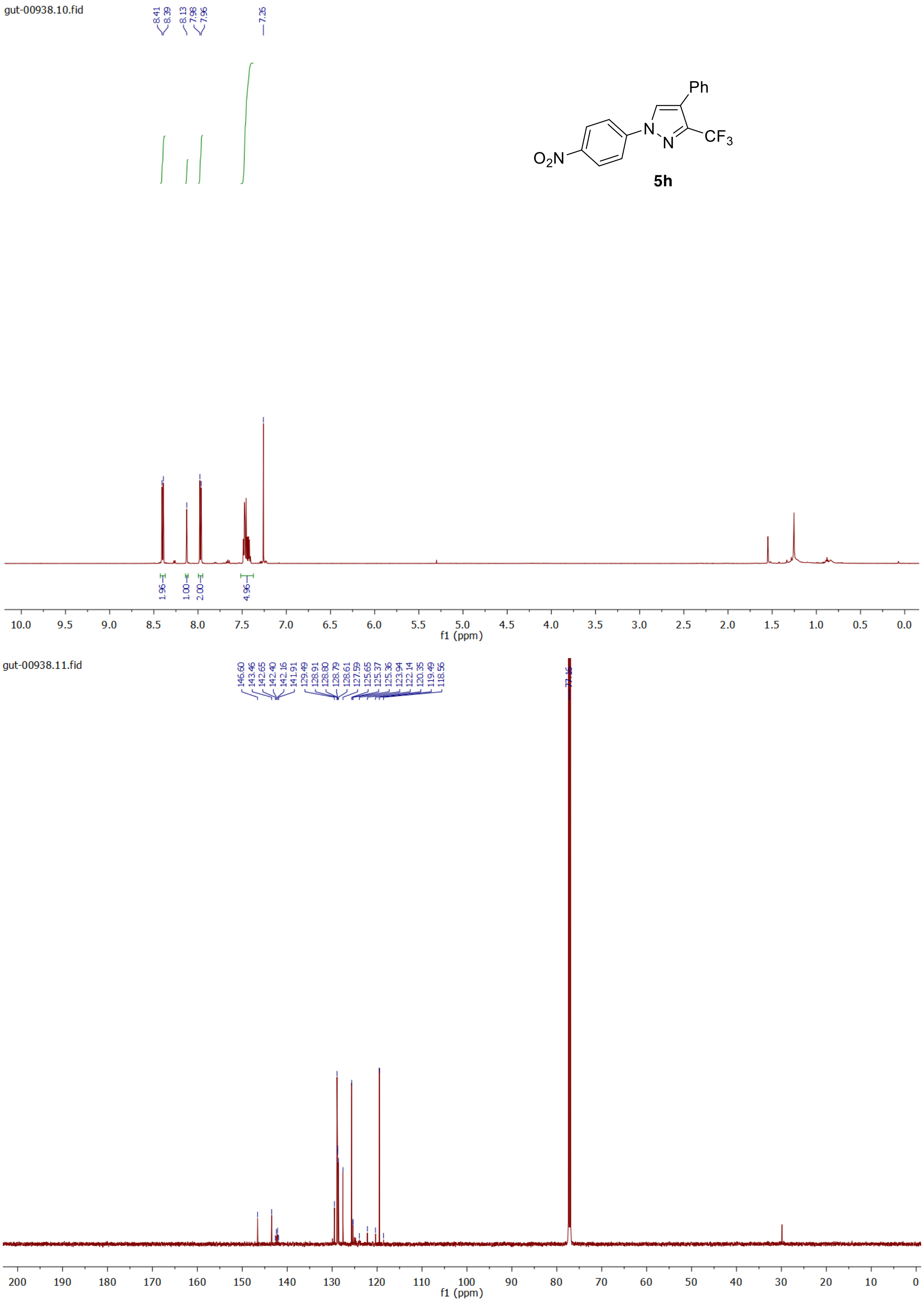

Fig S30. ^1H NMR (600 MHz, $CDCl_3$) and ^{13}C NMR (151 MHz, $CDCl_3$) spectra for compound **5h**.

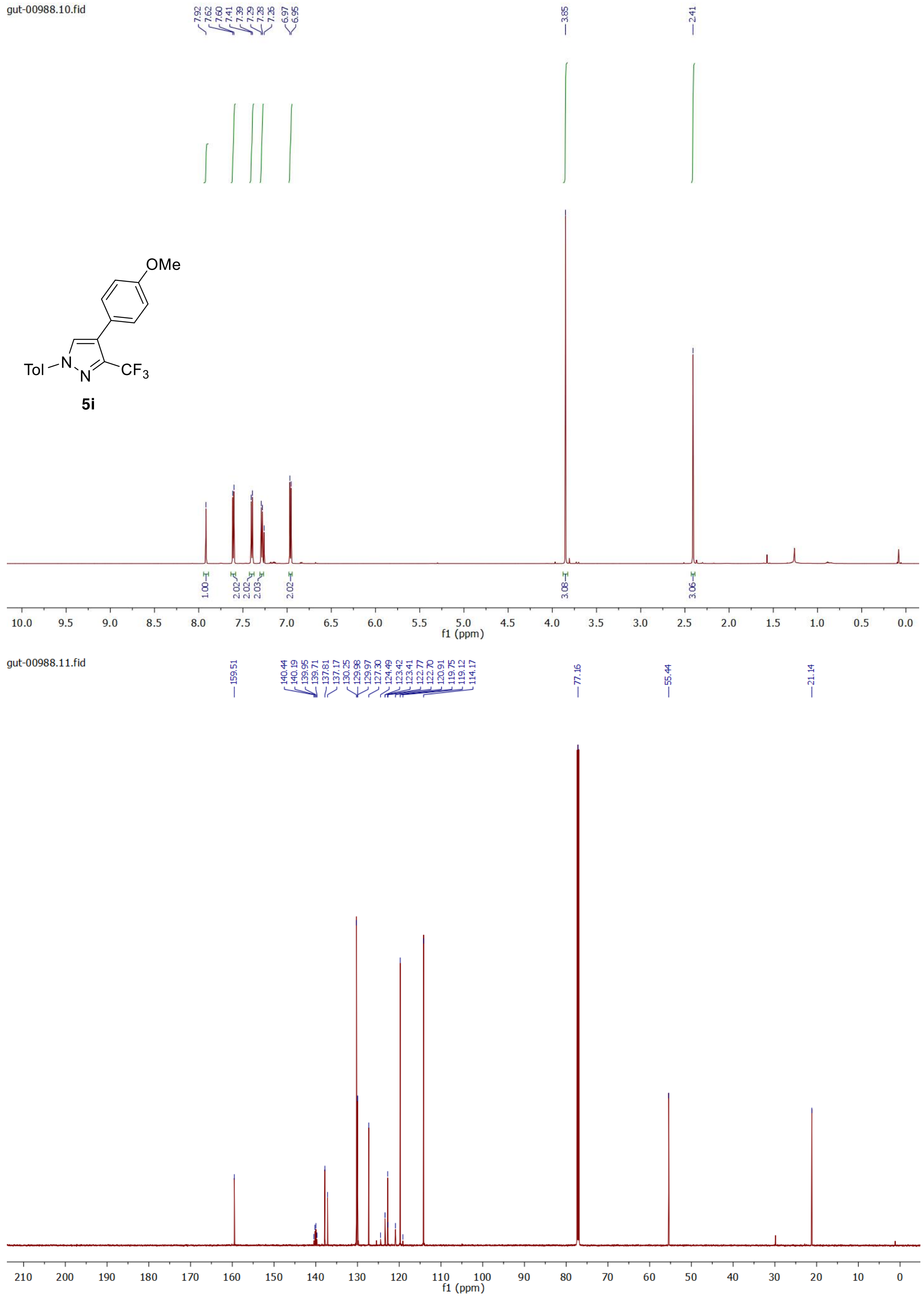

Fig S31. ^{1}H NMR (600 MHz, $CDCl_3$) and ^{13}C NMR (151 MHz, $CDCl_3$) spectra for compound **5i**.

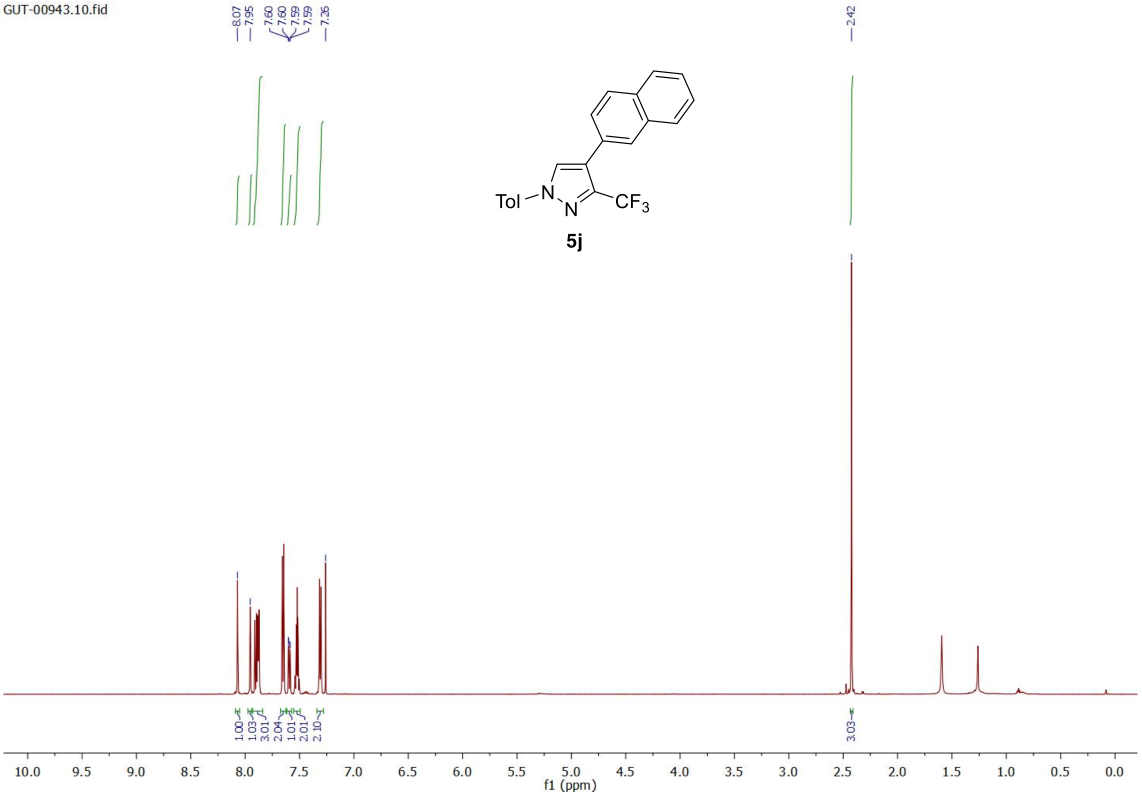

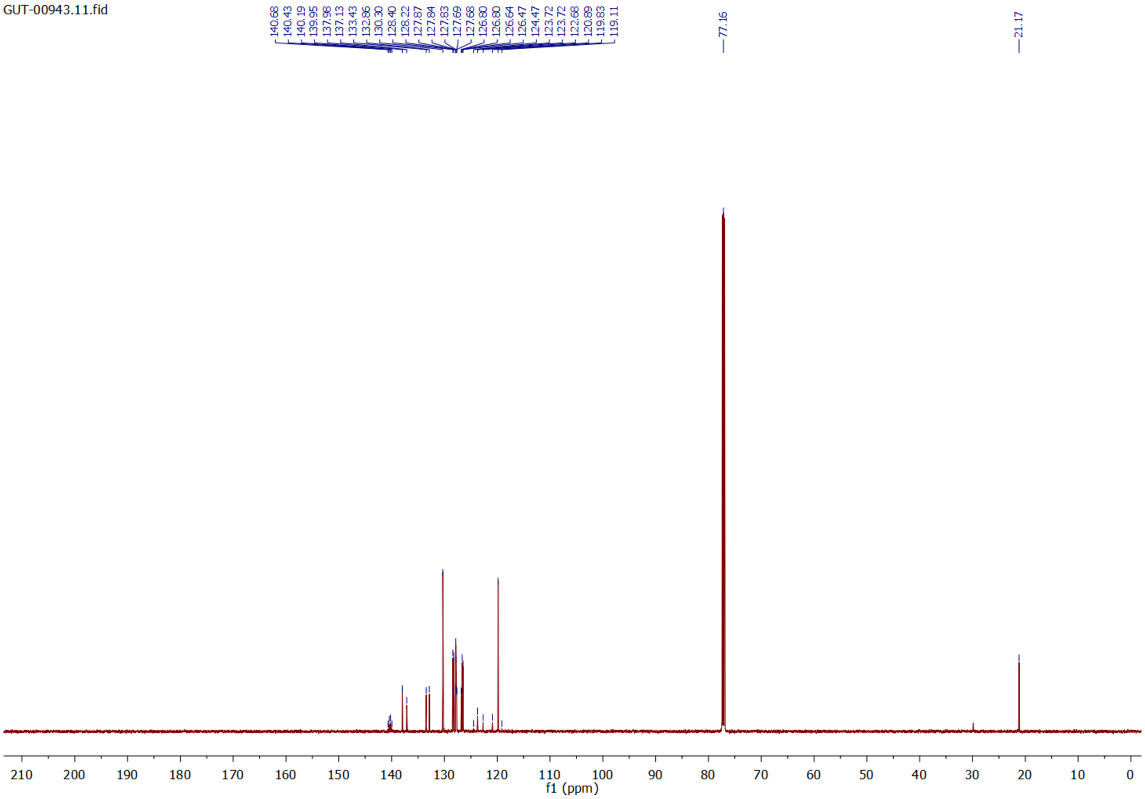

Fig S32. ^{1}H NMR (600 MHz, $CDCl_3$) and ^{13}C NMR (151 MHz, $CDCl_3$) spectra for compound **5j**.

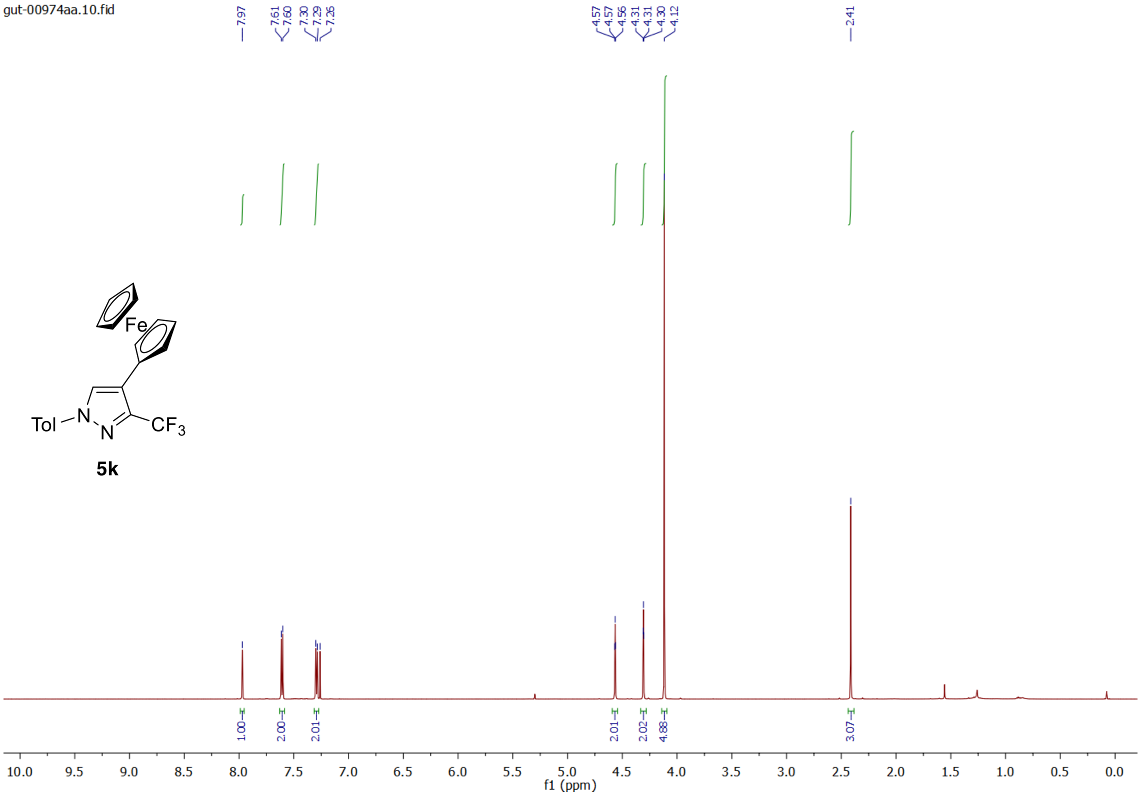

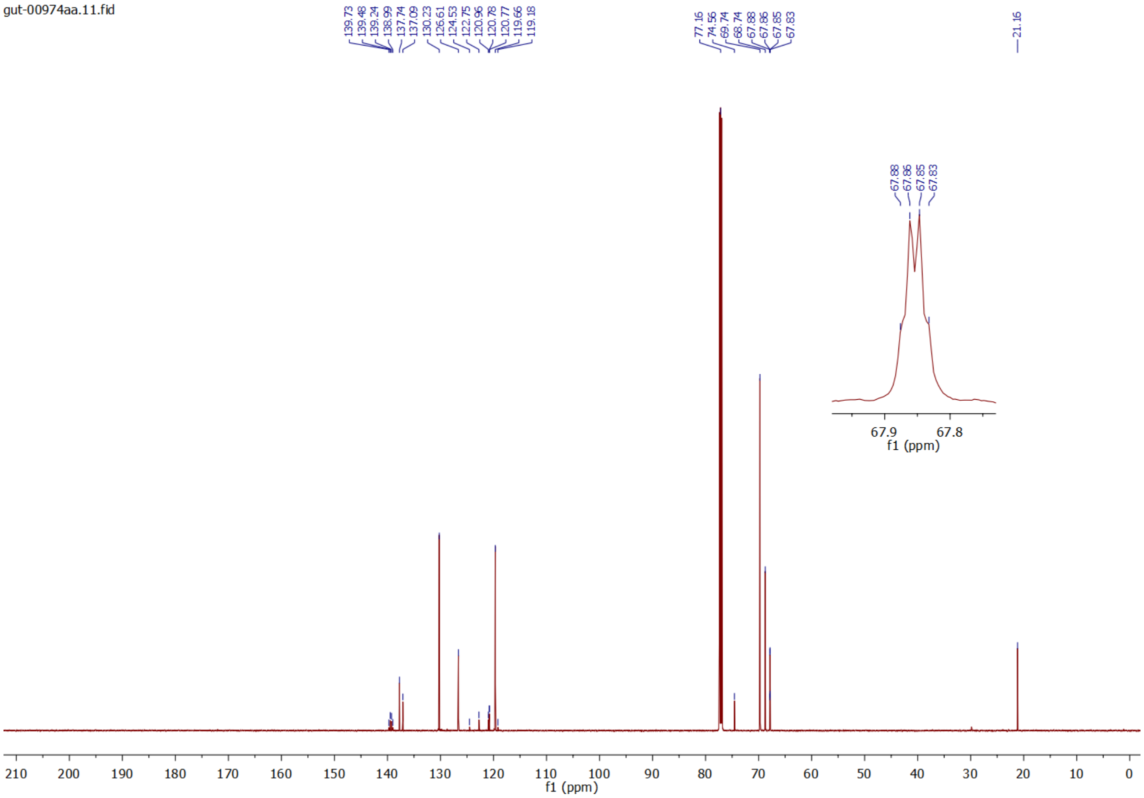

Fig S33. ^{1}H NMR (600 MHz, $CDCl_3$) and ^{13}C NMR (151 MHz, $CDCl_3$) spectra for compound **5k**.

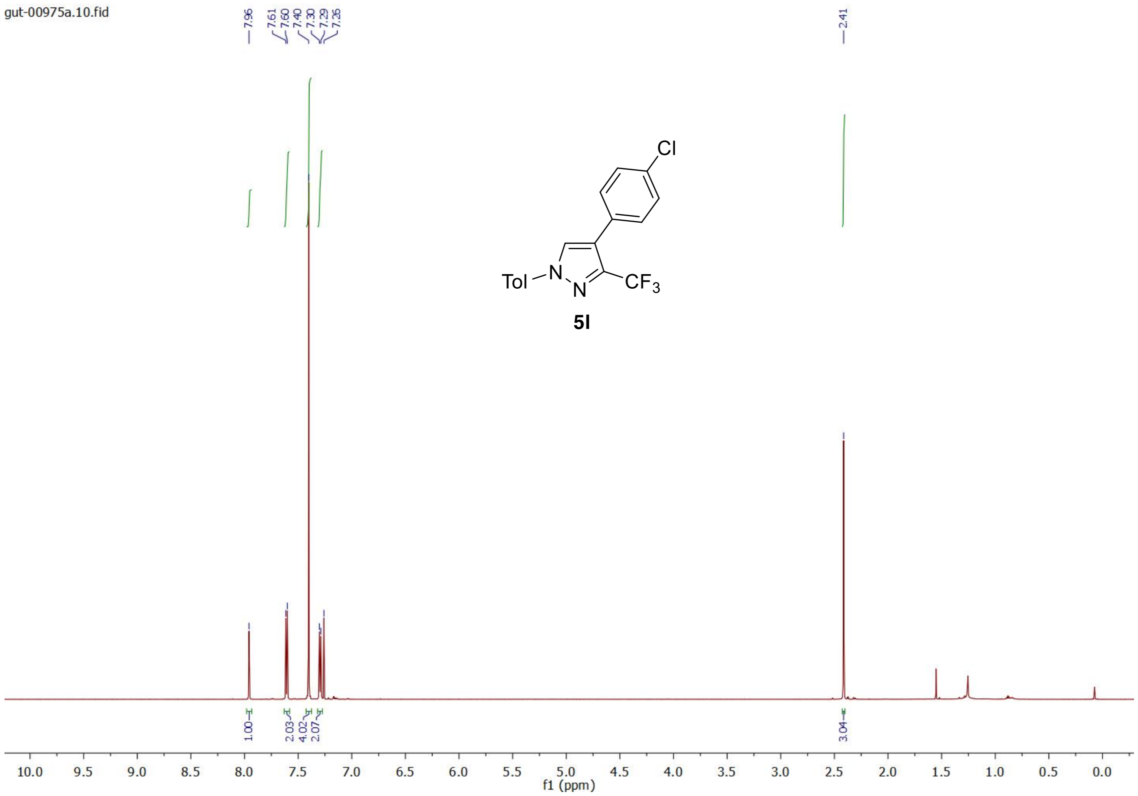

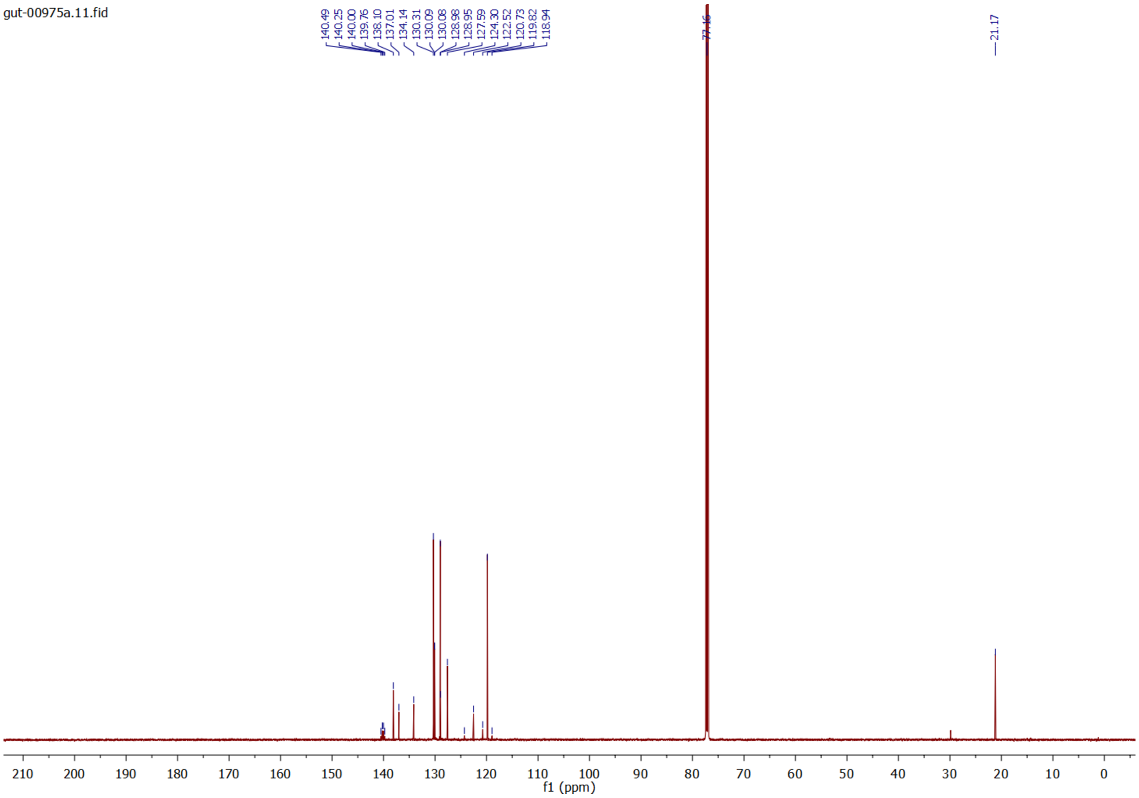

Fig S34. ^{1}H NMR (600 MHz, $CDCl_3$) and ^{13}C NMR (151 MHz, $CDCl_3$) spectra for compound **5l**.

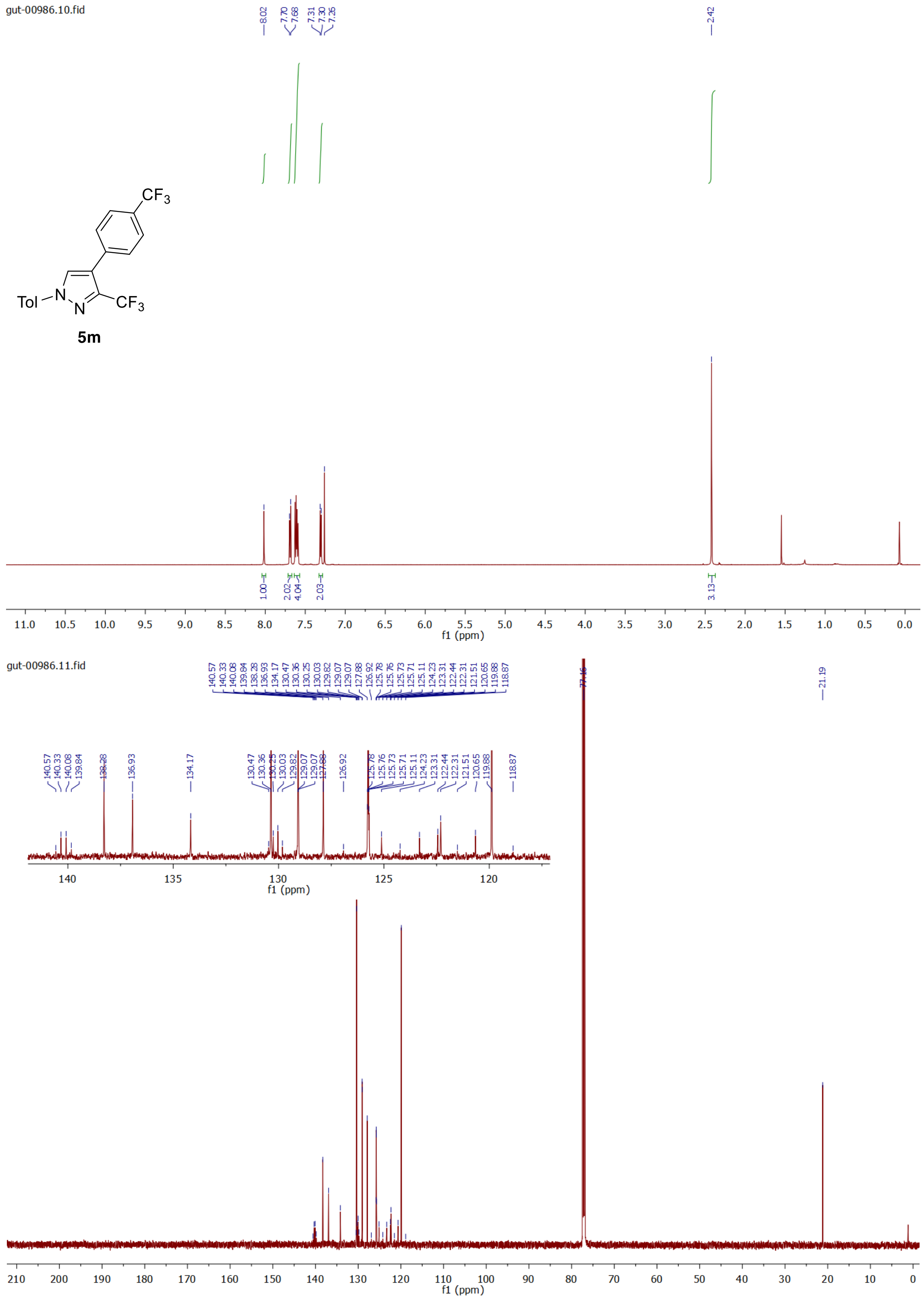

Fig S35. ¹H NMR (600 MHz, CDCl₃) and ¹³C NMR (151 MHz, CDCl₃) spectra for compound **5m**.

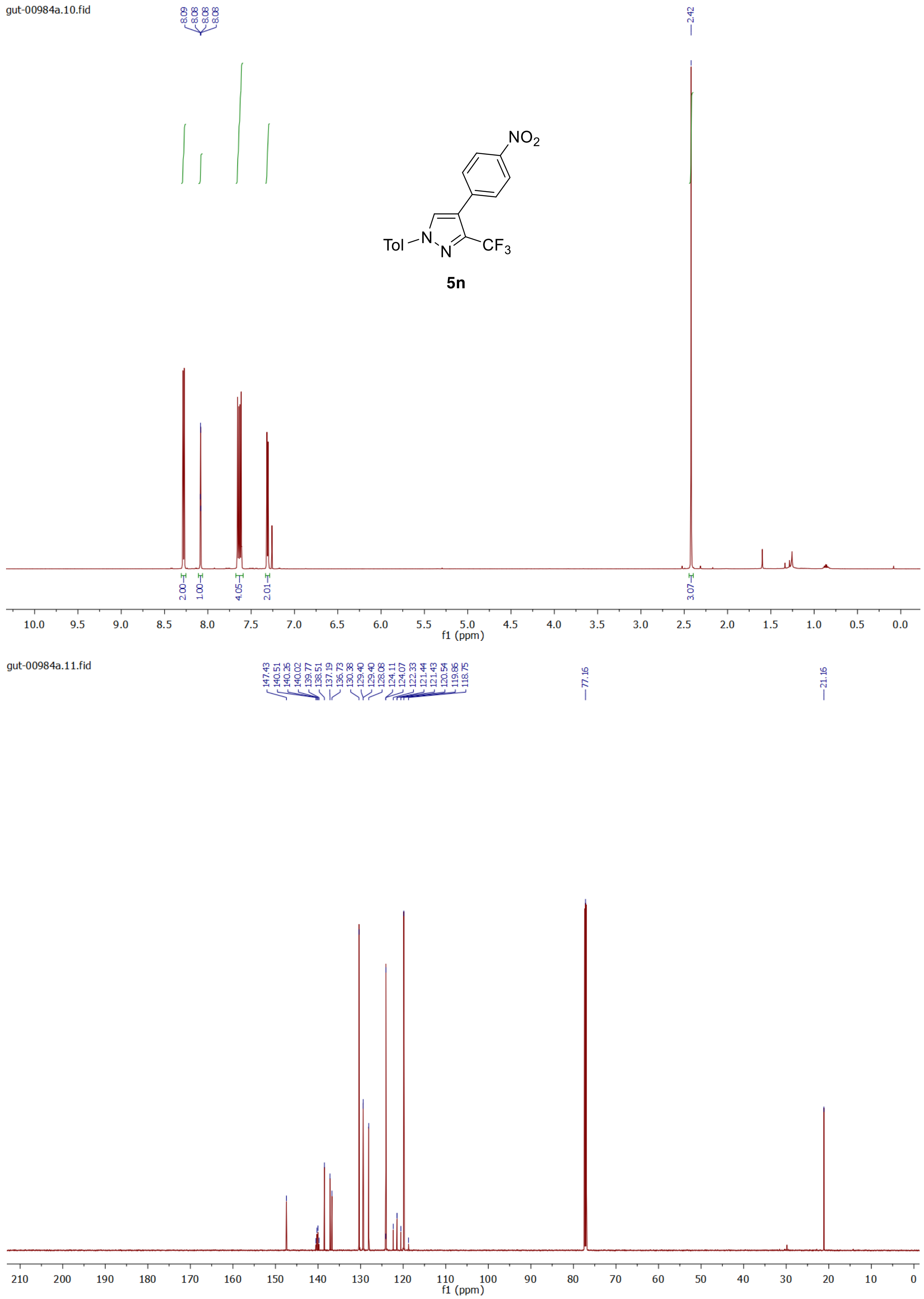

Fig S36. ^{1}H NMR (600 MHz, $CDCl_3$) and ^{13}C NMR (151 MHz, $CDCl_3$) spectra for compound **5n**.

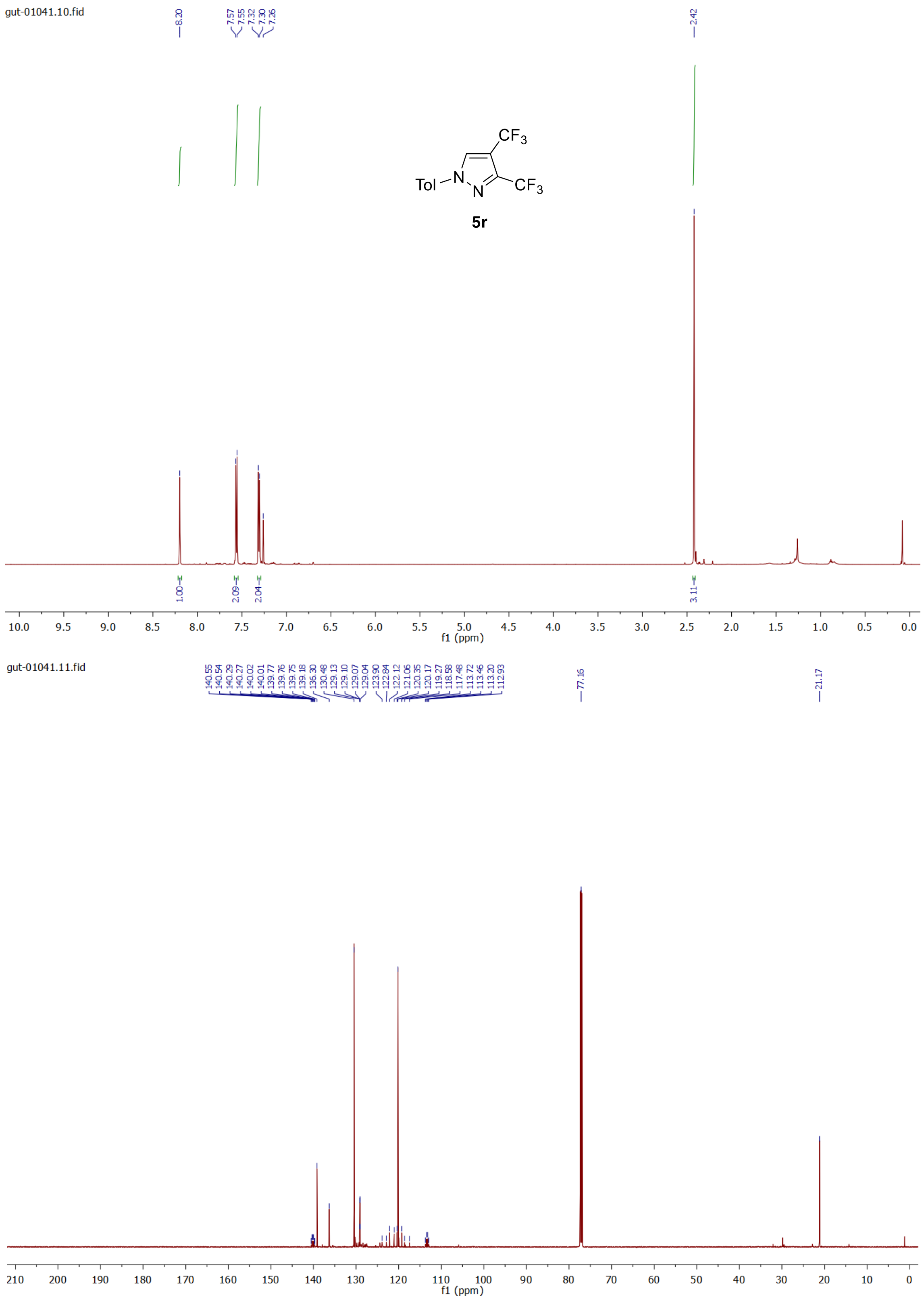

Fig S37. ^{1}H NMR (600 MHz, $CDCl_3$) and ^{13}C NMR (151 MHz, $CDCl_3$) spectra for compound **5r**.

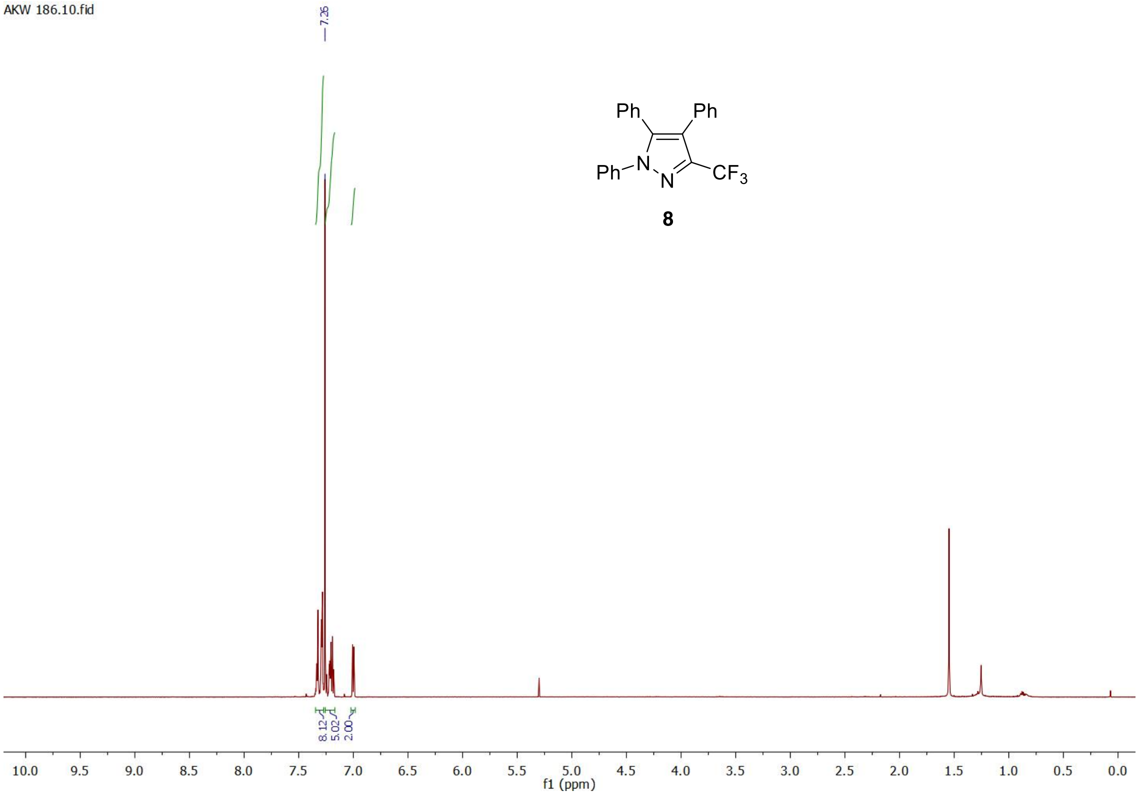

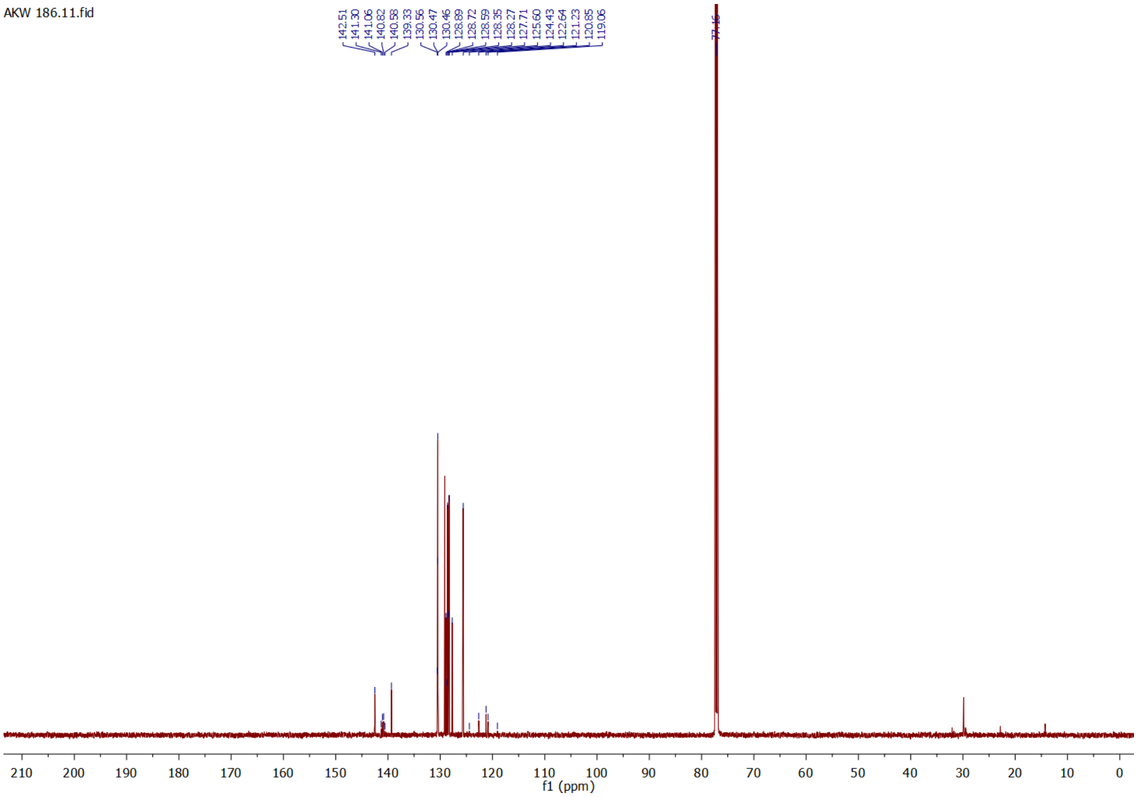

Fig S38. ^{1}H NMR (600 MHz, $CDCl_3$) and ^{13}C NMR (151 MHz, $CDCl_3$) spectra for compound **8**.

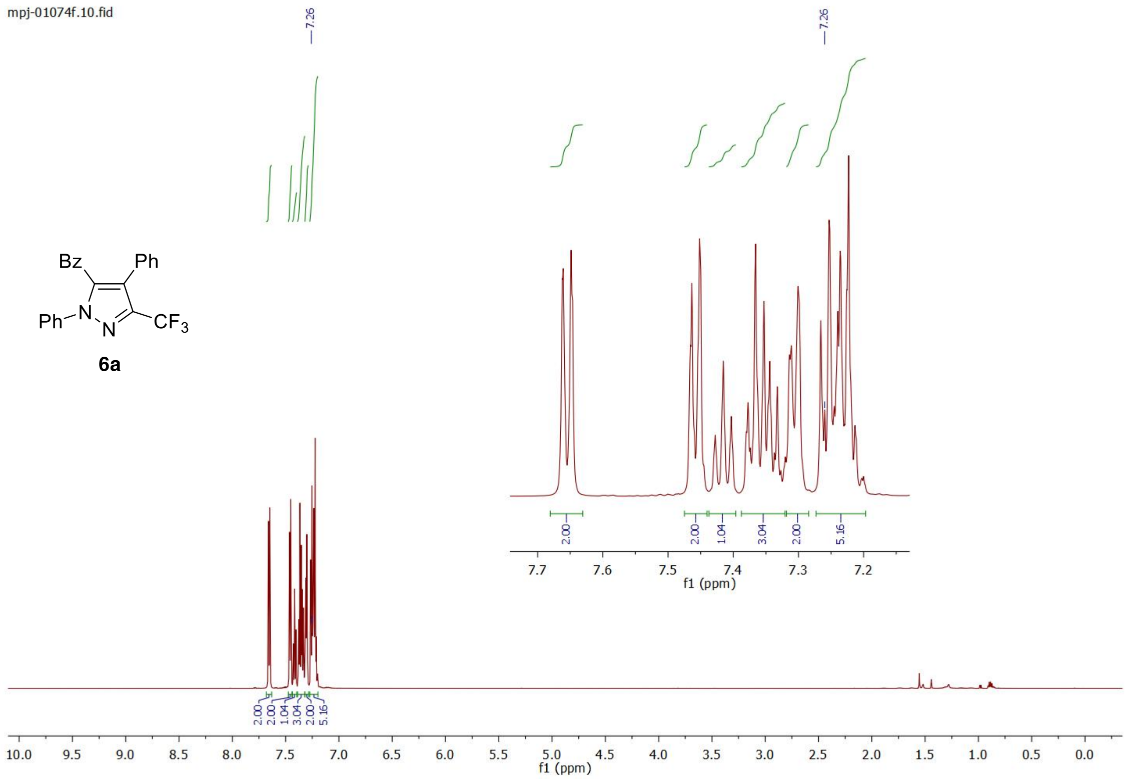

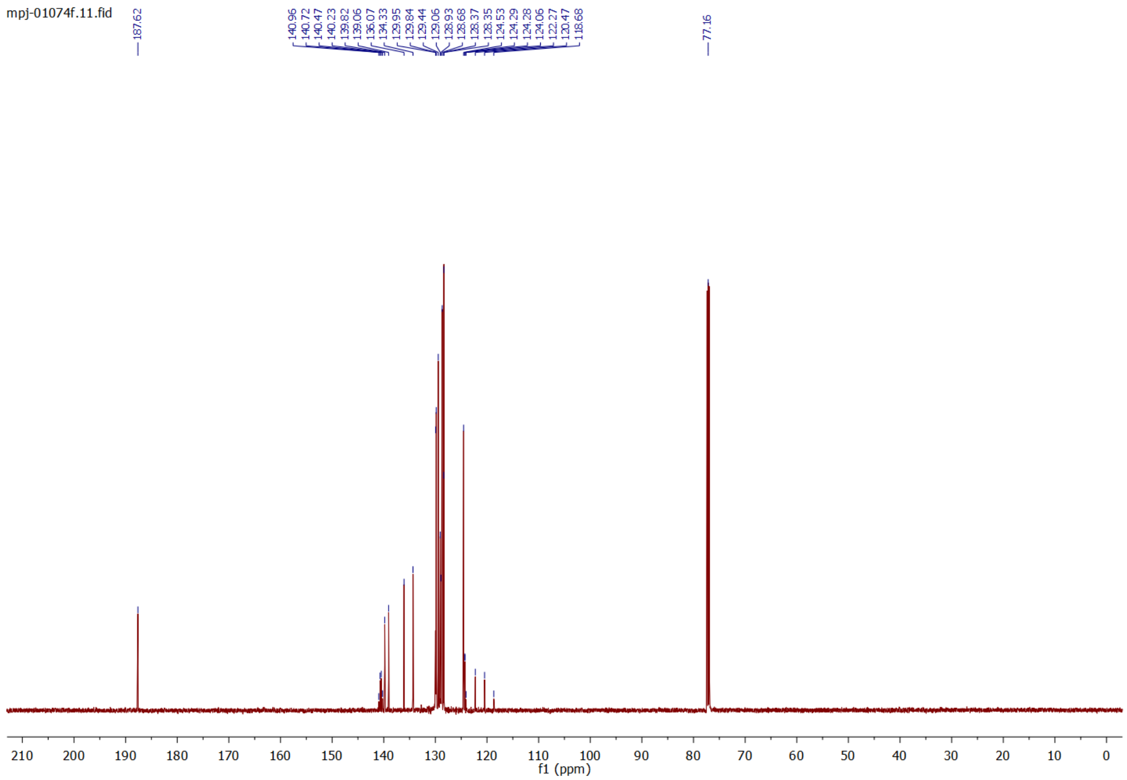

Fig S39. ^{1}H NMR (600 MHz, $CDCl_3$) and ^{13}C NMR (151 MHz, $CDCl_3$) spectra for compound **6a**.

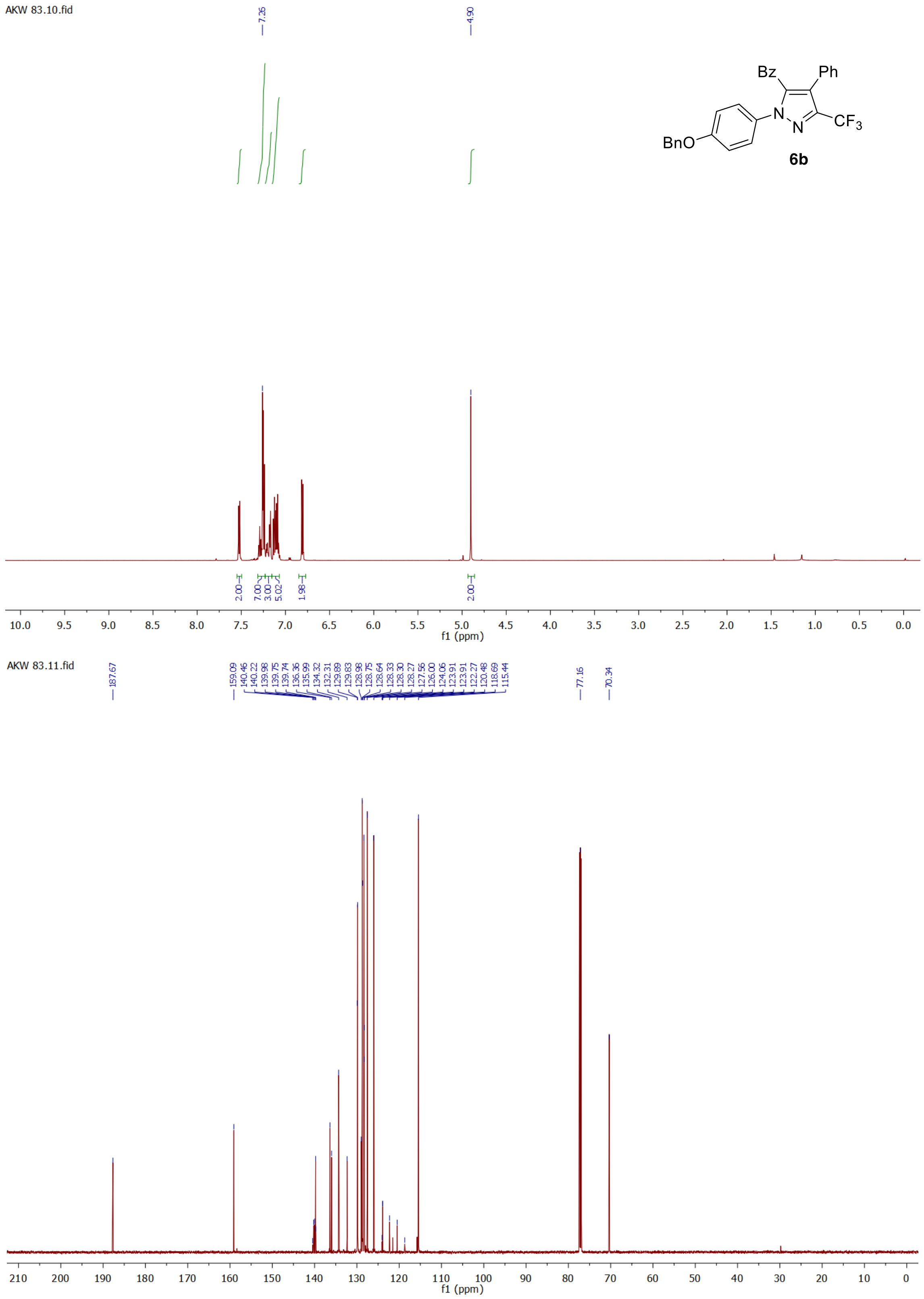

Fig S40. ^{1}H NMR (600 MHz, $CDCl_3$) and ^{13}C NMR (151 MHz, $CDCl_3$) spectra for compound **6b**.

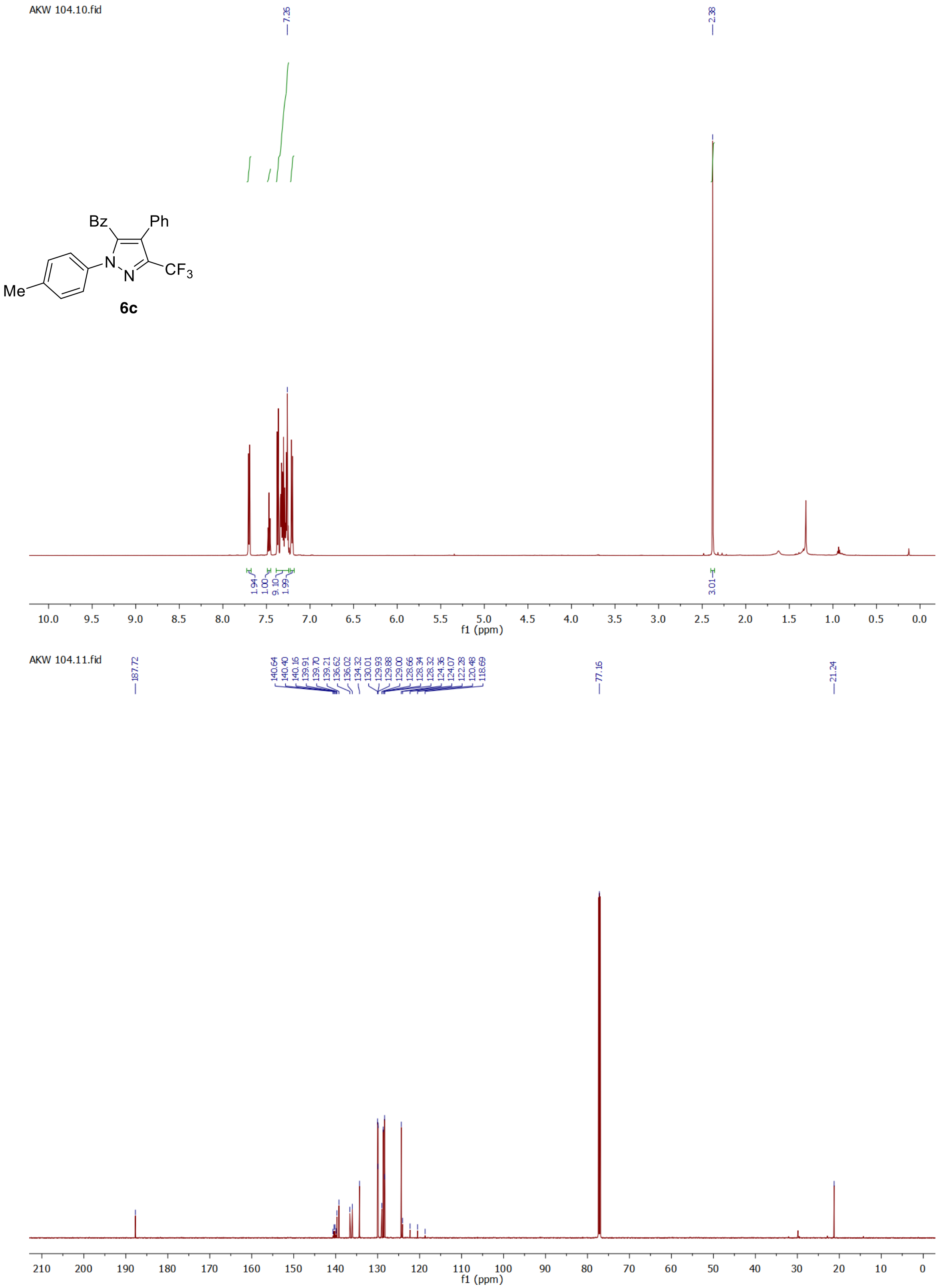

Fig S41. ^{1}H NMR (600 MHz, $CDCl_3$) and ^{13}C NMR (151 MHz, $CDCl_3$) spectra for compound **6c**.

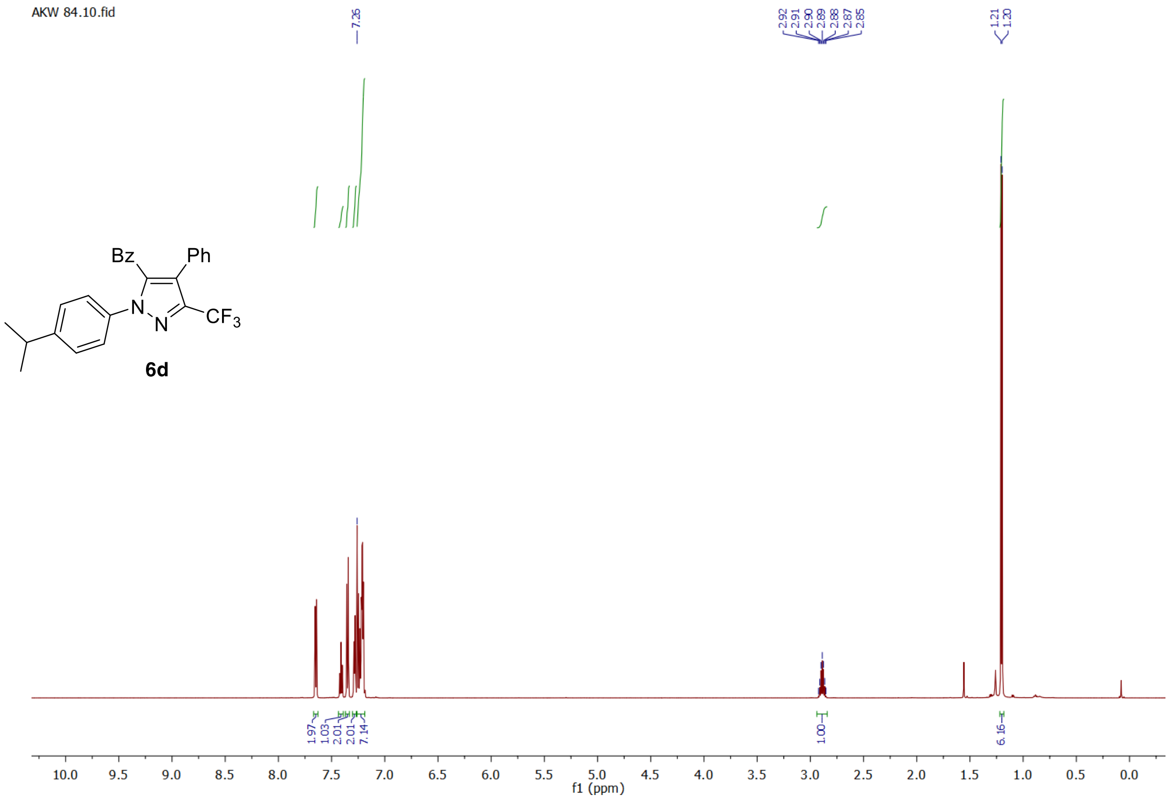

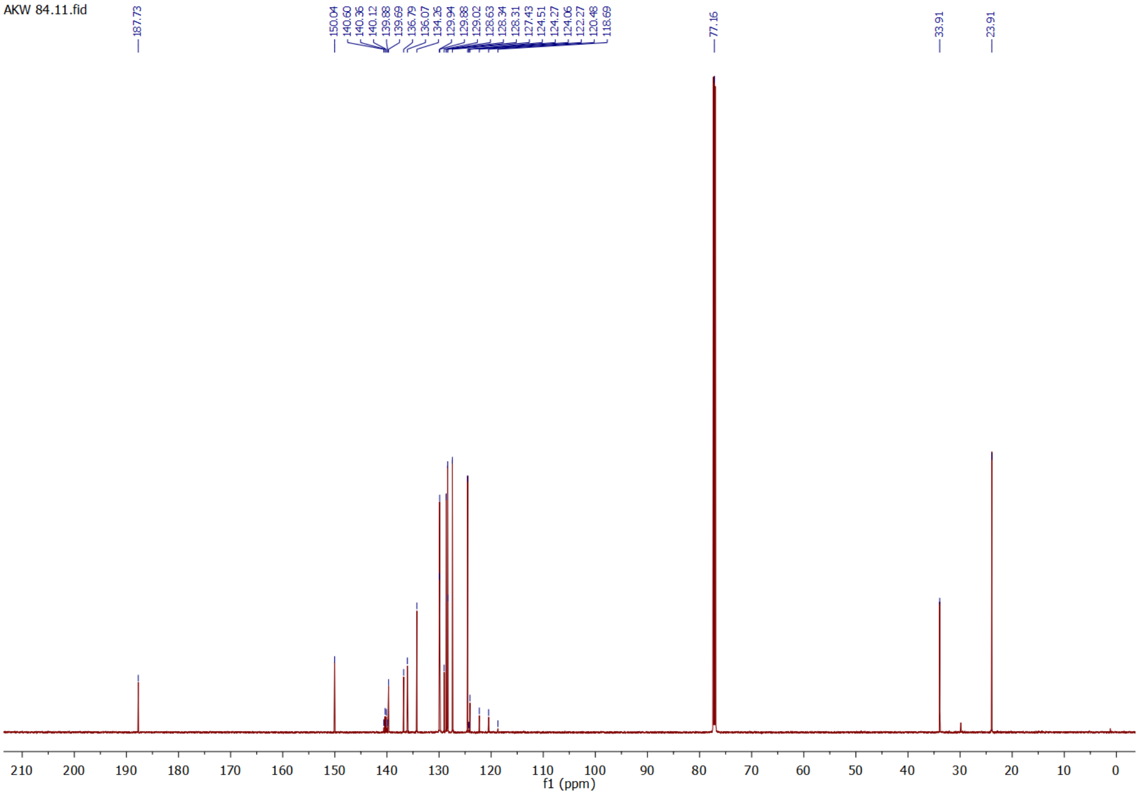

Fig S42. ^{1}H NMR (600 MHz, $CDCl_3$) and ^{13}C NMR (151 MHz, $CDCl_3$) spectra for compound **6d**.

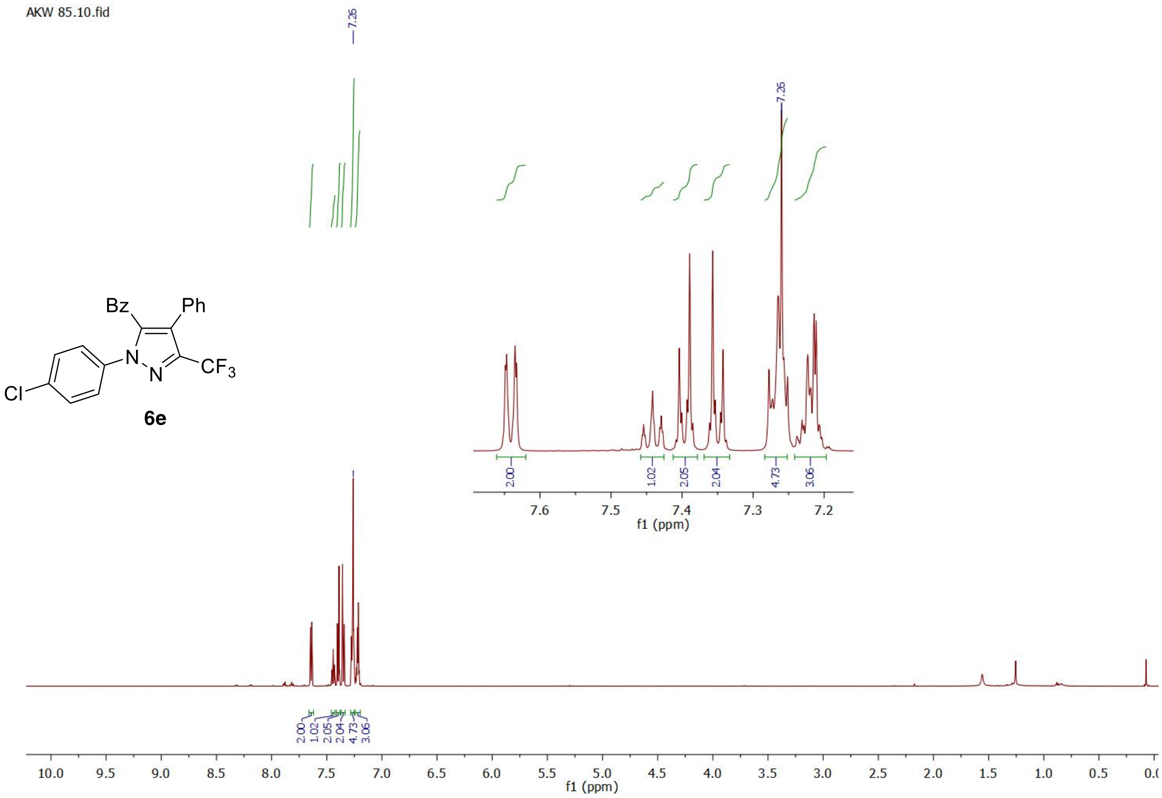

6e

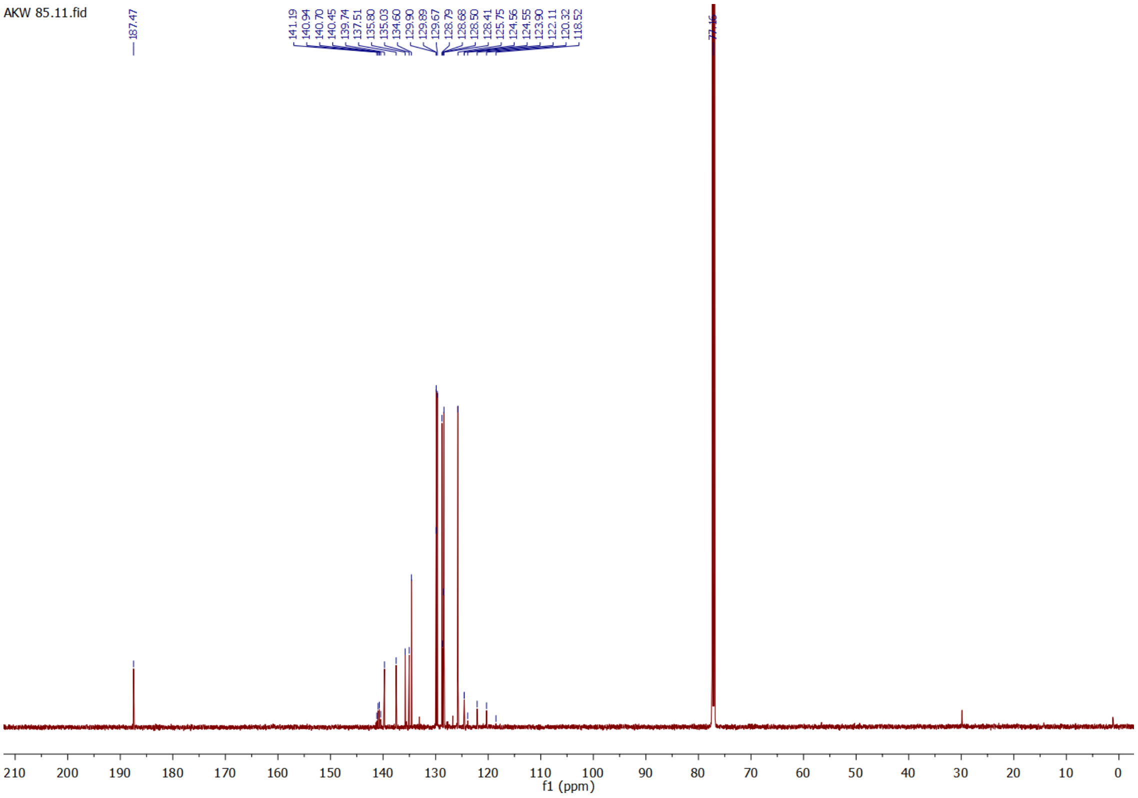

Fig S43. ^{1}H NMR (600 MHz, $CDCl_3$) and ^{13}C NMR (151 MHz, $CDCl_3$) spectra for compound **6e**.

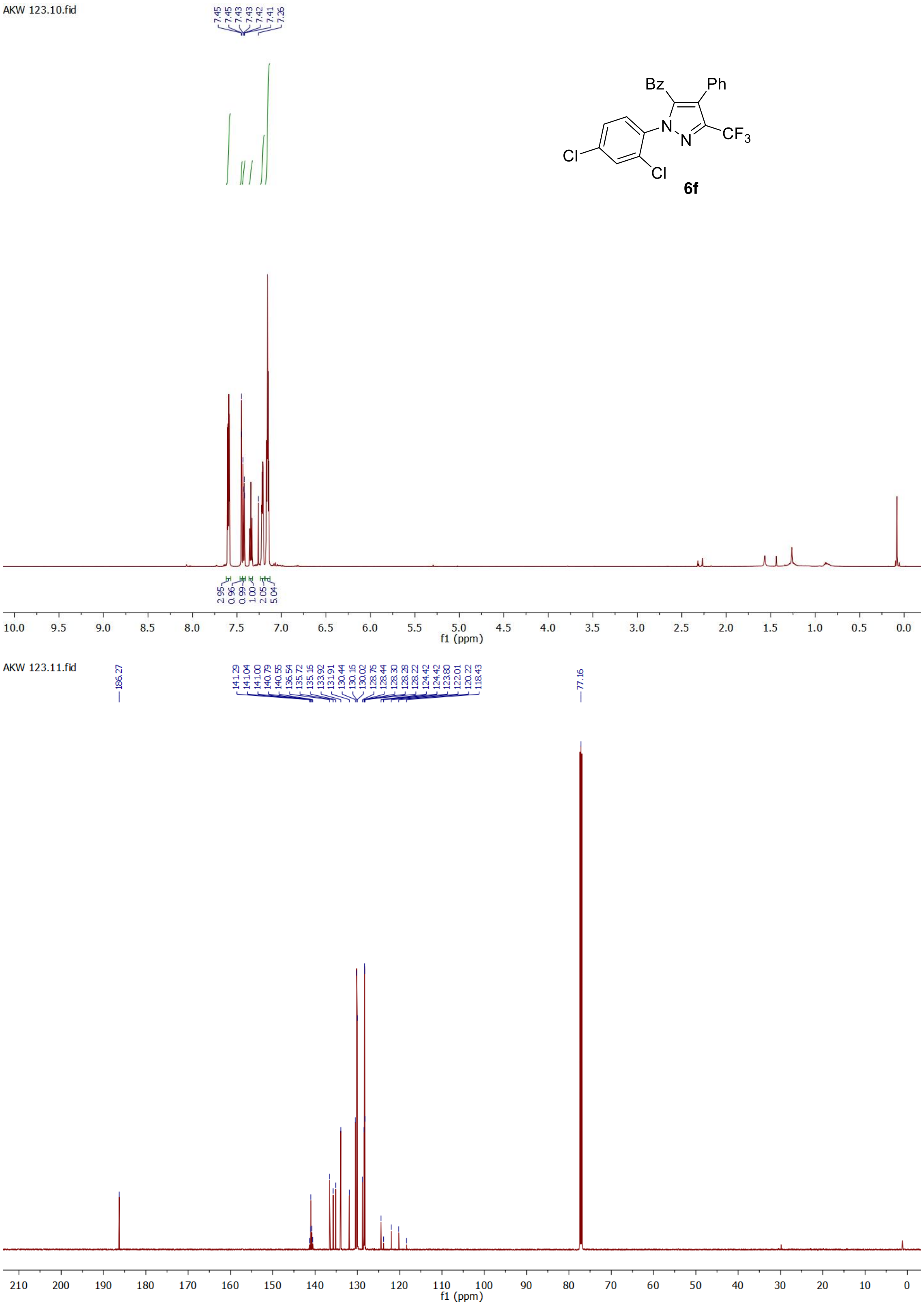

Fig S44. ^{1}H NMR (600 MHz, $CDCl_3$) and ^{13}C NMR (151 MHz, $CDCl_3$) spectra for compound **6f**.

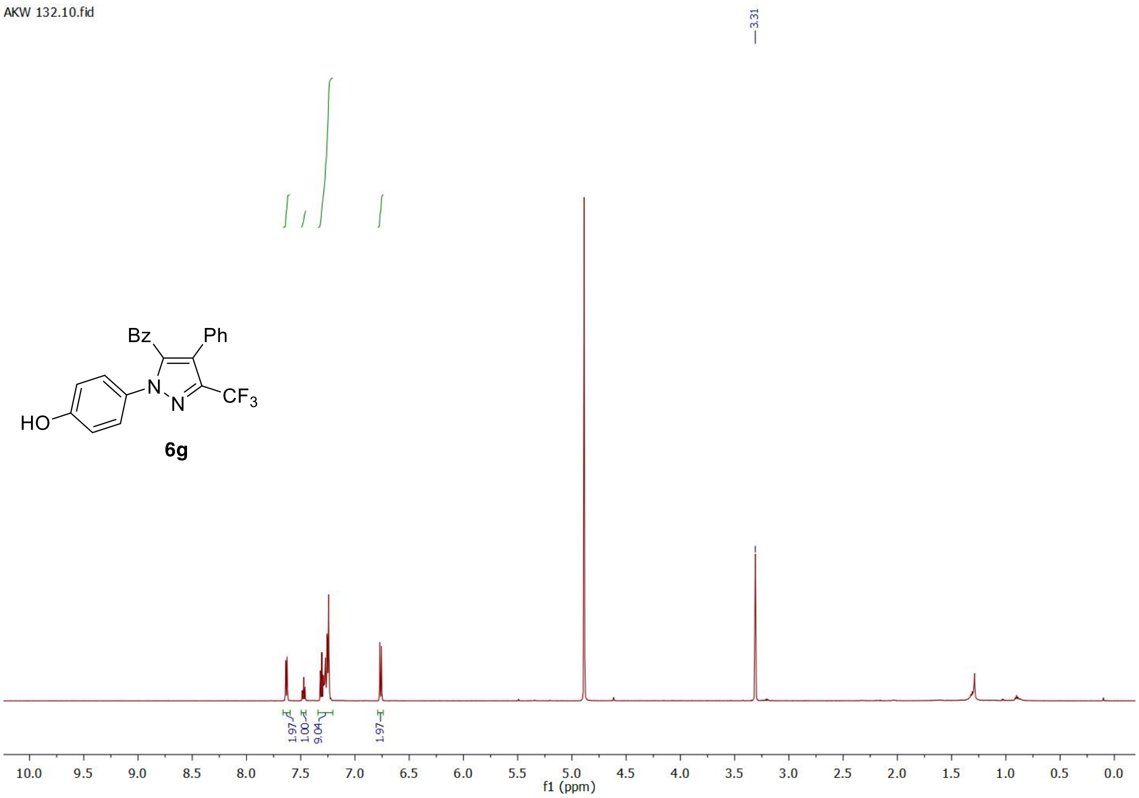

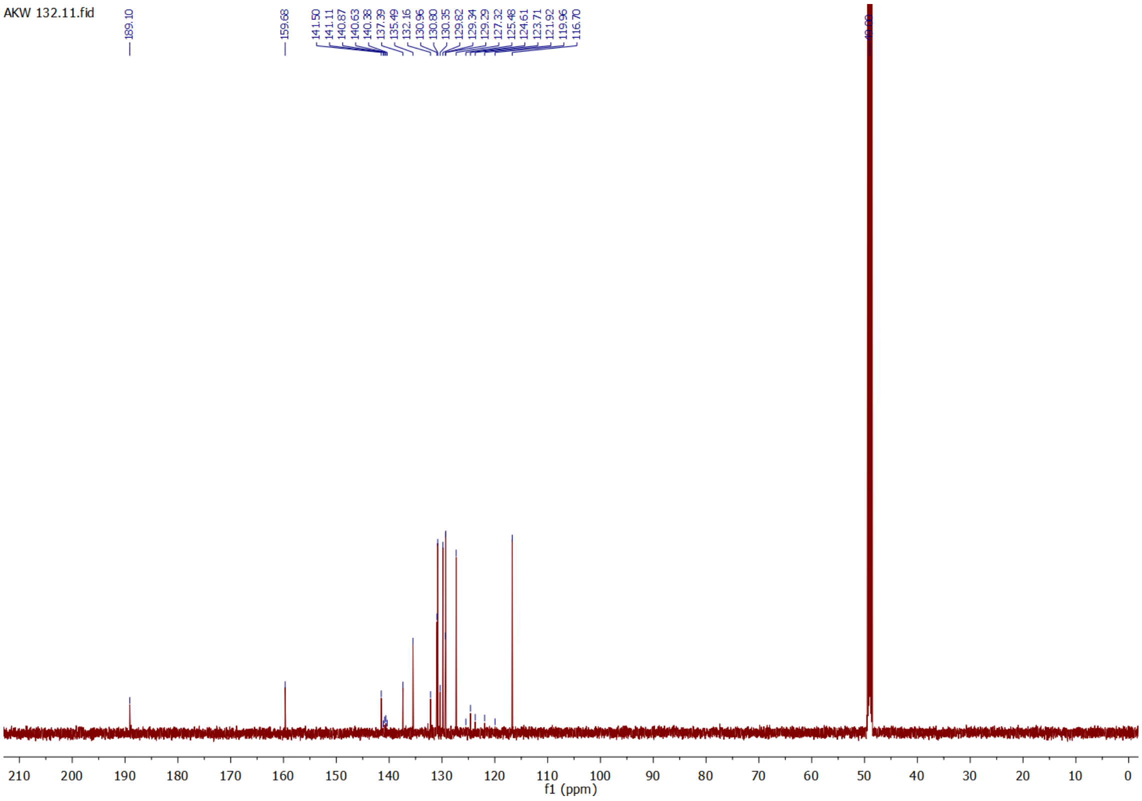

Fig S45. ^{1}H NMR (600 MHz, CD_3OD) and ^{13}C NMR (151 MHz, CD_3OD) spectra for compound **6g**.

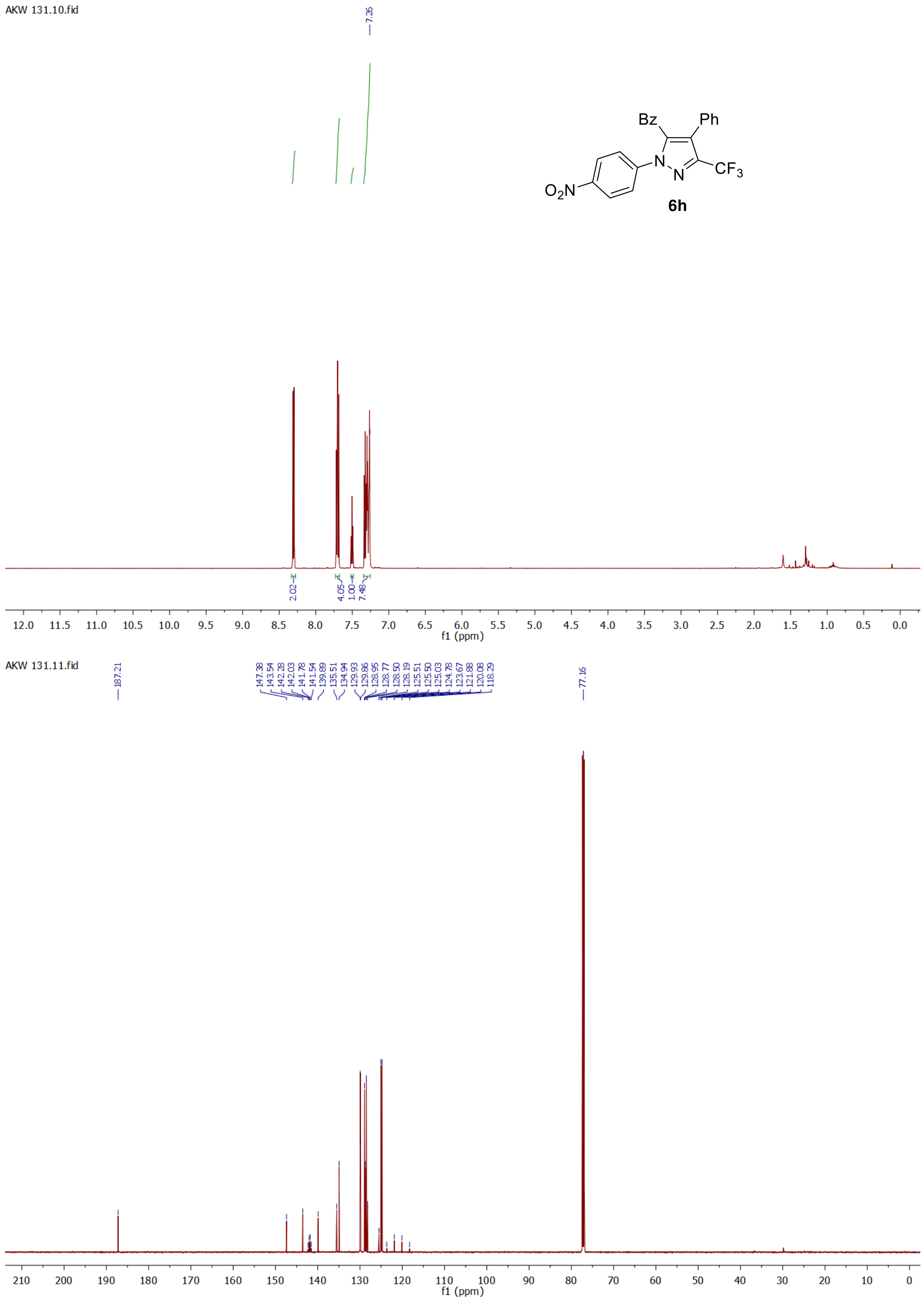

Fig S46. ^{1}H NMR (600 MHz, $CDCl_3$) and ^{13}C NMR (151 MHz, $CDCl_3$) spectra for compound **6h**.

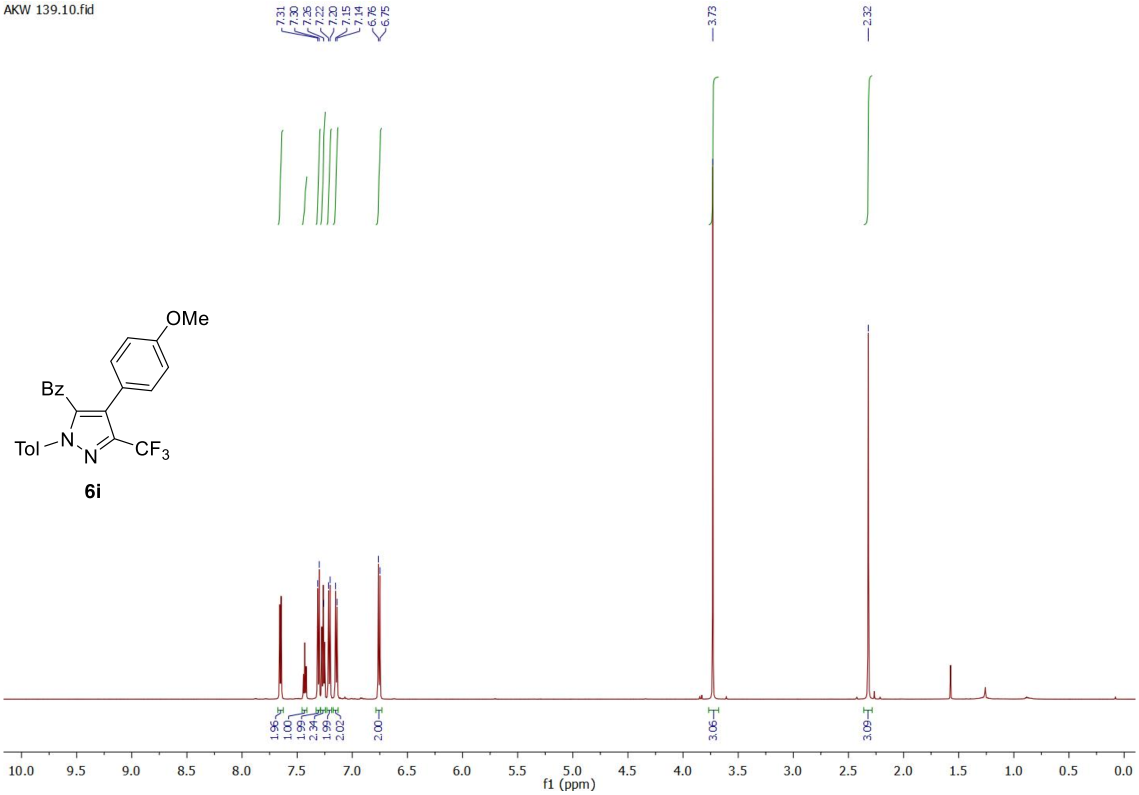

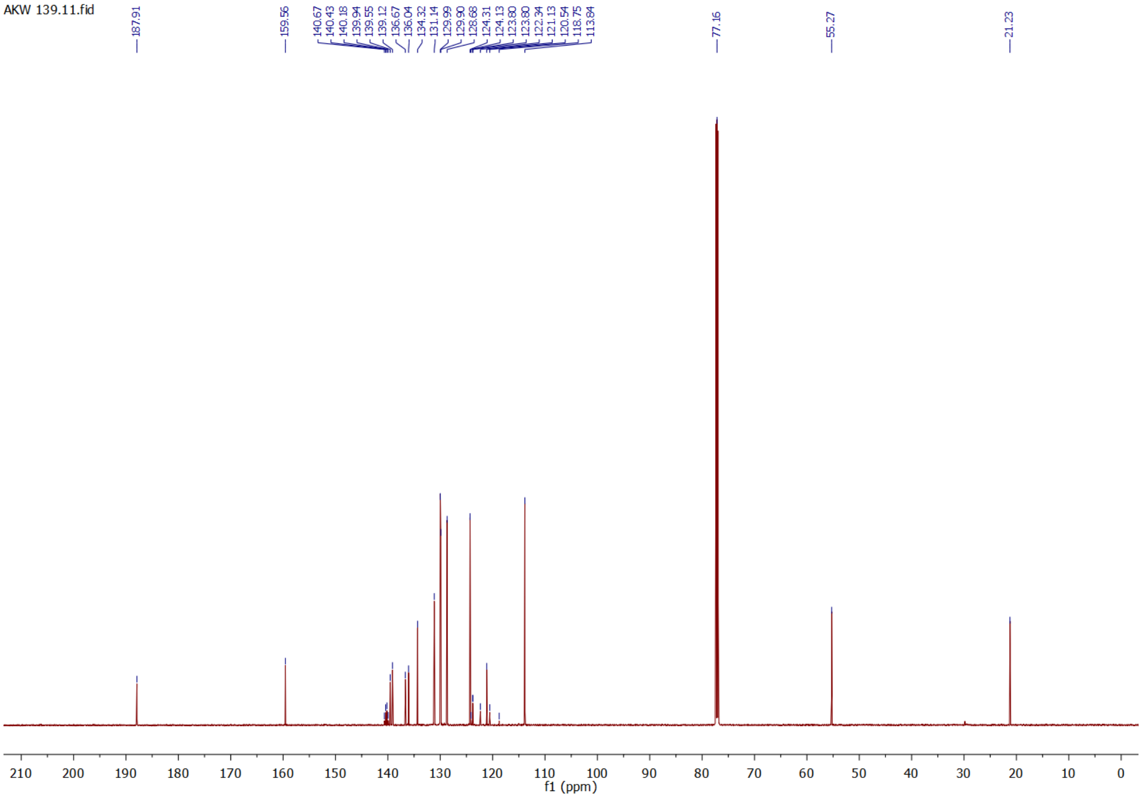

Fig S47. ^{1}H NMR (600 MHz, $CDCl_3$) and ^{13}C NMR (151 MHz, $CDCl_3$) spectra for compound **6i**.

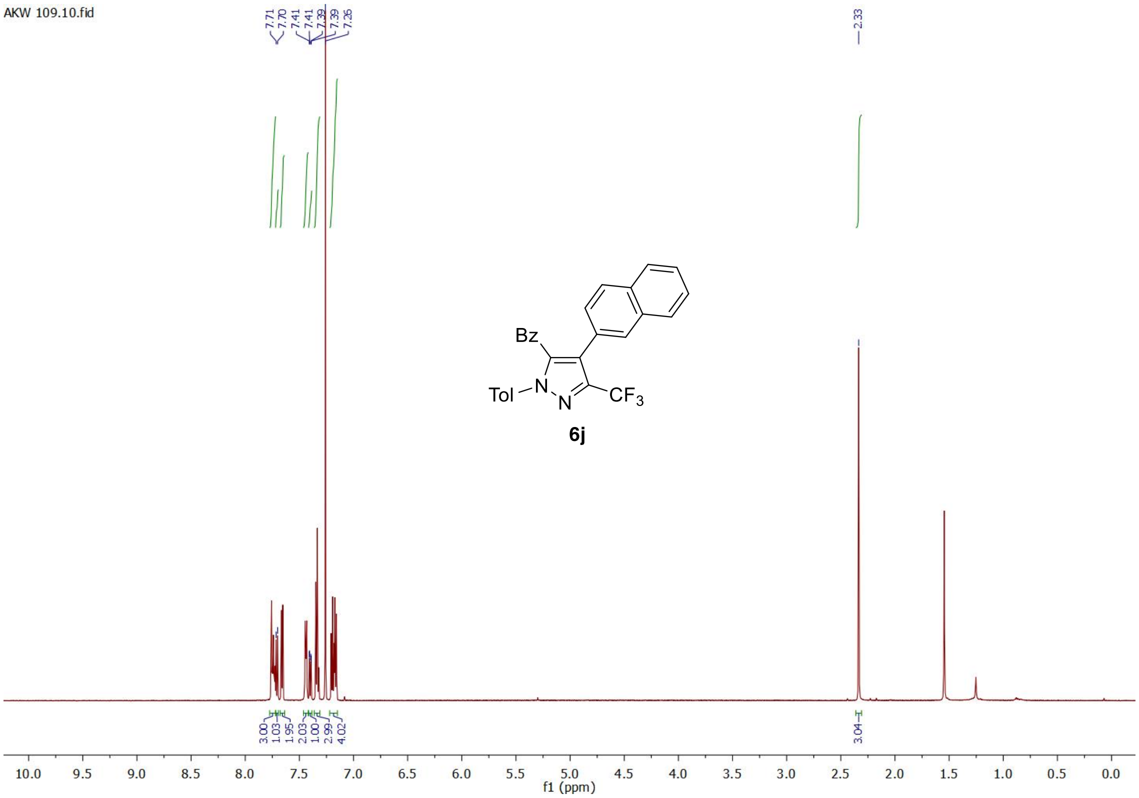

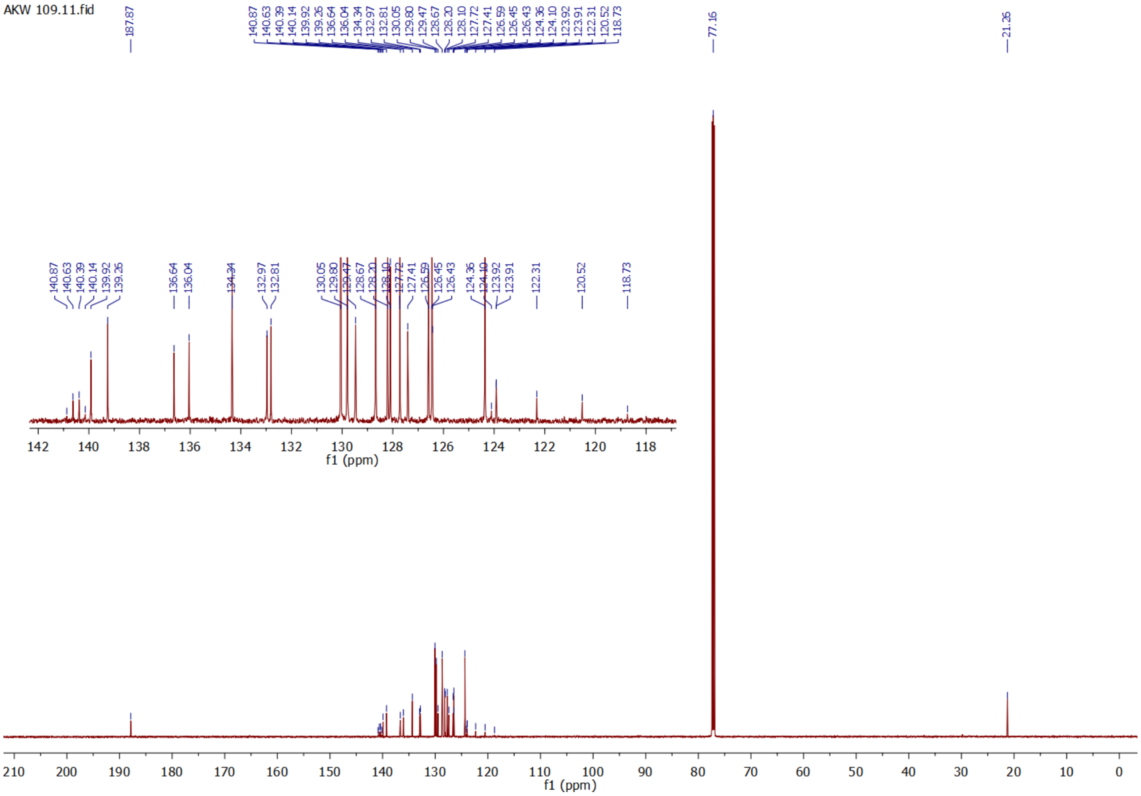

Fig S48. ^{1}H NMR (600 MHz, $CDCl_3$) and ^{13}C NMR (151 MHz, $CDCl_3$) spectra for compound **6j**.

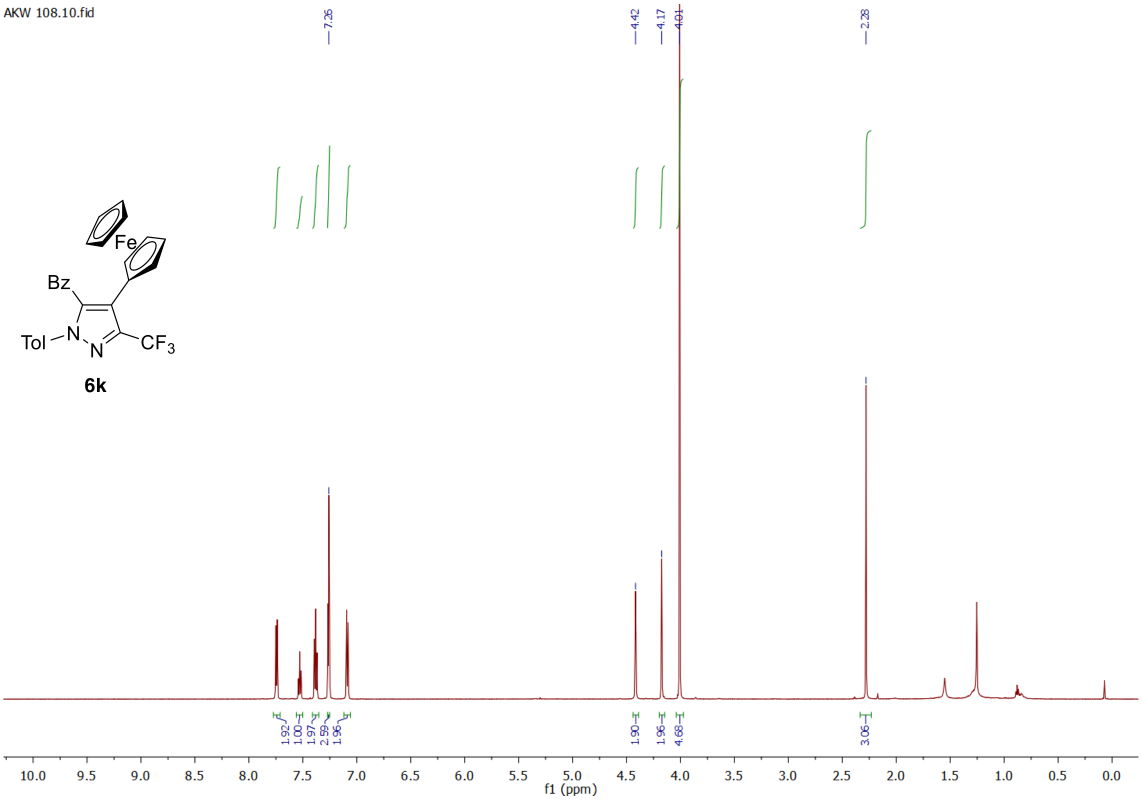

6k

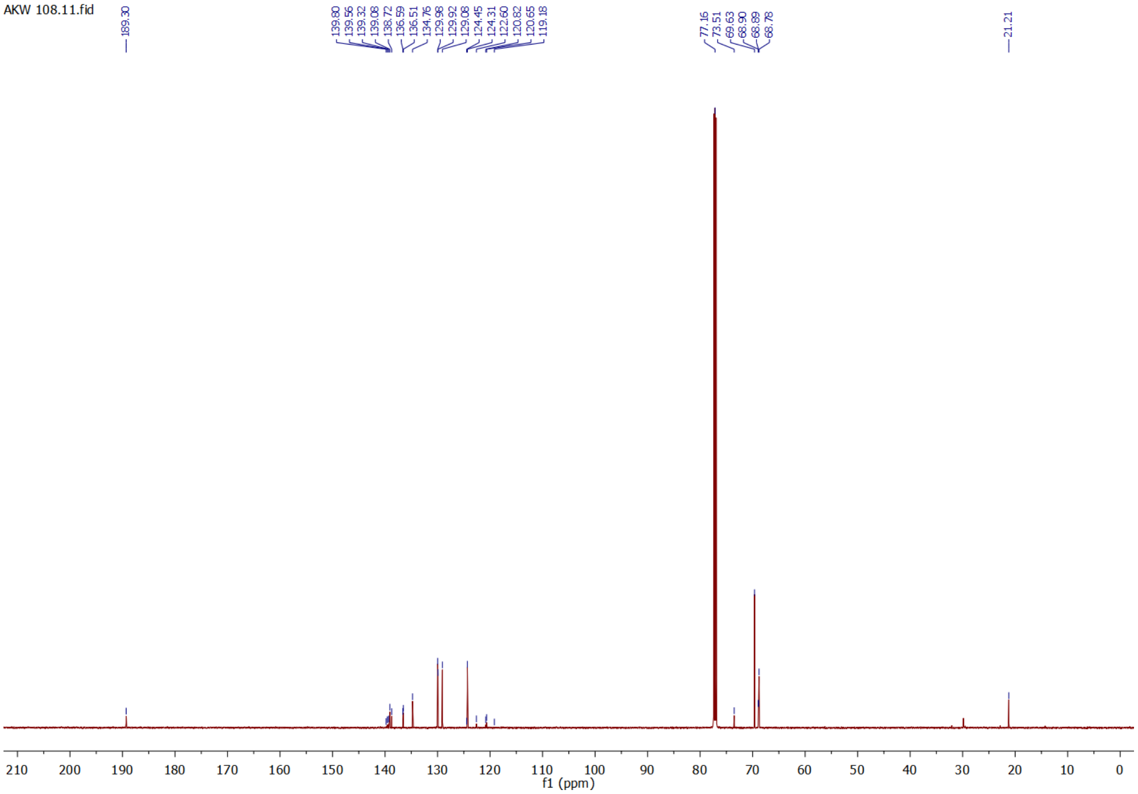

Fig S49. ^{1}H NMR (600 MHz, CDCl$_3$) and ^{13}C NMR (151 MHz, CDCl$_3$) spectra for compound **6k**.

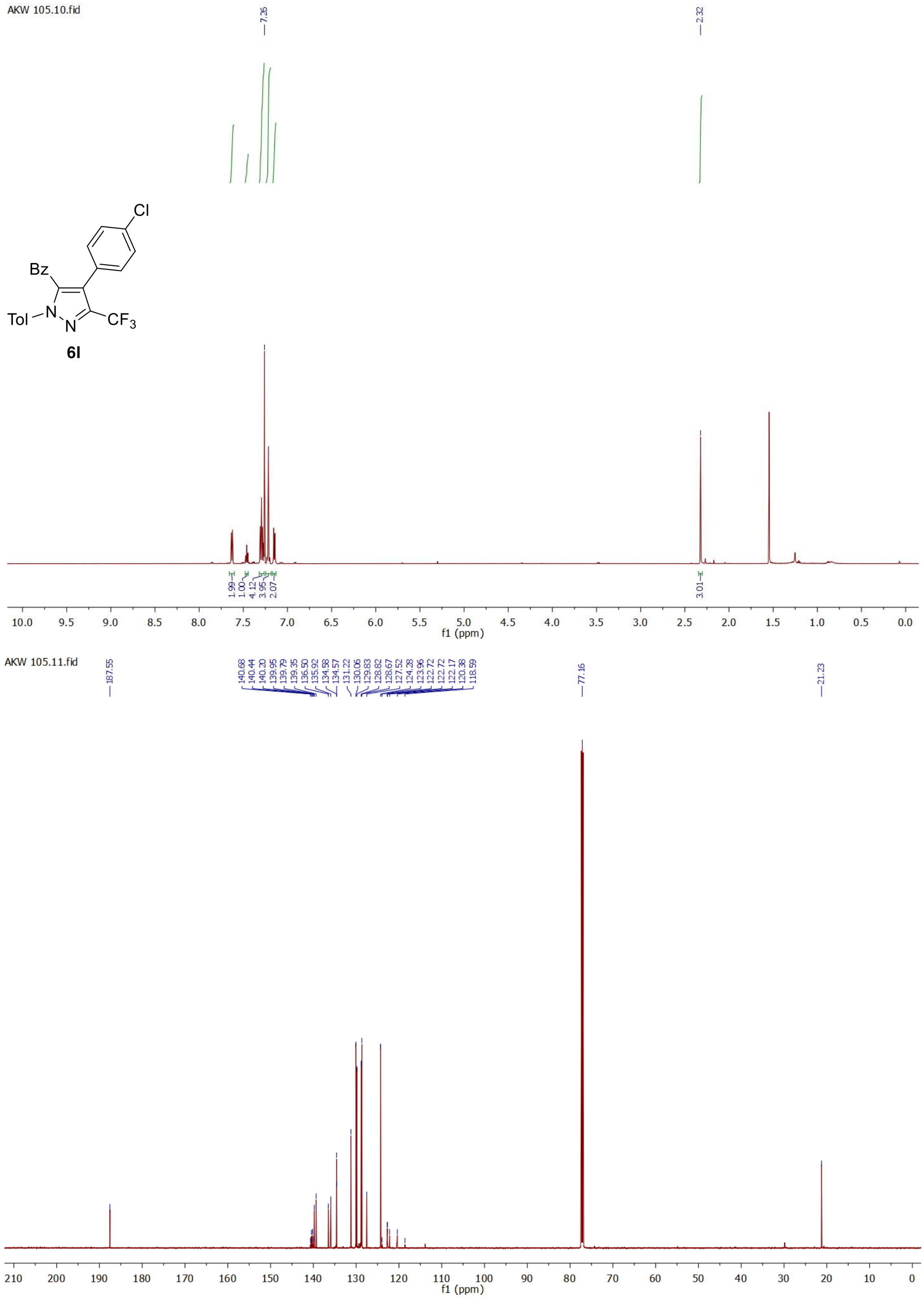

Fig S50. ^{1}H NMR (600 MHz, $CDCl_3$) and ^{13}C NMR (151 MHz, $CDCl_3$) spectra for compound **6l**.

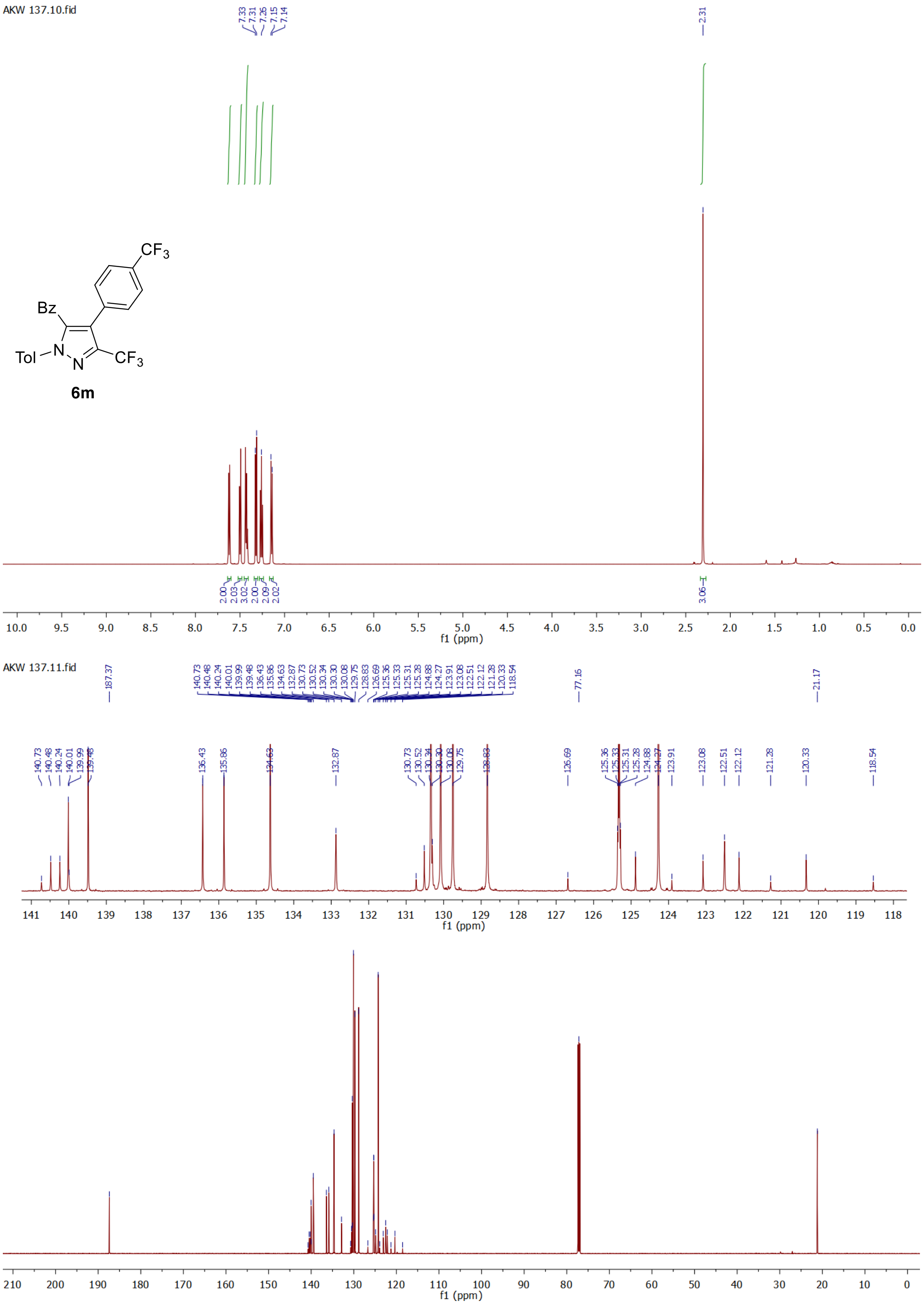

Fig S51. ^{1}H NMR (600 MHz, $CDCl_3$) and ^{13}C NMR (151 MHz, $CDCl_3$) spectra for compound **6m**.

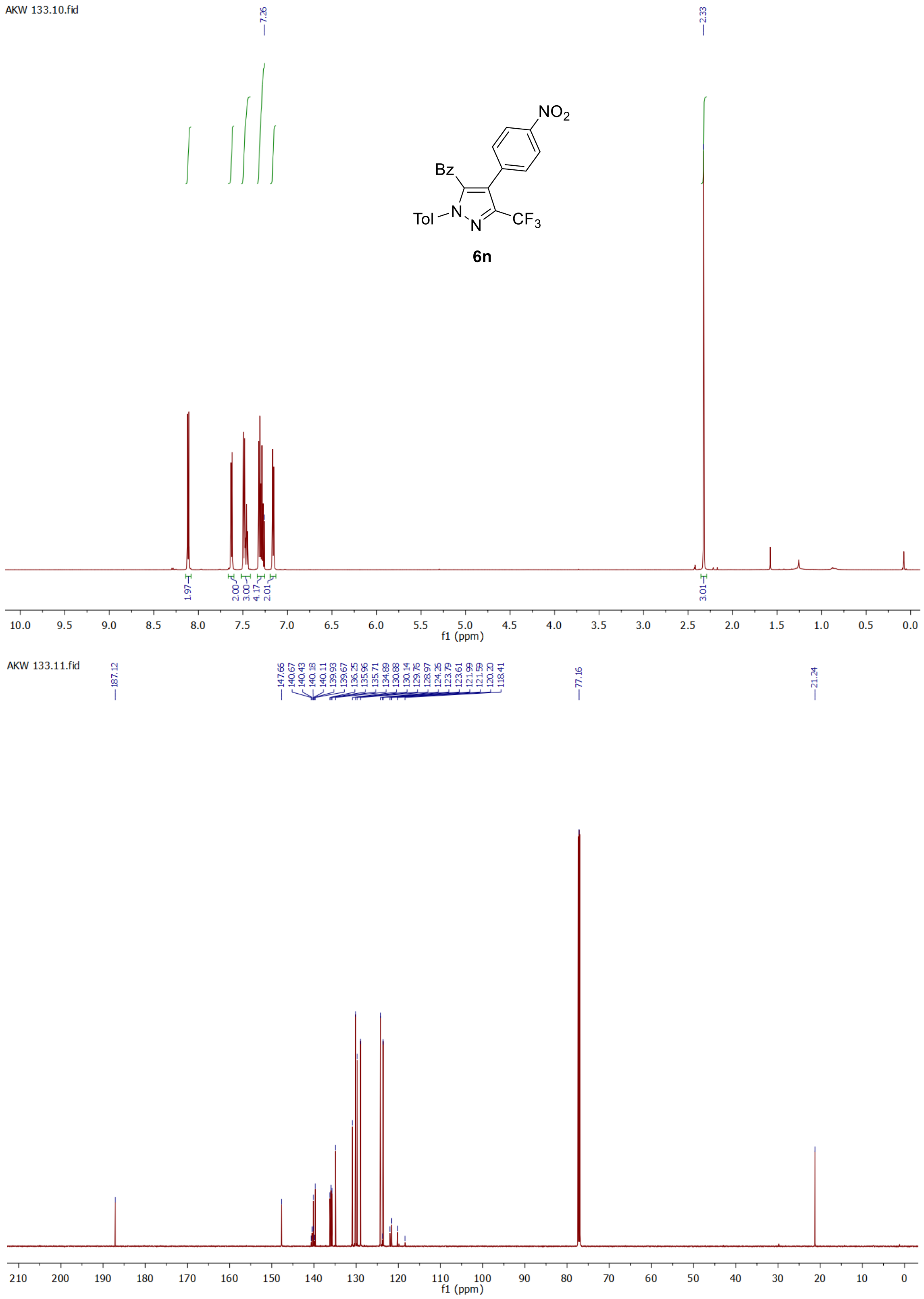

Fig S52. ^{1}H NMR (600 MHz, $CDCl_3$) and ^{13}C NMR (151 MHz, $CDCl_3$) spectra for compound **6n**.

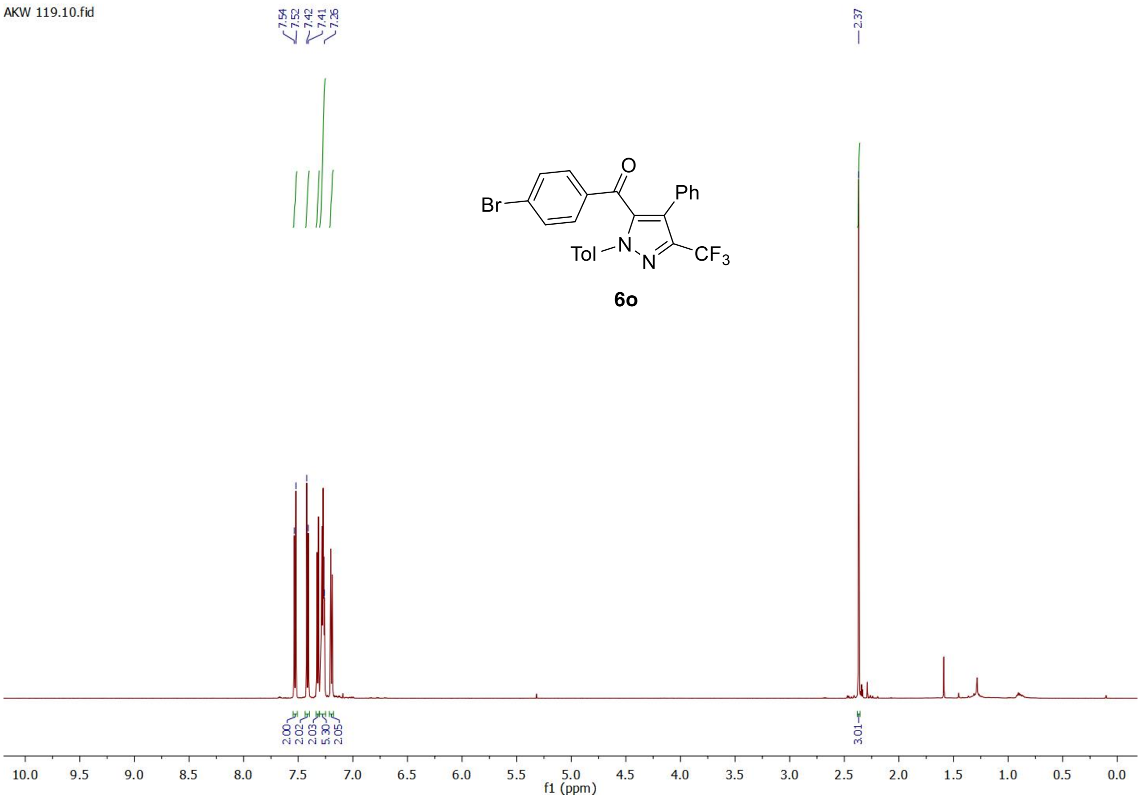

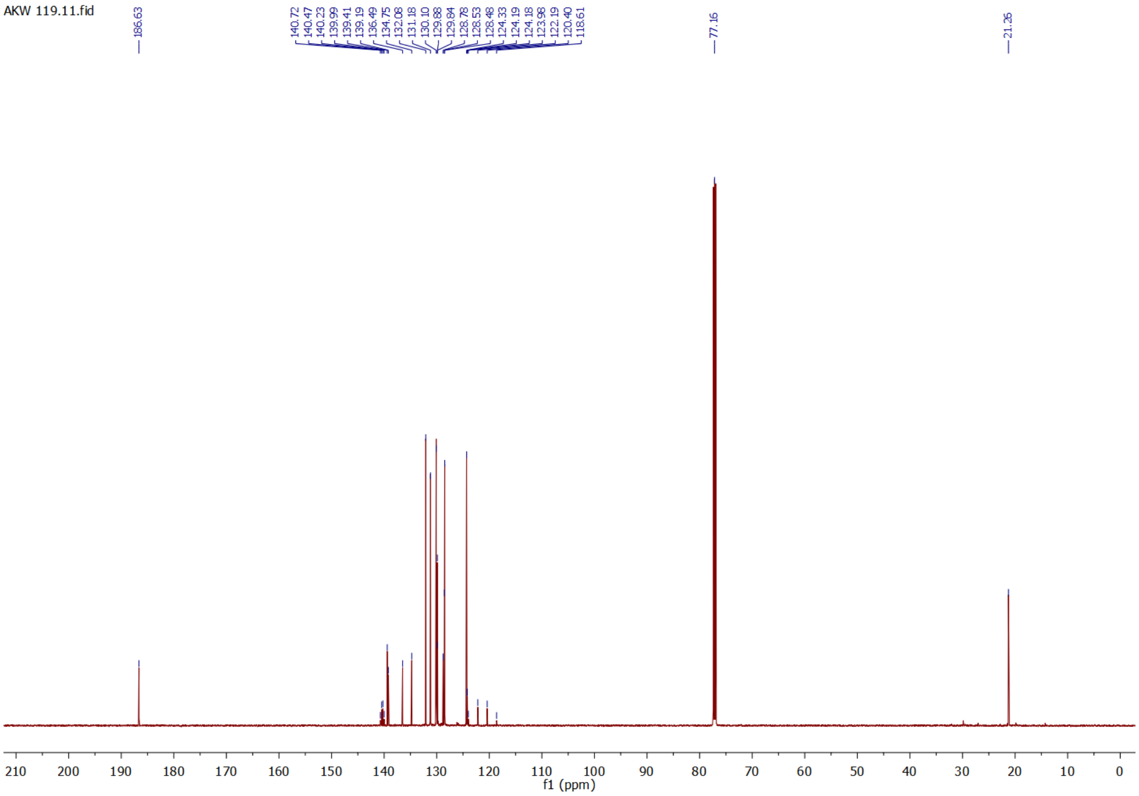

Fig S53. ^{1}H NMR (600 MHz, $CDCl_3$) and ^{13}C NMR (151 MHz, $CDCl_3$) spectra for compound **6o**.

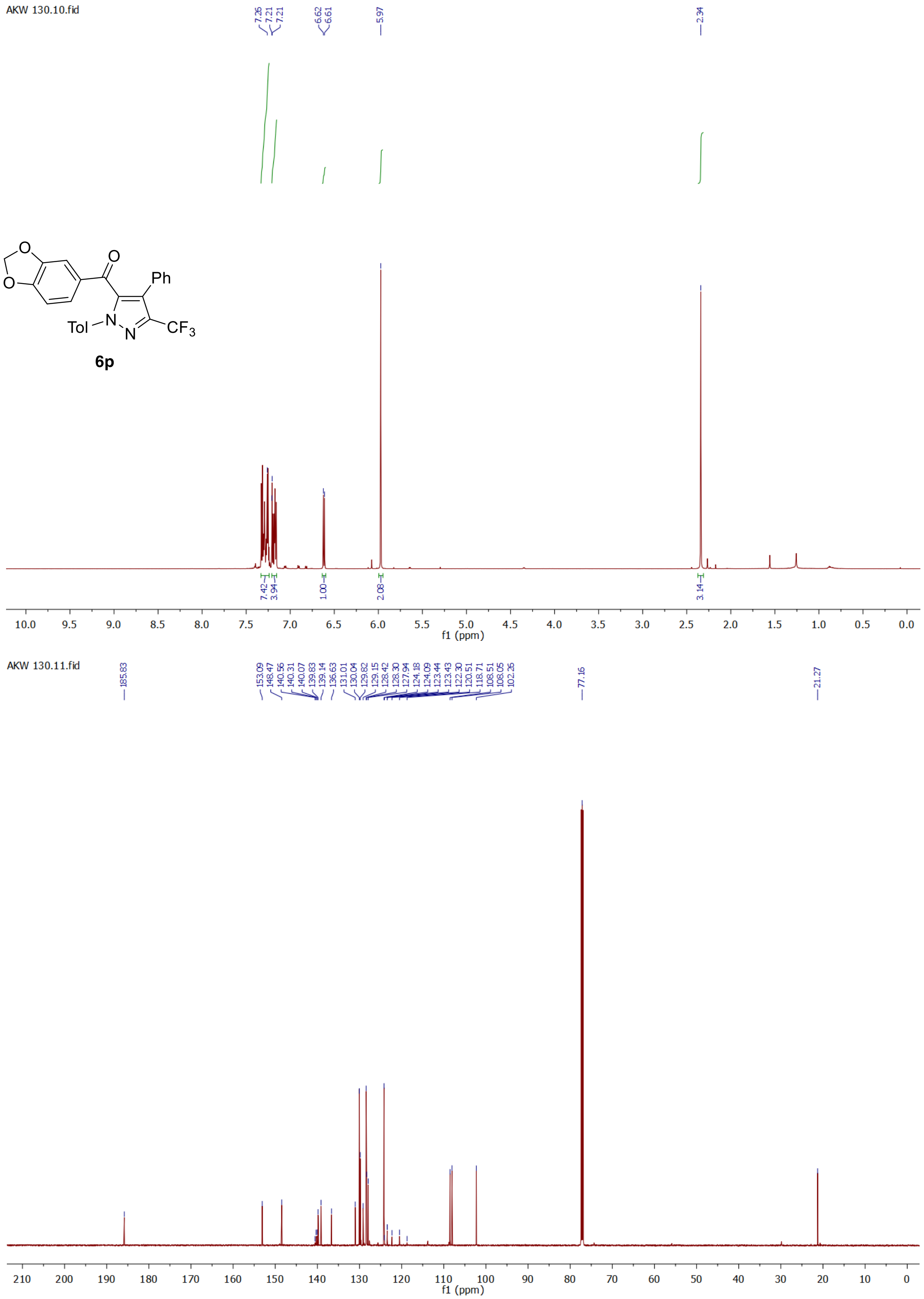

Fig S54. ^1H NMR (600 MHz, $CDCl_3$) and ^{13}C NMR (151 MHz, $CDCl_3$) spectra for compound **6p**.

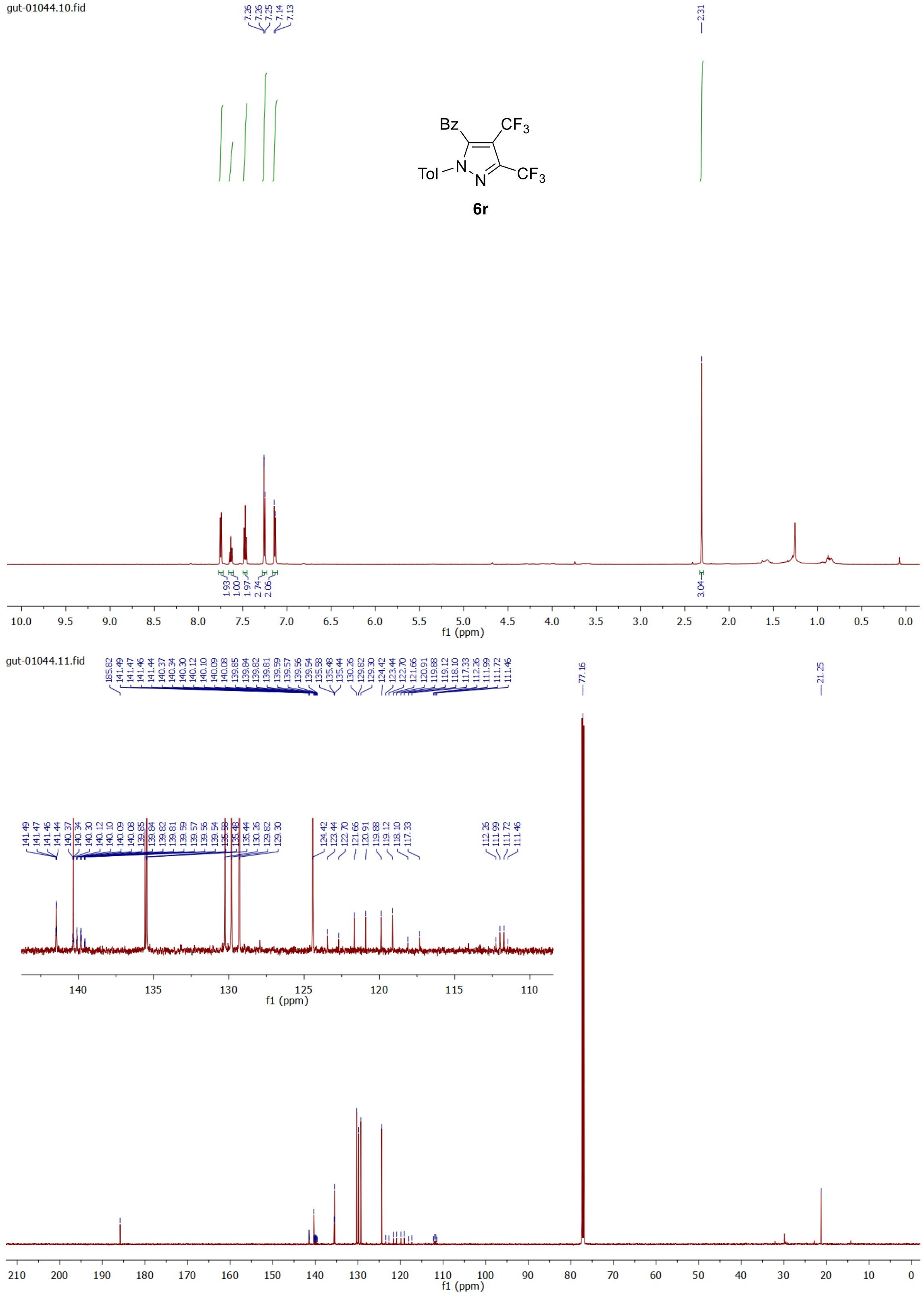

Fig S55. ^{1}H NMR (600 MHz, $CDCl_3$) and ^{13}C NMR (151 MHz, $CDCl_3$) spectra for compound **6r**.

4. Crystallographic analyses

Crystallographic analysis of 2q: A suitable crystal of compound **2q** was selected and measured on a XtaLAB Synergy, Dualflex, Pilatus 300K diffractometer. The crystal was kept at 100.01(10) K during data collection. Using Olex2,[13] the structure was solved with the XT[14] structure solution program using Intrinsic Phasing and refined with the XL[15] refinement package using Least Squares minimization. CCDC-2079231 contains the supplementary crystallographic data for this paper. These data can be obtained free of charge from the Cambridge Crystallographic Data Centre via
https://www.ccdc.cam.ac.uk/structures/

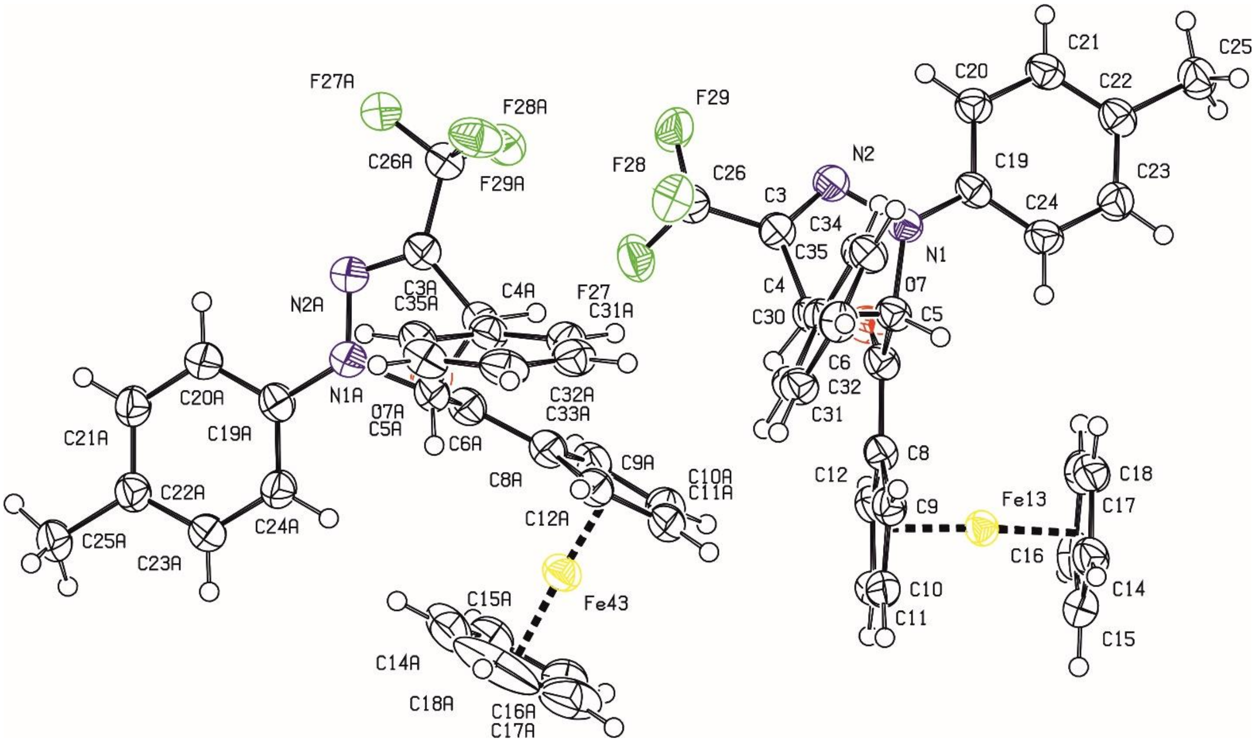

Fig S56. A view of the molecular structure of compound **2q**. Displacement ellipsoids are drawn at the 50% probability level. X-ray data collected at the ambient temperature 100 K.

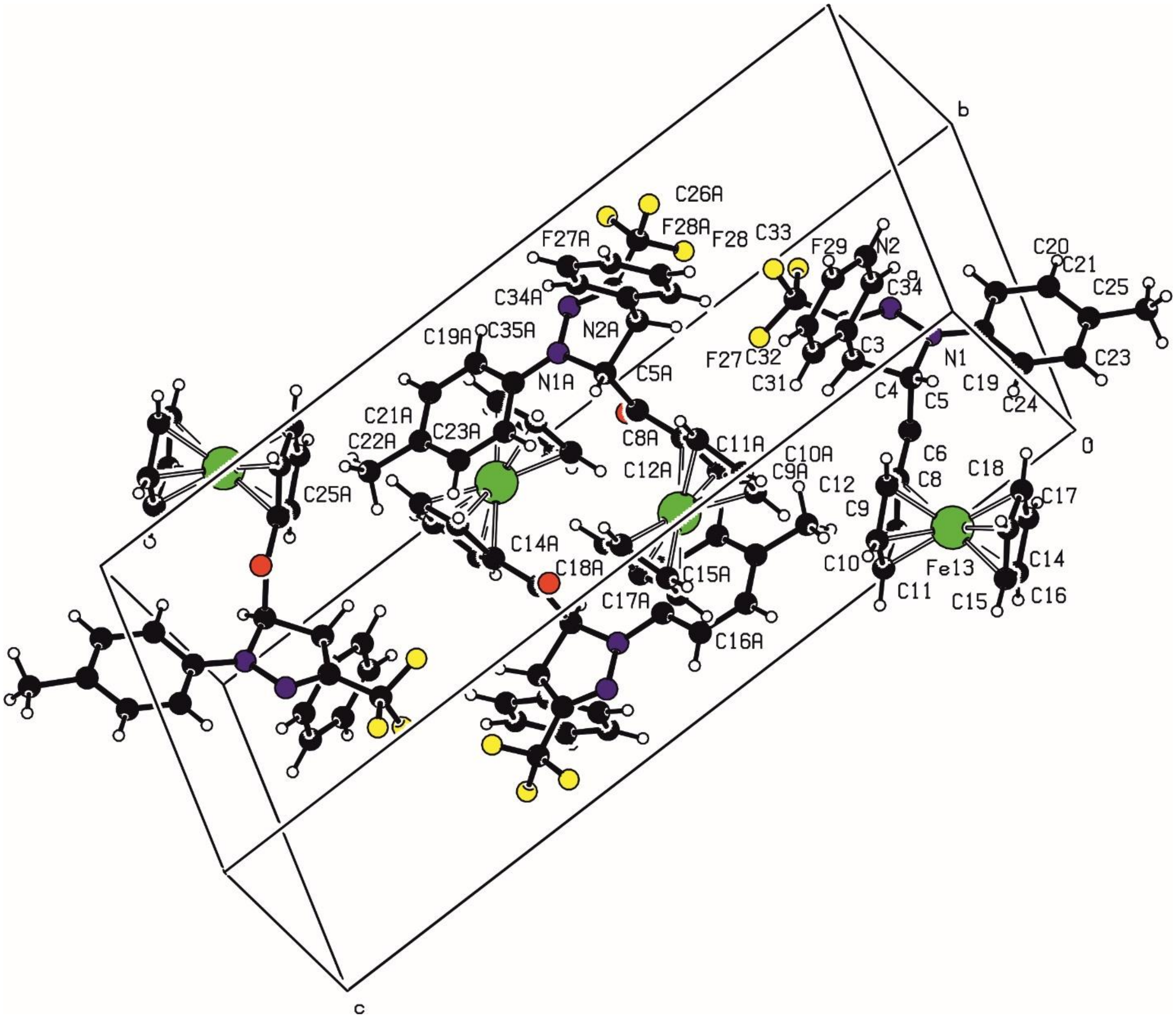

Fig S57. A view of the molecular packing in the structure of compound **2q**.

Table S1 Crystal data and structure refinement for **2q**.

Identification code	GUT_947
Empirical formula	$C_{56}H_{46}F_6Fe_2N_4O_2$
Formula weight	1032.67
Temperature/K	100.01(10)
Crystal system	triclinic
Space group	P-1
a/Å	9.8135(3)
b/Å	10.7419(3)
c/Å	23.2185(4)
α/°	99.306(2)
β/°	90.018(2)
γ/°	104.952(3)

Volume/Å^3	2331.33(11)
Z	2
ρ_{calc}g/cm^3	1.471
μ/mm^{-1}	5.606
F(000)	1064.0
Crystal size/mm^3	0.73 × 0.53 × 0.27
Radiation	Cu Kα (λ = 1.54184)
2Θ range for data collection/°	7.724 to 157.652
Index ranges	-12 ≤ h ≤ 12, -10 ≤ k ≤ 13, -26 ≤ l ≤ 29
Reflections collected	27285
Independent reflections	9445 [R_{int} = 0.0567, R_{sigma} = 0.0513]
Data/restraints/parameters	9445/0/633
Goodness-of-fit on F^2	1.058
Final R indexes [I>=2σ (I)]	R_1 = 0.0546, wR_2 = 0.1489
Final R indexes [all data]	R_1 = 0.0618, wR_2 = 0.1537
Largest diff. peak/hole / e Å^{-3}	1.02/-0.59

Table S2 Fractional Atomic Coordinates (×10^4) and Equivalent Isotropic Displacement Parameters (Å^2×10^3) for **2q**. U_{eq} is defined as 1/3 of of the trace of the orthogonalised U_{IJ} tensor.

**Atom**	***x***	***y***	***z***	**U(eq)**
Fe13	3211.7(4)	-1778.0(4)	1449.4(2)	32.39(12)
Fe43	6195.0(5)	2228.5(5)	4014.2(2)	40.31(14)
F28	7748.9(19)	5681.1(17)	1871.7(8)	50.2(4)
F27A	8659(2)	9700.4(17)	3320.6(8)	49.1(4)
F29A	7543(2)	7956.6(19)	2751.4(8)	52.3(4)
F29	5833(2)	6241.8(18)	1760.4(9)	57.9(5)
F27	6058(2)	5148.8(19)	2440.8(7)	52.8(4)
F28A	9798(2)	8593(2)	2740.1(8)	56.2(5)
O7	2657(2)	1654(2)	1472.0(9)	44.4(5)
O7A	6108(2)	5784(2)	4143.8(10)	47.6(5)
N2	4954(2)	3952(2)	1034.0(10)	35.6(5)
N1	4609(2)	2688(2)	748.2(9)	33.9(5)
N2A	8759(3)	8120(2)	4092.0(10)	38.5(5)
N1A	8825(3)	7154(2)	4397.5(10)	38.7(5)
C8	3784(3)	173(3)	1769.5(11)	32.7(5)
C4	6114(3)	2760(3)	1548.6(11)	33.5(5)
C5	4961(3)	1808(3)	1109.3(11)	33.1(5)
C20	3228(3)	3232(3)	-3.8(12)	35.4(5)
C6	3680(3)	1209(3)	1447.8(11)	33.9(5)
C9	4952(3)	-399(3)	1820.4(11)	35.6(5)
C12	2650(3)	-481(3)	2091.3(11)	35.9(5)

Table S2 Fractional Atomic Coordinates (×10^4) and Equivalent Isotropic Displacement Parameters (Å^2×10^3) for **2q**. U_{eq} is defined as 1/3 of of the trace of the orthogonalised U_{IJ} tensor.

Atom	*x*	*y*	*z*	U(eq)
C19	3650(3)	2302(3)	264.0(11)	33.6(5)
C11	3117(3)	-1417(3)	2348.5(12)	38.8(6)
C30	7619(3)	2818(3)	1381.2(11)	32.4(5)
C22	1911(3)	1511(3)	-766.6(12)	37.9(6)
C3	5742(3)	4022(3)	1483.8(11)	35.2(5)
C34	9507(3)	3292(3)	717.8(12)	37.9(6)
C18	3285(3)	-2221(3)	560.7(12)	41.2(6)
C35	8127(3)	3254(3)	867.9(11)	35.2(5)
C24A	8655(3)	6308(3)	5306.9(12)	39.2(6)
C31	8513(3)	2433(3)	1744.8(11)	35.2(5)
C5A	8557(3)	5892(3)	4014.0(11)	36.1(5)
C22A	8619(3)	7715(3)	6231.8(12)	39.6(6)
C21	2373(3)	2824(3)	-512.3(12)	38.4(6)
C4A	9039(3)	6318(3)	3416.3(11)	37.1(6)
C6A	6957(3)	5198(3)	3958.0(12)	39.4(6)
C19A	8694(3)	7342(3)	5006.1(12)	37.5(6)
C26	6337(3)	5274(3)	1882.2(12)	39.8(6)
C32	9895(3)	2481(3)	1594.2(12)	37.5(6)
C3A	8838(3)	7683(3)	3548.0(12)	38.0(6)
C24	3157(3)	991(3)	28.2(12)	38.4(6)
C8A	6572(3)	3853(3)	3632.3(12)	39.0(6)
C23A	8610(3)	6503(3)	5912.5(12)	40.3(6)
C23	2300(3)	600(3)	-482.7(12)	39.7(6)
C20A	8675(3)	8553(3)	5317.4(12)	41.0(6)
C25	1055(3)	1082(3)	-1340.2(13)	45.7(7)
C10	4524(3)	-1353(3)	2187.6(12)	38.8(6)
C30A	10549(3)	6275(3)	3297.5(12)	37.3(6)
C33	10389(3)	2904(3)	1081.8(12)	37.9(6)
C21A	8638(3)	8720(3)	5923.6(12)	41.5(6)
C26A	8715(3)	8492(3)	3094.9(12)	40.8(6)
C15	2639(3)	-3760(3)	1156.2(13)	42.4(6)
C11A	6596(4)	1816(3)	3151.7(12)	44.6(7)
C16	1476(3)	-3220(3)	1095.7(13)	45.4(7)
C35A	11663(3)	7013(3)	3684.6(12)	40.1(6)
C17	1876(3)	-2277(3)	724.5(13)	43.6(6)
C10A	5168(4)	1840(3)	3209.6(13)	46.9(7)
C12A	7480(3)	3046(3)	3411.7(12)	41.1(6)
C31A	10826(3)	5446(3)	2816.0(13)	41.6(6)
C14	3746(3)	-3141(3)	831.6(12)	40.6(6)

Table S2 Fractional Atomic Coordinates (×10^4) and Equivalent Isotropic Displacement Parameters (Å^2×10^3) for **2q**. U_{eq} is defined as 1/3 of of the trace of the orthogonalised U_{IJ} tensor.

**Atom**	***x***	***y***	***z***	**U(eq)**
C34A	13027(3)	6887(3)	3600.9(13)	45.1(7)
C25A	8629(4)	7931(3)	6894.8(12)	45.2(7)
C32A	12206(4)	5328(3)	2731.0(14)	46.2(7)
C9A	5142(3)	3091(3)	3510.2(12)	42.6(6)
C33A	13288(3)	6033(3)	3122.7(14)	47.7(7)
C16A	5097(4)	823(4)	4459.7(15)	57.6(9)
C15A	5125(4)	2041(4)	4768.3(14)	54.9(8)
C14A	6501(5)	2738(5)	4896.0(16)	74.8(13)
C17A	6457(6)	711(5)	4393.0(19)	77.8(14)
C18A	7380(5)	1930(8)	4667(2)	112(3)

Table S3 Anisotropic Displacement Parameters (Å^2×10^3) for **2q**. The Anisotropic displacement factor exponent takes the form: $-2\pi^2[h^2a^{*2}U_{11}+2hka^*b^*U_{12}+\ldots]$.

**Atom**	U_{11}	U_{22}	U_{33}	U_{23}	U_{13}	U_{12}
Fe13	34.8(2)	34.6(2)	28.7(2)	3.65(16)	2.57(15)	11.71(16)
Fe43	43.8(3)	44.1(3)	32.8(2)	10.60(18)	3.57(18)	8.64(19)
F28	44.4(9)	45.0(10)	57.0(10)	-1.0(8)	2.7(8)	10.3(7)
F27A	63.3(11)	42.0(10)	44.3(9)	8.4(7)	-2.1(8)	17.2(8)
F29A	54.7(10)	53.9(11)	48.0(9)	10.1(8)	-13.1(8)	12.8(8)
F29	77.7(13)	45.9(10)	55.2(10)	-7.1(8)	-22.4(9)	34.7(9)
F27	62.6(11)	57.5(11)	34.1(8)	0.3(8)	3.5(8)	12.4(9)
F28A	57.9(11)	71.0(13)	53.0(10)	29.8(9)	19.7(9)	28.5(9)
O7	39.9(10)	54.0(12)	49.6(11)	19.9(10)	10.9(9)	23.7(9)
O7A	42.4(11)	49.0(12)	51.8(12)	2.8(10)	6.4(9)	16.0(9)
N2	38.1(11)	37.6(12)	33.6(10)	5.6(9)	2.6(9)	14.4(9)
N1	36.7(11)	36.0(12)	31.3(10)	7.4(9)	-0.1(9)	12.3(9)
N2A	41.8(12)	37.4(12)	37.5(12)	8.8(10)	1.7(9)	10.9(9)
N1A	46.2(13)	35.9(12)	34.4(11)	7.3(9)	2.8(9)	10.6(10)
C8	32.6(12)	36.5(14)	29.4(11)	3.9(10)	-0.1(9)	10.9(10)
C4	35.1(13)	39.1(14)	29.1(11)	5.4(10)	2.5(10)	15.0(10)
C5	33.9(12)	37.4(14)	30.7(11)	6.9(10)	2.1(10)	13.2(10)
C20	36.2(13)	36.9(14)	35.0(12)	7.5(11)	2.5(10)	12.0(10)
C6	32.7(12)	39.7(14)	31.1(12)	6.1(10)	1.5(10)	12.3(10)
C9	35.4(13)	40.1(14)	32.3(12)	0.9(11)	-1.1(10)	14.6(11)
C12	40.1(13)	39.3(14)	29.0(11)	4.4(10)	4.8(10)	12.4(11)
C19	32.3(12)	42.2(14)	28.6(11)	8.1(10)	4.4(9)	12.4(10)
C11	49.0(15)	37.1(14)	32.3(12)	8.6(11)	4.9(11)	13.1(12)
C30	33.7(12)	33.9(13)	30.0(11)	2.7(10)	1.1(9)	11.1(10)

Table S3 Anisotropic Displacement Parameters (Å^2×10^3) for **2q**. The Anisotropic displacement factor exponent takes the form: $-2\pi^2[h^2a^{*2}U_{11}+2hka^*b^*U_{12}+\ldots]$.

Atom	U_{11}	U_{22}	U_{33}	U_{23}	U_{13}	U_{12}
C22	35.5(13)	47.2(16)	33.5(12)	7.2(11)	0.5(10)	15.1(11)
C3	35.4(13)	39.7(14)	33.6(12)	5.4(11)	2.7(10)	15.8(11)
C34	37.0(13)	43.8(15)	33.6(12)	8.6(11)	6.3(10)	10.4(11)
C18	51.1(16)	43.3(16)	31.8(12)	5.0(11)	2.2(11)	17.9(12)
C35	37.4(13)	36.2(14)	33.4(12)	5.5(10)	1.6(10)	12.5(10)
C24A	45.0(15)	35.3(14)	36.7(13)	3.1(11)	4.8(11)	11.0(11)
C31	38.2(13)	38.8(14)	30.7(12)	6.1(10)	3.0(10)	13.8(11)
C5A	39.2(13)	38.6(14)	30.7(12)	6.0(10)	3.6(10)	10.1(11)
C22A	43.1(14)	40.5(15)	33.7(13)	4.1(11)	4.1(11)	9.7(11)
C21	40.5(14)	44.5(16)	35.0(13)	10.1(11)	2.9(11)	17.7(11)
C4A	38.7(13)	40.6(15)	31.6(12)	6.6(11)	0.5(10)	9.5(11)
C6A	41.5(14)	43.2(16)	34.1(13)	9.1(11)	5.3(11)	10.5(12)
C19A	37.6(13)	42.8(15)	32.4(12)	5.4(11)	4.3(10)	11.4(11)
C26	40.2(14)	45.8(16)	37.2(13)	4.6(12)	-1.6(11)	19.4(12)
C32	36.9(13)	41.8(15)	37.7(13)	7.9(11)	0.4(11)	16.6(11)
C3A	39.4(14)	39.5(15)	35.3(13)	7.0(11)	2.3(11)	10.3(11)
C24	42.1(14)	40.1(15)	37.3(13)	9.9(11)	1.2(11)	16.3(11)
C8A	40.3(14)	43.1(15)	34.0(12)	8.6(11)	3.6(11)	10.2(11)
C23A	45.5(15)	39.8(15)	35.6(13)	7.6(11)	3.6(11)	10.2(12)
C23	41.2(14)	38.6(15)	39.1(14)	2.8(11)	-3.6(11)	12.4(11)
C20A	47.2(15)	38.2(15)	37.8(13)	7.5(11)	2.7(11)	10.9(12)
C25	49.7(16)	51.0(18)	39.0(14)	2.2(13)	-5.8(12)	20.8(14)
C10	47.5(15)	35.5(14)	34.1(13)	1.6(11)	-5.7(11)	14.6(11)
C30A	42.7(14)	39.8(15)	33.1(12)	11.8(11)	5.3(11)	13.6(11)
C33	33.1(13)	41.5(15)	39.7(13)	3.9(12)	5.9(11)	12.3(11)
C21A	46.8(15)	39.2(15)	36.9(14)	1.2(11)	3.5(11)	11.5(12)
C26A	45.1(15)	41.8(15)	36.4(13)	8.4(12)	-1.0(11)	12.0(12)
C15	52.5(16)	35.0(14)	38.4(14)	1.3(11)	0.6(12)	12.1(12)
C11A	58.5(18)	40.7(16)	32.6(13)	7.6(12)	5.0(12)	8.7(13)
C16	40.8(15)	48.2(17)	39.8(14)	-7.2(12)	4.1(12)	7.7(12)
C35A	40.1(14)	47.2(16)	33.9(12)	8.5(11)	5.2(11)	12.1(12)
C17	45.4(15)	48.5(17)	37.3(14)	-4.7(12)	-6.5(12)	20.4(13)
C10A	52.3(17)	42.9(16)	40.4(14)	8.9(12)	-3.4(13)	2.2(13)
C12A	48.3(16)	41.9(16)	32.9(13)	7.5(11)	6.4(11)	10.5(12)
C31A	49.3(16)	41.4(15)	36.9(13)	10.6(12)	6.3(12)	14.0(12)
C14	45.3(15)	46.2(16)	33.1(13)	2.7(11)	2.2(11)	19.8(12)
C34A	40.5(15)	57.2(19)	40.5(14)	17.4(13)	2.2(12)	12.2(13)
C25A	55.7(17)	44.9(16)	32.5(13)	2.2(12)	4.1(12)	11.6(13)
C32A	57.4(18)	45.6(17)	43.0(15)	14.9(13)	17.0(13)	21.8(14)

Table S3 Anisotropic Displacement Parameters (Å^2×10^3) for **2q**. The Anisotropic displacement factor exponent takes the form: $-2\pi^2[h^2a^{*2}U_{11}+2hka^*b^*U_{12}+\ldots]$.

Atom	U_{11}	U_{22}	U_{33}	U_{23}	U_{13}	U_{12}
C9A	41.8(15)	46.9(16)	36.3(13)	8.4(12)	-2.8(11)	5.3(12)
C33A	45.3(16)	58.8(19)	48.8(16)	24.4(15)	13.3(13)	21.6(14)
C16A	73(2)	52(2)	46.5(17)	18.5(15)	10.9(16)	6.6(16)
C15A	65(2)	62(2)	38.2(15)	16.5(15)	14.6(14)	14.0(16)
C14A	89(3)	79(3)	39.0(17)	21.1(18)	-7.5(18)	-16(2)
C17A	98(3)	100(3)	64(2)	46(2)	35(2)	57(3)
C18A	43(2)	230(8)	87(3)	110(4)	7(2)	27(3)

Table S4 Bond Lengths for **2q**.

Atom	Atom	Length/Å	Atom	Atom	Length/Å
Fe13	C8	2.036(3)	C19	C24	1.384(4)
Fe13	C9	2.033(3)	C11	C10	1.418(4)
Fe13	C12	2.047(3)	C30	C35	1.396(4)
Fe13	C11	2.067(3)	C30	C31	1.398(4)
Fe13	C18	2.047(3)	C22	C21	1.395(4)
Fe13	C10	2.061(3)	C22	C23	1.392(4)
Fe13	C15	2.056(3)	C22	C25	1.514(4)
Fe13	C16	2.050(3)	C3	C26	1.483(4)
Fe13	C17	2.045(3)	C34	C35	1.391(4)
Fe13	C14	2.047(3)	C34	C33	1.391(4)
Fe43	C8A	2.033(3)	C18	C17	1.423(4)
Fe43	C11A	2.041(3)	C18	C14	1.417(4)
Fe43	C10A	2.052(3)	C24A	C19A	1.397(4)
Fe43	C12A	2.036(3)	C24A	C23A	1.390(4)
Fe43	C9A	2.035(3)	C31	C32	1.391(4)
Fe43	C16A	2.043(3)	C5A	C4A	1.566(4)
Fe43	C15A	2.053(3)	C5A	C6A	1.550(4)
Fe43	C14A	2.033(4)	C22A	C23A	1.388(4)
Fe43	C17A	2.043(4)	C22A	C21A	1.385(4)
Fe43	C18A	2.026(4)	C22A	C25A	1.519(4)
F28	C26	1.342(3)	C4A	C3A	1.511(4)
F27A	C26A	1.334(3)	C4A	C30A	1.519(4)
F29A	C26A	1.341(4)	C6A	C8A	1.472(4)
F29	C26	1.329(3)	C19A	C20A	1.387(4)
F27	C26	1.346(3)	C32	C33	1.385(4)
F28A	C26A	1.338(3)	C3A	C26A	1.492(4)
O7	C6	1.216(3)	C24	C23	1.395(4)
O7A	C6A	1.208(4)	C8A	C12A	1.436(4)

Table S4 Bond Lengths for **2q**.

Atom	Atom	Length/Å	Atom	Atom	Length/Å
N2	N1	1.367(3)	C8A	C9A	1.432(4)
N2	C3	1.280(3)	C20A	C21A	1.392(4)
N1	C5	1.464(3)	C30A	C35A	1.397(4)
N1	C19	1.403(3)	C30A	C31A	1.385(4)
N2A	N1A	1.363(3)	C15	C16	1.424(4)
N2A	C3A	1.284(4)	C15	C14	1.407(4)
N1A	C5A	1.458(4)	C11A	C10A	1.415(5)
N1A	C19A	1.405(3)	C11A	C12A	1.419(4)
C8	C6	1.461(4)	C16	C17	1.414(5)
C8	C9	1.446(3)	C35A	C34A	1.391(4)
C8	C12	1.434(4)	C10A	C9A	1.417(5)
C4	C5	1.559(4)	C31A	C32A	1.404(4)
C4	C30	1.516(3)	C34A	C33A	1.390(5)
C4	C3	1.521(4)	C32A	C33A	1.375(5)
C5	C6	1.536(4)	C16A	C15A	1.380(5)
C20	C19	1.403(4)	C16A	C17A	1.376(6)
C20	C21	1.390(4)	C15A	C14A	1.371(6)
C9	C10	1.419(4)	C14A	C18A	1.421(8)
C12	C11	1.417(4)	C17A	C18A	1.434(9)

Table S5 Bond Angles for **2q**.

Atom	Atom	Atom	Angle/˚	Atom	Atom	Atom	Angle/˚
C8	Fe13	C12	41.11(10)	C11	C12	Fe13	70.61(16)
C8	Fe13	C11	68.37(11)	C11	C12	C8	108.0(2)
C8	Fe13	C18	114.11(11)	N1	C19	C20	120.9(2)
C8	Fe13	C10	68.27(11)	C24	C19	N1	119.9(2)
C8	Fe13	C15	177.95(11)	C24	C19	C20	119.1(2)
C8	Fe13	C16	140.09(12)	C12	C11	Fe13	69.09(15)
C8	Fe13	C17	113.92(12)	C12	C11	C10	108.1(2)
C8	Fe13	C14	141.17(11)	C10	C11	Fe13	69.65(15)
C9	Fe13	C8	41.63(10)	C35	C30	C4	120.8(2)
C9	Fe13	C12	69.59(11)	C35	C30	C31	119.5(2)
C9	Fe13	C11	68.75(11)	C31	C30	C4	119.7(2)
C9	Fe13	C18	112.85(12)	C21	C22	C25	121.7(3)
C9	Fe13	C10	40.56(11)	C23	C22	C21	117.6(3)
C9	Fe13	C15	137.60(12)	C23	C22	C25	120.7(3)
C9	Fe13	C16	177.66(12)	N2	C3	C4	115.2(2)
C9	Fe13	C17	141.77(12)	N2	C3	C26	121.8(2)
C9	Fe13	C14	111.36(11)	C26	C3	C4	122.8(2)
C12	Fe13	C11	40.29(11)	C33	C34	C35	120.0(2)

Table S5 Bond Angles for **2q**.

Atom	Atom	Atom	Angle/˚	Atom	Atom	Atom	Angle/˚
C12	Fe13	C10	67.93(11)	C17	C18	Fe13	69.59(16)
C12	Fe13	C15	137.56(12)	C14	C18	Fe13	69.77(16)
C12	Fe13	C16	110.82(11)	C14	C18	C17	107.4(3)
C12	Fe13	C14	177.33(11)	C34	C35	C30	120.1(2)
C18	Fe13	C12	141.77(11)	C23A	C24A	C19A	120.0(3)
C18	Fe13	C11	177.51(12)	C32	C31	C30	120.1(2)
C18	Fe13	C10	139.90(12)	N1A	C5A	C4A	101.4(2)
C18	Fe13	C15	67.89(12)	N1A	C5A	C6A	110.8(2)
C18	Fe13	C16	68.36(12)	C6A	C5A	C4A	108.9(2)
C18	Fe13	C14	40.50(11)	C23A	C22A	C25A	121.0(3)
C10	Fe13	C11	40.19(12)	C21A	C22A	C23A	117.5(3)
C15	Fe13	C11	109.63(12)	C21A	C22A	C25A	121.4(3)
C15	Fe13	C10	109.97(12)	C20	C21	C22	122.0(3)
C16	Fe13	C11	109.97(12)	C3A	C4A	C5A	97.4(2)
C16	Fe13	C10	137.25(13)	C3A	C4A	C30A	114.2(2)
C16	Fe13	C15	40.60(12)	C30A	C4A	C5A	112.3(2)
C17	Fe13	C12	112.91(11)	O7A	C6A	C5A	120.2(3)
C17	Fe13	C11	139.06(12)	O7A	C6A	C8A	123.9(3)
C17	Fe13	C18	40.69(12)	C8A	C6A	C5A	115.7(2)
C17	Fe13	C10	177.58(12)	C24A	C19A	N1A	119.4(3)
C17	Fe13	C15	67.82(12)	C20A	C19A	N1A	121.2(3)
C17	Fe13	C16	40.40(13)	C20A	C19A	C24A	119.3(3)
C17	Fe13	C14	68.02(11)	F28	C26	F27	105.5(2)
C14	Fe13	C11	137.38(11)	F28	C26	C3	112.7(2)
C14	Fe13	C10	111.04(11)	F29	C26	F28	106.8(2)
C14	Fe13	C15	40.11(12)	F29	C26	F27	107.5(2)
C14	Fe13	C16	68.12(12)	F29	C26	C3	113.0(2)
C8A	Fe43	C11A	68.59(12)	F27	C26	C3	110.9(2)
C8A	Fe43	C10A	68.67(12)	C33	C32	C31	120.1(2)
C8A	Fe43	C12A	41.32(12)	N2A	C3A	C4A	115.0(2)
C8A	Fe43	C9A	41.22(12)	N2A	C3A	C26A	120.6(3)
C8A	Fe43	C16A	157.15(14)	C26A	C3A	C4A	124.3(2)
C8A	Fe43	C15A	123.81(13)	C19	C24	C23	120.6(3)
C8A	Fe43	C14A	110.32(16)	C6A	C8A	Fe43	124.05(19)
C8A	Fe43	C17A	162.87(17)	C12A	C8A	Fe43	69.44(16)
C11A	Fe43	C10A	40.45(13)	C12A	C8A	C6A	128.8(3)
C11A	Fe43	C16A	122.02(14)	C9A	C8A	Fe43	69.44(17)
C11A	Fe43	C15A	157.50(14)	C9A	C8A	C6A	123.3(3)
C11A	Fe43	C17A	106.83(16)	C9A	C8A	C12A	107.8(3)
C10A	Fe43	C15A	122.07(15)	C22A	C23A	C24A	121.5(3)

Table S5 Bond Angles for **2q**.

Atom	Atom	Atom	Angle/˚	Atom	Atom	Atom	Angle/˚
C12A	Fe43	C11A	40.73(12)	C22	C23	C24	121.1(3)
C12A	Fe43	C10A	68.78(12)	C19A	C20A	C21A	119.4(3)
C12A	Fe43	C16A	159.04(14)	C9	C10	Fe13	68.66(15)
C12A	Fe43	C15A	160.57(14)	C11	C10	Fe13	70.15(16)
C12A	Fe43	C17A	124.57(15)	C11	C10	C9	109.4(2)
C9A	Fe43	C11A	68.40(13)	C35A	C30A	C4A	120.8(2)
C9A	Fe43	C10A	40.57(13)	C31A	C30A	C4A	119.9(3)
C9A	Fe43	C12A	69.41(12)	C31A	C30A	C35A	119.2(3)
C9A	Fe43	C16A	120.04(15)	C32	C33	C34	120.1(2)
C9A	Fe43	C15A	107.45(14)	C22A	C21A	C20A	122.3(3)
C9A	Fe43	C17A	154.1(2)	F27A	C26A	F29A	106.5(2)
C16A	Fe43	C10A	105.38(14)	F27A	C26A	F28A	107.6(2)
C16A	Fe43	C15A	39.39(15)	F27A	C26A	C3A	113.2(2)
C14A	Fe43	C11A	160.92(18)	F29A	C26A	C3A	111.5(2)
C14A	Fe43	C10A	158.33(19)	F28A	C26A	F29A	106.2(2)
C14A	Fe43	C12A	125.74(15)	F28A	C26A	C3A	111.5(2)
C14A	Fe43	C9A	124.23(19)	C16	C15	Fe13	69.48(17)
C14A	Fe43	C16A	66.67(16)	C14	C15	Fe13	69.63(17)
C14A	Fe43	C15A	39.19(17)	C14	C15	C16	108.3(3)
C14A	Fe43	C17A	68.3(2)	C10A	C11A	Fe43	70.19(17)
C17A	Fe43	C10A	119.29(19)	C10A	C11A	C12A	109.1(3)
C17A	Fe43	C16A	39.37(18)	C12A	C11A	Fe43	69.45(16)
C17A	Fe43	C15A	66.70(16)	C15	C16	Fe13	69.91(17)
C18A	Fe43	C8A	126.3(2)	C17	C16	Fe13	69.61(17)
C18A	Fe43	C11A	123.2(2)	C17	C16	C15	107.4(3)
C18A	Fe43	C10A	156.9(3)	C34A	C35A	C30A	120.6(3)
C18A	Fe43	C12A	109.61(15)	C18	C17	Fe13	69.72(16)
C18A	Fe43	C9A	162.2(3)	C16	C17	Fe13	69.99(17)
C18A	Fe43	C16A	67.44(19)	C16	C17	C18	108.5(3)
C18A	Fe43	C15A	67.20(17)	C11A	C10A	Fe43	69.37(17)
C18A	Fe43	C14A	41.0(2)	C11A	C10A	C9A	108.0(3)
C18A	Fe43	C17A	41.3(3)	C9A	C10A	Fe43	69.07(17)
C3	N2	N1	108.0(2)	C8A	C12A	Fe43	69.23(16)
N2	N1	C5	111.4(2)	C11A	C12A	Fe43	69.82(16)
N2	N1	C19	120.4(2)	C11A	C12A	C8A	107.1(3)
C19	N1	C5	125.2(2)	C30A	C31A	C32A	119.9(3)
C3A	N2A	N1A	107.7(2)	C18	C14	Fe13	69.72(16)
N2A	N1A	C5A	111.1(2)	C15	C14	Fe13	70.27(16)
N2A	N1A	C19A	120.1(2)	C15	C14	C18	108.4(3)
C19A	N1A	C5A	125.5(2)	C33A	C34A	C35A	119.6(3)

Table S5 Bond Angles for **2q**.

Atom	Atom	Atom	Angle/˚	Atom	Atom	Atom	Angle/˚
C6	C8	Fe13	125.63(18)	C33A	C32A	C31A	120.4(3)
C9	C8	Fe13	69.05(15)	C8A	C9A	Fe43	69.33(17)
C9	C8	C6	129.0(2)	C10A	C9A	Fe43	70.36(18)
C12	C8	Fe13	69.85(15)	C10A	C9A	C8A	108.0(3)
C12	C8	C6	123.1(2)	C32A	C33A	C34A	120.2(3)
C12	C8	C9	107.9(2)	C15A	C16A	Fe43	70.68(19)
C30	C4	C5	114.6(2)	C17A	C16A	Fe43	70.3(2)
C30	C4	C3	110.8(2)	C17A	C16A	C15A	109.5(4)
C3	C4	C5	97.8(2)	C16A	C15A	Fe43	69.93(19)
N1	C5	C4	102.2(2)	C14A	C15A	Fe43	69.6(2)
N1	C5	C6	110.6(2)	C14A	C15A	C16A	109.1(4)
C6	C5	C4	109.1(2)	C15A	C14A	Fe43	71.2(2)
C21	C20	C19	119.5(3)	C15A	C14A	C18A	107.9(4)
O7	C6	C8	122.0(2)	C18A	C14A	Fe43	69.2(2)
O7	C6	C5	119.9(2)	C16A	C17A	Fe43	70.3(2)
C8	C6	C5	117.9(2)	C16A	C17A	C18A	107.0(4)
C8	C9	Fe13	69.31(15)	C18A	C17A	Fe43	68.7(3)
C10	C9	Fe13	70.77(16)	C14A	C18A	Fe43	69.8(2)
C10	C9	C8	106.7(2)	C14A	C18A	C17A	106.5(4)
C8	C12	Fe13	69.04(15)	C17A	C18A	Fe43	70.0(2)

Table S6 Torsion Angles for **2q**.

A	B	C	D	Angle/˚	A	B	C	D	Angle/˚
Fe13	C8	C6	O7	93.8(3)	C3	N2	N1	C5	13.0(3)
Fe13	C8	C6	C5	-90.9(3)	C3	N2	N1	C19	174.5(2)
Fe13	C8	C9	C10	-61.26(18)	C3	C4	C5	N1	20.9(2)
Fe13	C8	C12	C11	60.14(19)	C3	C4	C5	C6	-96.2(2)
Fe13	C9	C10	C11	-58.4(2)	C3	C4	C30	C35	-47.0(3)
Fe13	C12	C11	C10	58.93(19)	C3	C4	C30	C31	132.9(3)
Fe13	C11	C10	C9	57.53(19)	C35	C30	C31	C32	-0.2(4)
Fe13	C18	C17	C16	-59.5(2)	C35	C34	C33	C32	-0.1(4)
Fe13	C18	C14	C15	59.8(2)	C24A	C19A	C20A	C21A	0.9(4)
Fe13	C15	C16	C17	59.8(2)	C31	C30	C35	C34	0.6(4)
Fe13	C15	C14	C18	-59.5(2)	C31	C32	C33	C34	0.5(4)
Fe13	C16	C17	C18	59.3(2)	C5A	N1A	C19A	C24A	17.3(4)
Fe43	C8A	C12A	C11A	59.85(19)	C5A	N1A	C19A	C20A	-166.1(3)
Fe43	C8A	C9A	C10A	-60.0(2)	C5A	C4A	C3A	N2A	17.9(3)
Fe43	C11A	C10A	C9A	58.4(2)	C5A	C4A	C3A	C26A	-161.2(3)
Fe43	C11A	C12A	C8A	-59.47(19)	C5A	C4A	C30A	C35A	-58.5(3)

Fe43	C10A	C9A	C8A	59.4(2)	C5A	C4A	C30A	C31A	118.2(3)
Fe43	C16A	C15A	C14A	-58.8(2)	C5A	C6A	C8A	Fe43	97.2(3)
Fe43	C16A	C17A	C18A	59.2(3)	C5A	C6A	C8A	C12A	7.2(4)
Fe43	C15A	C14A	C18A	-59.7(3)	C5A	C6A	C8A	C9A	-176.4(2)
Fe43	C14A	C18A	C17A	-60.7(3)	C21	C20	C19	N1	174.5(2)
Fe43	C17A	C18A	C14A	60.6(3)	C21	C20	C19	C24	-2.6(4)
O7A	C6A	C8A	Fe43	-87.4(3)	C21	C22	C23	C24	-1.9(4)
O7A	C6A	C8A	C12A	-177.4(3)	C4A	C5A	C6A	O7A	-99.9(3)
O7A	C6A	C8A	C9A	-1.0(4)	C4A	C5A	C6A	C8A	75.7(3)
N2	N1	C5	C4	-22.4(2)	C4A	C3A	C26A	F27A	-174.9(2)
N2	N1	C5	C6	93.6(2)	C4A	C3A	C26A	F29A	65.1(4)
N2	N1	C19	C20	11.9(4)	C4A	C3A	C26A	F28A	-53.4(4)
N2	N1	C19	C24	-171.0(2)	C4A	C30A	C35A	C34A	174.5(3)
N2	C3	C26	F28	-113.6(3)	C4A	C30A	C31A	C32A	-175.0(3)
N2	C3	C26	F29	7.5(4)	C6A	C5A	C4A	C3A	92.7(2)
N2	C3	C26	F27	128.3(3)	C6A	C5A	C4A	C30A	-147.3(2)
N1	N2	C3	C4	3.1(3)	C6A	C8A	C12A	Fe43	117.8(3)
N1	N2	C3	C26	178.3(2)	C6A	C8A	C12A	C11A	177.6(3)
N1	C5	C6	O7	-12.6(4)	C6A	C8A	C9A	Fe43	-118.0(3)
N1	C5	C6	C8	171.9(2)	C6A	C8A	C9A	C10A	-178.0(3)
N1	C19	C24	C23	-174.4(2)	C19A	N1A	C5A	C4A	-173.9(2)
N2A	N1A	C5A	C4A	26.5(3)	C19A	N1A	C5A	C6A	70.6(3)
N2A	N1A	C5A	C6A	-88.9(3)	C19A	C24A	C23A	C22A	-0.7(4)
N2A	N1A	C19A	C24A	175.1(3)	C19A	C20A	C21A	C22A	0.0(5)
N2A	N1A	C19A	C20A	-8.2(4)	C3A	N2A	N1A	C5A	-16.2(3)
N2A	C3A	C26A	F27A	6.1(4)	C3A	N2A	N1A	C19A	-177.0(2)
N2A	C3A	C26A	F29A	-114.0(3)	C3A	C4A	C30A	C35A	51.1(3)
N2A	C3A	C26A	F28A	127.5(3)	C3A	C4A	C30A	C31A	-132.2(3)
N1A	N2A	C3A	C4A	-2.6(3)	C23A	C24A	C19A	N1A	176.2(3)
N1A	N2A	C3A	C26A	176.6(2)	C23A	C24A	C19A	C20A	-0.6(4)
N1A	C5A	C4A	C3A	-24.2(2)	C23A	C22A	C21A	C20A	-1.2(5)
N1A	C5A	C4A	C30A	95.8(3)	C23	C22	C21	C20	2.0(4)
N1A	C5A	C6A	O7A	10.8(4)	C25	C22	C21	C20	-176.1(3)
N1A	C5A	C6A	C8A	-173.6(2)	C25	C22	C23	C24	176.2(3)
N1A	C19A	C20A	C21A	-175.8(3)	C30A	C4A	C3A	N2A	-100.7(3)
C8	C9	C10	Fe13	60.31(18)	C30A	C4A	C3A	C26A	80.2(3)
C8	C9	C10	C11	1.9(3)	C30A	C35A	C34A	C33A	0.9(4)
C8	C12	C11	Fe13	-59.15(18)	C30A	C31A	C32A	C33A	-0.1(4)
C8	C12	C11	C10	-0.2(3)	C33	C34	C35	C30	-0.5(4)
C4	C5	C6	O7	98.9(3)	C21A	C22A	C23A	C24A	1.5(4)
C4	C5	C6	C8	-76.5(3)	C15	C16	C17	Fe13	-60.0(2)
C4	C30	C35	C34	-179.5(2)	C15	C16	C17	C18	-0.7(3)

C4	C30	C31	C32	179.9(2)	C11A	C10A	C9A	Fe43	-58.6(2)
C4	C3	C26	F28	61.1(3)	C11A	C10A	C9A	C8A	0.8(3)
C4	C3	C26	F29	-177.7(2)	C16	C15	C14	Fe13	58.9(2)
C4	C3	C26	F27	-56.9(3)	C16	C15	C14	C18	-0.6(3)
C5	N1	C19	C20	170.8(2)	C35A	C30A	C31A	C32A	1.8(4)
C5	N1	C19	C24	-12.2(4)	C35A	C34A	C33A	C32A	0.7(4)
C5	C4	C30	C35	62.5(3)	C17	C18	C14	Fe13	-59.7(2)
C5	C4	C30	C31	-117.6(3)	C17	C18	C14	C15	0.2(3)
C5	C4	C3	N2	-16.0(3)	C10A	C11A	C12A	Fe43	59.1(2)
C5	C4	C3	C26	168.9(2)	C10A	C11A	C12A	C8A	-0.4(3)
C20	C19	C24	C23	2.7(4)	C12A	C8A	C9A	Fe43	59.03(19)
C6	C8	C9	Fe13	-119.5(3)	C12A	C8A	C9A	C10A	-1.0(3)
C6	C8	C9	C10	179.2(3)	C12A	C11A	C10A	Fe43	-58.7(2)
C6	C8	C12	Fe13	120.1(2)	C12A	C11A	C10A	C9A	-0.2(3)
C6	C8	C12	C11	-179.8(2)	C31A	C30A	C35A	C34A	-2.2(4)
C9	C8	C6	O7	-175.3(3)	C31A	C32A	C33A	C34A	-1.1(4)
C9	C8	C6	C5	0.0(4)	C14	C18	C17	Fe13	59.8(2)
C9	C8	C12	Fe13	-58.77(18)	C14	C18	C17	C16	0.3(3)
C9	C8	C12	C11	1.4(3)	C14	C15	C16	Fe13	-59.0(2)
C12	C8	C6	O7	6.0(4)	C14	C15	C16	C17	0.8(3)
C12	C8	C6	C5	-178.6(2)	C25A	C22A	C23A	C24A	-177.5(3)
C12	C8	C9	Fe13	59.27(18)	C25A	C22A	C21A	C20A	177.9(3)
C12	C8	C9	C10	-2.0(3)	C9A	C8A	C12A	Fe43	-59.03(19)
C12	C11	C10	Fe13	-58.59(19)	C9A	C8A	C12A	C11A	0.8(3)
C12	C11	C10	C9	-1.1(3)	C16A	C15A	C14A	Fe43	59.0(2)
C19	N1	C5	C4	177.2(2)	C16A	C15A	C14A	C18A	-0.8(4)
C19	N1	C5	C6	-66.9(3)	C16A	C17A	C18A	Fe43	-60.2(3)
C19	C20	C21	C22	0.2(4)	C16A	C17A	C18A	C14A	0.3(4)
C19	C24	C23	C22	-0.4(4)	C15A	C16A	C17A	Fe43	-60.0(3)
C30	C4	C5	N1	-96.4(2)	C15A	C16A	C17A	C18A	-0.8(4)
C30	C4	C5	C6	146.6(2)	C15A	C14A	C18A	Fe43	61.0(3)
C30	C4	C3	N2	104.1(3)	C15A	C14A	C18A	C17A	0.3(4)
C30	C4	C3	C26	-71.0(3)	C17A	C16A	C15A	Fe43	59.8(3)
C30	C31	C32	C33	-0.3(4)	C17A	C16A	C15A	C14A	1.0(4)

Table S7 Hydrogen Atom Coordinates (Å×10^4) and Isotropic Displacement Parameters (Å^2×10^3) for **2q**.

**Atom**	***x***	***y***	***z***	**U(eq)**
H4	5961.27	2568.26	1954.52	40
H5	5337.01	1117.38	871.21	40
H20	3524.77	4132.46	161.11	43
H9	5834.14	-178.79	1642.61	43

Table S7 Hydrogen Atom Coordinates (Å×10[4]) and Isotropic Displacement Parameters (Å[2]×10[3]) for **2q**.

**Atom**	***x***	***y***	***z***	**U(eq)**
H12	1745.74	-315.5	2125.99	43
H11	2580.18	-1987.33	2586.98	47
H34	9848.4	3582.98	366.7	45
H18	3818.95	-1667.46	314.93	49
H35	7527.81	3525.54	620.46	42
H24A	8658.61	5472.33	5097.77	47
H31	8176.49	2138.18	2095.58	42
H5A	9123.64	5327.89	4143.24	43
H21	2094.11	3459.35	-691.88	46
H4A	8379.09	5775.09	3088.57	44
H32	10501.85	2222.83	1843.23	45
H24	3403.96	352.21	215.86	46
H23A	8573.45	5790.79	6112.35	48
H23	1976.48	-304.05	-639.69	48
H20A	8686.96	9262.23	5118.57	49
H25A	795.51	1832.94	-1454.51	69
H25B	197.39	402.75	-1294.87	69
H25C	1618.52	734.34	-1642.59	69
H10	5091.71	-1868.95	2306.9	47
H33	11331.22	2929.93	978.66	46
H21A	8625.76	9553.1	6133.46	50
H15	2664.35	-4424.56	1377.8	51
H11A	6913.63	1091.01	2968.11	53
H16	592.58	-3450.63	1272.16	54
H35A	11485.78	7605.68	4008.15	48
H17	1301.92	-1768.02	604.69	52
H10A	4367.16	1140.79	3071.22	56
H12A	8482.74	3290.62	3435.34	49
H31A	10081.85	4957.95	2543.62	50
H14	4648.49	-3310.32	799.52	49
H34A	13777	7382.14	3869.19	54
H25D	7967.43	7185.45	7023.22	68
H25E	8344.42	8731.6	7037.92	68
H25F	9582.19	8014.07	7050.96	68
H32A	12393.59	4757.75	2401.04	55
H9A	4320.54	3374.53	3612.68	51
H33A	14216.99	5937.07	3066.25	57
H16A	4267.81	163.23	4315.5	69
H15A	4319.36	2347.78	4875.03	66
H14A	6812.26	3608.45	5101.15	90

Table S7 Hydrogen Atom Coordinates (Å×10^4) and Isotropic Displacement Parameters (Å^2×10^3) for **2q**.

Atom	*x*	*y*	*z*	U(eq)
H17A	6730.96	-31.14	4201.6	93
H18A	8382.54	2151.96	4691.46	134

Crystal structure determination of **2q**

Crystal Data for $C_{56}H_{46}F_6Fe_2N_4O_2$ (M =1032.67 g/mol): triclinic, space group P-1 (no. 2), a = 9.8135(3) Å, b = 10.7419(3) Å, c = 23.2185(4) Å, α = 99.306(2)°, β = 90.018(2)°, γ = 104.952(3)°, V = 2331.33(11) Å^3, Z = 2, T = 100.01(10) K, μ(Cu Kα) = 5.606 mm^{-1}, $Dcalc$ = 1.471 g/cm^3, 27285 reflections measured (7.724° ≤ 2Θ ≤ 157.652°), 9445 unique (R_{int} = 0.0567, R_{sigma} = 0.0513) which were used in all calculations. The final R_1 was 0.0546 (I > 2σ(I)) and wR_2 was 0.1537 (all data).

Refinement model description

Number of restraints - 0, number of constraints - unknown.

Details:

```
1. Fixed Uiso
 At 1.2 times of:
  All C(H) groups
 At 1.5 times of:
  All C(H,H,H) groups
2.a Ternary CH refined with riding coordinates:
 C4(H4), C5(H5), C5A(H5A), C4A(H4A)
2.b Aromatic/amide H refined with riding coordinates:
 C20(H20), C9(H9), C12(H12), C11(H11), C34(H34), C18(H18), C35(H35),
 C24A(H24A), C31(H31), C21(H21), C32(H32), C24(H24), C23A(H23A), C23(H23),
 C20A(H20A), C10(H10), C33(H33), C21A(H21A), C15(H15), C11A(H11A), C16(H16),
 C35A(H35A), C17(H17), C10A(H10A), C12A(H12A), C31A(H31A), C14(H14),
C34A(H34A),
  C32A(H32A), C9A(H9A), C33A(H33A), C16A(H16A), C15A(H15A), C14A(H14A),
 C17A(H17A), C18A(H18A)
2.c Idealised Me refined as rotating group:
 C25(H25A,H25B,H25C), C25A(H25D,H25E,H25F)
```

This report has been created with Olex2, compiled on 2020.11.27 svn.r5f609507 for Rigaku Oxford Diffraction. Please let us know if there are any errors or if you would like to have additional features.

Crystallographic analysis of 6n: A suitable crystal of compound **6n** was selectedand measured on a XtaLAB Synergy, Dualflex, Pilatus 300K diffractometer. The crystal was kept at 99.99(10) K during data collection. Using Olex2,[13] the structure was solved with the XT[14] structure solution program using Intrinsic Phasing and refined with the XL[15] refinement package using Least Squares minimisation. CCDC-2079230 contains the supplementary crystallographic data for this paper. These data can be obtained free of charge from the Cambridge Crystallographic Data Centre via https://www.ccdc.cam.ac.uk/structures/

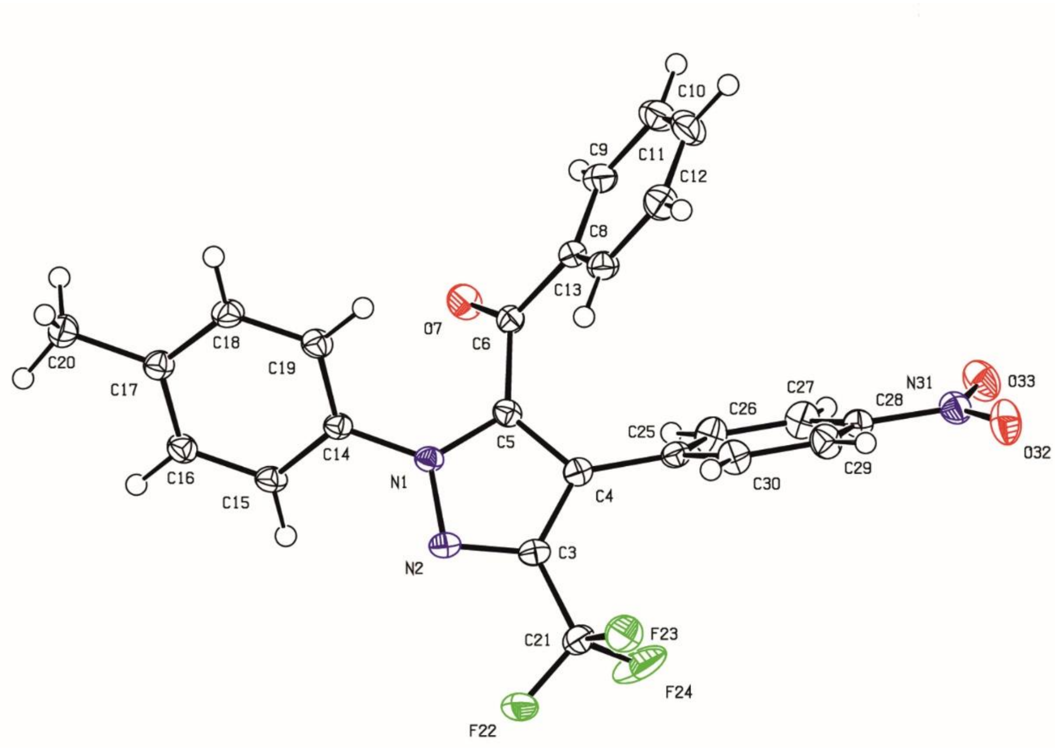

Fig S58. A view of the molecular structure of compound **6n**. Displacement ellipsoids are drawn at the 50% probability level. X-ray data collected at the ambient temperature 100 K.

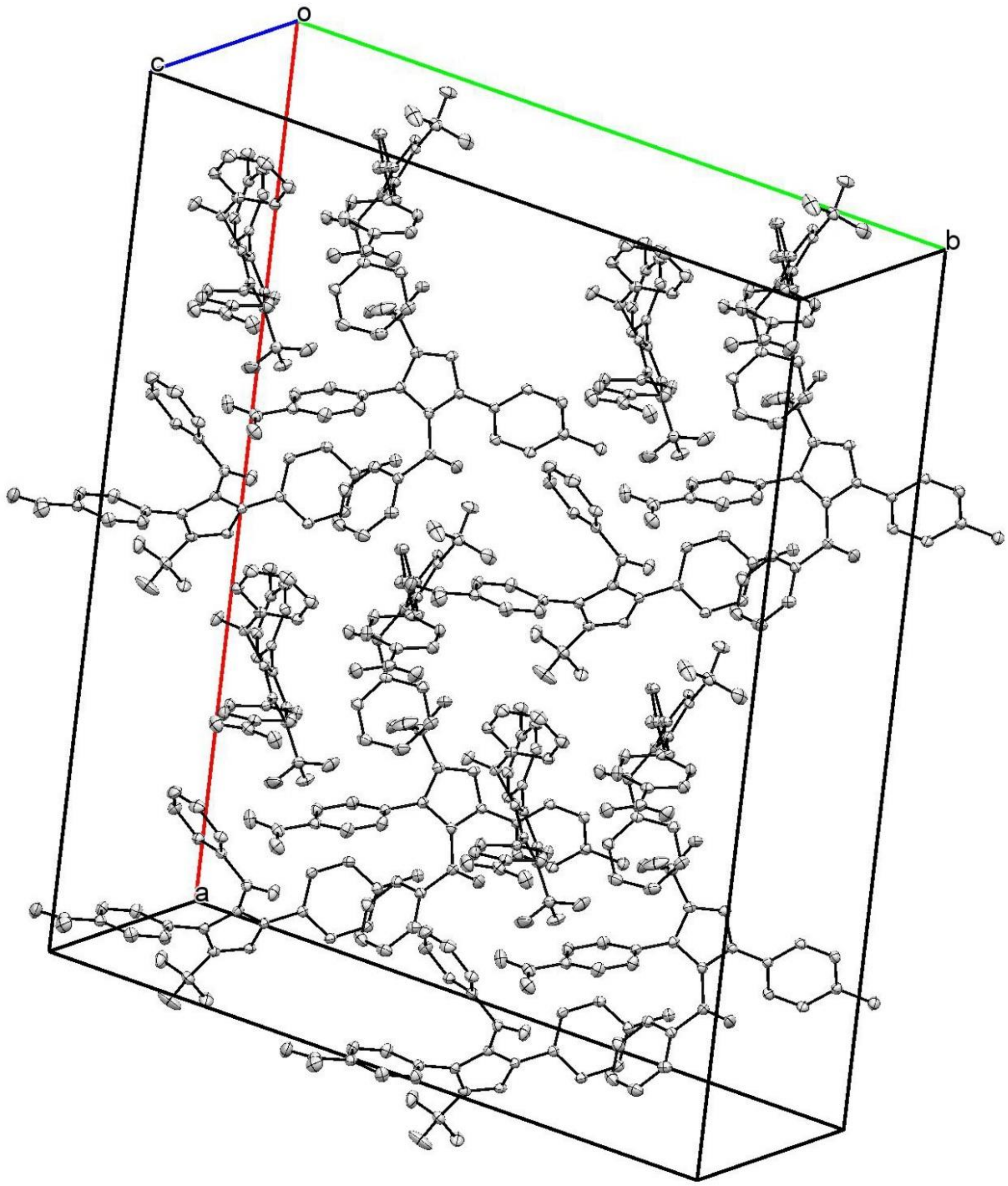

Fig S59. A view of the molecular packing in the structure of compound **6n**.

Table S8 Crystal data and structure refinement for **6n**.

Identification code	GUT-990
Empirical formula	$C_{24}H_{16}F_3N_3O_3$
Formula weight	451.40
Temperature/K	99.99(10)
Crystal system	orthorhombic
Space group	Fdd2

a/Å	33.2382(2)
b/Å	28.8297(2)
c/Å	8.72030(10)
α/°	90
β/°	90
γ/°	90
Volume/$Å^3$	8356.20(12)
Z	16
ρ_{calc}g/cm^3	1.435
μ/mm^{-1}	0.975
F(000)	3712.0
Crystal size/mm^3	0.64 × 0.09 × 0.07
Radiation	Cu Kα (λ = 1.54184)
2Θ range for data collection/°	8.12 to 157.634
Index ranges	-41 ≤ h ≤ 40, -36 ≤ k ≤ 34, -9 ≤ l ≤ 10
Reflections collected	62799
Independent reflections	4226 [R_{int} = 0.0456, R_{sigma} = 0.0135]
Data/restraints/parameters	4226/1/300
Goodness-of-fit on F^2	1.067
Final R indexes [I>=2σ (I)]	R_1 = 0.0249, wR_2 = 0.0655
Final R indexes [all data]	R_1 = 0.0250, wR_2 = 0.0655
Largest diff. peak/hole / e $Å^{-3}$	0.18/-0.18
Flack parameter	0.04(3)

Table S9 Fractional Atomic Coordinates (×10^4) and Equivalent Isotropic Displacement Parameters ($Å^2$×10^3) for **6n**. U_{eq} is defined as 1/3 of of the trace of the orthogonalised U_{IJ} tensor.

Atom	*x*	*y*	*z*	U(eq)
F22	4304.6(3)	4034.1(4)	3056.9(15)	31.3(3)
F23	4672.7(4)	4576.6(4)	3969.4(16)	35.2(3)
F24	4423.7(4)	4071.6(6)	5481.4(16)	50.6(4)
O7	5933.0(4)	2833.7(4)	4887.6(17)	27.3(3)
O33	5552.2(4)	4447.2(5)	11824.3(17)	32.8(3)
O32	5481.0(5)	5116.0(5)	10728.7(18)	32.8(3)
N1	5345.2(4)	3335.6(5)	2907.2(17)	16.5(3)
N2	4990.0(4)	3556.2(5)	2682.6(18)	17.7(3)
N31	5495.4(4)	4690.6(5)	10690.2(19)	23.9(3)
C4	5280.0(5)	3796.3(5)	4917(2)	17.4(3)
C3	4951.4(5)	3828.8(5)	3902(2)	17.7(3)
C25	5338.8(5)	4029.0(6)	6414(2)	18.4(3)
C17	5773.2(5)	2446.7(6)	-594(2)	20.1(4)
C14	5493.6(5)	3028.8(5)	1745(2)	16.7(3)

Table S9 Fractional Atomic Coordinates (×10^4) and Equivalent Isotropic Displacement Parameters (Å^2×10^3) for **6n**. U_{eq} is defined as 1/3 of of the trace of the orthogonalised U_{IJ} tensor.

Atom	*x*	*y*	*z*	U(eq)
C8	6222.9(5)	3572.8(6)	5406(2)	18.7(3)
C5	5528.3(5)	3471.9(5)	4230(2)	17.3(3)
C19	5888.5(5)	3068.6(6)	1237(2)	20.6(4)
C16	5377.0(5)	2418.6(6)	-67(2)	20.4(4)
C6	5906.0(5)	3254.6(6)	4841(2)	18.6(3)
C18	6025.9(5)	2777.4(6)	80(2)	21.9(4)
C28	5444.7(5)	4463.0(6)	9196(2)	20.9(3)
C13	6241.5(5)	4035.8(6)	4938(2)	21.4(4)
C21	4586.1(5)	4125.0(6)	4093(2)	22.8(4)
C15	5234.7(5)	2704.8(6)	1098(2)	19.6(3)
C26	5351.3(6)	3769.6(6)	7760(2)	24.7(4)
C20	5928.6(5)	2130.4(6)	-1829(2)	24.5(4)
C27	5402.6(6)	3986.0(6)	9162(2)	26.1(4)
C30	5383.4(6)	4511.0(6)	6488(2)	22.8(4)
C9	6511.8(5)	3399.4(6)	6419(2)	23.0(4)
C29	5437.2(5)	4730.7(6)	7882(2)	24.2(4)
C12	6544.5(6)	4322.2(7)	5485(2)	27.3(4)
C10	6813.4(6)	3685.4(8)	6961(2)	29.3(4)
C11	6828.7(6)	4146.6(7)	6501(2)	31.4(4)

Table S10 Anisotropic Displacement Parameters (Å^2×10^3) for **6n**. The Anisotropic displacement factor exponent takes the form: $-2\pi^2[h^2a^{*2}U_{11}+2hka^*b^*U_{12}+\ldots]$.

Atom	U_{11}	U_{22}	U_{33}	U_{23}	U_{13}	U_{12}
F22	20.0(5)	32.3(6)	41.7(7)	-8.4(5)	-8.1(5)	7.3(4)
F23	34.8(6)	20.4(5)	50.5(8)	-8.2(5)	-9.6(6)	9.1(4)
F24	36.3(7)	82.1(11)	33.3(7)	17.3(7)	17.3(6)	30.4(7)
O7	31.5(7)	15.7(6)	34.7(8)	-1.2(5)	-8.7(6)	3.6(5)
O33	36.8(8)	40.1(8)	21.4(7)	3.4(6)	-3.0(6)	-6.4(6)
O32	43.9(8)	26.9(7)	27.7(8)	-6.6(6)	2.4(6)	-9.9(6)
N1	14.1(6)	14.5(6)	20.9(7)	0.3(5)	-1.1(5)	-0.4(5)
N2	14.0(6)	17.1(6)	22.0(7)	2.7(6)	0.7(5)	0.4(5)
N31	21.3(7)	29.3(8)	21.0(8)	-1.0(6)	1.6(6)	-5.6(6)
C4	18.0(7)	14.0(7)	20.2(9)	1.5(6)	0.9(7)	-0.6(6)
C3	15.3(7)	16.2(7)	21.5(9)	3.7(6)	1.4(6)	-0.3(6)
C25	15.6(7)	17.6(7)	22.1(9)	-0.3(7)	0.4(6)	2.1(6)
C17	19.9(8)	17.3(8)	23.1(9)	0.5(7)	-0.5(7)	1.0(6)
C14	16.1(7)	15.0(7)	18.9(8)	1.4(6)	-1.1(6)	1.0(6)
C8	15.4(7)	21.7(8)	19.1(8)	-2.9(7)	0.6(7)	1.0(6)

Table S10 Anisotropic Displacement Parameters ($Å^2 \times 10^3$) for **6n**. The Anisotropic displacement factor exponent takes the form: $-2\pi^2[h^2a^{*2}U_{11}+2hka^*b^*U_{12}+\ldots]$.

Atom	U_{11}	U_{22}	U_{33}	U_{23}	U_{13}	U_{12}
C5	17.4(7)	14.4(7)	20.0(9)	0.6(6)	-1.6(6)	-1.5(6)
C19	17.9(8)	17.8(7)	26.0(10)	-0.6(7)	-0.2(7)	-3.7(6)
C16	19.3(8)	19.0(8)	23.0(9)	-0.9(7)	-2.2(7)	-2.0(6)
C6	18.9(8)	17.6(8)	19.3(9)	-1.6(6)	-1.3(6)	2.0(6)
C18	16.3(8)	22.2(8)	27.3(10)	-1.3(7)	2.2(7)	-2.2(6)
C28	17.1(7)	24.3(8)	21.3(9)	-1.7(7)	0.3(6)	-0.8(6)
C13	17.9(8)	21.1(8)	25.4(9)	-0.9(7)	0.2(7)	-0.1(6)
C21	20.7(8)	25.3(8)	22.5(10)	-0.2(7)	1.7(7)	4.3(7)
C15	14.5(7)	19.4(8)	25.0(9)	0.7(7)	-0.8(6)	-2.0(6)
C26	31.6(10)	16.8(8)	25.5(10)	1.7(7)	2.6(7)	0.9(7)
C20	23.7(8)	23.8(9)	26.0(9)	-4.8(7)	2.5(7)	-0.9(7)
C27	32.4(10)	22.8(8)	23.1(10)	4.6(7)	1.5(8)	1.3(7)
C30	28.5(9)	17.5(8)	22.6(10)	4.1(7)	-2.5(7)	1.0(7)
C9	19.2(8)	27.0(9)	22.9(9)	2.5(7)	0.2(7)	2.3(7)
C29	27.7(9)	17.5(8)	27.4(10)	-0.7(7)	-2.3(8)	-1.9(7)
C12	26.3(9)	24.2(9)	31.5(11)	-2.6(8)	2.8(8)	-5.2(7)
C10	22.3(9)	40.0(11)	25.5(10)	1.4(8)	-4.7(7)	-0.5(8)
C11	24.7(9)	41.0(11)	28.6(11)	-5.9(9)	-2.1(8)	-10.6(8)

Table S11 Bond Lengths for **6n**.

Atom	Atom	Length/Å	Atom	Atom	Length/Å
F22	C21	1.327(2)	C17	C18	1.400(2)
F23	C21	1.338(2)	C17	C20	1.503(3)
F24	C21	1.335(2)	C14	C19	1.390(2)
O7	C6	1.217(2)	C14	C15	1.390(2)
O33	N31	1.227(2)	C8	C6	1.481(2)
O32	N31	1.228(2)	C8	C13	1.397(2)
N1	N2	1.3552(19)	C8	C9	1.397(2)
N1	C14	1.433(2)	C5	C6	1.501(2)
N1	C5	1.362(2)	C19	C18	1.390(3)
N2	C3	1.328(2)	C16	C15	1.391(3)
N31	C28	1.469(2)	C28	C27	1.382(2)
C4	C3	1.409(2)	C28	C29	1.382(3)
C4	C25	1.481(2)	C13	C12	1.387(3)
C4	C5	1.383(2)	C26	C27	1.383(3)
C3	C21	1.494(2)	C30	C29	1.382(3)
C25	C26	1.392(3)	C9	C10	1.381(3)
C25	C30	1.399(2)	C12	C11	1.391(3)

Table S11 Bond Lengths for **6n**.

Atom	Atom	Length/Å	Atom	Atom	Length/Å
C17	C16	1.397(2)	C10	C11	1.390(3)

Table S12 Bond Angles for **6n**.

Atom	Atom	Atom	Angle/˚	Atom	Atom	Atom	Angle/˚
N2	N1	C14	119.17(14)	C4	C5	C6	128.87(16)
N2	N1	C5	112.10(14)	C18	C19	C14	119.47(16)
C5	N1	C14	128.56(14)	C15	C16	C17	121.74(16)
C3	N2	N1	104.25(14)	O7	C6	C8	123.63(15)
O33	N31	O32	123.72(17)	O7	C6	C5	119.31(15)
O33	N31	C28	118.50(15)	C8	C6	C5	117.02(14)
O32	N31	C28	117.78(16)	C19	C18	C17	121.26(16)
C3	C4	C25	128.75(15)	C27	C28	N31	118.35(17)
C5	C4	C3	103.64(15)	C29	C28	N31	119.23(15)
C5	C4	C25	127.51(16)	C29	C28	C27	122.42(18)
N2	C3	C4	112.85(14)	C12	C13	C8	120.04(17)
N2	C3	C21	120.40(15)	F22	C21	F23	106.80(15)
C4	C3	C21	126.75(16)	F22	C21	F24	108.04(15)
C26	C25	C4	120.26(15)	F22	C21	C3	112.61(15)
C26	C25	C30	119.45(17)	F23	C21	C3	111.88(14)
C30	C25	C4	120.29(16)	F24	C21	F23	105.80(16)
C16	C17	C18	117.84(16)	F24	C21	C3	111.35(16)
C16	C17	C20	121.63(16)	C14	C15	C16	118.99(16)
C18	C17	C20	120.52(16)	C27	C26	C25	120.43(16)
C19	C14	N1	119.97(15)	C28	C27	C26	118.69(18)
C15	C14	N1	119.28(15)	C29	C30	C25	120.61(17)
C15	C14	C19	120.70(16)	C10	C9	C8	120.09(17)
C13	C8	C6	121.75(16)	C28	C29	C30	118.41(16)
C13	C8	C9	119.73(16)	C13	C12	C11	119.71(18)
C9	C8	C6	118.51(16)	C9	C10	C11	119.94(18)
N1	C5	C4	107.16(14)	C10	C11	C12	120.49(18)
N1	C5	C6	123.63(15)				

Table S13 Torsion Angles for **6n**.

A	B	C	D	Angle/˚	A	B	C	D	Angle/˚
O33	N31	C28	C27	9.6(2)	C25	C30	C29	C28	-0.2(3)
O33	N31	C28	C29	-170.97(16)	C17	C16	C15	C14	0.4(3)
O32	N31	C28	C27	-170.91(16)	C14	N1	N2	C3	-176.49(14)
O32	N31	C28	C29	8.5(2)	C14	N1	C5	C4	175.54(15)

Table S13 Torsion Angles for **6n**.

A	B	C	D	Angle/˚	A	B	C	D	Angle/˚
N1	N2	C3	C4	0.98(18)	C14	N1	C5	C6	-10.6(3)
N1	N2	C3	C21	-178.60(14)	C14	C19	C18	C17	0.5(3)
N1	C14	C19	C18	-178.26(16)	C8	C13	C12	C11	-0.1(3)
N1	C14	C15	C16	177.83(16)	C8	C9	C10	C11	0.2(3)
N1	C5	C6	O7	-46.7(2)	C5	N1	N2	C3	-0.85(17)
N1	C5	C6	C8	135.22(17)	C5	N1	C14	C19	-44.0(2)
N2	N1	C14	C19	130.80(16)	C5	N1	C14	C15	138.35(17)
N2	N1	C14	C15	-46.8(2)	C5	C4	C3	N2	-0.75(18)
N2	N1	C5	C4	0.42(18)	C5	C4	C3	C21	178.80(16)
N2	N1	C5	C6	174.24(15)	C5	C4	C25	C26	-61.9(2)
N2	C3	C21	F22	8.2(2)	C5	C4	C25	C30	117.9(2)
N2	C3	C21	F23	-112.15(18)	C19	C14	C15	C16	0.2(3)
N2	C3	C21	F24	129.69(18)	C16	C17	C18	C19	0.1(3)
N31	C28	C27	C26	179.46(16)	C6	C8	C13	C12	-179.58(17)
N31	C28	C29	C30	-179.08(16)	C6	C8	C9	C10	179.54(17)
C4	C3	C21	F22	-171.36(16)	C18	C17	C16	C15	-0.5(3)
C4	C3	C21	F23	68.3(2)	C13	C8	C6	O7	160.49(18)
C4	C3	C21	F24	-49.8(2)	C13	C8	C6	C5	-21.6(3)
C4	C25	C26	C27	-179.42(17)	C13	C8	C9	C10	0.3(3)
C4	C25	C30	C29	179.80(16)	C13	C12	C11	C10	0.6(3)
C4	C5	C6	O7	125.7(2)	C15	C14	C19	C18	-0.7(3)
C4	C5	C6	C8	-52.4(3)	C26	C25	C30	C29	-0.4(3)
C3	C4	C25	C26	113.9(2)	C20	C17	C16	C15	178.69(17)
C3	C4	C25	C30	-66.3(2)	C20	C17	C18	C19	-179.16(17)
C3	C4	C5	N1	0.18(17)	C27	C28	C29	C30	0.3(3)
C3	C4	C5	C6	-173.22(16)	C30	C25	C26	C27	0.8(3)
C25	C4	C3	N2	-177.30(16)	C9	C8	C6	O7	-18.8(3)
C25	C4	C3	C21	2.3(3)	C9	C8	C6	C5	159.18(16)
C25	C4	C5	N1	176.79(15)	C9	C8	C13	C12	-0.3(3)
C25	C4	C5	C6	3.4(3)	C9	C10	C11	C12	-0.7(3)
C25	C26	C27	C28	-0.6(3)	C29	C28	C27	C26	0.0(3)

Table S14 Hydrogen Atom Coordinates (Å×10^4) and Isotropic Displacement Parameters (Å^2×10^3) for **6n**.

Atom	*x*	*y*	*z*	U(eq)
H19	6063.02	3293.02	1677.04	25
H16	5200.04	2198.17	-515.4	25
H18	6296.54	2803.22	-260.58	26
H13	6046.52	4154.56	4245.04	26
H15	4964.74	2679.16	1445.14	24

Table S14 Hydrogen Atom Coordinates (Å×10[4]) and Isotropic Displacement Parameters (Å[2]×10[3]) for **6n**.

**Atom**	***x***	***y***	***z***	**U(eq)**
H26	5324.45	3441.81	7715.06	30
H20A	6131.37	1921.28	-1395.81	37
H20B	6050.18	2315.84	-2649.04	37
H20C	5705.57	1947.64	-2248	37
H27	5408.78	3810.42	10083.32	31
H30	5376.61	4689.03	5571.14	27
H9	6500.68	3084.34	6736.11	28
H29	5468.25	5057.85	7934.98	29
H12	6557.81	4637.02	5167	33
H10	7010.19	3566.96	7647.07	35
H11	7034.79	4343.34	6883.96	38

Crystal structure determination of **6n**:

Crystal Data for $C_{24}H_{16}F_3N_3O_3$ (M =451.40 g/mol): orthorhombic, space group Fdd2 (no. 43), a = 33.2382(2) Å, b = 28.8297(2) Å, c = 8.72030(10) Å, V = 8356.20(12) Å^3, Z = 16, T = 99.99(10) K, μ(Cu Kα) = 0.975 mm^{-1}, $Dcalc$ = 1.435 g/cm^3, 62799 reflections measured (8.12° ≤ 2Θ ≤ 157.634°), 4226 unique (R_{int} = 0.0456, R_{sigma} = 0.0135) which were used in all calculations. The final R_1 was 0.0249 (I > 2σ(I)) and wR_2 was 0.0655 (all data).

Refinement model description

Number of restraints - 1, number of constraints - unknown.

Details:

```
1. Fixed Uiso
 At 1.2 times of:
  All C(H) groups
 At 1.5 times of:
  All C(H,H,H) groups
2.a Aromatic/amide H refined with riding coordinates:
 C19(H19), C16(H16), C18(H18), C13(H13), C15(H15), C26(H26), C27(H27),
 C30(H30), C9(H9), C29(H29), C12(H12), C10(H10), C11(H11)
2.b Idealised Me refined as rotating group:
 C20(H20A,H20B,H20C)
```

This report has been created with Olex2, compiled on 2020.11.27 svn.r5f609507 for Rigaku Oxford Diffraction. Please let us know if there are any errors or if you would like to have additional features.

5. References

1. G. R. Fulmer, A. J. M. Miller, N. H. Sherden, H. E. Gottlieb, A. Nudelman, B. M. Stoltz, J. E. Bercaw, K. I. Goldberg, *Organometallics* **2010**, *29*, 2176–2179.
2. G. Mlostoń, K. Urbaniak, G. Utecht, D. Lentz, M. Jasiński, *J. Fluorine Chem.* **2016**, *192*, 147–154.
3. A. Wojciechowska, M. Jasiński, P. Kaszyński, *Tetrahedron* **2015**, *71*, 2349–2356.
4. (a) L. Claisen, A. Claparede, *Ber. Dtsch. Chem. Ges.* **1881**, *14*, 2460–2468. (b) J. G. Schmidt, *Ber. Dtsch. Chem. Ges.* **1881**, *14*, 1459–1461.
5. A. T. Davies, J. E. Taylor, J. Douglas, C. J. Collett, L. C. Morrill, C. Fallan, A. M. Z. Slawin, G. Churchill, and A. D. Smith, *J. Org. Chem.* **2013**, *78*, 9243–9257.
6. H. Xie, J. Zhu, Z. Chen, S. Li, Y. Wu, *Synthesis* **2011**, *17*, 2767–2774.
7. P. Grzelak, G. Utecht, M. Jasiński, G. Mlostoń, *Synthesis* **2017**, *49*, 2129–2137.
8. V. M. Muzalevskiy, Z. A. Sizova, V. V. Panyushkin, V. A. Chertkov, V. N. Khrustalev, V. G. Nenajdenko, *J. Org. Chem.* **2021**, *86*, 2385–2405.
9. K. Tanaka, S. Maeno, K. Mitsuhashi, *Chem. Lett.* **1982**, 543–546.
10. A. Touzot, M. Soufyane, H. Berber, L. Toupet, C. Mirand, *J. Fluorine Chem.* **2004**, *125*, 1299–1304.
11. S. L. Jeon, J. H. Choi, B. T. Kim, I. H. Jeong, *J. Fluorine Chem.* **2007**, *128*, 1191–1197.
12. A. E. Ivanova, Ya. V. Burgart, V. I. Saloutin, *Russ. J. Org. Chem.* **2018**, *54*, 1265–1267.
13. O. V. Dolomanov, L. J. Bourhis, R. J Gildea, J. A. K. Howard, H. Puschmann, *J. Appl. Cryst.* **2009**, *42*, 339–341.
14. G. M. Sheldrick, *Acta Cryst.* **2015**, *A71*, 3–8.
15. G. M. Sheldrick, *Acta Cryst.* **2008**, *A64*, 112–122.
